# Structure over States: Planarity, Not Energy, Dictates Photoactivation in Ru(II) PACT Agents

**DOI:** 10.1021/jacs.5c12226

**Published:** 2025-09-22

**Authors:** Matthijs L. A. Hakkennes, Irene Regeni, Yurii Husiev, Valeriia D. Andreeva, Maxime A. Siegler, Francesco Buda, Sylvestre Bonnet

**Affiliations:** † Leiden Institute of Chemistry, 4496 Leiden University, P.O. Box 9502, 2300 RA Leiden, The Netherlands; ‡ Institut de Science et d’Ingénierie Supramoléculaires (ISIS), 129648 Strasbourg University, Strasbourg 67083, France; § Department of Chemistry, 1466 Johns Hopkins University, 3400 N Charles Street, Baltimore, Maryland 21218, United States

## Abstract

Photoactivated chemotherapy (PACT) employs light to precisely control the activity of prodrugs, enabling spatial and temporal regulation of therapeutic effects while minimizing systemic toxicity. Transition-metal complexes, particularly Ru­(II) polypyridyl compounds, have emerged as promising PACT agents due to their ability to undergo photodissociation via triplet excited states. However, rationalizing and predicting photosubstitution quantum yields remain challenging due to complex excited-state dynamics, solvent effects, and limitations of traditional modeling approaches. In this study, we synthesized nine Ru­(II) complexes incorporating a monodentate thioether ligand, a terpyridine ligand, and various bidentate polypyridyl ligands. Upon red-light irradiation in aqueous solution, these complexes showed selective photosubstitution of the thioether by an aqua ligand. Despite similar absorption properties, these compounds exhibited markedly different photosubstitution quantum yields that static DFT calculations failed to explain. These modeling difficulties prompted us to develop a novel triplet-state molecular dynamics protocol using the GFN-xTB method, explicit solvation, and enhanced sampling techniques. Our approach enabled full simulations of the ligand exchange process on the triplet hypersurface and comparison to experimental data. It revealed that both *cis* and *trans* substitution of the thioether by a water molecule is possible; it was also able to distinguish between photoactive and photoinactive compounds. Unexpectedly, we found that the deviation from planarity of the bidentate ligand, rather than the energy levels of the different triplet excited states (^3^MLCT, ^3^MC) involved in the photosubstitution reaction, was the primary determinant of photosubstitution efficiency, as it promoted access to the *trans* photosubstitution pathway. Furthermore, our simulations uniquely identified whether dissociative, interchange, or associative mechanisms governed the reactivity of each complex. These results provide for the first time mechanistic insights into Ru­(II)-based photosubstitution reactions in a solvent and offer a practical, scalable computational tool for designing next-generation PACT agents with optimized light-responsive properties.

## Graphical abstract

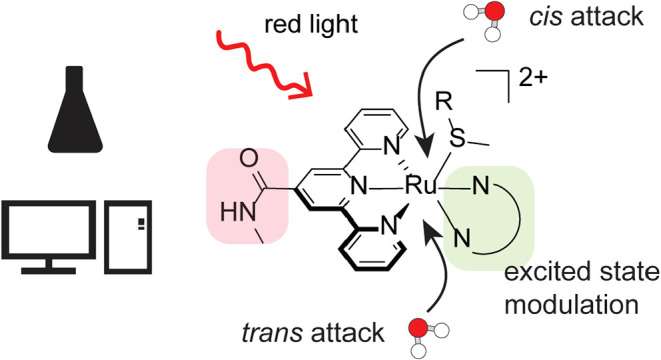

## Introduction

Light is a powerful tool to control spatially and temporally the activity of a drug.^1–4^ A widely adopted strategy in photoactivated drugs involves covalently attaching a bioactive inhibitor to a photocleavable protecting group to afford a nontoxic prodrug that can distribute in the body without side effects. Its biological action is recovered upon local light irradiation, which removes the protecting group. This strategy has been extensively investigated in cancer therapy, where it is known as photoactivated chemotherapy (PACT).^5–12^ To optimize the efficacy of prodrug activation, two particularly important criteria need to be met: the compound needs to absorb light in the red or near-infrared (NIR) region of the spectrum to efficiently penetrate human tissue;^13–16^ in addition, the quantum yield of the bond-cleavage reaction needs to remain high enough at such high wavelengths.^17,18^

A family of molecular compounds that have often been used for PACT are d6 transition-metal-based complexes, such as those based on ruthenium­(II), as they can trigger a bond-cleavage photoreaction once excited on their triplet hypersurface. In these systems, an electron is typically promoted photochemically from a nonbonding t_2g_ orbital localized on the metal center into an empty antibonding orbital (π*) located on a conjugated polypyridyl ligand, thereby forming a singlet metal-to-ligand charge transfer (^1^MLCT) excited state. The complex then efficiently undergoes a spin flip, as the intersystem crossing (ISC) rate is strongly increased by spin–orbit coupling.^19^ The spin flip results in the formation of a ^3^MLCT state. Once in this state, dissociation of one of the ligands in the first coordination sphere may occur if the π-back-bonding between the metal and the coordinating ligand is sufficiently reduced, compared to the ground state.^20–23^ This mechanism is commonly observed with weakly binding ligands, such as nitriles, and it can be influenced by the *trans* effect in the excited state.^20^ For more strongly coordinating ligands, such as pyridines or thioethers, thermal activation of the electron localized on the ligand to the antibonding e_g_ orbital located on the metal is required for photodissociation to occur. The resulting dissociative excited state is called a metal-centered state (^3^MC), which results in elongation of the metal ligand bond. It has been postulated that photosubstitution reactions of bidentate ligands can proceed through two distinct ^3^MC states. Either the d_z^2^_-like orbital is occupied, forming the so-called ^3^MC_trans_ state, or the d_x^2^-y^2^_ orbital is occupied, forming the ^3^MC_cis_ state.^24–27^ Ultimately, the ligand substitution concludes with one or two solvent molecule(s) replacing the photosubstituted ligand in the first coordination sphere of the metal center, leading to the formation of the triplet product state (^3^P), which finally decays in the singlet ground state of the photosubstituted product (see Figure 1C).^28^

**Figure 1:**
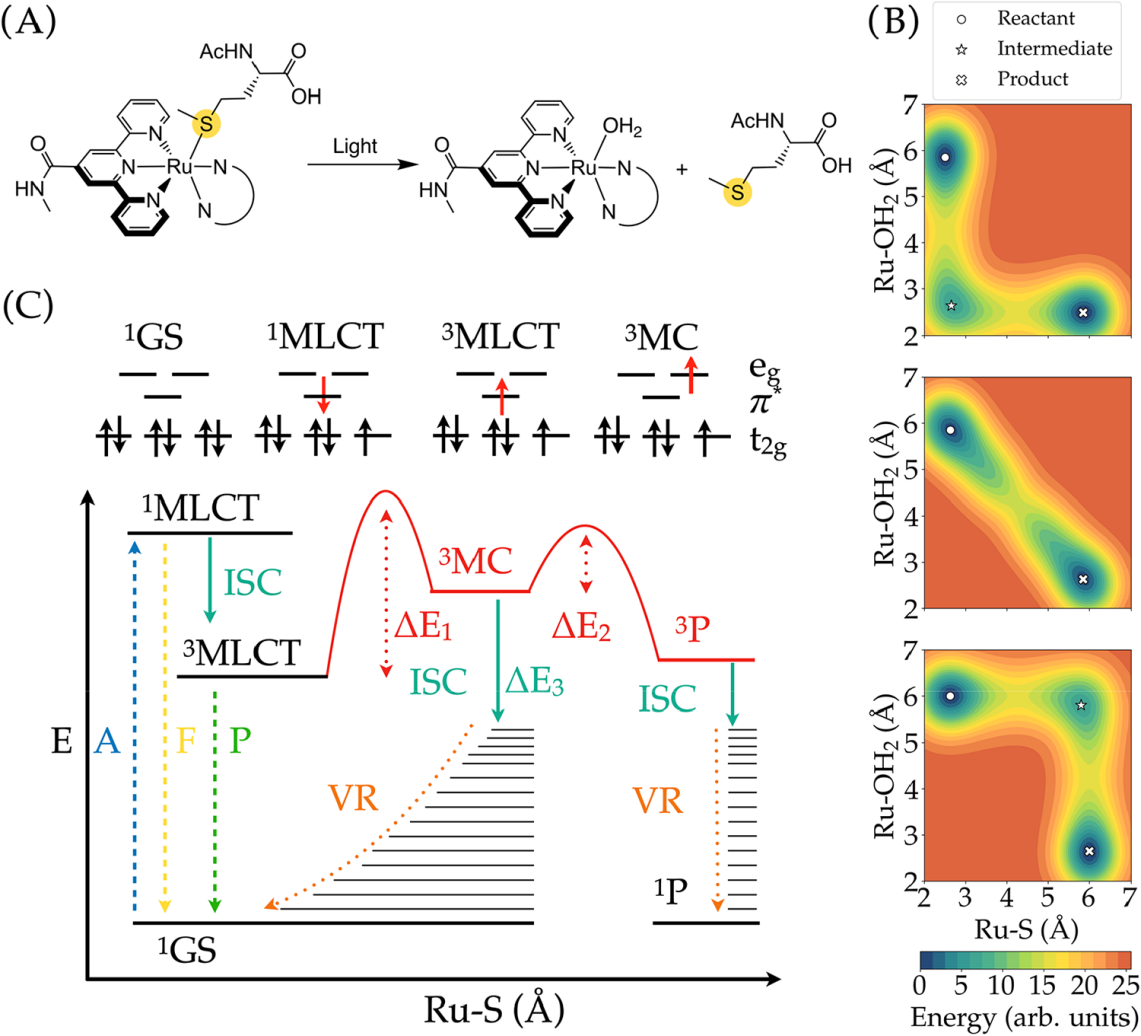
(A) Schematic representation of the photosubstitution reaction studied in this work. (B) Schematic 2D surface of the triplet energy surface of associative (top panel), interchange (middle panel), and dissociative (bottom panel) photosubstitution mechanism. (C) Jablonski diagram of the photosubstitution reaction, accompanied by the orbital diagram of the different states involved in the photosubstitution reaction. The following photochemical processes are indicated: the blue line represents absorption (femtoseconds, fs); the yellow line indicates fluorescence (nanoseconds to milliseconds, ns–ms); the green line shows phosphorescence (milliseconds to seconds, ms–s); the cyan-blue line corresponds to intersystem crossing (nanoseconds, ns); the orange dotted line denotes vibrational relaxation (picoseconds, ps) time scale; the red line represents photosubstitution (ns to ms, ns-ms); and the thin black lines depict vibrational levels.

The wavelength of the light at which photosubstitution can be triggered essentially depends on the level of the ^1^MLCT states, which is correlated to that of the ^3^MLCT state. To predict photosubstitution quantum yields three factors are usually considered as key: the ^3^MLCT^3^MC energy barrier,^29^ the ^3^MC–^3^P energy barrier, and the singlet–triplet energy difference (E_T‑S_) along the reaction path.^30–32^ Until recently, the latter two have been essentially ignored, and it is generally considered that tuning the ^3^MLCT and ^3^MC energy levels is enough to rationalize and tune the values of the photosubstitution quantum yields. For example, photosubstitution reactions are known to become faster when the temperature increases, an effect usually attributed to the thermal energy requirements for reaching the ^3^MC states from the photogenerated ^3^MLCT states.^33,34^ However, this simplification overlooks the critical role of solvent effects on the reaction mechanism, which are known to strongly influence the experimentally measured photosubstitution reaction rates and thermal barriers. Moreover, most theoretical studies aimed at explaining the dynamics of photosubstitution face important methodological limitations. In some cases, the system is being examined using a level of theory that is too low, such as harmonic-potential-based force fields that fail to accurately describe bond breaking. Alternatively, dynamics simulations are often too short due to the computational limits of capturing a full ligand exchange process occurring on a nanosecond to microsecond time scale.^35^ Overall, the only remaining option is to use static DFT calculations focusing on one complex molecule with at most one or two explicit solvent molecules.^18,36,37^

Recently, our group used such static DFT modeling to demonstrate that what happens after ^3^MC formation was at least as important as what happens before, in particular, if one wants to explain the influence of the solvent on the thermal barrier to photosubstitution reactions. However, the necessary simplifications of photosubstitution models fail to predict the value of the thermal barrier to the photoreaction, as well as the ligand exchange mechanism, which may be dissociative, interchange, or associative.^38,39^ In the dissociative mechanism (Figure 1B bottom), the photocleaved ligand first fully detaches from the metal complex, forming a coordinatively unsaturated intermediate. A solvent molecule then interacts with that intermediate to establish a new coordination bond. The interchange mechanism (Figure 1B middle), by contrast, involves a pre-equilibrium between the incoming solvent molecule and the ruthenium complex, leading to the simultaneous exchange of positions between the incoming solvent molecule and the leaving ligand without the formation of a stable intermediate characterized by a local minimum on the triplet or singlet hypersurface. Finally, the associative mechanism (Figure 1B top) begins with the solvent molecule binding to the metal atom, while the photocleavable ligand remains attached, forming a stable heptacoordinated intermediate. The leaving ligand then dissociates to complete the reaction and recover the solvate adduct. These mechanisms are best understood as part of a continuum rather than discrete categories. Interchange-associative pathways exhibit an associative mode of interaction without forming a stable intermediate, while interchange-dissociative pathways proceed through a dissociative activation process, likewise without the formation of an isolable intermediate.^39,40^ Although ruthenium polypyridyl octahedral complexes possess a fully solvated first coordination sphere, it has been noted that a long ^3^MLCT lifetime and significant charge separation can favor an associative or interchange-associative mechanism, as these factors increase the time available for an incoming ligand to bind and enhance the electrostatic attraction to the metal center.^41^ Up to now, the photosubstitution mechanisms, as well as predicting the value of the photosubstitution quantum yields and of the thermal barriers experimentally observed, have remained beyond any modeling approach.

To solve this knowledge gap, gain insights into the mechanism of photosubstitution reactions, and design better ruthenium-based photocages sensitive to low-energy visible light, a new molecular dynamics approach was needed with explicit solvation, a simple reaction coordinate, and the ability to model triplet excited states during nanosecond time scales. To be able to compare modeling results to the experimental reality, and considering the well-documented ability of thioether ligands to be photosubstituted from ruthenium polypyridyl complexes,^36,42–49^ we first prepared a series of 9 analogous ruthenium polypyridyl compounds where a monodentate N-acetyl-l-methionine ligand (AcMet, Figure 1A) was bound to different ruthenium-based photocages. In order to study how fast these complexes react under red-light (630 nm) irradiation, both experimentally and theoretically, we designed our compounds based on three principles: (i) a [κ^3^, κ^2^, κ^1^] coordination mode for the ruthenium complex allows to use a simple reaction coordinate, i.e., the Ru–S distance, to define the advancement of the photosubstitution reaction; (ii) an amide substituent in the 4′ position of a 2,2′:6′,2″-terpyridine (tpy) ligand stabilizes the ^3^MLCT excited states of ruthenium-thioether complexes of the type [Ru­(tpy)­(bpy)­(RSR′)]^2+^ (bpy = 2,2′-bipyridine), which accelerates red-light-triggered photosubstitution;^50^ and (iii) extended π-conjugation of polypyridyl ligand shifts light absorption of its ruthenium complex toward the red region of the spectrum, hence increasing the molar absorption coefficient of the complex at 630 nm.^51^ The choice of the different spectator ligands in this series of complexes was initially driven by our interest in studying the electronic effects of the ligands on the energies of the ^3^MLCT and ^3^MC excited states of their Ru complexes. Two different tridentate ligands were finally used: a 2,6-di­(1,8-naphthyridin-2-yl)­pyridine (dpn) in complex [Ru­(dpn)­(dppz)­(AcMet)]­Cl_2_ ([Ru1]­Cl_2_, dppz = dipyrido-[3,2-a:2′,3′-c]-phenazine), and 4′-methylamide-2,2′:6′,2″-terpyridine (tpa) in complexes [Ru­(tpa)­(N–N)­(AcMet)]­Cl_2_ ([Ru2]­Cl_2_-[Ru9]­Cl_2_), where N–N were different bidentate ligands with electron-donating, electron-withdrawing, and extended conjugated rings (shown in Figure 2). In this series of thermally stable complexes, one was only photosubstitutionally inactive, while 5 showed some photosubstitution properties under red-light irradiation (630 nm), and three were remarkably fast. Static DFT analysis of the energy levels of the ^3^MLCT and ^3^MC excited states did not allow to rationalize these experimental observations. We hence developed a new computational protocol, which we hereafter refer to as AQUAMEPTH (Adaptive QM/MM with Umbrella Sampling and Metadynamics for Explicit Photosubstitution on Triplet Hypersurface). This protocol is based on the semiempirical GFN1-xTB method and is designed to explore the triplet-state potential energy surface of metal complexes in the presence of explicit solvent molecules. AQUAMEPTH proved efficient in uncovering two distinct reaction pathways for photosubstitution, characterized by either a *trans* or *cis* attack of the incoming aqua ligand on the complex. Furthermore, it enabled us to distinguish, for each compound, associative, interchange, and dissociative substitution mechanisms. Most notably, our results reveal that the planarity of the bidentate ligand, rather than electronic effects, correlates most strongly with the observed variations in photosubstitution quantum yields.

**Figure 2:**
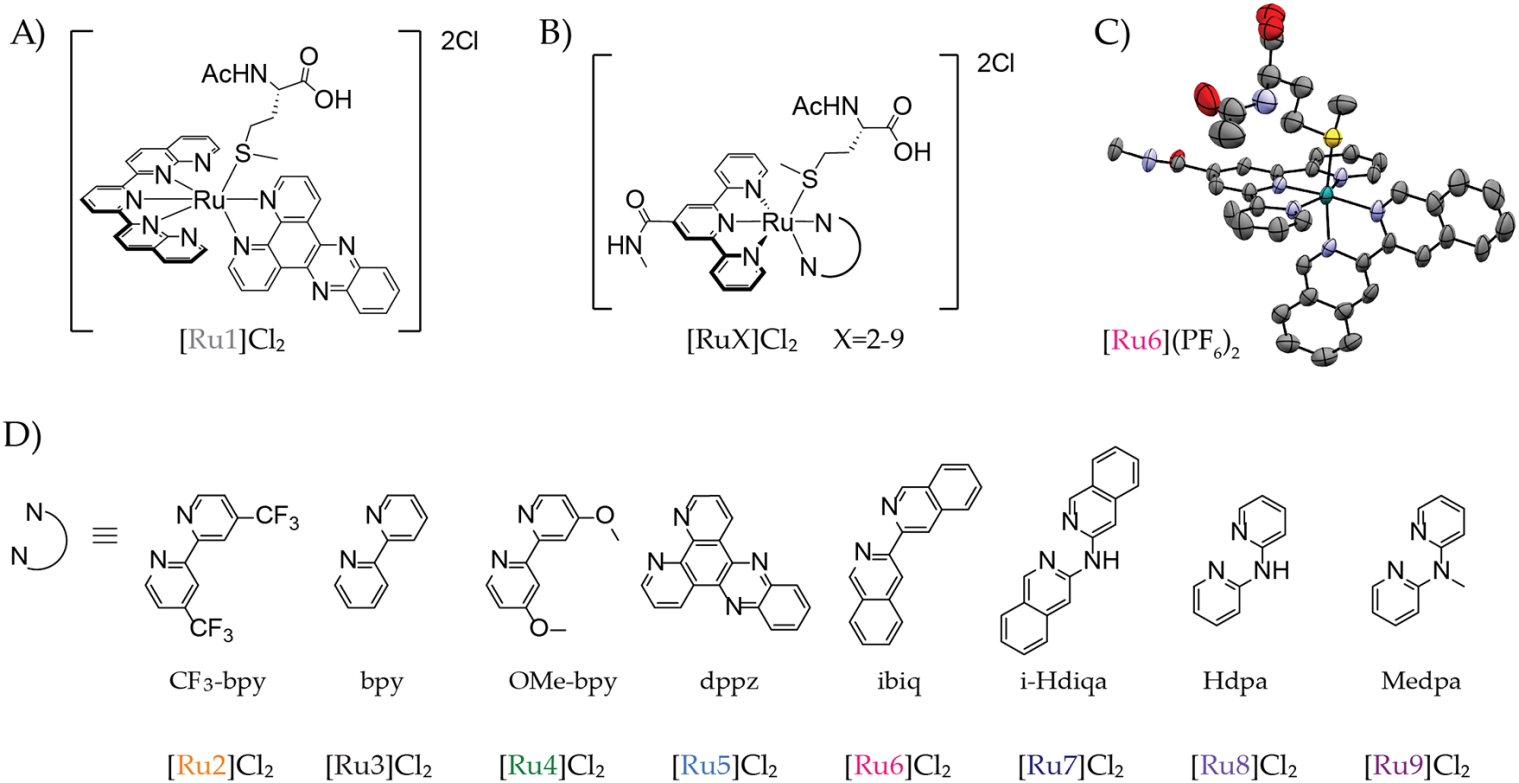
(A, B, D) Chemical formulas and numbering of the ruthenium polypyridyl complexes studied in this work. (C) Crystal structure of compound [Ru6]­(PF_6_)_2_.

## Results

### Synthesis

Complex [Ru1]­Cl_2_ was prepared in a two-step synthesis starting from tridentate ligand dpn and precursor RuCl_3_·3H_2_O heated together to reflux in a deoxygenated mixture in EtOH/H_2_O, to yield [Ru­(dpn)­Cl_3_]. Two chloride ligands were then exchanged for the bidentate ligand dppz in the presence of triethylamine.^3^ In the final step, [Ru­(dpn)­(dppz)­Cl]Cl and N-acetyl-l-methionine (AcMet) were dissolved in deionized water and heated to reflux to afford [Ru1]­Cl_2_. Complexes [Ru2]­Cl_2_-[Ru9]­Cl_2_, were synthesized in a three-step synthesis starting from the amide-functionalized terpyridine ligand (tpa) and the dichloro­(p-cymene)­ruthenium­(II) dimer precursor, similarly to reported procedures.^16^ The product of this reaction, the tpa-ruthenium­(II) dimer [Ru­(tpa)­Cl_2_]_2_, was reacted with the corresponding bidentate (*N–N*) ligand to yield the chloride precursor [Ru­(tpa)­(*N–N*)­(Cl)]­Cl. In the final step, the chloride ligand was exchanged for AcMet by reflux in deionized water. The complexes were first isolated as their PF_6_^–^ salt, purified with flash chromatography on silica gel, and finally, the counterion was exchanged for a Cl^–^ on an anion-exchange resin. All reactions were carried out under the exclusion of light and under an inert atmosphere. Full characterization of the compounds was performed by NMR and UV–vis spectroscopy, ESI mass spectrometry, HPLC, and elemental analysis (see Supporting Information). The crystal structure of [Ru6]­(PF_6_)_2_ (Figure 2C) was determined by single-crystal X-ray diffraction. The ^1^H NMR assignments of [Ru1]­Cl_2_-[Ru9]­Cl_2_ were determined using ^1^H–^1^H COSY and NOESY NMR. While complexes [Ru2]­Cl_2_ -[Ru6]­Cl_2_ showed a symmetric tridentate ligand (see Supporting Information), for [Ru7]­Cl_2_-[Ru9]­Cl_2_ the protons of the tpa ligand split into two sets due to the nonperpendicular arrangement of the bidentate ligand. Notably, at 298 K, the ^1^H NMR spectrum of [Ru9]­Cl_2_ appeared heavily broadened, while the peaks of the tpa ligand remained invisible. Variable temperature ^1^H NMR experiments revealed interconversion dynamics of the *N*-methyl-*N*-(pyridin-2-yl)­pyridin-2-amine (Medpa) ligand and confirmed the coalescence temperature to be between 288 and 298 K (Figure S56), thus explaining the broad signals observed for [Ru9]­Cl_2_ at room temperature. As observed earlier,^52^ this effect is likely due to the nonplanarity of the Medpa chelate coupled to steric clash with the thioether ligand. Altogether, both effects combine to remove the plane of symmetry present in [Ru3]­Cl_2_, for example, thus generating in the presence of the chiral, enantiomerically pure thioether ligand of [Ru9]­Cl_2_, two interconverting diastereomers.

### Photochemistry

The absorption and emission properties of complexes [Ru1]­Cl_2_-[Ru9]­Cl_2_ were first measured in water and are reported in Table 1, Figures 3 and S62. [Ru1]­Cl_2_, as expected from the literature, absorbed in the NIR region of the spectrum.^3^ [Ru2]­Cl_2_-[Ru9]­Cl_2_ were characterized by ^1^MLCT transitions between 400 and 700 nm with absorption maxima around 490 nm. Compared to the reported compound [Ru­(tpy)­(bpy)­(AcMet)]^2+^, which does not have the amide auxochromic group on the terpyridine ligand and has an absorption maximum at 450 nm,^53^ [Ru2]­Cl_2_-[Ru9]­Cl_2_ were all characterized by a bathochromic shift of their absorption maximum. Moreover, the shift was consistent with the electron-donating and -accepting capabilities of the bidentate ligands: the larger the electron-donating nature of the ligand, the larger the shift of the ^1^MLCT absorption maximum to a higher wavelength. This trend was even more striking when the emission properties of the different complexes were observed (Figure 3B). Indeed, the emission maxima spanned a range of more than 70 nm in this series of complexes, from 632 nm for [Ru2]­Cl_2_ to 700 nm for [Ru8]­Cl_2_, providing direct insight into the energy levels of the emissive ^3^MLCT excited states of these complexes. These emission data suggested that this series of ruthenium complexes offered a unique opportunity to test the influence of the ^3^MLCT energy level and all quantities related to it (i.e., the ^3^MLCT-^3^MC barrier) on the photochemical properties of these complexes.

**Figure 3:**
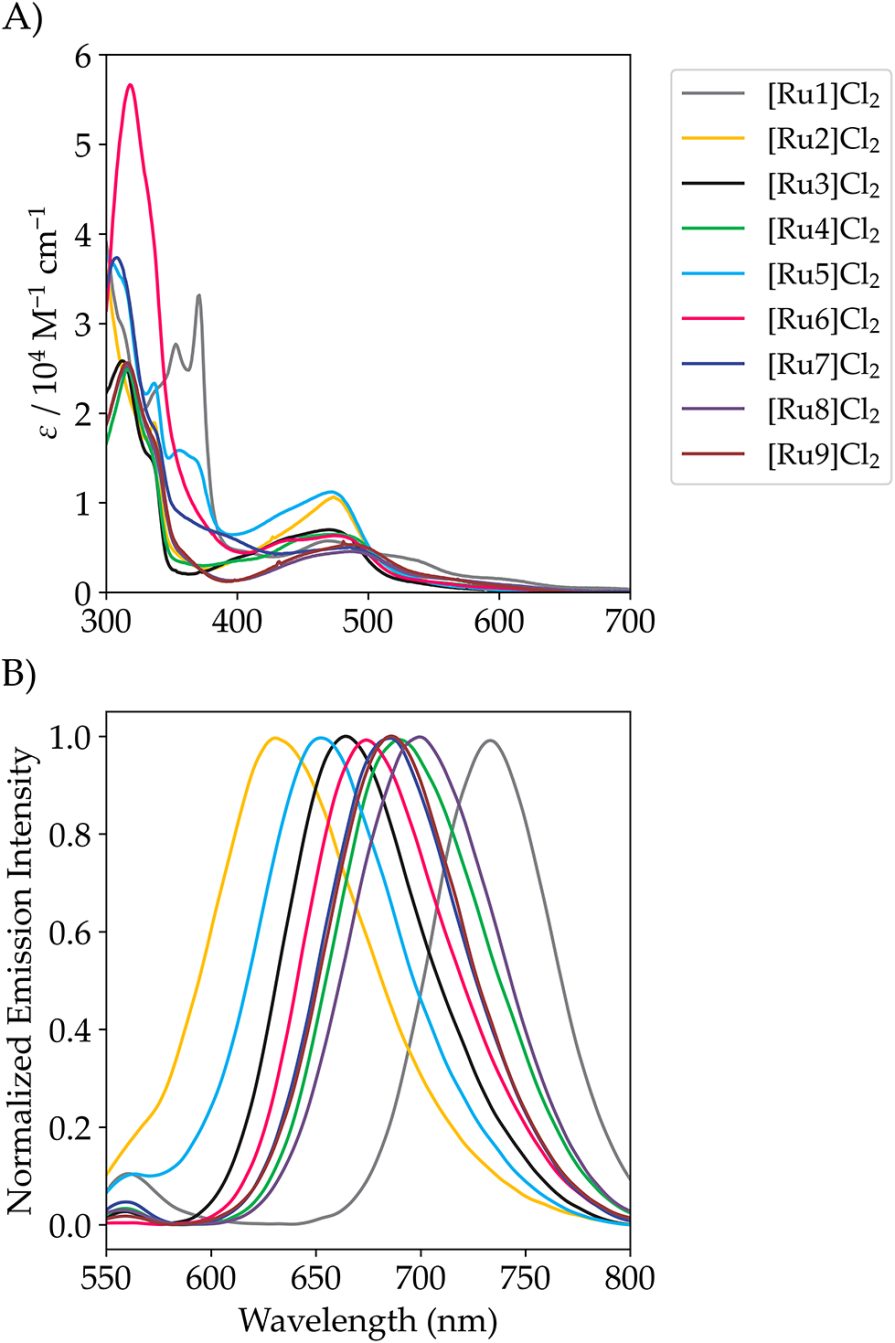
(A) Absorption and (B) normalized emission spectra (λ_ex_ = 480 nm) of complexes [Ru1]­Cl_2_-[Ru9]­Cl_2_ in Milli-Q water.

**Table 1:** Photophysical Data for the Complexes Reported in This Study: Maximum of Absorption (λ_max_ Abs), Maximum of Emission (λ_max_ Em), Extinction Coefficient (ε), Photosubstitution (Φ) Quantum Yield and Reactivity (ξ) with Green (515) and Red (625) Light, Emission Quantum Yield (Φ_P_) and Singlet Oxygen Quantum Yield (Φ_1O2_) Measured with a 450 nm Laser

Complex	λ_max_ Abs (nm)	λ_max_ Em (nm)	ε_515_ (cm^–1^ M^–1^)	Φ_515_	ξ_515_ (cm^–1^ M^–1^)	ε_625_ (cm^–1^ M^–1^)	Φ_625_	ξ_625_ (cm^–1^ M^–1^)	Φ_P_	Φ_1O2_
**[Ru1]Cl**_**2**_	490	733	4140			1018	**-**	-	0.00146	0.045
**[Ru2]Cl**_**2**_	473	632	3040	**0.0009**	3	76	**0.0005**	0.04	0.00011	<0.001
**[Ru3]Cl**_**2**_	470	664	1850	**0.0010**	2	26	**0.0061**	2	0.00104	<0.001
**[Ru4]Cl**_**2**_	477	691	2880	**0.0004**	1	355	**0.0005**	0.2	0.00249	<0.001
**[Ru5]Cl**_**2**_	471	652	2830	**0.0007**	2	32	**0.0025**	0.08	0.00178	<0.001
**[Ru6]Cl**_**2**_	475	676	2360	**0.0008**	2	279	**0.0008**	0.2	0.00196	<0.001
**[Ru7]Cl**_**2**_	485	687	3180	**0.0056**	18	509	**0.0067**	3.4	0.00101	0.027
**[Ru8]Cl**_**2**_	487	700	3120	**0.0063**	20	580	**0.0091**	5.3	0.00109	0.019
**[Ru9]Cl**_**2**_	487	688	3610	**0.0043**	16	329	**0.0077**	2.5	0.00145	0.025

In a second step, the photosubstitution properties of the metal complexes were investigated upon red (625 nm) and green light (515 nm) irradiation in water at 25 °C. For biological applications, the red-light photosubstitution reactivity is more interesting, but the photoreactivity under green light irradiation is easier to measure as molar coefficients are higher at green wavelengths than for red light, which minimizes errors on photosubstitution quantum yields. First, complex [Ru1]­Cl_2_ neither showed any spectral evolution upon green light irradiation in water, nor upon green- or red-light irradiation in acetonitrile, indicating that this complex was not photoactive at room temperature (Figure S70). This surprising result was confirmed by mass spectrometry before and after irradiation, which showed only the *m*/*z* = 455.1 peak of the starting complex (calcd 455.09, see Figure S70d–f). All other complexes [Ru2]­Cl_2_-[Ru9]­Cl_2_ were found to be photoreactive upon green- or red-light irradiation in water. The nature of this photoreaction was first demonstrated by mass spectrometry analysis of solutions of each complex in Milli-Q, either kept in the dark or irradiated with light. While solutions kept in the dark for 24 h showed only the *m*/*z* peaks of the starting complex (Figures S80–S90), identical solutions irradiated with green or red light showed the disappearance of the parent ion and the appearance of a new peak corresponding to the substitution of the thioether ligand by a water molecule, strongly suggesting the occurrence of ligand photosubstitution (Figure S71c–78c). Following the time evolution of the ^1^H NMR spectrum of a D_2_O solution of [Ru5]­Cl_2_ or [Ru9]­Cl_2_ (as prototypical examples) confirmed previous reports that complexes of this type release their thioether ligand (here, AcMet) under visible light irradiation, but not in the dark (Figures S88–S91): the coordinated acetyl and thiomethyl singlets (CH_3_) near 1.2–1.4 and 1.8 ppm gradually disappeared to be replaced by the corresponding peaks of the free AcMet ligand in the ∼2.0 ppm region. Then, using UV–Vis spectroscopy allowed both to redemonstrate the good thermal stability of all photosensitive compounds [Ru2]­Cl_2_-[Ru9]­Cl_2_ in the dark (Figure S79) and to quantify their photosubstitution reactivity under green (Φ_515_) and red (Φ_625_) light irradiation. Since the AcMet starting complex and the photosubstituted aqua product have different absorption properties, the change in the absorption spectrum of the solution upon light irradiation and the well-defined isosbestic points indicative of a one-step reaction can be used to establish the concentration profiles of each photoreaction using the Glotaran software package and from there to calculate the photosubstitution quantum yields Φ_515_ and Φ_625_ (see Table 1 and the Experimental Part).^54^ A prototypical example of the time evolution of the UV–vis spectrum of [Ru3]­Cl_2_ under red-light irradiation is shown in Figure 4A, while Figure 4B shows the kinetics of all red-light reactions for comparison. All data sets are provided in Figures S71–S78. Finally, to obtain a full picture of the photoreactivity of all compounds, the singlet oxygen generation quantum yields (Φ_Δ_) and the phosphorescence quantum yields (Φ_P_) were also determined: all compounds (including [Ru1]­Cl_2_) showed low triplet emission (Φ_P_ < 0.003) and negligible ^1^O_2_ generation quantum yields (Φ_Δ_ < 0.05). All of the photophysical properties are summarized in Table 1.

**Figure 4:**
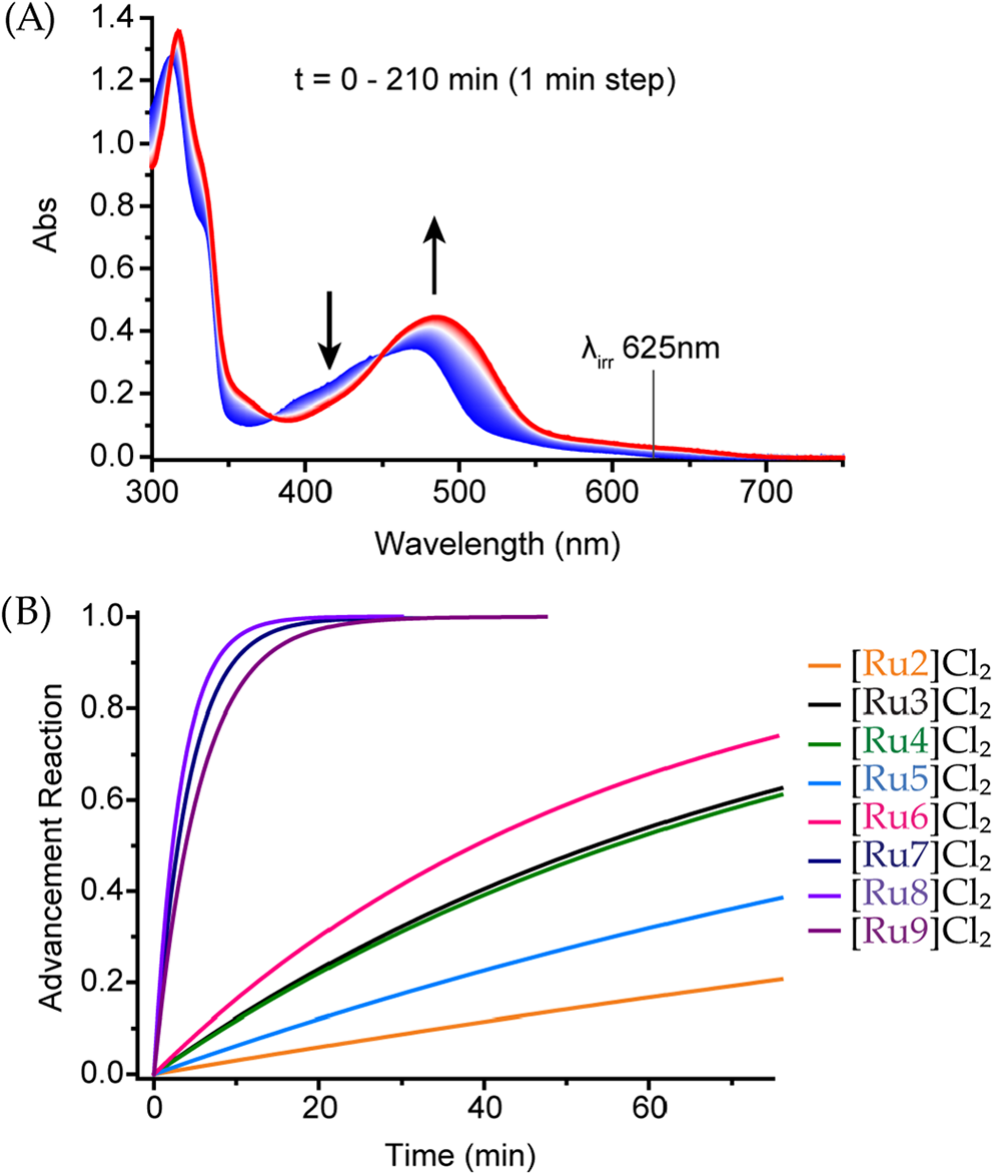
Light-driven ligand photosubstitution. (A) Evolution of absorption spectra upon irradiation at 625 nm for complex [Ru3]­Cl_2_, taken as an example. (B) Advancement of the reaction for photoreactive complexes [Ru2]­Cl_2_-[Ru9]­Cl_2_.

Correlating even qualitatively the structure of complexes [Ru1]­Cl_2_-[Ru9]­Cl_2_ to the rate of red-light photosubstitution was not straightforward. A higher photoreactivity ξ_625_ can be a consequence of better red-light absorption (hence a higher molar extinction coefficient ε_625_), a higher photosubstitution quantum yield Φ_625_, or both. In this series of complexes, both ε_625_ and Φ_625_ values clearly depended on the structure of the complex. For instance, [Ru4]­Cl_2_ exhibited a 5-fold increase in ε_625_ compared with [Ru2]­Cl_2_ (355 vs 76 cm^–1^ M^–1^), yet their Φ_625_ values were nearly identical (∼0.0005). Conversely, while [Ru6]­Cl_2_ and [Ru9]­Cl_2_ had comparable ε_625_ values (279 vs 329 cm^–1^ M^–1^), the latter showed a 10-fold increase in Φ_625_ with respect to the former (0.0008 vs 0.0077, respectively). Overall, [Ru1]­Cl_2_ stood out for its absence of photoreactivity, as well as [Ru7]­Cl_2_-[Ru9]­Cl_2_, which showed unexpectedly higher reactivity because of better red-light absorption and higher photosubstitution quantum yields.

### Static DFT Calculations

To rationalize these experimental results and better understand the molecular design principles influencing the photosubstitution quantum yields in this series of compounds, we first followed standard and simple modeling methods based on static DFT calculations. As it is often argued that the experimentally observed thermal barrier to the photochemical reaction is due to the transition from the ^3^MLCT to the ^3^MC state, while the subsequent ^3^MC to ^3^P step proceeds barrierless, a widely used metric for rationalizing the photosubstitution activity of ruthenium polypyridyl compounds is the ^3^MLCT–^3^MC energy barrier (Δ*E*_MLCT‑MC_). The singlet ground state (^1^GS) and triplet excited states (^3^MLCT and ^3^MC) involved in the photosubstitution mechanism were hence minimized at the PBE0 ^55^/TZP^56,57^ /GRIMME3-BJDAMPING^58^/ZORA^59^/COSMO^60–62^ level in water using the AMS 2023 suite^63^ and an unrestricted formalism for the triplet states. The energy of the ^3^MLCT state correlated well with the experimentally observed emission maximum energy (Figure 5A), suggesting that the level of theory was good to model the triplet excited states of such complexes by DFT. Then, the energy barriers for the interconversion of the ^3^MLCT state into the ^3^MC state were calculated by two methods. In the first method, Δ*E*_MLCT‑MC_ was simply defined as the difference between the energy of the ^3^MC state and that of the ^3^MLCT state. In a second, photochemically more precise method, a transition state search was performed on the triplet hypersurface using a nudged elastic band between the ^3^MLCT and ^3^MC states; then the difference between the energy of the transition state and that of the ^3^MLCT state was calculated (Δ*E*_MLCT‑TS_). All energies and barriers are reported in Table 2. We then compared these results to experimental photosubstitution quantum yields under green light irradiation (Figure 5D). Due to the very low molar extinction coefficients at 625 nm, experimental errors in the quantum yield values are comparatively higher than those measured at 520 nm. In addition, photoreactions are often supposed to follow Kasha’s rule,^64^ which states that photoreactivity should depend only weakly on the irradiation wavelength, as it occurs from the lowest excited state (here, from the photochemically generated ^3^MLCT). The plot shown in Figure 5D,E qualitatively demonstrates the shockingly low correlation between the photosubstitution quantum yields and the DFT barriers Δ*E*_MLCT‑MC_ and Δ*E*_MLCT‑TS_, which are characterized by an *R* ^2^ of 0.01 for both energy terms and a Spearman rank correlation coefficient (ρ) of −0.40 and −0.43, respectively. Moreover, the associated p-value of the Spearman correlation coefficient of this plot was larger than 0.05, suggesting that the observed trend can likely be attributed to random noise rather than a statistically significant relationship. A comparison of both barriers to red-light photosubstitution quantum yields is also given in Figure S103, showing an equally bad correlation. Overall, the interconversion energy barrier between the ^3^MLCT state and the ^3^MC state was found to be a bad predictor of the experimentally measured photosubstitution quantum yield in this series of complexes.

**Figure 5:**
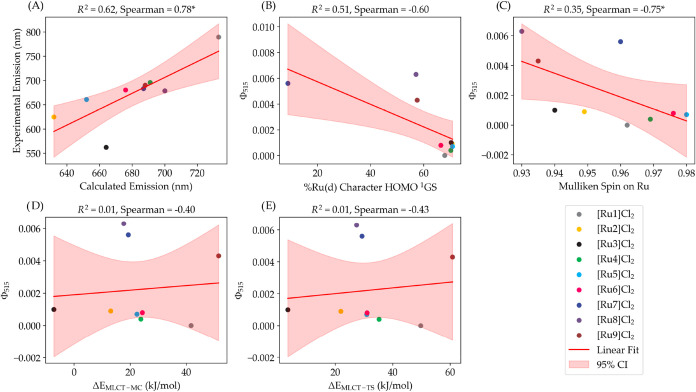
Static DFT modeling predicts emission maxima but cannot predict photosubstitution quantum yields of compounds [Ru1]­Cl_2_-[Ru9]­Cl_2_. (A) Plot of the experimental vs calculated maximum emission wavelengths. (B–D) Plots of the green light photosubstitution quantum yield vs (B) the percentage of ruthenium d orbitals character in the HOMO of the ground singlet state, (C) the Mulliken spin density on the ruthenium atom in the ^3^MLCT excited state, or (D) The energy difference between the MLCT and MC state (Δ*E*_MLCT‑MC_), and (E) the energy difference between the MLCT and TS state (Δ*E*_MLCT‑TS_). The red line in each plot is the linear fit of the data points. A Spearman correlation coefficient without a * indicates *p* ≥ 0.05 (not statistically significant). A single * indicates that *p* < 0.05, suggesting the correlation is statistically significant with less than 5% chance of occurring by random chance. The pink area represents the 95% confidence interval (CI) around the linear regression line as calculated by propagating the standard error of the regression residuals, accounting for the degrees of freedom (number of data points minus two), and scaled by the critical value of the t-distribution at 95% confidence (eq S2).^65^

**Table 2:** Energy Barriers Determined by Static DFT for the Transition of the ^3^MLCT State to the Dissociative ^3^MC State^a^

Complex	Δ*E*_MLCT‑MC_ (kJ/mol)	Δ*E*_MLCT‑TS_ (kJ/mol)
**[Ru1]** ^**2+**^	9.96	11.89
**[Ru2]** ^**2+**^	3.12	5.24
**[Ru3]** ^**2+**^	–1.72	0.83
**[Ru4]** ^**2+**^	5.68	8.43
**[Ru5]** ^**2+**^	5.35	7.39
**[Ru6]** ^**2+**^	5.81	7.42
**[Ru7]** ^**2+**^	4.62	7.00
**[Ru8]** ^**2+**^	4.23	6.54
**[Ru9]** ^**2+**^	12.31	14.54

a The first approach calculates the thermodynamic difference between these two states, and the second approach calculates the difference between the ^3^MLCT and the TS state to the ^3^MC state.

As the Turro group has demonstrated that, for the photosubstitution of nitrile ligands from ruthenium polypyridyl complexes, the degree of ruthenium-ligand orbital mixing in the ground-state HOMO significantly influences the quantum yield,^20,21^ we also extracted from the DFT data of the S_0_ states of our compounds the ruthenium d character of the HOMO. Values of 70.4, 70.8, 68.0, 70.3, 70.9, and 66.4% were found for [Ru1]^2+^, [Ru2]^2+^, [Ru3]^2+^, [Ru4]^2+^, [Ru5]^2+^, and [Ru6]^2+^, respectively. The compounds with the largest photosubstitution quantum yields [Ru8]^2+^ and [Ru9]^2+^ had HOMOs where the ligand contributed more significantly to the HOMO and the contribution of the ruthenium d orbitals was smaller, i.e., 57.1 and 57.5%, respectively. We also found that the HOMO of [Ru7]^2+^ had almost no ruthenium d character, while its quantum yield was lower than that of [Ru8]^2+^ and [Ru9]^2+^ (see Figure 5B). These results suggested an inverse relationship between the percentage of d orbital character in the HOMO of the ^1^GS state and the green light photosubstitution quantum yield of this series of complexes, as indicated by a Spearman rank correlation coefficient (ρ) of −0.60. However, the associated *p*-value was above 0.05, indicating that this trend was not statistically significant and may be due to random noise. A similar inverse relationship, but this time statistically significant (*p* < 0.05), was observed between the quantum yield and the Mulliken spin on the ruthenium atom in the ^3^MLCT state, with a Spearman rank correlation coefficient (ρ) of −0.75 (Figure 5C). Overall, a higher Mulliken spin in the ^3^MLCT state appeared to be associated with a lower photosubstitution quantum yield, though the absolute differences in spin values were extremely small, on the order of only 0.05. Given the well-known basis set sensitivity of atomic population-based charges, such as the Mulliken charge,^66^ we regarded this metric as unreliable.

Since the mechanism of photosubstitution starts from a ^3^MLCT state, where π-back-bonding is significantly reduced compared to the ground state due to the formal electron transfer from the metal to a ligand, we decided to also analyze the relative changes in bonding when going from the ^1^GS to ^3^MLCT state using Mayer bond orders.^67^ Photoinactive [Ru1]^2+^ exhibited only a small decrease in the Ru–N_trans_ bond order at −4.4% (see Table S5), where N_trans_ is the nitrogen atom trans to the sulfur atom. Additionally, the Ru–S bond strength remained largely unchanged. In contrast, several other photoactive compounds showed increased Ru–S bond orders in the ^3^MLCT state. Notably, [Ru2]^2+^ displayed a significant +31.4% increase in the bond order of the Ru–N_trans_ bond, yielding the highest bond order in the ^3^MLCT state, at 0.334. Compounds [Ru7]^2+^, [Ru8]^2+^, and [Ru9]^2+^, which feature an amine bridge and exhibit higher photoactivity, all showed weaker Ru–N_trans_ bonds in the ground state compared to the other compounds except compound [Ru6]^2+^. However, after formation of the ^3^MLCT state, the bond order of Ru–N_trans_ was found to be similar to that of the other compounds, at approximately 2.6. Altogether, these results provided no evidence that the Ru–S bond or the Ru–N bond *trans* to it was weakened in the ^3^MLCT state, nor did they suggest that photosubstitution occurred from this state.

Collectively, these findings emphasized the limited ability of static, solventless DFT calculations to predict the photoreactivity of this series of ruthenium polypyridyl complexes. They also prompted us to look for a modeling method that would offer a more real mechanistic understanding of the photoactivity observed in such complexes, a method that would address the critical need to account for solvent, bond breaking, and bond forming, and dynamical effects.

### Designing the AQUAMEPTH Computational Protocol for Dynamics Simulation of Photosubstitutionally Active Metal Complexes in the Presence of Explicit Solvent Molecules

Understanding the mechanism of photosubstitution reactions in metal complexes is a complex task. Like any ligand exchange reaction happening on the hypersurface of the singlet ground-state, photosubstitution reactions can proceed through three different mechanisms, including dissociative, interchange, or associative, but on the triplet hypersurface of the excited state (see Figure 1B). The reaction also involves various electronic states that collectively influence the photosubstitution quantum yield. A key aspect of such studies is tracking the singlet–triplet energy gap to identify deactivation sinks. Nonadiabatic molecular dynamics that incorporate trajectory surface hopping to account for triplet-singlet transitions can circumvent this problem, as it explores multiple electronic states at the same time and is thus well-suited for investigating photoactivated processes.^68^ However, these simulations are computationally intensive even for small systems, and are typically limited to the ∼100 fs time scale, which is far shorter than the time scales required for photosubstitution reactions to unfold (ns to μs). Even when focusing on a single electronic state, such as the reactive triplet state, computational demands remain a significant limitation due to the need to use (spin-) unrestricted methods. Such simulations, for example, using the SHARC software, are typically restricted to just a few picoseconds, which is also insufficient to fully explore the complete free energy surface.^35,68^ To mitigate these time constraints, we employed the semiempirical GFN1-xTB method, which yielded satisfactory agreement with DFT, *vide infra*.

Despite the use of a semiempirical approach, accounting for explicit solvent effects still imposes substantial computational demands, necessitating additional approximations such as using quantum mechanics/molecular mechanics (QM/MM). Standard QM/MM simulations typically require defining all of the QM atoms prior to the simulation. However, in a photosubstitution reaction in water, it is *a priori* not known which H_2_O molecule will interact with the ruthenium atom to form the triplet state of the photoproduct (^3^P). Consequently, a ^3^P state may be generated where the interacting solvent molecule is treated at the MM level, while the ruthenium atom is treated at the QM level, leading to boundary artifacts and errors. To address this issue, for AQUAMEPTH we opted for an adaptive buffer QM/MM approach (AdBF-QM/MM).^69^ In this approach, the solute is always treated at the QM level, while the solvent molecules are dynamically treated as QM when they come within a certain distance of the solute. To mitigate force discontinuities caused by abrupt switching between treating the atoms at the QM or MM level, a transition (buffer) region is introduced. Within this region, the forces are computed as a linear combination of QM and MM contributions, providing a smooth interpolation between the two regimes. Implementations of this approach typically require two distinct QM/MM calculations to properly account for the mixed interactions, one where the QM region is small and one with an enlarged QM region.^69^

To allow AQUAMEPTH to explore the transition from the ^3^MLCT state to the ^3^MC state, we also used enhanced sampling techniques. Many chemical simulations use small timesteps to accurately integrate all modes of motion and maintain chemical accuracy and stable dynamics. However, many chemical processes occur on time scales that are orders of magnitude longer than the time step, which makes them difficult or impossible to observe within feasible simulation durations. To address these limitations, enhanced sampling methods have been developed: they bias simulations toward more relevant regions of the phase space, thereby accelerating the computational extraction of the desired properties. Fortunately, the photosubstitution reaction in our series of complexes is primarily characterized by a single reaction coordinate: the Ru–S distance. To steer the reaction and produce ^3^MC states, we can hence use an enhanced sampling approach named “umbrella sampling”.^70^ In this approach, we chose a reaction coordinate and ran multiple simulations in parallel, each with a different restraint along that reaction coordinate (Figure 6A). In our case, we varied the Ru–S bond distance from 2.0 Å (reagent) to 6.0 Å (photoproduct) in small steps of 0.15 Å. As long as these steps are small enough to ensure that adjacent simulations sample overlapping regions, we can reconstruct an accurate energy profile of the (photo)­reaction. However, given the three possible photosubstitution mechanisms highlighted above, the bond formation with a water molecule may occur at different stages towards the product ^3^P. To explore the free energy surface without biasing the system toward a specific mechanism, we utilized a second enhanced sampling approach called well-tempered metadynamics (Figure 6B).^71,72^ This method applies a history-dependent Gaussian bias along the reaction coordinate, gradually filling the potential energy landscape. As the simulation progresses, regions that have already been sampled become energetically unfavorable, promoting exploration of conformations that are typically inaccessible due to high energy barriers. With sufficient sampling, the bias compensates for the underlying free energy, eventually flattening the energy landscape and yielding a converged free energy surface. For our calculation, we decided to choose the distance Ru–O_min_ between the ruthenium and the nearest oxygen of a water molecule as a reaction coordinate to be biased by well-tempered metadynamics. A schematic representation of these approaches is shown in Figure 6. Overall, the AQUAMEPTH protocol consisted of the combination of adaptive QM/MM with umbrella sampling and metadynamics; we used it for modeling photosubstitution in the triplet states of [Ru1]^2+^-[Ru9]^2+^ using explicit water solvent molecules. More details and all computational settings of AQUAMEPTH can be found in the computational section of the Experimental Part.

**Figure 6:**
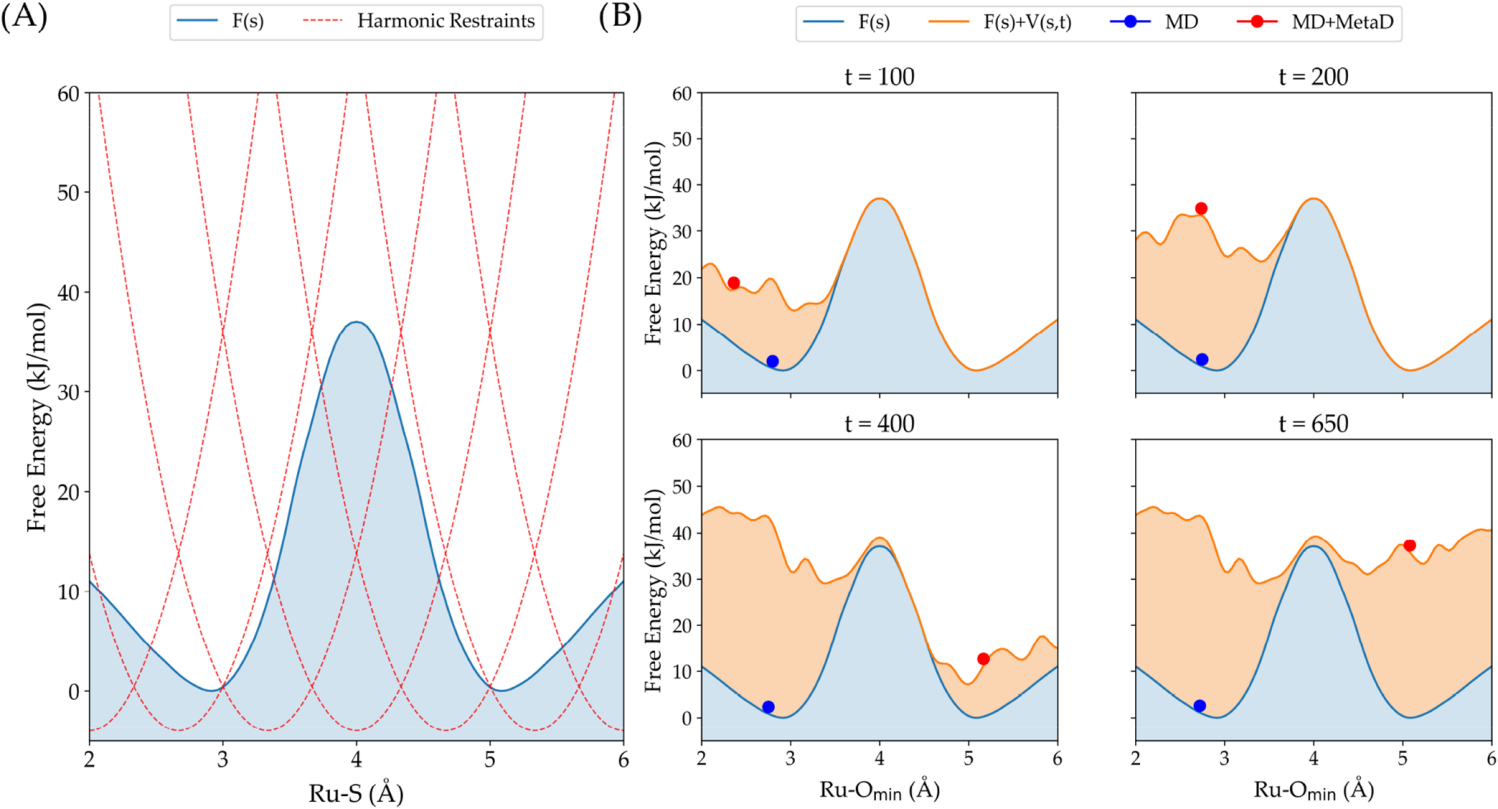
Schematic illustration of the two enhanced sampling methods applied in the AQUAMEPTH protocol for the simulation of photosubstitution reactions. (A) Umbrella sampling: we begin by defining in advance the range over which we want to extend the reaction coordinate, here the ruthenium–sulfur bond distance. This range is divided into multiple overlapping windows, each corresponding to a separate molecular dynamics simulation. In each window, a harmonic restraint is applied centered at a specific Ru–S bond length. This setup confines the system to a particular segment of the reaction coordinate, enabling efficient sampling of the free energy landscape. By combining the results from all windows, we can reconstruct the full free energy surface along the reaction coordinate (in blue). (B) Well-tempered metadynamics: biasing potentials *V*(s,t), where s represents the reaction coordinate (in our case, the Ru–O_min_ distance between ruthenium and the closest oxygen atom) are gradually added along the reaction coordinate as the system evolves. These biases encourage the system to escape local minima and explore higher energy configurations, potentially overcoming significant energy barriers to sample alternative states. As the simulation continues, the height of the added bias decreases gradually, ensuring a controlled exploration of the energy landscape and allowing the system to smoothly converge toward the true free energy surface.

### Protocol Validation

Utilizing the AdBF-QM/MM method presented several challenges, the first being its greater computational cost compared to conventional QM/MM, as it requires two QM/MM calculations per molecular dynamics step. To address this issue and extend our simulation time range, we adopted for AQUAMEPTH the semiempirical GFN1-xTB method to model the QM system (the solute), while the MM region (the solvent molecules) was described using a classical force field. This approach reduced the computational overhead by a factor of ∼33. Although we also tested treating the whole system, solute, and solvent molecules at the GFN1-xTB level, it remained computationally too demanding for our approach. A second major challenge lies in the optimization of two critical hyperparameters. As previously described, the AdBF scheme employs a dynamical QM region defined by a cutoff distance r_QM_, within which atoms are treated fully at the QM level. Surrounding this QM core region is the buffer region, defined by a second cutoff, r_buffer_, which extends from the boundary of the dynamical QM region. Within the buffer region, atomic forces are interpolated between the QM and MM contributions to ensure a smooth transition. To tune these parameters, we generated a representative configuration of compound [Ru8]^2+^, approximating a transition state where a water molecule interacts with both ruthenium and sulfur atoms (Figure S95). We then evaluated forces, Mulliken charges, and spin populations across a range of *r*_QM_ and *r*_buffer_ values. We found that a combination of *r*_QM_ = 3.0 Å and *r*_buffer_ = 1.0 Å yielded the best compromise between computational speed and accuracy in force, charge, and spin predictions (Figures S81–S86). Interestingly, despite GFN1-xTB being parametrized in a spin-restricted manner, the computed Mulliken spin closely matched those obtained with DFT (PBE^73^/DZVP-MOLOPT-GTH^74,75^ /D3 ^76^), with an average deviation for the compound of only ∼0.04 (Figures S83 and S86).

### *Cis* and *Trans* Photosubstitution Pathways

Following the method validation of AQUAMEPTH, we analyzed the total trajectory for each compound. This analysis first revealed that, contrary to our initial expectation that the aqua molecule would always attack the ruthenium from the same side as the leaving sulfur ligand (*cis* attacks, S–Ru–O_min_ < 90°, Figure 7), two distinct pathways were observed, i.e., the aqua ligand sometimes also attacked *trans* to the sulfur atom (S–Ru–O_min_ > 90°, Figure 7). Figure 8 displays the distribution of sampled points on a grid, with the Ru–O_min_ distance plotted on the *y*-axis and the S–Ru–O_min_ angle on the *x*-axis. The anticipated ^3^P state forms at a Ru–O_min_ distance of approximately 2.0–2.5 Å. The *cis* reaction pathway to form the ^3^P state is allowed by the elongation of the Ru–S bond in the ^3^MC state, which creates space for an incoming water molecule on the same side of the complex. In the second *trans* pathway, the aqua ligand makes use of the elongated Ru–N_trans_ bond in the ^3^MC state and of a possible rotation of the bidentate ligand around the Ru hinge, which also generates space to attack with an S–Ru–O_min_ angle of incidence exceeding 90°.

**Figure 7:**
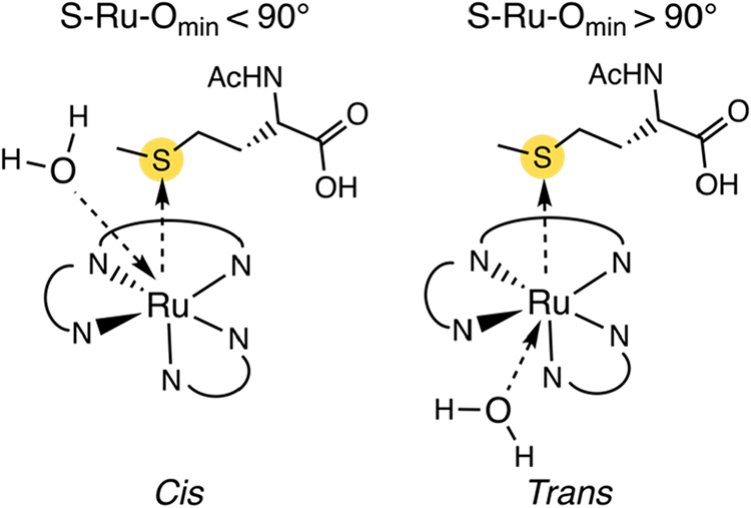
Schematic figure of the *cis* and the *trans* mechanism for photosubstitution of the Ru–S photocleavable bond.

**Figure 8:**
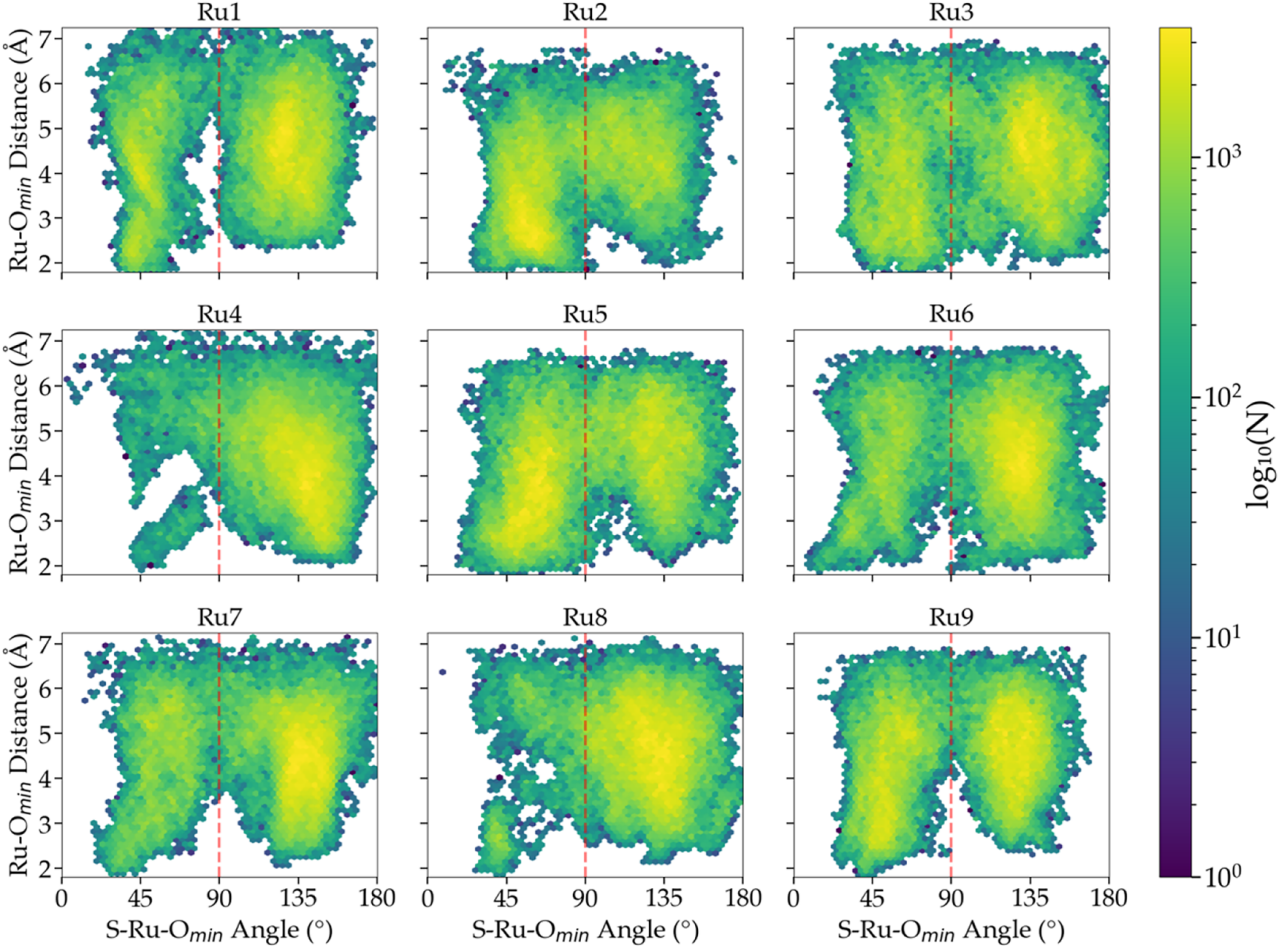
Distribution of attack angles of the aqua ligand during photosubstitution of thioether obtained for [Ru1]^2+^-[Ru9]^2+^. Two predominant reaction pathways can be defined: in the *cis* case, the S–Ru–*O*_min_ angle is smaller than 90°; and in the *trans* case, this angle is larger than 90°.

The preference for one or the other pathway was found to be compound dependent. We categorized each compound based on the *cis*/*trans* attack configurations percentage, where for each configuration the Ru–O_min_ distance was smaller than 4 Å (Table 3). According to this analysis, we can group the complexes into three different categories: the complexes where the *cis* pathway was dominant (e.g., [Ru2]^2+^, [Ru5]^2+^, and [Ru9]^2+^), those that have no preference (only [Ru3]^2+^), and those that preferentially went *trans* ([Ru1]^2+^, [Ru4]^2+^, [Ru6]^2+^, [Ru7]^2+^, and [Ru8]^2+^). Though these results provided interesting new insight into the mechanism of ligand photosubstitution in this series of complexes, they did not allow us to rationalize the trend observed in the experimental photosubstitution quantum yields.

**Table 3:** Percentage of Configurations That Had a *cis* or a *trans* S–Ru–*O*_min_ Angle of Incidence Where the Ru–*O*_min_ Distance Was Smaller than 4 Å

Complex	*Cis* (%)	*Trans* (%)
**[Ru1]** ^**2+**^	38.7	61.3
**[Ru2]** ^**2+**^	75.1	24.9
**[Ru3]** ^**2+**^	48.5	51.5
**[Ru4]** ^**2+**^	4.2	95.8
**[Ru5]** ^**2+**^	74.0	26.0
**[Ru6]** ^**2+**^	33.2	66.8
**[Ru7]** ^**2+**^	33.6	66.4
**[Ru8]** ^**2+**^	14.2	85.8
**[Ru9]** ^**2+**^	62.7	37.3

### Dynamics of a Bidentate Ligand

We further investigated the influence of two other properties that may influence photosubstitution: the planarity and rigidity of the bidentate ligand. We define these two properties based on two planes: one including the Ru atom, the coordinating N_C_ atoms of the bidentate chelate that is in *cis* position to the monodentate thioether ligand, and the carbon atom C_C_ bound to N_C_ and located toward the other pyridyl ligand of the chelate; and a second plane defined similarly but including the N_T_ atom *trans* to the monodentate thioether ligand instead, and the C_T_ atom bound to it (Figure 9A). These two planes define a dihedral angle θ (Figure 9B), which can be used to capture the ligand’s planarity and rigidity during a dynamical simulation (Figure 9C). In this approach, we define the planarity μ­(θ) of the ligand as the average value of θ during a dynamics simulation of the complex: a value of 0° corresponds to a flat geometry and hence a “planar” ligand. The rigidity of the bidentate chelate is related to the fluctuations of θ around this average value; we quantified it here as the standard deviation σ­(θ) of θ throughout the same dynamics simulation. As a note, since the ligand can bend in either direction, the dihedral angle θ can be positive or negative depending on the orientation of the bend. In the context of processes like accessing the *trans* pathway, it is the degree of bending that matters, not whether the ligand bends “left” or “right.” We map the angle θ onto a scale from 0 to 90°, where 0° corresponds to a completely flat (planar) ligand and 90° corresponds to one that is maximally bent (perpendicular). This transformation ensured that bending in either direction was treated equally. A low mean value of μ­(θ) indicated a predominantly planar (flat) structure, whereas a high value of the standard deviation σ­(θ) suggested conformational flexibility and, hence, a low rigidity of the chelate. This analysis (see also Figure S102) showed that the photoinactive compound [Ru1]^2+^ exhibited the highest planarity, with a mean angle μ­(θ) of 12.1°. The ligands of the less photoactive compounds [Ru2]^2+^, [Ru3]^2+^, [Ru4]^2+^, [Ru5]^2+^, and [Ru6]^2+^, displayed planarity values fluctuating around 15°. In contrast, the more photoactive compounds, [Ru7]^2+^, [Ru8]^2+^, and [Ru9]^2+^, demonstrated significant deviations from planarity with mean angles of 69.0, 47.1, and 58.7°, respectively. A linear regression analysis was performed to correlate the green light photosubstitution quantum yield (**Φ**_**515**_) with the planarity of the ligand, μ­(θ) (Figure 10). This analysis yielded a strong correlation with an *R* ^2^ value of 0.85 and a Spearman rank correlation coefficient (ρ) of 0.87, which clearly outperformed the traditionally used ^3^MLCT–^3^MC DFT barrier metric (see above). The rigidity of the bidentate ligand σ­(θ) exhibited no strong correlation with the observed photosubstitution quantum yield (Figures S105–S106). According to this approach, the planarity μ­(θ) of the bidentate chelate is the best molecular descriptor for the prediction of the photosubstitution quantum yield value **Φ**_**515**_ in compounds of the family [Ru­(tpy)­(N–N)­(AcMet)]^2+^.

**Figure 9:**
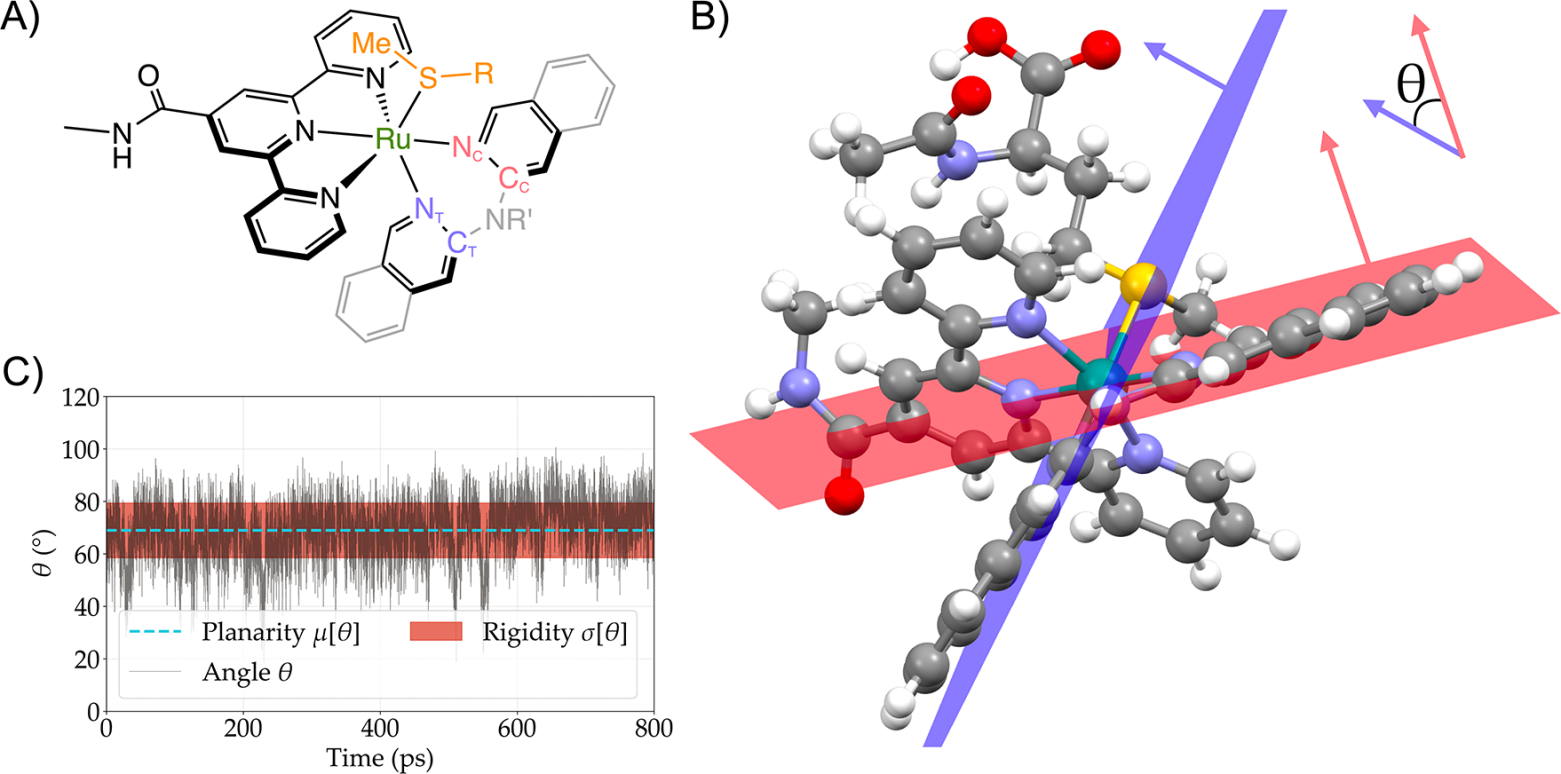
Schematic representation of how the planarity and rigidity of the bidentate chelate in [Ru1]^2+^-[Ru9]^2+^ are defined and computed. (A) N_C_/C_C_ and N_T_/C_T_ are defined by their *cis* or *trans* position with respect to the S atom of the thioether ligand (all non-S atoms of the thioether ligand have been omitted for clarity). (B) θ is defined as the dihedral angle between the two planes Ru–N_C_-C_C_ (in red) and Ru–N_T_-C_T_ (in blue). (C) The angle θ is monitored throughout any simulation, defining planarity of the bidentate ligand μ­(θ) as the mean of θ (blue dashed line) and its rigidity σ­(θ) as the standard deviation of θ (red area).

**Figure 10:**
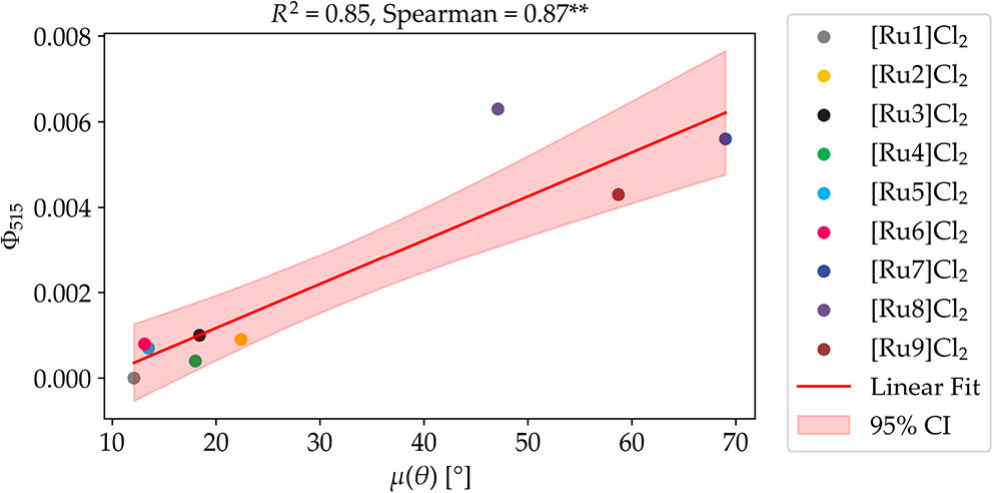
Correlation between the experimental green light photosubstitution quantum yield (**Φ**_**515**_) and the planarity of bidentate ligand μ­(θ) (θ is the angle between the bond vectors highlighted as straight arrows in Figure 9). We are only concerned about the deviation from planarity, so 0° indicates that both parts of the bidentate ligand are planar to each other. A double ** indicates that *p* < 0.01, suggesting the correlation is strongly statistically significant with less than 1% chance of occurring by random chance. The pink area represents the 95% confidence interval (CI) around the linear regression line, calculated by propagating the standard error of the regression residuals, accounting for the degrees of freedom (number of data points minus two), and scaled by the critical value of the t-distribution at 95% confidence (eq S2).^65^

### Photoinactivity

To measure if the AQUAMEPTH protocol would help us understand the lack of photoreactivity of [Ru1]^2+^, two free energy surfaces were generated: one where we considered only configurations that had a *cis* attack resulting in a *cis* free energy surface (S–Ru–*O*_min_ angle < 90°), and one for the *trans* attack obtaining the *trans* free energy surface *(*S–Ru–*O*_min_ angle > 90°) (see Figure 11). We calculated the standard deviation of the Ru–S and Ru–*O*_min_ distances for the unbiased singlet state dynamics of each system to identify the 95% probable area on the generated free energy surface where photon absorption is likely to occur. As photon absorption and intersystem crossing are ultrafast processes in ruthenium complexes that occur on the fs time scale, we assumed that no large structural rearrangement would occur during these two events. Our analysis provided the starting point on the triplet hypersurface, pinpointing the most probable region for the formation of the ^3^MLCT state (black dotted circle in Figure 11). As exemplified on the corresponding ruthenium Mulliken spin density surface generated for the triplet hypersurface, each starting position showed a low Mulliken spin on ruthenium of approximately 0.7, supporting the ^3^MLCT character of the starting point, as 1 of the unpaired electrons in the triplet state is located on a polypyridyl ligand. For the *cis* pathway, no stable intermediates were identified where the Mulliken spin on the ruthenium atom increased beyond 1.2, a threshold indicative of ^3^MC state formation as the 2 unpaired electrons are located on the metal center. Instead, the observed local minima correspond to Ru–S distances characteristic of the ^3^MLCT states. This suggested that reactions via the *cis* pathway are unlikely for this molecule, with other photochemical processes such as nonradiative decay and phosphorescence likely dominating instead. A similar result was found for the *trans* pathway, where no stable minima were found either where the Ru–S and Mulliken spin were indicative of a ^3^MC state. Irradiation of the compound would thus likely lead to activation to the ^3^MLCT state, after which deactivation to the ground state occurs via either nonradiative decay or phosphorescence. Overall, our protocol predicted the absence of reactive ^3^MC states, indicative of an absence of photosubstitution reactivity for [Ru1]^2+^, in agreement with the experimental observation.

**Figure 11:**
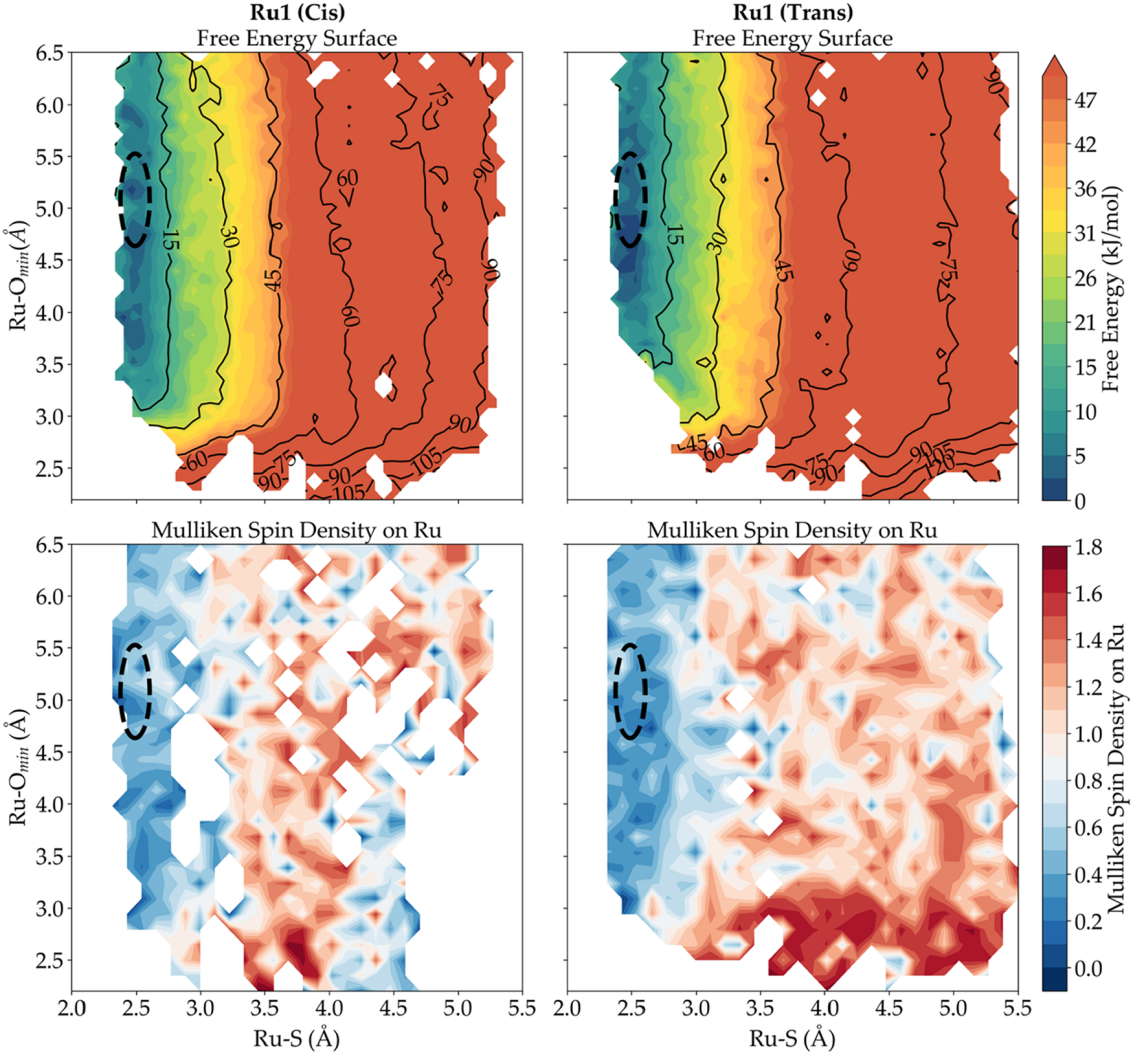
Top panel: free energy surfaces for the *cis* and *trans* pathways of photosubstitutionally inert compound [Ru1]^2+^ according to the AQUAMEPTH protocol. Bottom panel: Mulliken spin density surface on ruthenium at the PBE^73^/DZVP-MOLOPT-GTH^75^/D3.^76^ The black dotted circle represents the 95% probable region on the generated free energy surface where photon absorption is most likely to occur and corresponds to the ^3^MLCT state starting the photosubstitution reaction on the triplet hypersurface.

### Mechanistic Insights into Photoactive Compounds

Interestingly, application of the AQUAMEPTH protocol to the photoactive compounds [Ru2]^2+^-[Ru9]^2+^ uncovered distinct free energy profiles, enabling us to identify various mechanistic pathways taken by apparently similar photosubstitution reactions (Figure 1B). As discussed, the key distinction between different ligand exchange mechanisms lies in whether a pentacoordinate dissociative intermediate, no intermediate, or a heptacoordinate associative intermediate is formed during photosubstitution. To characterize these pathways, we define a cutoff distance beyond which we consider there is no interaction between the ruthenium center and the relevant oxygen or sulfur atoms. In the case of ^3^MC states, the departing ligand still interacts with the metal atom. Therefore, we set the cutoff distance to 4.0 Å, which corresponds to the maximum separation observed in previously identified extreme static DFT structures.^47^

We observed for [Ru2]^2+^ and [Ru3]^2+^ a minimum energy path that was consistent with a dissociative mechanism, where the thioether ligand dissociated first to result in a pentacoordinate species representing a local minimum on the triplet hypersurface before an aqua ligand bound to Ru to afford the photoproduct. For [Ru2]^2+^ (Figures 12A and S92), the major *cis* reaction pathway (75.1% of all trajectories, see Table 3), the system was taken from the photochemically generated ^3^MLCT region to a nearby local minimum characterized by a slightly elongated Ru–S distance of approximately 3.0 Å and a Ru–O_min_ distance of 4.0 Å characteristic of unbound aqua ligands. The Mulliken spin density on Ru in this intermediate (∼1.2–1.4, see bottom of Figure 12A) confirmed that this minimum corresponded to a ^3^MC state that probably fitted well with the ^3^MC states typically modeled by static, solvent-free DFT methods.^45^ The reaction further progressed via a transition state to another local minimum on the triplet hypersurface, where the Ru–S distance extended to ∼4.5 Å while the Ru–O_min_ distance remained nearly constant (∼4.0 Å). In this geometry, both Ru–S and Ru–O_min_ distances were too large to correspond to a coordination bond; hence, this second intermediate should be considered as pentacoordinate. Subsequently, the reaction proceeded through a transition state to another local minimum where the Ru–O_min_ decreased to 2.5–3.0 Å, indicative of the formation of a true Ru–O coordination bond and hence a true product triplet state (^3^P), from which ISC to the singlet afforded the final singlet photoproduct. For [Ru3]^2+^, which went equally via the *cis* and *trans* pathways, the initial ^3^MLCT structure (Figure S109) quickly evolved into the nearby ^3^MC state local minimum, characterized by a Ru–S distance of ∼3.0 Å and a Ru–O_min_ distance of 5.0 Å (noncoordinated water). The system then evolved into a pentacoordinate intermediate, where both Ru–S and Ru–O_min_ were approximately ∼4.5 Å. The ^3^P was then readily formed after crossing a small energy barrier.

**Figure 12:**
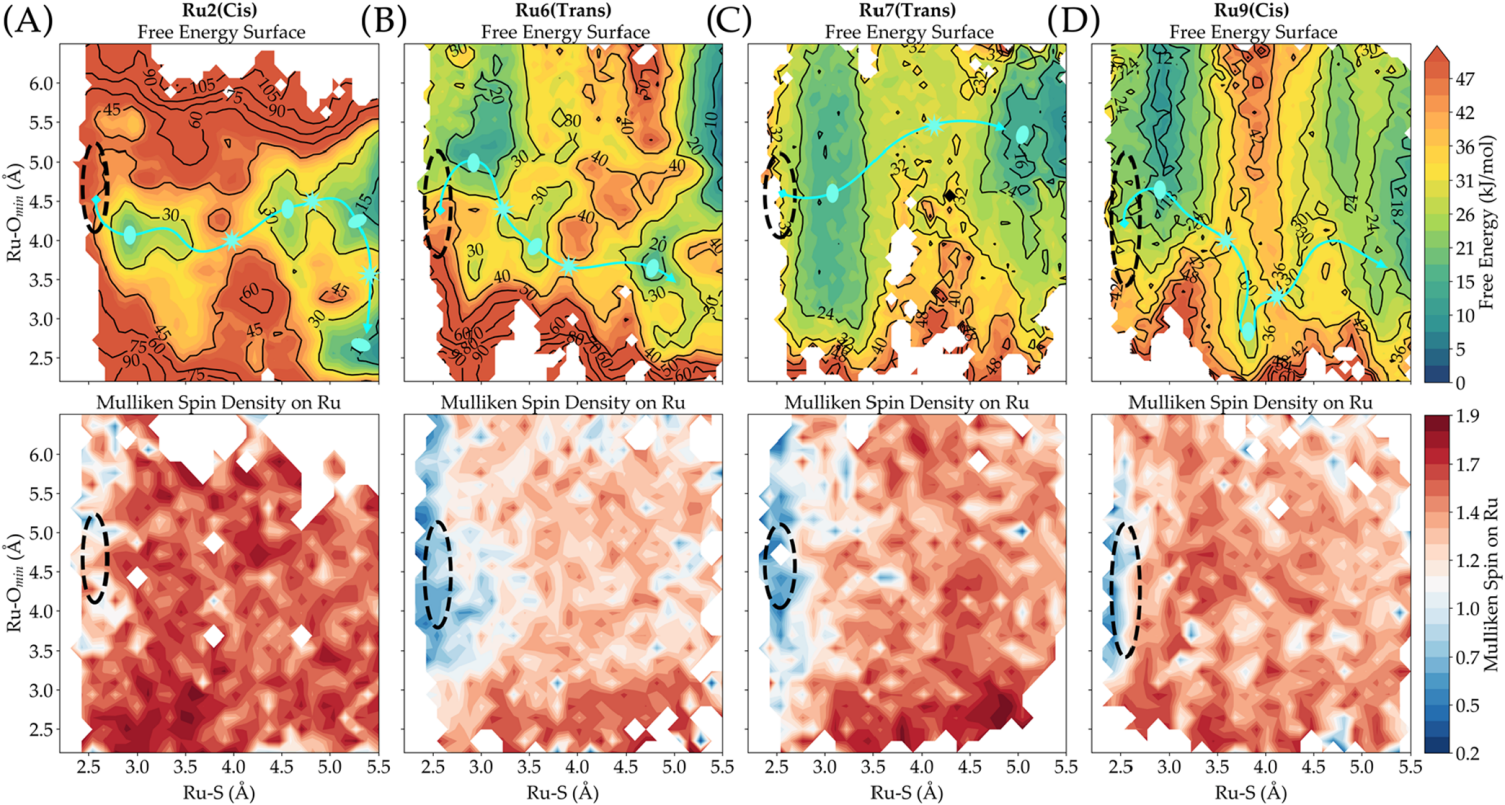
Free energy surfaces for the different types of photosubstitution mechanisms obtained by applying the AQUAMEPTH protocol: (A) dissociative, (B) interchange-associative, (C) interchange-dissociative, and (D) associative. The black dotted circle represents the 95% probability region on the generated free energy surface where photon absorption is most likely to bring the system; it also corresponds to the ^3^MLCT state, which starts the photosubstitution reaction on the triplet hypersurface. For visual guidance, the most probable trajectory along the minimum energy path is shown in cyan. Energy minima are marked with ovals, and approximate transition states with stars. The path and markers were manually added to aid interpretation.

A slightly different reaction mechanism was revealed by our modeling protocol for [Ru5]^2+^, [Ru7]^2+^, and [Ru8]^2+^, which followed a mechanism closer to the dissociative interchange model. [Ru5]^2+^ followed in the majority of the cases the *cis* pathway (Figure S111), and in this case, a single transition state needed to be crossed for the reaction to reach the product (^3^P) state. The transition state was located at a Ru–S distance of ∼3.5 Å and an Ru–*O*_min_ distance of ∼4.5 Å. Reminiscent of an interchange-dissociative mechanism, the Ru–S first increases while the Ru–*O*_min_ is constant, and no stable intermediate dissociative state was observed after crossing the transition state, allowing the direct formation of the ^3^P state. Compound [Ru8]^2+^ (Figure S114) went mostly *trans* and exhibited features similar to those of [Ru5]^2+^; the reaction transitioned from the initial ^3^MLCT state to a broad ^3^MC state region as well. The reaction then progressed with a slight elongation of the Ru–S bond, eventually reaching a ^3^P-like state at an approximate Ru–S distance of 4.5 Å without the involvement of any intermediate states. Notably, the local minimum ^3^P was not present on the *cis* free energy surface, which could be the reason the *trans* reaction pathway is preferred for this compound (86% majority). Like those of [Ru5]^2+^ and [Ru8]^2+^, the ^3^MC minimum of [Ru7]^2+^ (Figures 12C and S97) was broad and close to the ^3^MLCT region. This complex followed primarily the *trans* (66%) pathway, but a significant difference was found between the *trans* and *cis* pathways. In the *cis* pathway, an aqua-coordinated ^3^P local minimum was clearly present, while it was absent from the *trans* pathway, where ISC to the singlet state seems to be necessary so that the aqua ligand binds to Ru again. Only a local minimum corresponding to a pentacoordinated state, where both Ru–S and Ru–O_min_ distances were ∼5.5 Å, was found after crossing the reaction barrier. This observation was consistent with calculations from Dixon et al. on the [Ru­(tpy)­(dcbpy)­(Hmte)]^2+^ analogue (dcbpy = 6,6′-dichloro-2,2′-bipyridine, Hmte = 2-thiomethylethanol), where initial dissociation of the thioether took place on the triplet hypersurface, followed by rotation of the bipyridine into a pentacoordinated, symmetrical triplet geometry that could not bind to any aqua ligand. It was necessary for this molecule to undergo intersystem crossing back to the singlet ground state, which was concomitant to a rotation of the dcbpy chelate, before an aqua ligand could bind to Ru to give the final singlet aqua complex (^1^P).^35^

A mechanism reminiscent of “concerted” mechanisms in organic chemistry was found for [Ru4]^2+^. [Ru4]^2+^ almost exclusively followed the *trans* pathway (96%), and the energy landscape was broad and nearly barrierless, with the Ru–S distance extending beyond 4.0 Å. A very flat potential energy surface indicates that the energy changes only minimally along the reaction coordinate, consistent with a concerted process in which bond formation and bond cleavage are synchronized. This is characteristic of interchange mechanisms, where the transition state is diffuse and lacks the distinct intermediates or transition states seen in more interchange-associative or interchange-dissociative pathways.^77^ However, no distinct minima characteristic of a ^3^P state, defined by a large Ru–S distance and a small Ru–*O*_min_ distance, could be found (Figure S110). This result suggested that, like [Ru7]^2+^ for which a *trans* reaction occurred, the formation of the aqua photoproduct for [Ru4]^2+^ likely occurred in the singlet ground state, and only dissociation takes place on the triplet hypersurface.

Compound [Ru6]^2+^ (Figures 12B and S96) primarily followed the *trans* pathway (67%), initially stabilizing in a ^3^MC state, where the Ru–O_min_ distance was slightly longer than that in the ^3^MLCT region. The reaction then proceeded through an intermediate state with a Ru–O_min_ distance of approximately 4.0 Å, while the Ru–S bond remained relatively short at 3.25 Å. Along the minimum energy path, the Ru–S and Ru–O_min_ distances evolved concurrently (Ru–S increasing and Ru–O_min_ decreasing), leading to the final product state. Although a transient heptacoordinate-like geometry is observed, the mechanism is more accurately classified as interchange-associative instead of associative since the Ru–O_min_ distance remains near the defined cutoff of 4.0 Å.

In contrast to all previously observed mechanisms, [Ru9]^2+^ (Figures 12D and S115) exhibited a distinctly associative reaction mechanism. The compound follows primarily the *cis* pathway (63%), and the calculated ^3^MC region formed a broad minimum where the Ru–O_min_ distance first slightly increased compared to the ^3^MLCT state before it experienced a significant decrease, to reach a local minimum at Ru–O_min_ 3.75 and Ru–S 3.0 Å. This local minimum can be considered as a heptacoordinate species since the interaction cutoff for a ^3^MC-like interaction was set at 4.0 Å. From this intermediate state, the reaction progressed toward complete Ru–S dissociation, before undergoing ISC to the singlet photoproduct hypersurface.

## Discussion

Computational modeling of photosubstitution reactions is a recognized endeavor in photochemistry. Here, the weak phosphorescence in this series of 9 photolabile complexes spanned through a wide range of wavelengths, which allowed us to probe the influence of the ^3^MLCT excited state energies on photoreaction rates. On the one hand, we could rule out that stabilizing ^3^MLCT enhanced photosubstitution quantum yields, as had been suggested for other types of complexes.^22^ On the other hand, we had initially anticipated that incorporating electron-donating bidentate chelates would destabilize the ^3^MLCT state, thereby lowering the ^3^MLCT-^3^MC energy gap and hence increasing the photosubstitution quantum yields. Our experimental measurements immediately indicated that this trend did not materialize either. Static DFT calculations revealed a very good correlation between computed ^3^MLCT and measured ones, but no correlation was found between the ^3^MLCT-^3^MC barrier and the photosubstitution quantum yield. Hence, the efficacy of these photoreactions must be linked to another molecular parameter, up to now unknown.

To address the limitations of static DFT calculations and the typically high computational cost associated with dynamics simulations of photosubstitution using QM/MM, we developed the AQUAMEPTH protocol to explore the dynamical features of each complex in the series with explicit inclusion of the solvent in a computationally affordable manner. Several key insights could be determined from the dynamics that had been, up to now, impossible to address with static calculations. First, we identified that in contrast to prior beliefs, the reaction could occur not only via a *cis* attack of the aqua molecule from the solvent, but also in a *trans* fashion. It is often assumed that the photosubstitution of monodentate ligands follows a mechanism in which the angle of incidence between the dissociating coordinating atom, the metal, and the incoming solvent molecule is ∼90°. In our ruthenium complexes, a *trans* reaction pathway was found as the major reaction pathway for several complexes in the series. Such a *trans* attack must occur via a ^3^MC state reminiscent of the ^3^MC_trans_ states reported by Elliot et al. for bidentate ligand photosubstitution.^27^ In these states, occupation of the d_z^2^_ or d_x^2^–y^2^_ orbital leads to elongation of a coordination bond and structural reorganization. This *trans* reaction pathway strongly supports our second key finding, which is that the primary factor influencing the photosubstitution quantum yield is not the electronic effects on the energy levels of the ^3^MLCT and ^3^MC states but, instead, the structural characteristics of the bidentate ligand. We found that from our defined parameter θ, two properties of the bidentate ligand naturally emerged, namely its planarity μ­(θ) and rigidity σ­(θ). Specifically, we observed an unexpected but strong correlation between the planarity of the bidentate ligand and the photosubstitution quantum yield, with an *R* ^2^ value of 0.85 and a Spearman rank correlation coefficient of 0.87. If the reaction occurred exclusively via the *cis* pathway, ligand planarity would play a less significant role, as this feature of the bidentate chelate is not directly influencing the incoming solvent molecule. However, since the reaction can also proceed through a *trans*-like mechanism, the spatial requirements and the reorganization energy needed to accommodate the incoming solvent molecule become crucial factors. More rigid ligands require greater energy for reorganization, whereas the more photoactive compounds in our series, particularly those with an amine bridge between the two coordinated pyridine rings, exhibited a lower planarity and greater flexibility. Such flexibility makes them better at generating the necessary space in the coordination sphere for substitution, which leads to higher photosubstitution quantum yields.

Our third key finding is that AQUAMEPTH can successfully predict the photoactivity and photoinactivity of a complex, even though the level of theory was only based on the semiempirical GFN1-xTB level. The photoinactive compound [Ru1]^2+^ exhibited free energy surfaces where no local minima were observed that would correspond to ^3^MC states or any other dissociative states beyond the ^3^MLCT region. In contrast to photoactive compounds, a distinct minimum was observed directly around the ^3^MLCT state region, suggesting that this state represents the final accessible point on the triplet hypersurface. Even though we did not generate the singlet free energy surface to directly compare the singlet triplet free energy difference, it is likely that most nonreactive processes occur from the ^3^MLCT. This assumption is supported by our experiments, as [Ru1]^2+^ has the highest absorption maximum wavelength, indicating that the ^1^GS–^1^MLCT energy difference is the smallest of the series, which also predicts a low ^3^MLCT state.

A final key insight that could be generated with AQUAMEPTH is that we observed for the first time mechanistic insights into the mechanism, which can be associative, interchange, or dissociative. Next to the wide range of photosubstitution quantum yields and ^3^MLCT levels observed in this series of compounds [Ru1]^2+^-[Ru9]^2+^, we found a great diversity of such mechanisms. A purely dissociative pathway was observed for [Ru2]^2+^ and [Ru3]^2+^. [Ru5]^2+^, [Ru7]^2+^, and [Ru8]^2+^ exhibited an interchange-dissociative mechanism, while [Ru4]^2+^ exhibited an interchange mechanism. In contrast, [Ru6]^2+^ proceeded via an interchange-associative mechanism, while [Ru9]^2+^ followed a distinctly associative mechanism. Although no direct correlation was found between a preference for the *cis* or *trans* reaction and the type of mechanism, we observed that, for each compound, the *cis* and *trans* free energy surfaces displayed similar features, usually with only minor differences in energy levels and position of the minima. GFN1-xTB reliably predicts vibrational frequencies and geometries near local energy minima that are comparable to DFT.^78^ Reweighting approaches have demonstrated that transforming the GFN1-xTB free energy surface into a DFT free energy surface results in significant changes in energy differences, but does not substantially alter the location of the minima located on the free energy surface with respect to the reaction coordinates.^79,80^ One of the disadvantages of using the AdBF-QM/MM approach is that we have a nonconstant potential energy surface. This makes reweighting the GFN1-xTB to DFT level energies currently not possible, allowing us to analyze the mechanisms on the free energy surface only semiquantitatively, i.e., we cannot compare thermodynamic and kinetic properties obtained from our surfaces. Moreover, even though we obtain very similar Mulliken spin values on our atoms, intrinsically, GFN1-xTB is not parametrized for spin-unrestricted structures. Future endeavors should focus on developing efficient methods that are able to transform the AdBF-QM/MM free energy surface from GFN1-xTB to DFT, as well as improving GFN-xTB methods by including spin effects in the parametrization of the Hamiltonian.

The influence of planarity on photosubstitution quantum yields has been explored in previous studies of the photosubstitution of bidentate ligands. In some of these works, the planarity of a ligand was defined as an N–Ru–N angle, and was interpreted as a “metal–ligand bond misalignment” with respect to ideal octahedral geometry.^81–83^ This definition is entirely based on the geometry of the first coordination sphere, and it is measured in a static way, either from DFT studies or from a crystal structure. A recent study from Glazer et al. examined the photosubstitution of dipyridylamine analogues where the amine bridge of the chelate was replaced by a CH_2_, C_2_H_4_, S, or O bridge. These authors considered the same Ru–N–C atoms as in our analysis, but they focused on the Ru–N–C angle as a measure of the direction of the nitrogen lone pair to the metal, and found no correlation between this angle and the photosubstitution quantum yields.^84^ Our approach differs from previous work in three fundamental ways: (i) it incorporates dynamic features rather than relying on static geometries; (ii) the bidentate chelate is a spectator ligand rather than the photosubstituted ligand, which offers an easier definition of the reaction coordinate; and (iii) it evaluates the influence of the dihedral angle θ between two Ru–N–C planes of the chelate on reactivity, rather than that of the Ru–N–C angles. These differences may be key in providing the structural insight that was needed to capture, as we do now, a correlation between the geometry of ruthenium polypyridyl compounds and their photosubstitution reactivity.

To see if our computational insights could be translated into the design of improved red-light photoactive compounds, we investigated the synthesis of [Ru­(dpn)­(i-Hdiqa)­(AcMet)]­Cl_2_ ([Ru10]­Cl_2_), the analogue of [Ru1]­Cl_2_ bearing the i-Hdiqa bidentate chelate instead of dppz. According to our results, the dpn tridentate ligand should provide excellent absorption properties in the far-red region of the spectrum, while the nonrigid and nonplanar i-Hdiqa bidentate chelate should switch on photosubstitution properties. As a first step, we successfully prepared the chlorido precursor [Ru­(dpn)­(i-Hdiqa)­Cl]­Cl ([pRu10]­Cl, see Figure S92). While this complex could be purified, it proved unstable within ∼4 h at room temperature in CD_3_OD (Figure S93). This reactivity is unusual, as other chlorido complexes in this series were all thermally stable in such mild conditions; they would only hydrolyze in aqueous solutions. Such methanolysis of [pRu10]­Cl suggested increased steric hindrance in this complex compared with other chlorido analogues in the series. And indeed, all attempts to isolate the thioether analogue [Ru10]­Cl_2_ by the reaction of [pRu10]Cl and AcMet were unsuccessful (Figure S94): the free i-Hdiqa chelate was clearly observed at *m*/*z* = 272.1 by mass spectrometry analysis of the reaction mixture, demonstrating decomposition of the complex in such reaction conditions. Static DFT modeling of the target complex [Ru10]^2+^ suggested a high steric hindrance, as the structural distortion of the dpn ligand within the ruthenium coordination sphere was high (Figure S116). In fact, these results made us realize that our AQUAMEPTH modeling approach does not address the question of thermal stability of the complex, which should be a primary feature of any candidate for light-triggered photosubstitution. As a side note, these findings highlighted the need for theoretical modeling tools that can rapidly and reliably predict the experimental thermal stability of a metal complex in solution. Overall, the finding that *N*,*N*′-dipyridylamine chelates affords comparatively photosensitive ruthenium complexes is clearly demonstrated by our experimental results on [Ru7]^2+^-[Ru9]^2+^, but combining this type of bidentate chelate with a tridentate chelate that further shifts the absorption to the NIR domain of the spectrum will require additional studies, including thermal stability as a prerequisite.

## Conclusion

In this study, we synthesized a series of nine [κ^3^, κ^2^, κ^1^] ruthenium polypyridyl complexes bound to a monodentate thioether ligand, eight of which were photosubstitutionally active under green- and red-light irradiation. Among them, three compounds, [Ru7]^2+^, [Ru8]^2+^, and [Ru9]^2+^, exhibited remarkable red-light photosubstitution rates, which affording full activation within minutes of irradiation with milliwatt LEDs. We confirmed that the commonly used ^3^MLCT-^3^MC barrier metric and the electronic richness of the bidentate ligand were not reliable predictors of photosubstitution quantum yields. To find better molecular design principles for this family of molecules, we explored the reaction dynamics of the photosubstitution and developed an innovative simulation protocol called AQUAMEPTH. This new protocol provided unexpected insights into the geometry of the attack of the entering aqua ligand and the mechanism of photosubstitution. Such insights are normally unattainable with static DFT and prohibitively expensive with DFT-based dynamics simulations. Our GFN1-xTB-based dynamic studies were not so computationally costly but strong at making prediction. It revealed a previously unknown reaction pathway for monodentate ligand photosubstitution, which could take place not only via the conventional *cis* approach but also via a *trans* approach. In addition, our protocol allowed us to correlate for the first time a lower planarity of the bidentate ligand with higher photosubstitution quantum yields. This result highlights the importance of including nonplanar ancillary ligands to generate space in the first coordination sphere of the complex for the incoming solvent molecule to attack via the *trans* pathway. Furthermore, we were able to distinguish between photoactive and nonphotoactive species with remarkable accuracy, despite employing a semiempirical level of theory.

While we do not claim that the current results translate directly to all classes of ligands and ruthenium complexes, the protocol itself was designed to be transferable: it is built on fundamental principles of monodentate metal–ligand interactions and grounded in electronic structure theory, which preserves the relevant physics across different donor types (e.g., pyridine-, nitrile-, amine-, and sulfur-based ligands). In principle, structural and dynamic descriptors similar to those employed here may be applied to new systems, although doing so would require new and dedicated simulations. Still, our protocol is to our knowledge the first successful modeling attempt to predict different types of photosubstitution mechanisms across a series of structurally similar metal complexes, and this within a reasonable computational time. It offers a practical solution for overcoming the current challenges in simulating condensed-phase photosubstitution reactions. Clearly, the insights gained from these simulations will provide valuable guidance for optimizing the design of next-generation PACT compounds.

## Experimental Section

Ligands *N*-acetyl-l-methionine (AcMet), 4,4′-bis­(trifluoromethyl)- 2,2′-bipyridine (CF_3_-bpy), 2,2′-bipyridine (bpy), 4,4′-dimethoxy-2,2′-bipyridine (Ome-bpy), and di­(pyridin-2-yl)­amine (Hdpa) were purchased from commercial suppliers and used as received. Ligands 2,6-di­(1,8-naphthyridin-2-yl)­pyridine (dpn), *N*-methyl-[2,2′:6′,2″-terpyridine]-4′-carboxamide (tpa), 3,3′-biisoquinoline (i-biq), dipyrido­[3,2-a:2′,3′-*c*]­phenazine (dppz), di­(isoquinolin-3-yl)­amine (i-Hdiqa) and *N*-methyl-*N*-(pyridin-2-yl)­pyridin-2-amine (Medpa), as well as [Ru­(tpa)­Cl_2_]_2_, were synthesized as previously reported.^3,16,45,85^ All reactions were performed under the exclusion of air and light unless stated otherwise.

### Synthesis of [pRu1]­Cl

A three-neck round-bottom flask equipped with nitrogen purge and a condenser was charged with dpn (300 mg, 0.90 mmol, 1.00 equiv) and EtOH (100 mL, flushed with nitrogen for 30 min before). Then, an aqueous solution of RuCl_3_·3H_2_O was added (250 mg, 0.96 mmol, 1.07 equiv). The reaction mixture was stirred well and refluxed for 3 h. Then, dppz (253 mg, 0.90 mmol, 1.00 equiv) and triethylamine (3 mL) were added, and the reaction mixture was refluxed for 24 h. The reaction mixture was concentrated under reduced pressure. The residue was treated with H_2_O (10 mL), and the precipitate was isolated by filtration. The precipitate was washed with H_2_O (20 mL) and Et_2_O (20 mL) and dry-loaded on a column for purification (gradient eluent DCM:MeOH 10:0 to 9:1). Further purification by an SEC column in MeOH yielded the final product in 18% yield (124 mg, 0.15 mmol). ^1^H NMR (600 MHz, MeOD) δ 10.75 (dd, J = 5.2, 1.4 Hz, 1H), 10.03 (dd, J = 8.2, 1.4 Hz, 1H), 9.19 (dd, J = 8.0, 1.3 Hz, 1H), 9.08 (d, J = 8.1 Hz, 2H), 8.80 (d, J = 8.6 Hz, 2H), 8.58 (ddd, J = 8.7, 1.4, 0.7 Hz, 1H), 8.53 (d, J = 8.6 Hz, 2H), 8.45 (dd, J = 8.2, 5.2 Hz, 1H), 8.40–8.34 (m, 2H, Hg), 8.25 (dd, J = 8.0, 1.9 Hz, 2H), 8.13 (ddd, J = 8.5, 6.8, 1.4 Hz, 1H), 8.06 (ddd, J = 8.3, 6.8, 1.4 Hz, 1H), 7.91 (m, 3H, Hc), 7.31 (m, 3H, Hb). ^13^C NMR (151 MHz, MeOD) δ 163.57, 161.54, 158.75, 155.56, 155.19, 154.18, 152.64, 143.89, 143.53, 141.65, 140.51, 140.10, 138.51, 134.43, 132.80, 132.50, 132.43, 132.21, 130.38, 130.35, 129.78, 129.52, 126.09, 125.89, 125.69, 125.62, 124.71, 120.96. ESI MS calculated for [C_39_H_23_N_9_RuCl]^+^: 754.1, measured: 754.1.

### General Procedure [pRu2]­Cl–[pRu9]­Cl

Compounds [pRu2]­Cl–[pRu9]Cl were synthesized according to the general procedure (see Supporting Information for more details and characterization information). [Ru­(tpa)­Cl_2_]_2_ (50 mg, 0.05 mmol, 1.0 equiv) and the bidentate ligand *N–N* (0.1 mmol, 2.0 equiv) were added to a microwave vial along with ethylene glycol (2 mL) as the solvent. The mixture was degassed by bubbling nitrogen gas through it for 10 min. The reaction mixture was then stirred and heated in a sealed vial at 180 °C for 1.5 h with a conventional block. After the completion of the reaction, the solvent was removed under reduced pressure to yield the crude product as a dark red powder. The product was purified using column chromatography on silica gel with a dichloromethane/methanol gradient (0–10% methanol). This yielded the final product as a dark red powder in 58–85% yield.

### General Procedure [Ru1]­Cl_2_–[Ru9]­Cl_2_

Compounds [Ru1]­Cl_2_–[Ru9]­Cl_2_ were synthesized according to the general procedure (see Supporting Information for more details and characterization information). [pRuX]Cl (0.07 mmol, 1.0 equiv) and *N*-acetly-(l)-methionine (0.56 mmol, 8.0 equiv) were suspended in 4 mL of H_2_O in a microwave vial. The mixture was degassed by bubbling nitrogen gas through it for 10 min. The reaction mixture was then stirred and heated in the sealed vial at 100 °C for 16 h with conventional heating. After cooling to room temperature, the product was precipitated as [RuX]­(PF_6_)_2_ salt by the addition of saturated KPF_6_ water (satKPF_6aq._,1 mL). The solid was isolated by centrifugation and subsequently purified by flash column chromatography on silica gel and the acetone/H_2_O/satKPF_6aq._ 8:1:1 eluent mixture. The hexafluorophosphate counterions were exchanged with chloride anions through an anion-exchange column. The purity of the compounds was confirmed by analytical HPLC (Figures S59–S61). The final product was obtained as a bright red solid in 46–89%yield.

### Photosubstitution Studies Monitored by UV–vis Absorption Spectroscopy and MS

The absorption spectra were recorded in water at 298 K under air using a macro spectrophotometer cuvette for 3 mL from Hellma Analytics (lightpath: 1 cm) and an Agilent Technologies Cary 60 UV–vis spectrometer equipped with a Cary Single Cell Peltier Accessory for temperature control, and a previously reported custom-built setup for irradiation with LEDs.^18^ The light intensities at the irradiation wavelength were measured using Nova Power Meter from Ophir Photonics, and the respective photon fluxes were determined using potassium ferrioxalate actinometry (515 nm LED: 3.3 mW, 2.21 × 10^–7^ mol s^–1^; 625 nm LED: 12.4 mW, 5.97 × 10^–7^ mol s^–1^).^14^ The modeling of photosubstitution reaction kinetics was done using Glotaran 1.5.1 and R 4.2.2 (Figures S71–78). The calculations of the photosubstitution quantum yields were done by a previously described method.^14,16,18^ Mass spectrometry was performed before and after the irradiation experiments to identify the photoproducts.

### Phosphorescence Quantum Yields

The steady-state emission measurements were performed on a previously reported custom-built setup utilizing a slightly modified experimental procedure, the results are presented in Table S1 and Figure S63.^86^ [Ru­(bpy)_3_]­Cl_2_ was used as a reference with reported Φ_P_ = 0.040 ± 0.002 in air-saturated H_2_O.^87^ All of the compounds were dissolved in 3 mL of H_2_O and transferred into a macro fluorescence cuvette from Hellma Analytics (lightpaths: 1 cm × 1 cm). The irradiation of samples was done at 298 K using a 450 nm LRD 0450 Laserglow fiber-coupled laser set to 80 mW at the cuvette with the help of PM100USB Thorlabs power meter. The UV–vis and emission spectra were recorded at 298 K with an Agilent Cary 60 UV–vis and Avantes 2048L StarLine spectrometers, respectively. The emission spectra were acquired within 100 ms. All the spectral data were processed with OriginPro 9.1 and MS Excel 2016.

### Singlet Oxygen Quantum Yield Measurement

The steady-state singlet oxygen emission measurements were performed on a previously reported custom-built setup utilizing a slightly modified experimental procedure; results are presented in Table S2 and Figures S64–66.^87–89^ Perinaphthenone was used as a reference with reported Φ_Δ_ (^1^O_2_) = 0.98 ± 0.07 in air-saturated acetonitrile.^90^ [Ru­(bpy)_3_]­Cl_2_ was used as validation compound with reported Φ_Δ_ (^1^O_2_) = 0.57 ± 0.06 in air-saturated acetonitrile.^91^ The NIR spectra were acquired within 20 s at 298 K with an Avantes NIR256-1.7TEC spectrometer. Other conditions were similar to those described in the Phosphorescence Quantum Yields section above.

### Computational Methods

Static DFT calculations were performed with the ADF 2023 package.^92^ The hybrid PBE0 exchange-correlation functional was used, with a TZP basis set for the ruthenium atom and a DZP basis set for the other atoms.^56,57^ Relativistic effects were included using the ZORA method,^59^ and dispersion interactions were accounted for using Grimme’s D3 correction with BJ damping.^58^ Implicit solvent effects were modeled using the COSMO approach in water.^60–62^ Ground-state geometries were optimized using restricted Kohn–Sham DFT, while triplet states were determined with the unrestricted Kohn–Sham formalism, employing the collinear approximation.^93,94^ The numerical quality was set to good during the optimization.

All molecular dynamics simulations were performed with CP2K and with a QM/MM setup.^74,95,96^ Each compound was initially solvated in a 30 × 30 × 30 Å box with a total of 860 water molecules. We used the OPC3 force field to model the water at the molecular mechanical level, where we added Lennard–Jones parameters to prevent electron spill from the hydroxyl group of the GAFF force field.^97,98^ Lennard–Jones parameters for the ruthenium compound were obtained from literature.^99^ The QM system, which consisted of the ruthenium compound and the dynamically assigned water molecules, were treated at the GFN1-xTB level. We coupled the QM and MM box electrostatically via a Coulomb potential term. The QM box was set to nonperiodic, and the Martyna Tuckerman Poisson solver was used.^100^ The periodicity of the MM system was set to xyz, and the periodic Poisson solver was used, where we used smooth particle mesh Ewald summation with an α of 0.35 and a number of grid points of 80.^101^

The structures of the solvent and the ruthenium compound were initially minimized at the QM/MM level. Then, the temperature of the system was increased via simulated annealing in an NVT ensemble, increasing the temperature in four steps at 60 K and simulating for 5 ps. The system was finally simulated for 30 ps at 300 K. Subsequently, an NPT simulation was performed at 300 K and at 1 bar for 50 ps with a barostat time constant of 150 fs. The average box size dimensions were determined and used in subsequent simulations. During the NVT and NPT simulations, a thermostat was used with a time constant of 150 fs.

After the initial equilibration phase, we switched to the adaptive buffer QM/MM method, where we kept GFN1-xTB as the QM level and OPC3 as the MM level of theory. We determined the radius parameters for the adaptive buffer method by evaluating the force differences on a solvated compound configuration that was ^3^P-like (see Figure S95) between a fully DFT-calculated system and systems using various radius parameters. Ultimately, the best compromise between accuracy and efficiency resulted in the following radii: *r*_QM_ 2.5–3.0 Å and *r*_buf_ 0.5–1.0 Å (see Figures S96 and S99). As required for adaptive buffer methods, we used an adaptive Langevin thermostat that was coupled to each degree of freedom, so to obtain the correct temperature profile.^102^

Enhanced sampling calculations were performed with the PLUMED interface to CP2K.^103^ We used umbrella sampling simulations to model the dissociation of the ruthenium sulfur bond. The equilibrium bond length of the initial slice was set at 2.50 Å, with a force constant of 350 kJ/mol/Å. An initial equilibration phase of 5 ps was performed before starting a production run of 40 ps, where we included a well-tempered metadynamics bias along the minimum distance between ruthenium and the oxygen atom of all water molecules. The pace of the bias was set to 50 frames, the height of the Gaussian to 2.0 kJ/mol, the Gaussian width to 0.1 Å, and the bias factor to 15. After 40 ps, we increased the equilibrium distance with 0.15 Å and repeated the equilibrium and production run. This was repeated until we obtained 20 slices, ranging from 2.50 to 5.35 Å.

After all molecular dynamics simulations were performed, we obtained configurations using the delta-net algorithm to obtain a quasi-uniform distribution.^104^ DFT single-point calculations were then performed with CP2K, treating the same atoms at the QM level as in the extracted configurations. A plane wave basis was used where we used the DZVP-MOLOPT-GTH basis set to describe the valence electrons using a cutoff of 280 Ry as well as 5 multigrids.^75^ The core electrons were modeled with PBE-optimized pseudopotentials.^105^ The GGA PBE functional was used, and dispersion interactions were accounted for using Grimme’s D3 correction with a 16 Å cutoff.^73,76^ We coupled the QM and MM box electrostatically via the fast Gaussian expansion of the electrostatic potential.^95,96^ These single-point calculations were used to map out the Mulliken spin surfaces.

## Supplementary Material



## Notes

Experimental procedures, photosubstitution experiments, X-ray crystallographic data, and all computational results, including the free energy surfaces for each compound (PDF)
