# Supplementary material for: Structure over States: Planarity, Not Energy, Dictates Photoactivation in Ru(II) PACT Agents
Source: J Am Chem Soc. 2025 Sep 22;147(39):35767–87. doi: 10.1021/jacs.5c12226 (PMC12498397; doi:10.1021/jacs.5c12226)
Supplement: Supplementary file 1 [file ja5c12226_si_001.pdf]

Supporting Information

Structure Over States: Planarity, Not Energy, Dictates Photoactivation in Ru(II) PACT Agents

Matthijs L. A. Hakkennes,[†#] Irene Regeni, [†‡#] Yurii Husiev,[†] Valeriia D. Andreeva,[†] Maxime A. Siegler,[°] Francesco Buda,*[†] Sylvestre Bonnet*[†]

[†]Leiden Institute of Chemistry, Leiden University, P.O. Box 9502, 2300 RA Leiden, The Netherlands; [‡]Institut de Science et d'Ingénierie Supramoléculaires (ISIS), Strasbourg University, France; [°]Department of Chemistry, Johns Hopkins University, 3400 N Charles Street, Baltimore, MD, 21218, United States.

* Corresponding author: f.buda@chem.leidenuniv.nl, bonnet@chem.leidenuniv.nl

[#] M.L.A.H. and I.R. contributed equally. All authors have given approval to the final version of the manuscript

Contents

1. Methods and Materials 3
2. Synthesis 4
[Ru(dpn)(dppz)Cl]Cl – [pRu1]Cl 4
[Ru(tpa)(*N-N*)Cl]Cl – [pRu2]Cl - [pRu9]Cl 6
[Ru(tpa)(CF_3-bpy)Cl]Cl – [pRu2]Cl 6
[Ru(tpa)(bpy)Cl]Cl – [pRu3]Cl 8
[Ru(tpa)(OMe-bpy)Cl]Cl – [pRu4]Cl 9
[Ru(tpa)(dppz)Cl]Cl – [pRu5]Cl 11
[Ru(tpa)(i-biq)Cl]Cl – [pRu6]Cl 12
[Ru(tpa)(i-Hdiqa)Cl]Cl – [pRu7]Cl 14
[Ru(tpa)(Hdpa)Cl]Cl – [pRu8]Cl 16
[Ru(tpa)(Medpa)Cl]Cl – [pRu9]Cl 17
[Ru(dpn)(dppz)(NAcMet)]Cl_2 – [Ru1]Cl_2 19
[Ru(tpa)(*N-N*)(NAcMet)]Cl_2 – [Ru2]Cl_2 – [Ru9]Cl_2 21
[Ru(tpa)(CF_3-bpy)(NAcMet)]Cl_2 – [Ru2]Cl_2 21
[Ru(tpa)(bpy)(NacMet)]Cl_2 – [Ru3]Cl_2 23
[Ru(tpa)(OMe-bpy)(NAcMet)]Cl_2 – [Ru4]Cl_2 25
[Ru(tpa)(dppz)(NAcMet)]Cl_2 – [Ru5]Cl_2 27
[Ru(tpa)(i-biq)(NAcMet)]Cl_2 – [Ru6]Cl_2 29
[Ru(tpa)(i-Hdiqa)(NAcMet)]Cl_2 – [Ru7]Cl_2 31
[Ru(tpa)(Hdpa)(NAcMet)]Cl_2 – [Ru8]Cl_2 33
[Ru(tpa)(Medpa)(NAcMet)]Cl_2 – [Ru9]Cl_2 34
Analytical HPLC 37
3. Photochemistry 40
Absorption and emission properties of [Ru1]Cl_2 -[Ru9]Cl_2 40
Phosphorescence Quantum Yields 42
Ligand Photosubstition Properties 47
4. [Ru(dpn)(i-Hdiqa)(AcMet)]Cl_2 - [Ru10]Cl_2 62
5. Single Crystal X-ray Crystallography 65
6. Computational Methods 68
7. References 87

Abbreviations:

dpn = 2,6-di(1,8-naphthyridin-2-yl)pyridine
tpa = N-methyl-[2,2':6',2''-terpyridine]-4'-carboxamide
dppz = dipyrido[3,2-a:2',3'-c]phenazine
CF_3-bpy = 4,4'-bis(trifluoromethyl)- 2,2'-bipyridine
bpy = 2,2'-bipyridine
Ome-bpy = 4,4'-dimethoxy-2,2'-bipyridine
i-biq = 3,3'-biisoquinoline
i-Hdiqa = di(isoquinolin-3-yl)amine
Hdpa = di(pyridin-2-yl)amine
Medpa = N-methyl-N-(pyridin-2-yl)pyridin-2-amine
NAcMet = N-acetyl-L-methionine
SatKPF$_{6aq.}$ = saturated KF_6 aqueous solution

1. Methods and Materials

Ligands NAcMet, CF_3-bpy, bpy , Ome-bpy and Hdpa were purchased from commercial suppliers and used as received. Ligands dpn, tpa, i-biq, dppz, i-Hdiqa and Medpa, as well as the Ru(II)-tpa dimer $[Ru(tpa)Cl_2]_2$, were synthesized as previously reported.[1–4] All reactions were performed under the exclusion of air and light, unless stated otherwise. All reactions involving ruthenium(II) complexes were carried out in capped 5 mL microwave vials as included in the Biotage Microwave Reaction Kit, 2-5 mL. Reactions were kept at constant temperature and stirring using a MR Hei-Tec Magnetic hotplate stirrer (Heidolph) and a metal heating block with microwave vial place holders. All analyses by thin layer chromatography (TLC) were performed using SiliCycle Aluminum-Backed Silica TLC plates (200 µm). Silica column chromatography was performed using Screening Devices BV silica with a particle size of 40–63 µm and a pore size of 60 Å. Column packing was done by making a silica slurry, where the silica was first dissolved in eluent. For the ion exchange column, DowexTM 22 resin saturated with 1 M HCl in methanol was used. Vacuum filtrations were performed using Whatman regenerated cellulose membrane filters, RC60 membrane circles with a diameter of 50 mm and a pore size of 1 µm. Ultrapure water (Milli-Q water) was obtained using the Milli-Q Advantage A10 water purification system. All NMR spectra were measured at room temperature (298 K), unless stated otherwise, using either a Bruker Avance 400, 500, 600 or 850 MHz NMR spectrometer and were analyzed using MestreNova version 15 software. All chemical shift values were taken as relative to the solvent residual signals as present in the solvent list of MestreNova software (methanol-d_4: δ (1H, quintet) = 3.31 ppm; δ (^{13}C, heptet) = 49.00 ppm; chloroform-*d*: δ (^{1}H, singlet) = 7.26 ppm; acetone-d_6: δ (^{1}H, quintet) = 2.05 ppm). Liquid chromatograph mass spectrometer (LC-MS) spectra were recorded on a Shimadzu LCMS-2020. High-resolution mass spectra (HRMS) were measured on Thermo Finnigan LTQ orbitrap. HPLC purification was performed with a 250 x 21.2 mm Jupiter® 4 µm Proteo 90 Å C12 column and a Thermo Scientific UHPLC system. The gradient was controlled by four pumps. The mobile phase consisted of H_2O containing 0.1% (v/v) formic acid (phase A) and acetonitrile containing 0.1% (v/v) formic acid (phase B). Analytical HPLC analysis was performed on an Alltima 5 µm C18 column with a Thermo Scientific UHPLC system. The gradient was controlled by one pump. The mobile phase consisted of H_2O containing 0.1% (v/v) trifluoroacetic acid (phase A) and acetonitrile containing 0.1% (v/v) trifluoroacetic acid (phase B). All UV-Vis absorption measurements were performed on a Agilent Cary 60 UV-Vis spectrophotometer equipped with a Cary single cell peltier accessory (25 °C and magnetic stir bar) using Hellma 10

mm × 10 mm quartz cuvettes. Emission measurements were performed at 25 °C using a Jasco J-1500 CD spectrometer and Hellma fluorescence quartz cuvettes (10 mm × 10 mm).

2. Synthesis

[Ru(dpn)(dppz)Cl]Cl – [pRu1]Cl

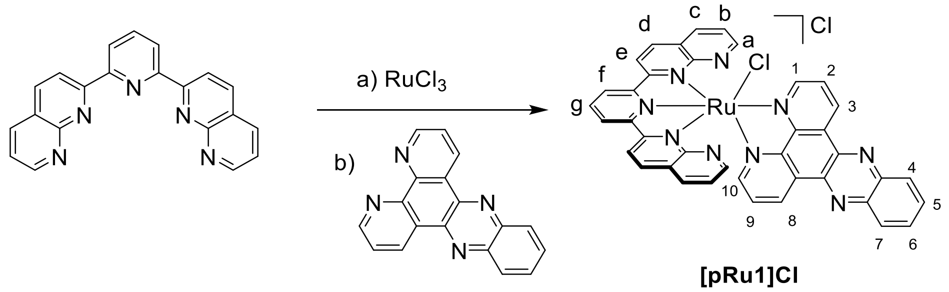

Figure S1: Scheme

A three-neck round-bottom flask equipped with nitrogen purge and a condenser, was charged with dpn (300 mg, 0.90 mmol, 1.00 equiv.) and EtOH (100 mL, flushed with N_2 for 30 min before). Then, aqueous solution of $RuCl_3 \cdot 3H_2O$ was added (250 mg, 0.96 mmol, 1.07 equiv.). The reaction mixture was well-stirred and refluxed for 3 h. Then, dppz (253 mg, 0.90 mmol, 1.00 equiv.) and triethylamine (3 mL) were added, and the reaction mixture was refluxed for 24 h. The reaction mixture was concentrated under reduced pressure. The residue was treated with H_2O (10 mL) and the precipitate was isolated by filtration. The precipitate was washed with H_2O (20 mL) and Et_2O (20 mL) and dry-loaded on a column for purification (gradient eluent DCM:MeOH 10:0 to 9:1). Further purification by SEC column in MeOH yielded the final product in 18% yield (124 mg, 0.15 mmol). ^{1}H NMR (600 MHz, MeOD) δ 10.75 (dd, J = 5.2, 1.4 Hz, 1H, H1), 10.03 (dd, J = 8.2, 1.4 Hz, 1H, H3), 9.19 (dd, J = 8.0, 1.3 Hz, 1H, H8), 9.08 (d, J = 8.1 Hz, 2H, Hf), 8.80 (d, J = 8.6 Hz, 2H, He), 8.58 (ddd, J = 8.7, 1.4, 0.7 Hz, 1H, H4), 8.53 (d, J = 8.6 Hz, 2H, Hd), 8.45 (dd, J = 8.2, 5.2 Hz, 1H, H2), 8.40 – 8.34 (m, 2H, Hg, H7), 8.25 (dd, J = 8.0, 1.9 Hz, 2H, Ha), 8.13 (ddd, J = 8.5, 6.8, 1.4 Hz, 1H, H6), 8.06 (ddd, J = 8.3, 6.8, 1.4 Hz, 1H, H5), 7.91 (m, 3H, Hc, H10), 7.31 (m, 3H, Hb, H9). ^{13}C NMR (151 MHz, MeOD) δ 163.57, 161.54, 158.75, 155.56, 155.19, 154.18, 152.64, 143.89, 143.53, 141.65, 140.51, 140.10, 138.51, 134.43, 132.80, 132.50, 132.43, 132.21, 130.38, 130.35, 129.78, 129.52, 126.09, 125.89, 125.69, 125.62, 124.71, 120.96. ESI MS calculated for $[C_{39}H_{23}N_9RuCl]^+$: 754.1, measured: 754.1.

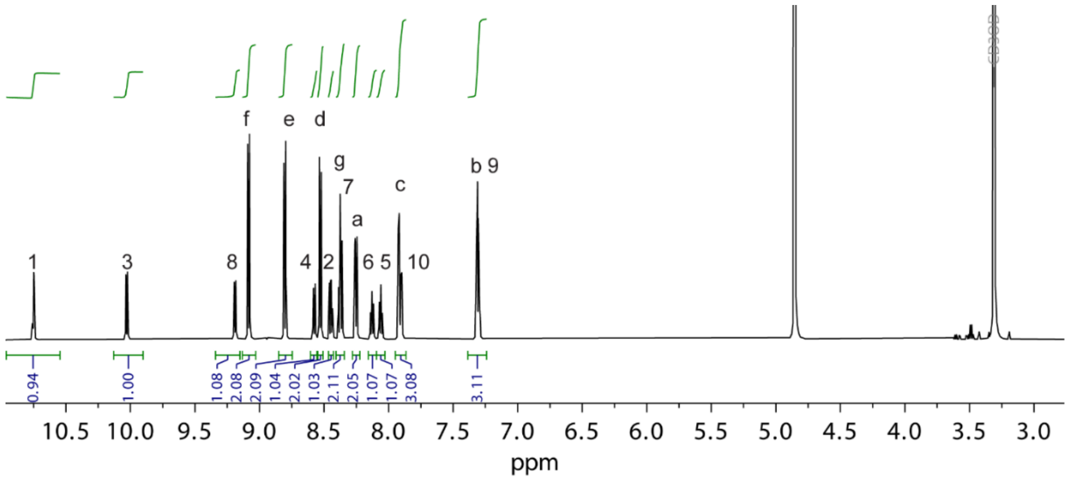

Figure S2: ^{1}H NMR (600 MHz, MeOD) of [pRu1]Cl with proton assignment.

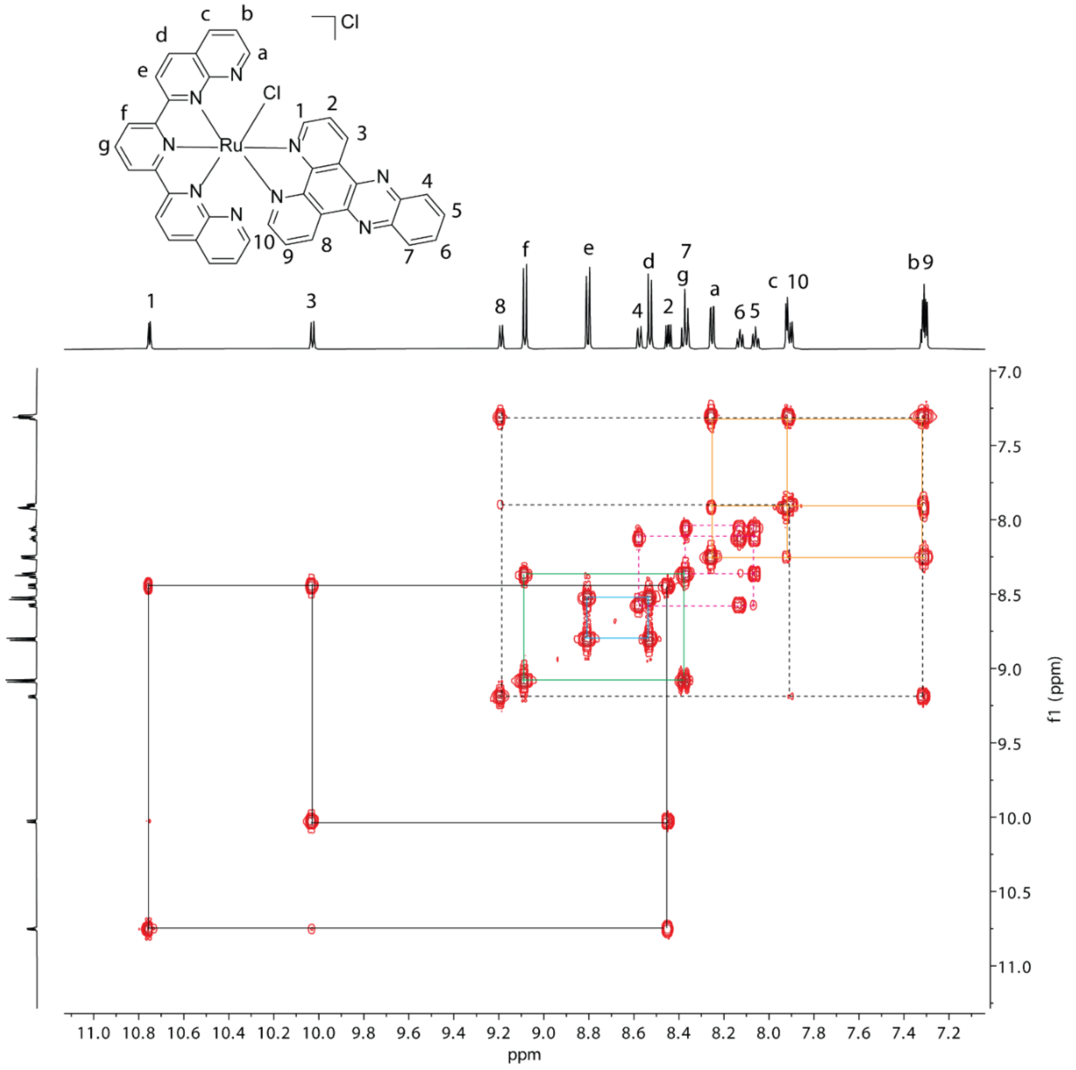

Figure S3: Enlargement of aromatic region ^{1}H-^{1}H COSY (600 MHz, MeOD) of [pRu1]Cl.

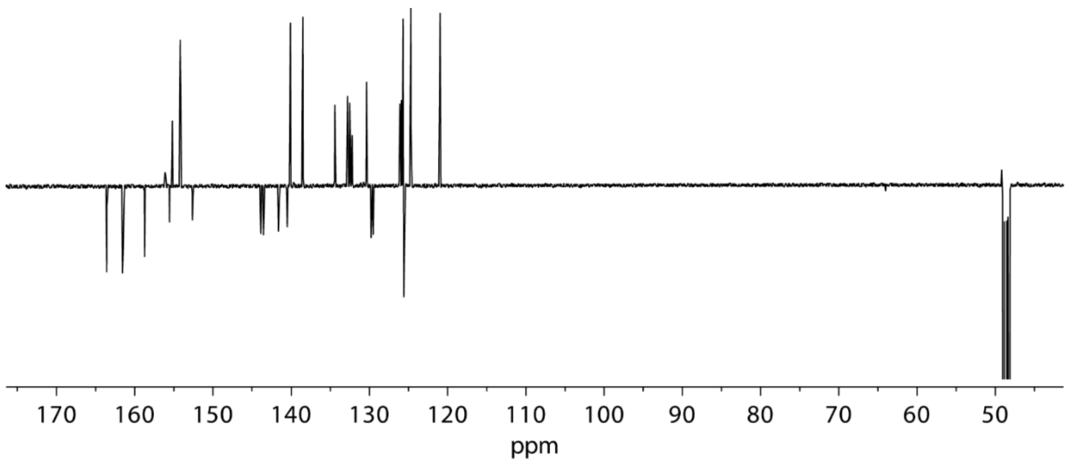

Figure S4: ^{13}C NMR (151 MHz, MeOD) of [pRu1]Cl.

[Ru(tpa)(*N-N*)Cl]Cl – [pRu2]Cl - [pRu9]Cl

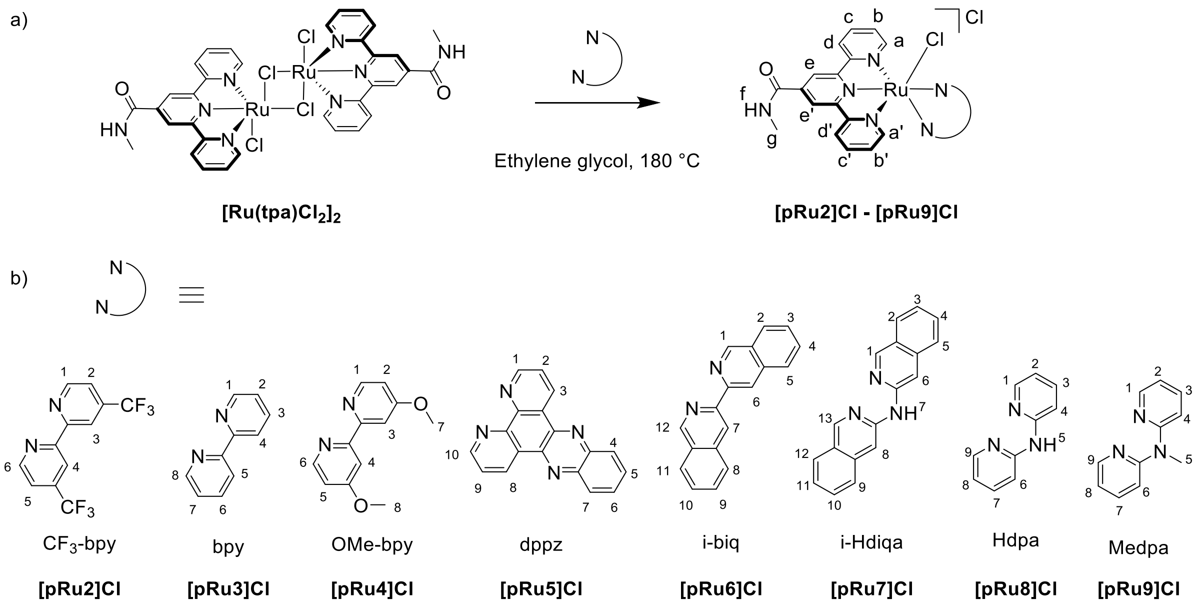

Figure S5: Generic reaction scheme for [pRu2]Cl - [pRu9]Cl and scope of bidentate ligands explored in this study.

[Ru(tpa)(CF_3-bpy)Cl]Cl – [pRu2]Cl

[Ru(tpa)Cl_2]$_2$ (50 mg, 0.05 mmol, 1.0 equiv.) and CF_3-bpy ligand (32 mg, 0.1 mmol, 2.0 equiv.) were added to a microwave vial along with ethylene glycol (2 mL) as the solvent. The mixture was degassed by bubbling nitrogen gas through it for 10 min. The reaction mixture was then stirred and heated in the sealed vial at 180 °C for 1.5 h with a conventional block. After completion of the reaction, the solvent was removed under reduced pressure to yield the crude product as a dark red powder. The product was purified using column chromatography on silica gel with a dichloromethane/methanol gradient (0–10% methanol). This yielded the final product as a dark red powder in 80% yield (65 mg, 0.086 mmol). ^1H NMR (850 MHz, MeOD) δ 10.50 (d, J = 5.8 Hz, 1H, H1), 9.39 – 9.38 (m, 1H, H3), 9.11 (d, J = 1.9 Hz, 1H, H4), 9.07 (s, 2H, He), 8.64 (dt, J = 8.0, 1.1 Hz, 2H, Hd), 8.38 (dd, J = 5.8, 1.9 Hz, 1H, H2), 8.04 (td, J = 7.8, 1.5 Hz, 2H, Hc), 7.78 (d, J = 6.2 Hz, 1H, H5), 7.73 (ddd, J = 5.6, 1.5, 0.8 Hz, 2H, Ha), 7.40 (ddd, J = 7.2, 5.6, 1.3 Hz, 2H, Hb), 7.37 (dd, J = 6.3,

1.9 Hz, 1H, H6), 3.15 (s, 3H, Hg). ^{13}C NMR (214 MHz, MeOD) δ 165.41, 159.80, 158.12, 157.94, 156.98, 153.64, 153.16, 152.35, 140.51, 137.90, 137.77, 137.74, 136.70, 136.54, 127.62, 123.92, 122.93, 122.10, 120.71, 120.23, 120.21, 25.93. ESI MS calculated for $[C_{29}H_{20}N_6F_6ORuCl]^+$: 719.0, measured: 719.1.

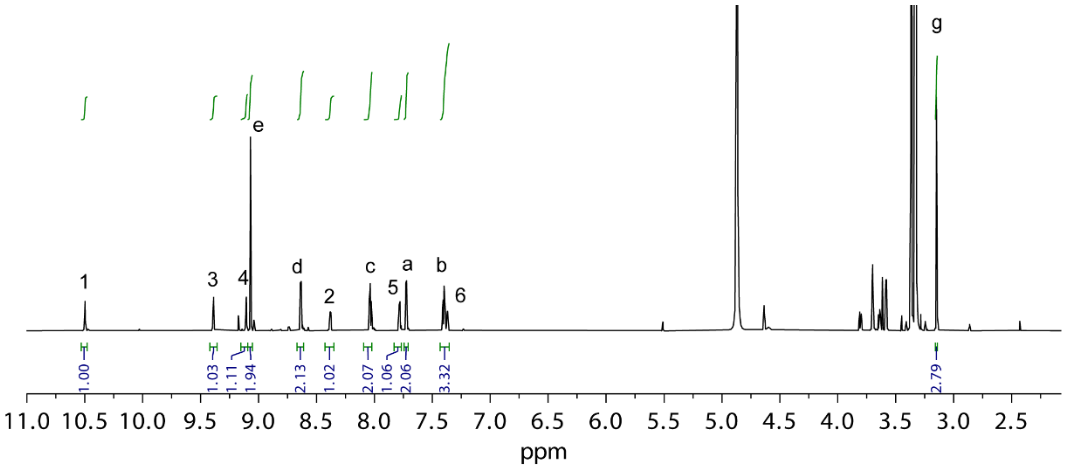

Figure S6: ^{1}H NMR (850 MHz, MeOD) of [pRu2]Cl with proton assignment.

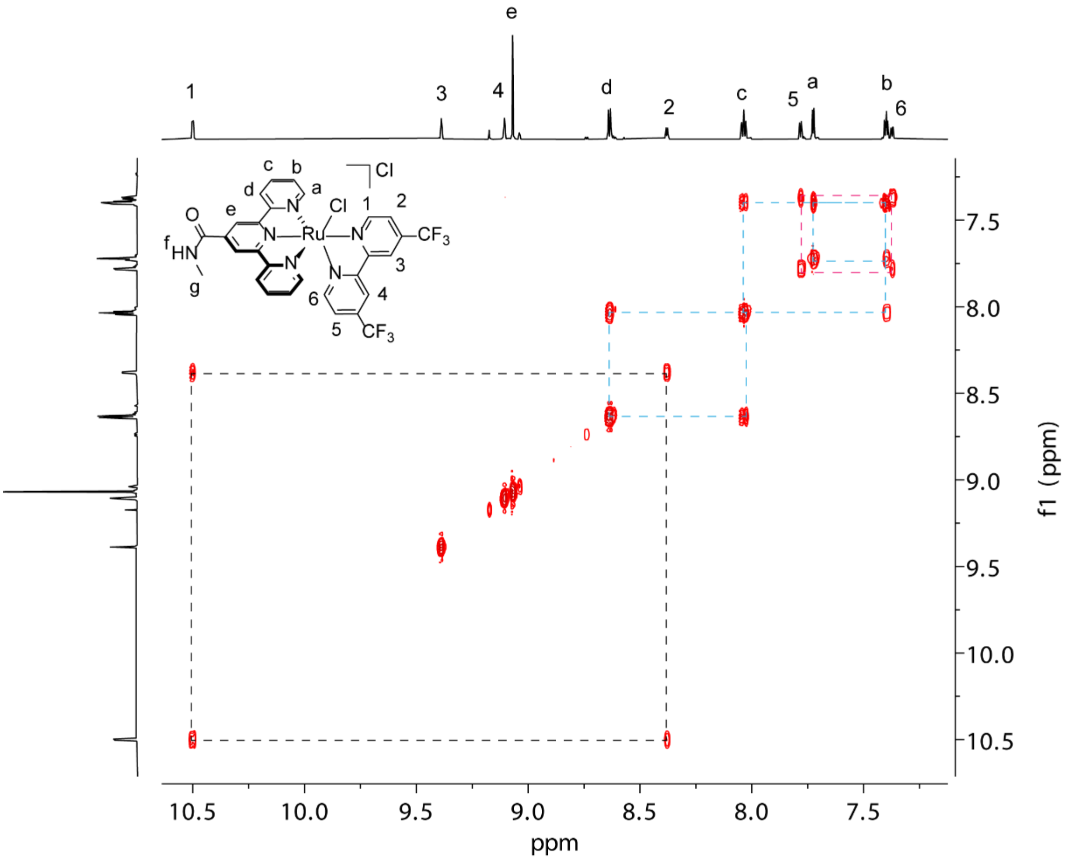

Figure S7 : Enlargement of aromatic region ^{1}H-^{1}H COSY (850 MHz, MeOD) of [pRu2]Cl.

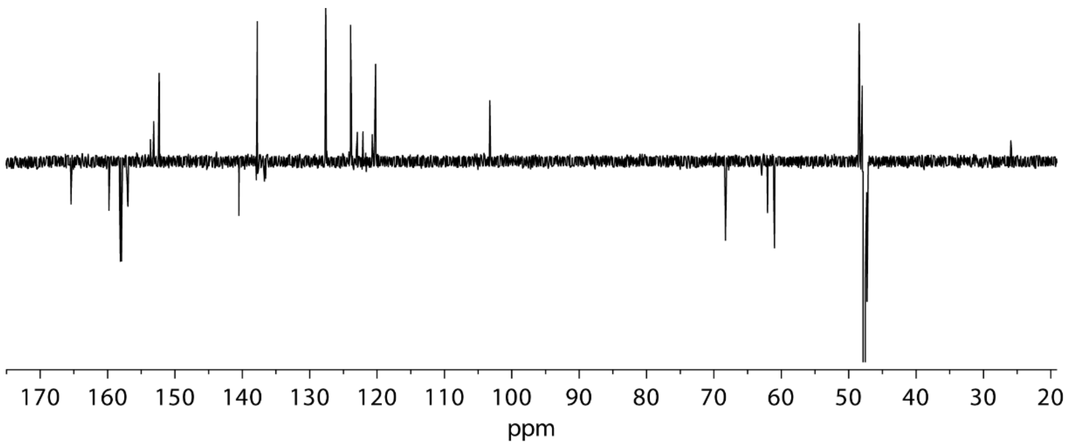

Figure S8 : ^{13}C NMR (214 MHz, MeOD) of [pRu2]Cl.

[Ru(tpa)(bpy)Cl]Cl – [pRu3]Cl

[Ru(tpa)Cl_2]$_2$ (50 mg, 0.05 mmol, 1.0 equiv.) and bpy ligand (17 mg, 0.1 mmol, 2.0 equiv.) were added to a microwave vial along with ethylene glycol (2 mL) as the solvent. The mixture was degassed by bubbling nitrogen gas through it for 10 min. The reaction mixture was then stirred and heated in the sealed vial at 180 °C for 1.5 h with a conventional block. After completion of the reaction, the solvent was removed under reduced pressure to yield the crude product as a dark red powder. The product was purified using column chromatography on silica gel with a dichloromethane/methanol gradient (0–10% methanol). This yielded the final product as a dark red powder in 85% yield (54 mg, 0.9 mmol). ^{1}H NMR (850 MHz, MeOD) δ 10.22 (ddd, J = 5.5, 1.6, 0.8 Hz, 1H, H1), 9.03 (s, 2H, He), 8.81 (dt, J = 8.2, 1.1 Hz, 1H, H4), 8.61 (dt, J = 7.8, 1.1 Hz, 2H, Hd), 8.52 (dt, J = 8.1, 1.1 Hz, 1H, H5), 8.37 (td, J = 7.9, 1.5 Hz, 1H, H3), 8.05 (ddd, J = 7.8, 5.5, 1.3 Hz, 1H, H2), 8.00 (td, J = 7.8, 1.5 Hz, 2H, Hc), 7.77 (ddd, J = 8.1, 7.4, 1.4 Hz, 1H, H6), 7.73 (ddd, J = 5.6, 1.5, 0.8 Hz, 2H, Ha), 7.43 – 7.38 (m, 2H, Hb), 7.36 (ddd, J = 5.9, 1.5, 0.7 Hz, 1H, H8), 7.05 (ddd, J = 7.4, 5.9, 1.3 Hz, 1H, H7), 3.13 (s, 3H, Hg). ^{13}C NMR (214 MHz, MeOD) δ 165.67, 158.62, 158.59, 158.52, 156.00, 152.19, 151.91, 151.47, 139.10, 137.20, 136.99, 135.82, 127.44, 126.80, 126.14, 123.62, 123.44, 123.25, 119.91, 25.90. ESI MS calculated for [$C_{27}H_{22}N_6ORuCl$]$^+$: 583.1, measured: 583.1.

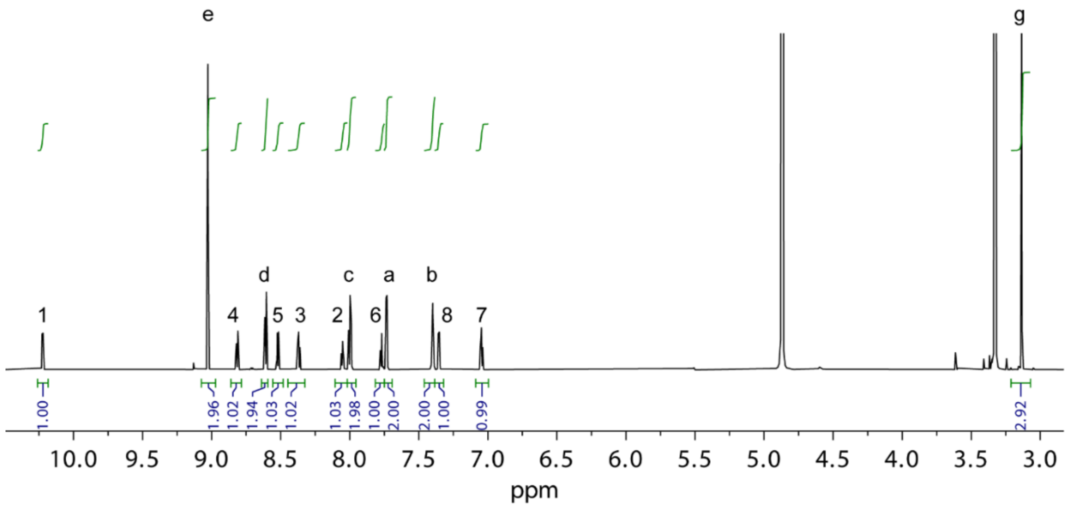

Figure S9: ^{1}H NMR (850 MHz, 298 K) in MeOD of [pRu3]Cl.

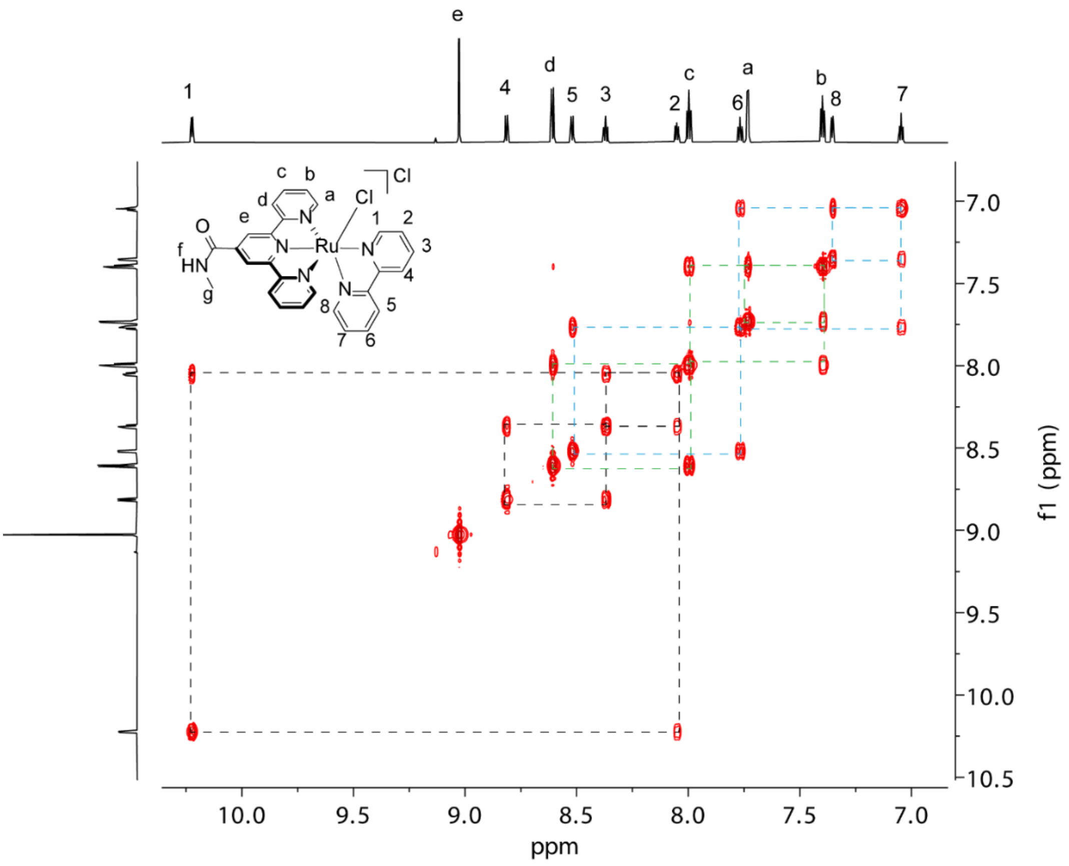

Figure S10: Enlargement of aromatic region ^{1}H-^{1}H COSY (850 MHz, MeOD) of [pRu3]Cl.

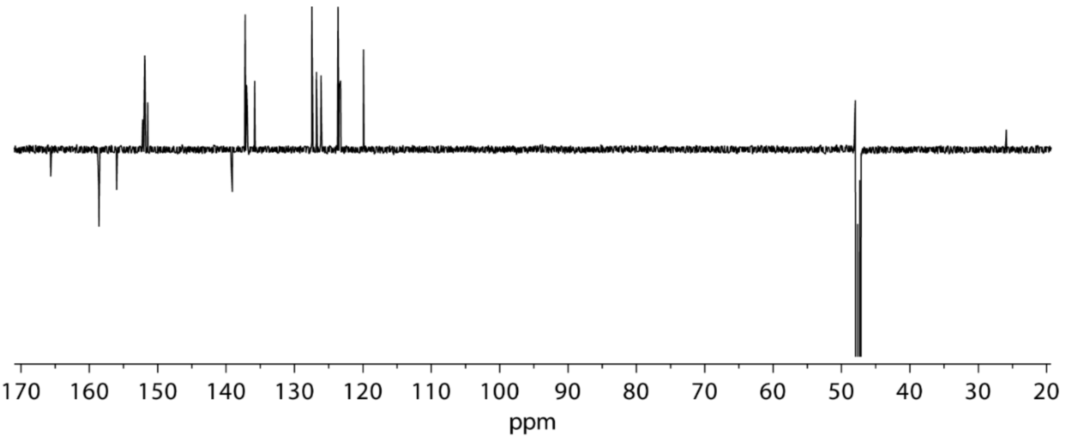

Figure S11: ^{13}C NMR (214 MHz, MeOD) of [pRu3]Cl.

[Ru(tpa)(OMe-bpy)Cl]Cl – [pRu4]Cl

$[Ru(tpa)Cl_2]_2$ (50 mg, 0.05 mmol, 1.0 equiv.) and OMe-bpy ligand (24 mg, 0.1 mmol, 2.0 equiv.) were added to a microwave vial along with ethylene glycol (2 mL) as the solvent. The mixture was degassed by bubbling nitrogen gas through it for 10 min. The reaction mixture was then stirred and heated in the sealed vial at 180 °C for 1.5 h with a conventional block. After completion of the reaction, the solvent was removed under reduced pressure to yield the crude product as a dark red powder. The product was purified using column chromatography on silica gel with a dichloromethane/methanol gradient (0–10% methanol). This yielded the final product as a dark red powder in 75% yield (51 mg, 0.08 mmol). ^{1}H NMR (850 MHz, MeOD) δ 9.95 (d, J = 6.4 Hz, 1H, H1), 8.99 (s, 2H, He), 8.58 (dd, J = 8.0, 1.3 Hz, 2H, Hd), 8.43 – 8.37 (m, 1H, H4), 8.15 – 8.08 (m, 1H,

H5), 7.97 (td, J = 7.6, 1.3 Hz, 2H, Hc), 7.82 (dt, J = 5.7, 2.8 Hz, 2H, Ha), 7.72 – 7.63 (m, 1H, H2), 7.42 (ddd, J = 7.1, 5.5, 1.3 Hz, 2H, Hb), 6.99 – 6.94 (m, 1H, H8), 6.63 (dd, J = 6.8, 2.7 Hz, 1H, H7), 4.25 (s, 3H, H3), 3.87 (s, 3H, H6), 3.11 (d, J = 2.0 Hz, 3H, Hg). ^{13}C NMR (214 MHz, MeOD) δ 167.40, 166.41, 165.74, 159.24, 159.18, 158.78, 156.95, 152.79, 151.85, 151.64, 137.87, 136.78, 127.34, 123.35, 119.67, 113.15, 112.72, 110.15, 110.09, 55.91, 55.64, 25.87. ESI MS calculated for $[C_{29}H_{26}N_6O_3RuCl]^+$: 643.1, measured: 643.1.

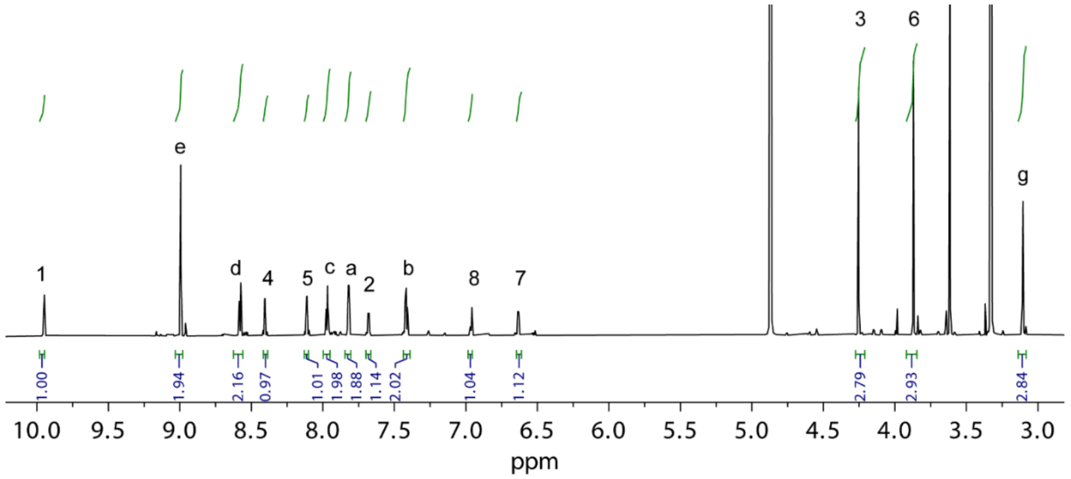

Figure S12 : ^{1}H NMR (850 MHz, MeOD) of [pRu4]Cl with proton assignment.

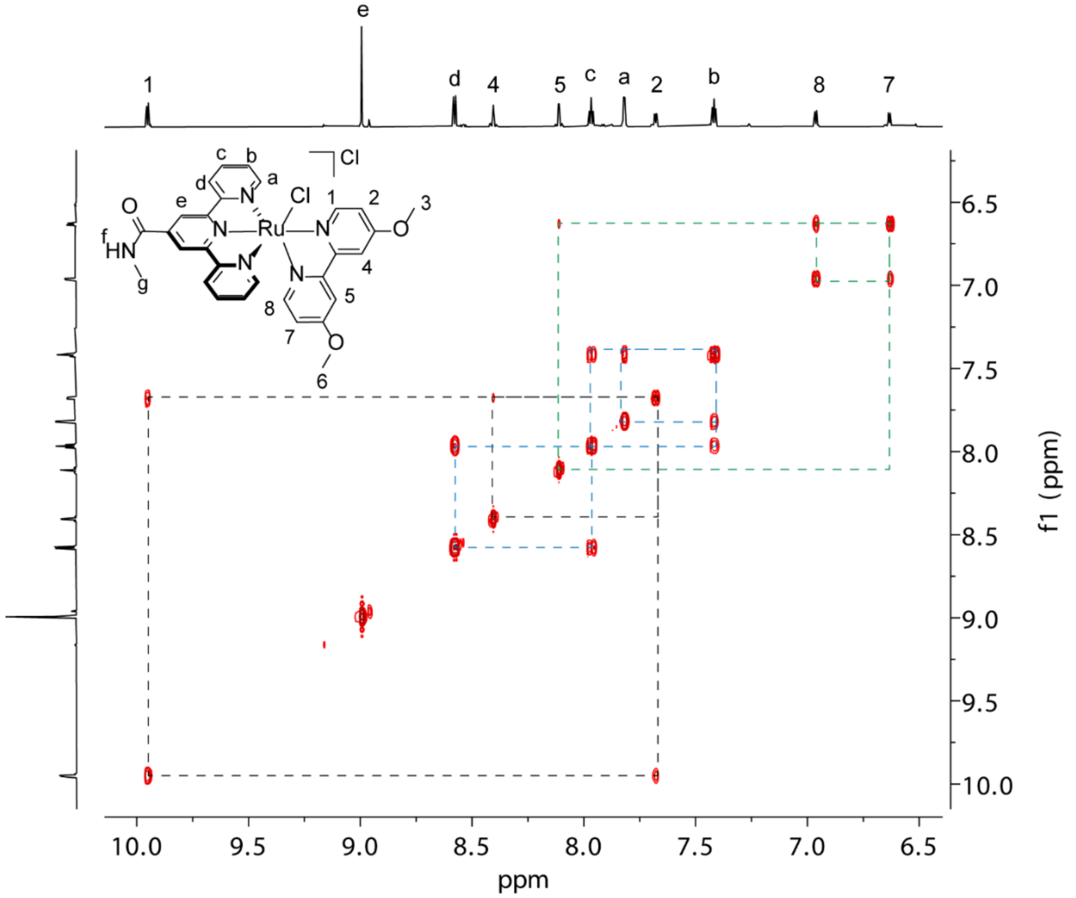

Figure S13 : Enlargement of aromatic region ^{1}H-^{1}H COSY (850 MHz, MeOD) of [pRu4]Cl.

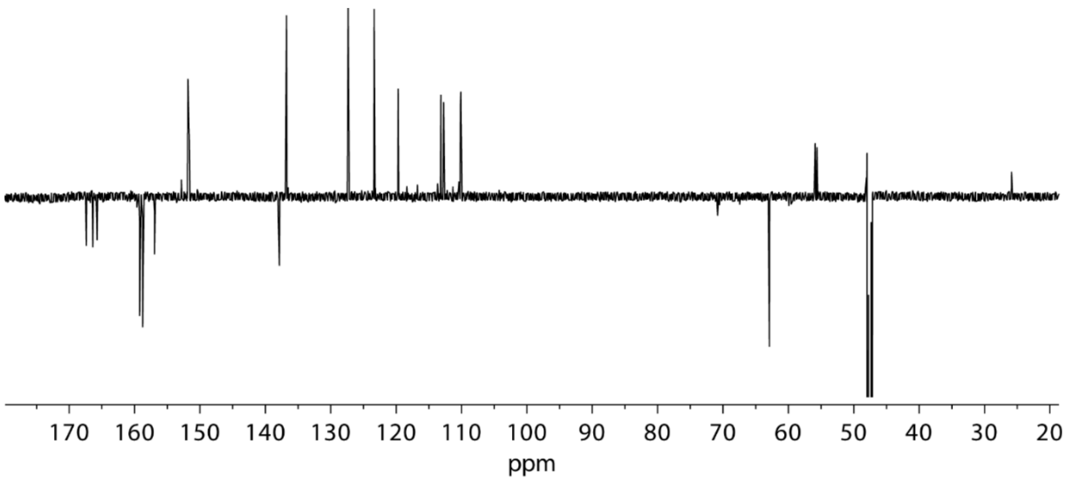

Figure S14 : ^{13}C NMR (214 MHz, MeOD) of [pRu4]Cl.

[Ru(tpa)(dppz)Cl]Cl – [pRu5]Cl

$[Ru(tpa)Cl_2]_2$ (50 mg, 0.05 mmol, 1.0 equiv.) and dppz ligand (31 mg, 0.1 mmol, 2.0 equiv.) were added to a microwave vial along with ethylene glycol (2 mL) as the solvent. The mixture was degassed by bubbling nitrogen gas through it for 10 min. The reaction mixture was then stirred and heated in the sealed vial at 180 °C for 1.5 h with a conventional block. After completion of the reaction, the solvent was removed under reduced pressure to yield the crude product as a dark red powder. The product was purified using column chromatography on silica gel with a dichloromethane/methanol gradient (0–10% methanol). This yielded the final product as a dark red powder in 70% yield (58 mg, 0.08 mmol). ^{1}H NMR (850 MHz, MeOD) δ 10.55 (dd, J = 5.1, 1.4 Hz, 1H, H1), 10.03 (dd, J = 8.3, 1.4 Hz, 1H, H3), 9.45 (dd, J = 8.0, 1.3 Hz, 1H, H8), 9.09 (s, 2H, He), 8.64 (dt, J = 8.0, 1.0 Hz, 2H, Hd), 8.59 (dt, J = 8.8, 1.0 Hz, 1H, H2), 8.56 (dd, J = 8.2, 5.1 Hz, 1H, H4), 8.47 (dt, J = 8.2, 0.9 Hz, 1H, H7), 8.17 (ddd, J = 8.5, 6.7, 1.4 Hz, 1H, H5), 8.13 (ddd, J = 8.2, 6.7, 1.5 Hz, 1H, H6), 8.01 – 7.93 (m, 2H, Hc), 7.85 (dd, J = 5.5, 1.3 Hz, 1H, H10), 7.78 (dt, J = 5.6, 1.2 Hz, 2H, Ha), 7.55 (dd, J = 7.9, 5.5 Hz, 1H, H9), 7.30 (ddd, J = 7.2, 5.8, 1.3 Hz, 2H, Hb), 3.17 (s, 3H, Hg). ^{13}C NMR (214 MHz, MeOD) δ 165.72, 164.12, 161.90, 158.76, 158.51, 154.41, 154.00, 152.39, 142.91, 142.75, 139.91, 139.47, 137.28, 132.90, 131.98, 131.90, 131.73, 130.57, 129.47, 129.41, 127.43, 126.76, 125.93, 123.63, 119.99, 26.56 (3Cq overlapping). ESI MS calculated for $[C_{35}H_{24}N_8ORuCl]^+$: 709.1, measured: 709.1.

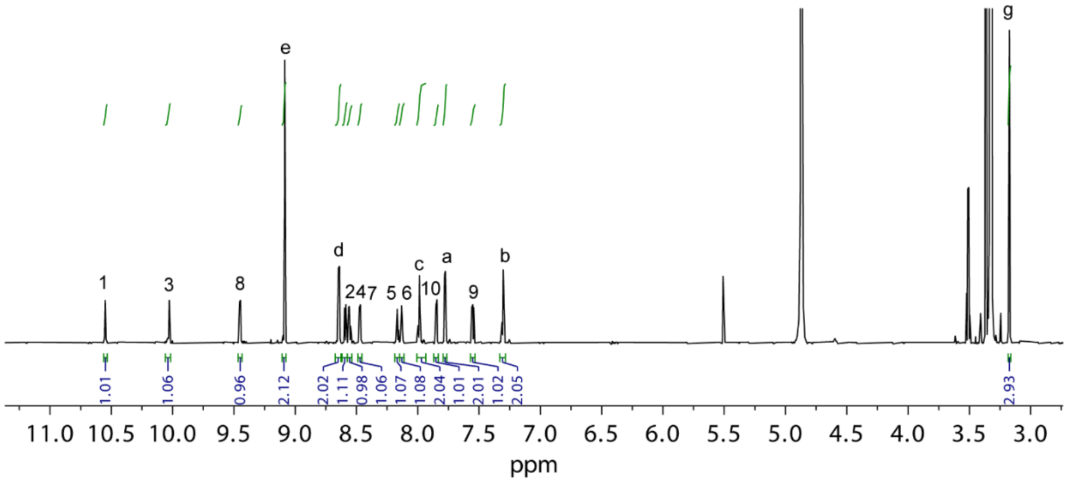

Figure S15 : ^{1}H NMR (850 MHz, MeOD) of [pRu5]Cl with proton assignment.

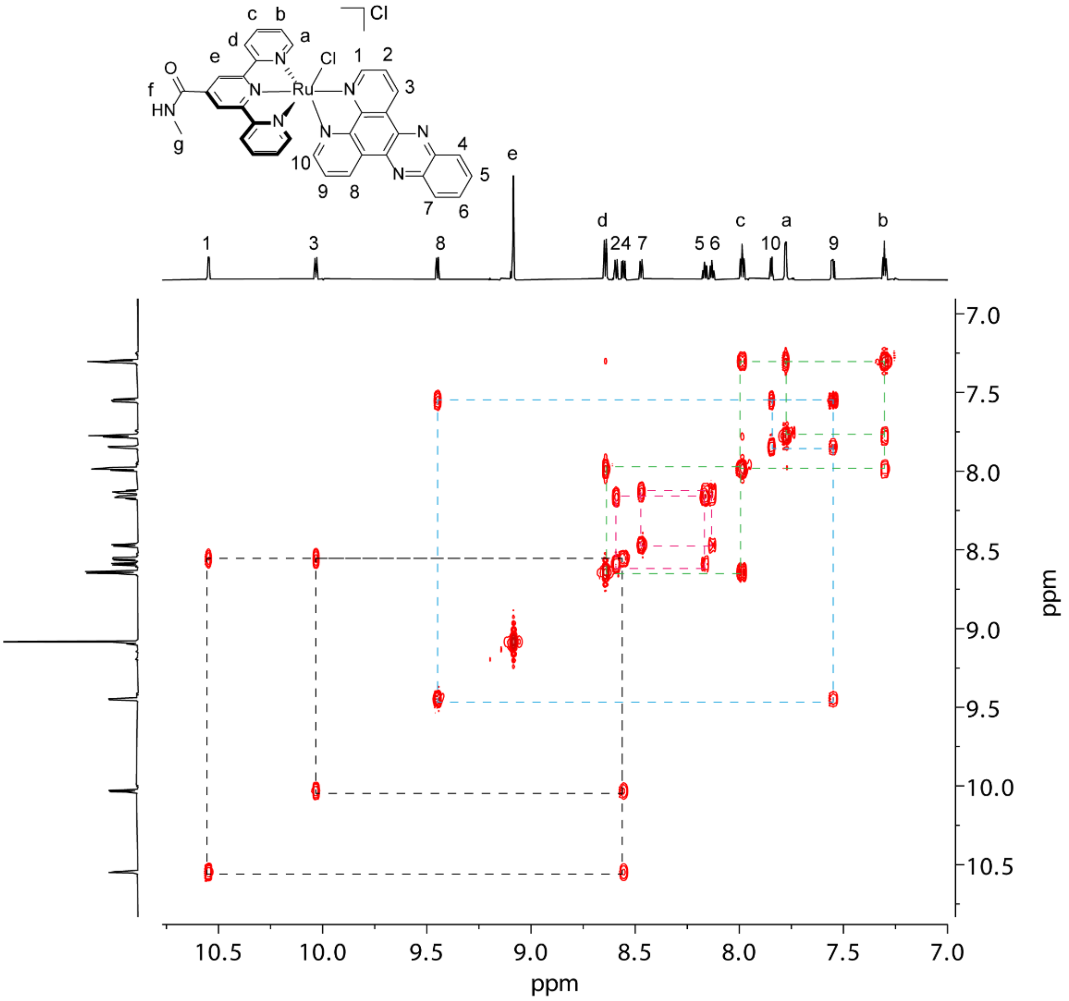

Figure S16 : Enlargement of aromatic region ^1H-^1H COSY (850 MHz, MeOD) of [pRu5]Cl.

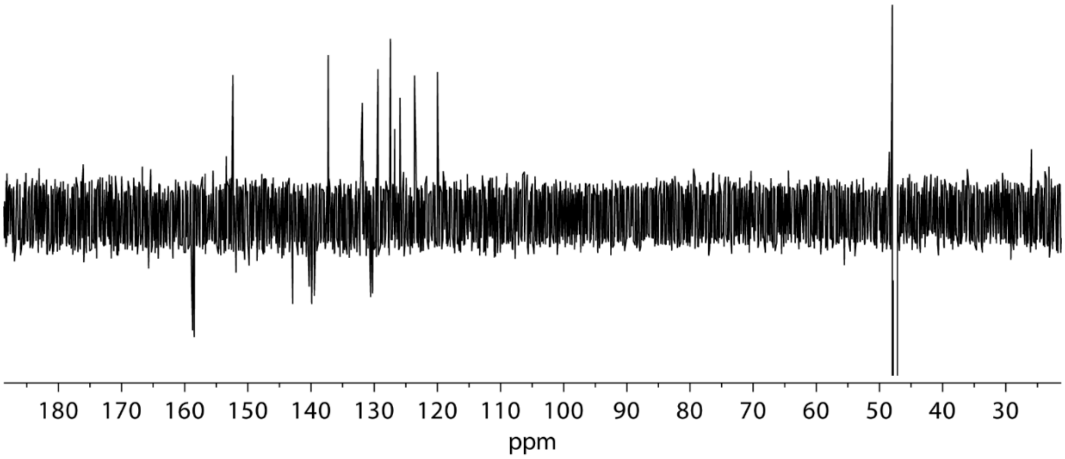

Figure S17 : ^{13}C NMR (214 MHz, MeOD) of [pRu5]Cl.

[Ru(tpa)(i-biq)Cl]Cl – [pRu6]Cl

$[Ru(tpa)Cl_2]_2$ (50 mg, 0.05 mmol, 1.0 equiv.) and i-biq ligand (28 mg, 0.1 mmol, 2.0 equiv.) were added to a microwave vial along with ethylene glycol (2 mL) as the solvent. The mixture was degassed by bubbling nitrogen gas through it for 10 min. The reaction mixture was then stirred and heated in the sealed vial at 180 °C for 1.5 h with a conventional block. After completion of the

reaction, the solvent was removed under reduced pressure to yield the crude product as a dark red powder. The product was purified using column chromatography on silica gel with a dichloromethane/methanol gradient (0–10% methanol). This yielded the final product as a dark red powder in 60% yield (47 mg, 0.06 mmol). ^{1}H NMR (600 MHz, MeOD) δ 10.80 (s, 1H, H1), 9.35 (s, 1H, H6), 9.05 (s, 2H, He), 9.04 (s, 1H, H7), 8.61 (ddd, J = 8.1, 1.1, 1.1 Hz, 2H, Hd), 8.40 (dd, 2H, H5, H2), 8.07 (ddd, J = 8.4, 7.1, 1.2 Hz, 1H, H4), 8.03 (dd, J = 8.3, 1.0 Hz, 1H, H3), 7.99 (ddd, 1H, H11), 7.98 (s, 1H, H12), 7.95 (ddd, J = 8.0, 7.8, 1.5 Hz, 2H, Hc), 7.88 (ddd, J = 5.6, 1.5, 0.7 Hz, 2H, Ha), 7.73 (ddd, 1H, H10), 7.63 (dd, J = 8.5, 1.1 Hz, 1H, H8), 7.56 (ddd, J = 8.2, 6.7, 1.1 Hz, 1H, H9), 7.32 (ddd, J = 7.2, 5.6, 1.3 Hz, 2H, Hb), 3.15 (s, 3H, Hg). ^{13}C NMR (151 MHz, MeOD) δ 167.30, 160.43 (2C), 160.13 (2C), 156.01, 153.14, 152.30, 150.86, 140.25, 138.53, 137.00, 135.84, 133.87, 133.57, 131.34, 131.04, 130.88, 130.30, 128.89, 128.78, 128.49, 127.33, 125.09, 121. 53, 121.31, 120.94, 27.35. ESI MS calculated for $[C_{35}H_{26}N_6ORuCl]^+$: 683.1, measured: 683.1.

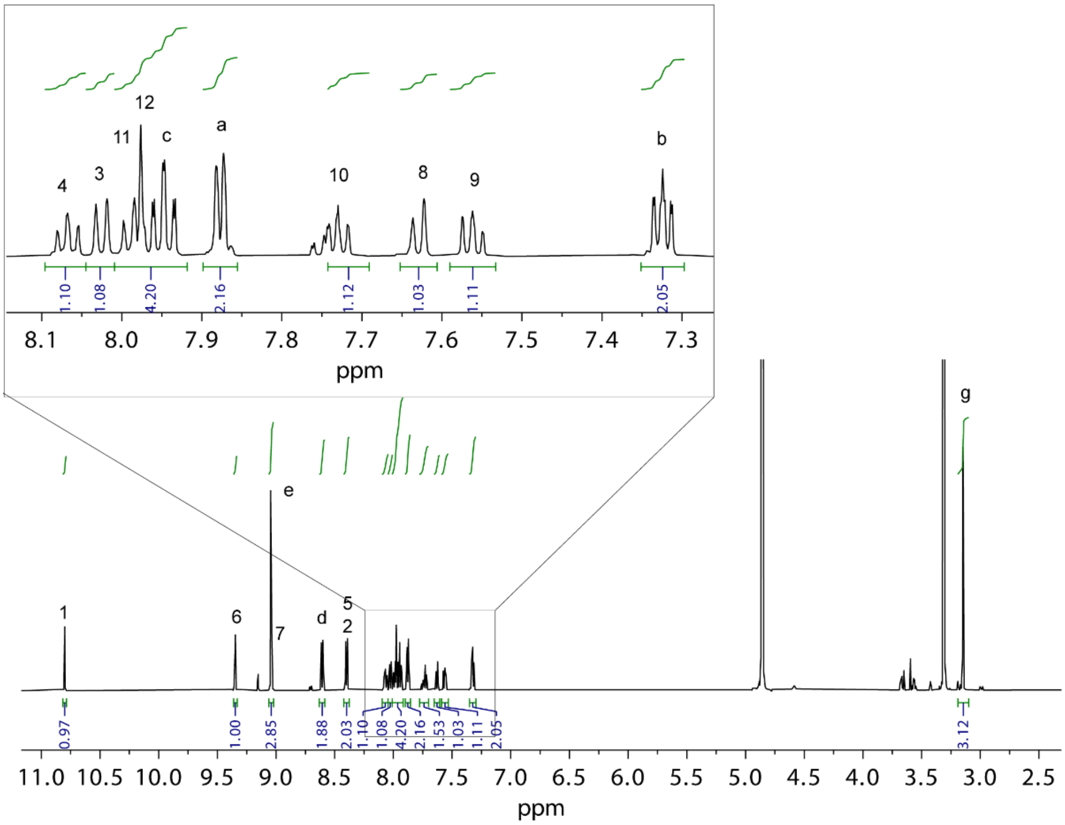

Figure S18 : ^{1}H NMR (850 MHz, MeOD) of [pRu6]Cl with proton assignment.

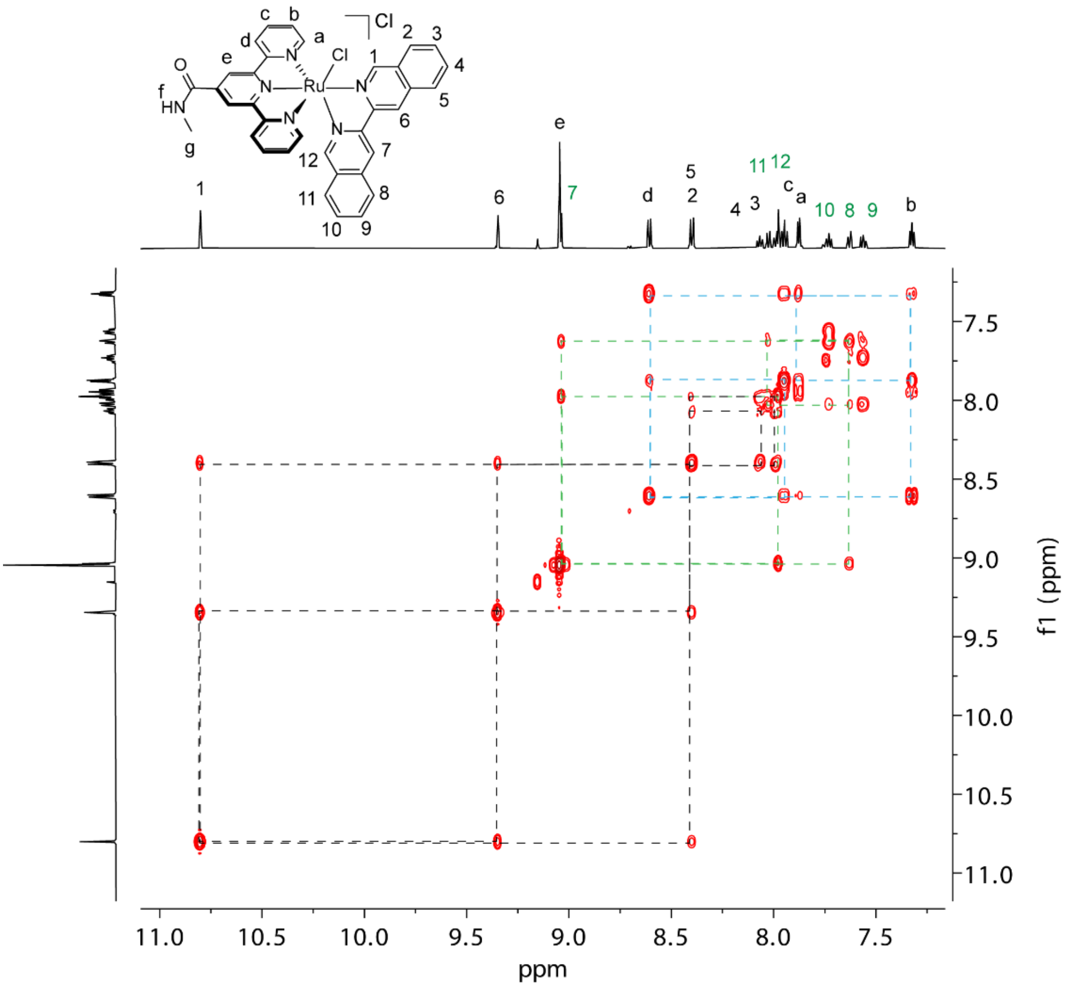

Figure S19 : Enlargement of aromatic region ^{1}H-^{1}H COSY (850 MHz, MeOD) of [pRu6]Cl.

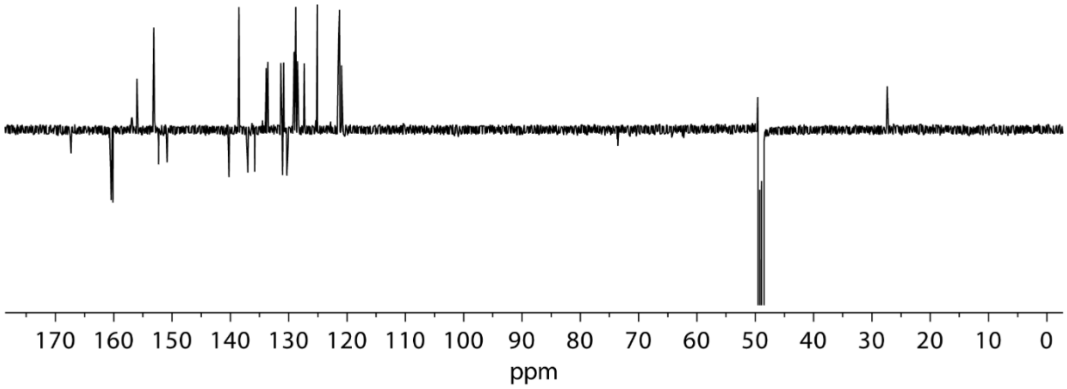

Figure S20 : ^{13}C NMR (214 MHz, MeOD) of [pRu6]Cl.

[Ru(tpa)(i-Hdiqa)Cl]Cl – [pRu7]Cl

$[Ru(tpa)Cl_2]_2$ (50 mg, 0.05 mmol, 1.0 equiv.) and i-Hdiqa ligand (30 mg, 0.1 mmol, 2.0 equiv.) were added to a microwave vial along with ethylene glycol (2 mL) as the solvent. The mixture was degassed by bubbling nitrogen gas through it for 10 min. The reaction mixture was then stirred and heated in the sealed vial at 180 °C for 1.5 h with a conventional block. After completion of the reaction, the solvent was removed under reduced pressure to yield the crude product as a dark red powder. The product was purified using column chromatography on silica gel with a dichloromethane/methanol gradient (0–10% methanol). This yielded the final product as a dark red powder in 58% yield (46 mg, 0.06 mmol). ^{1}H NMR (850 MHz, MeOD) δ 10.37 (s, 1H, H1), 8.98

(s, 2H, He), 8.65 – 8.56 (m, 4H, Hd, Ha), 8.17 (d, J = 8.0 Hz, 1H, H2), 8.12 – 8.01 (m, 3H, H5, Hc), 7.94 – 7.84 (m, 2H, H6, H4), 7.66 (ddd, J = 7.9, 6.7, 1.1 Hz, 1H, H3), 7.64 – 7.56 (m, 3H, Hb, H9), 7.53 (ddd, J = 8.1, 6.6, 1.2 Hz, 1H, H10), 7.45 (s, 1H, H13), 7.41 – 7.32 (m, 1H, H12), 7.30 – 7.21 (m, 2H, H8, H11), 3.13 (s, 3H, Hb). ^{13}C NMR (214 MHz, MeOD) δ 165.87, 159.91, 159.08, 158.55, 153.18, 152.81, 150.88, 149.81, 138.77, 138.36, 137.24, 137.11, 132.39, 132.12, 127.52, 127.15, 126.50, 126.35, 126.07, 125.90, 125.50, 125.41, 124.73, 123.74, 119.89, 106.87, 106.17, 25.89. ESI MS calculated for $[C_{35}H_{27}N_7ORuCl]^+$: 698.1, measured: 698.0.

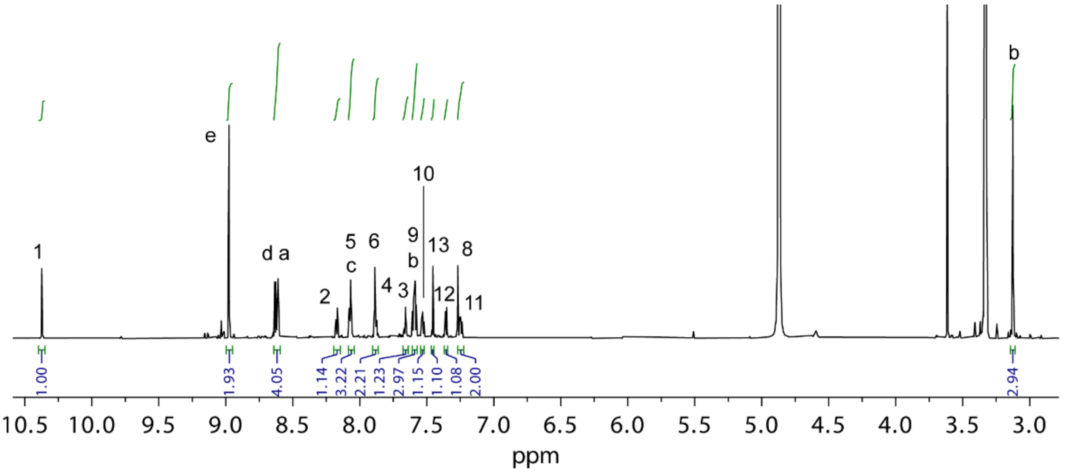

Figure S21 : ^{1}H NMR (850 MHz, MeOD) of [pRu7]Cl with proton assignment.

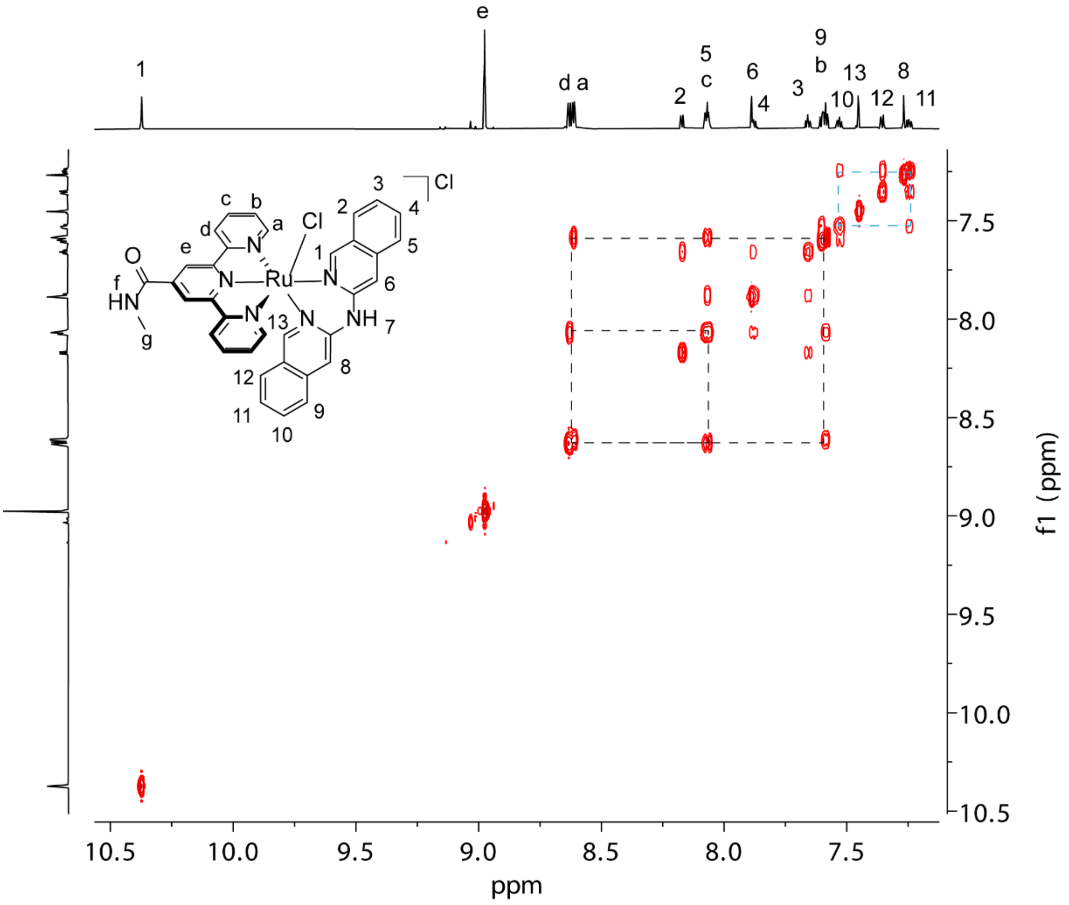

Figure S22 : Enlargement of aromatic region ^{1}H-^{1}H COSY (850 MHz, MeOD) of [pRu7]Cl.

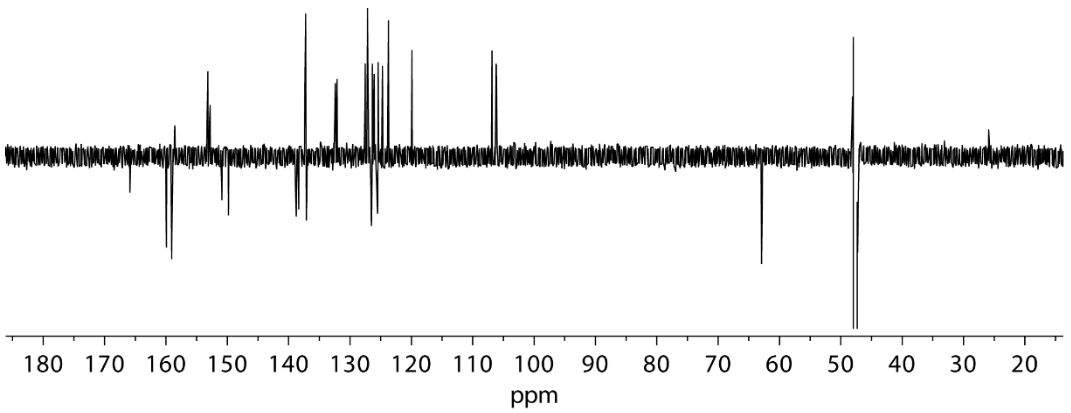

Figure S23 : ^{13}C NMR (214 MHz, MeOD) of [pRu7]Cl.

[Ru(tpa)(Hdpa)Cl]Cl – [pRu8]Cl

$[Ru(tpa)Cl_2]_2$ (50 mg, 0.05 mmol, 1.0 equiv.) and Hdpa ligand (19 mg, 0.1 mmol, 2.0 equiv.) were added to a microwave vial along with ethylene glycol (2 mL) as the solvent. The mixture was degassed by bubbling nitrogen gas through it for 10 min. The reaction mixture was then stirred and heated in the sealed vial at 180 °C for 1.5 h with a conventional block. After completion of the reaction, the solvent was removed under reduced pressure to yield the crude product as a dark red powder. The product was purified using column chromatography on silica gel with a dichloromethane/methanol gradient (0–10% methanol). This yielded the final product as a dark red powder in 80% yield (55 mg, 0.086 mmol). ^{1}H NMR (850 MHz, MeOD) δ 9.56 (ddd, J = 5.8, 1.7, 0.7 Hz, 1H, H1), 8.91 (s, 2H, He), 8.61 – 8.54 (m, 4H, Hd, Ha), 8.11 (ddd, J = 8.6, 7.2, 1.7 Hz, 1H, H3), 8.06 (td, J = 7.7, 1.5 Hz, 2H, Hc), 7.61 (ddd, J = 7.4, 5.5, 1.3 Hz, 2H, Hb), 7.50 (ddd, J = 8.4, 1.4, 0.7 Hz, 1H, H4), 7.43 – 7.38 (m, 2H, H2, H7), 6.87 (ddd, J = 8.3, 1.4, 0.7 Hz, 1H, H6), 6.62 (ddd, J = 6.3, 1.7, 0.6 Hz, 1H, H9), 6.40 (ddd, J = 7.5, 6.3, 1.3 Hz, 1H, H8), 3.09 (s, 3H, Hg). ^{13}C NMR (214 MHz, MeOD) δ 165.71, 159.96, 159.17, 155.49, 153.85, 153.83, 152.82, 148.59, 138.85, 138.14, 137.51, 137.16, 127.16, 123.61, 119.68, 119.14, 118.42, 113.74, 113.21, 25.84. ESI MS calculated for $[C_{27}H_{23}N_7ORuCl]^+$: 598.1, measured: 598.1.

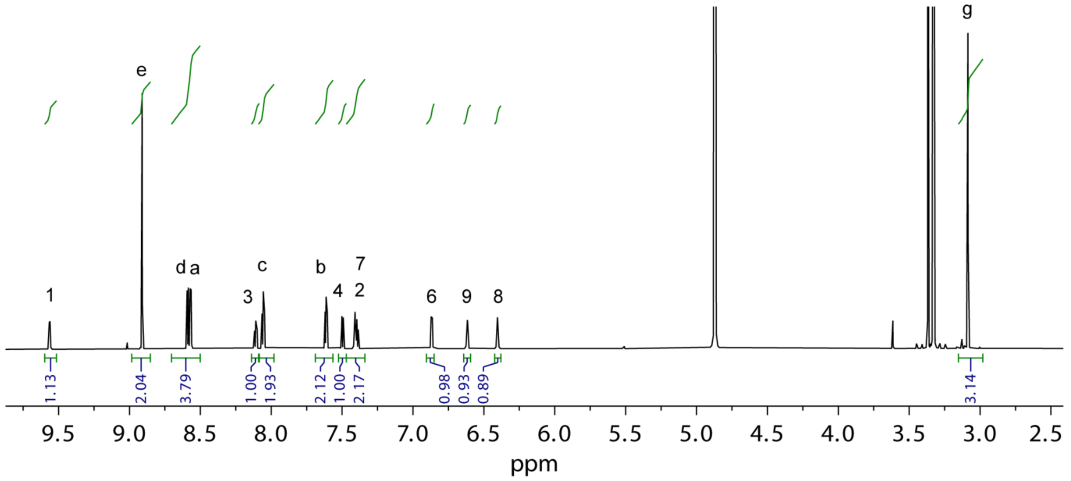

Figure S24 : ^{1}H NMR (850 MHz, MeOD) of [pRu8]Cl with proton assignment.

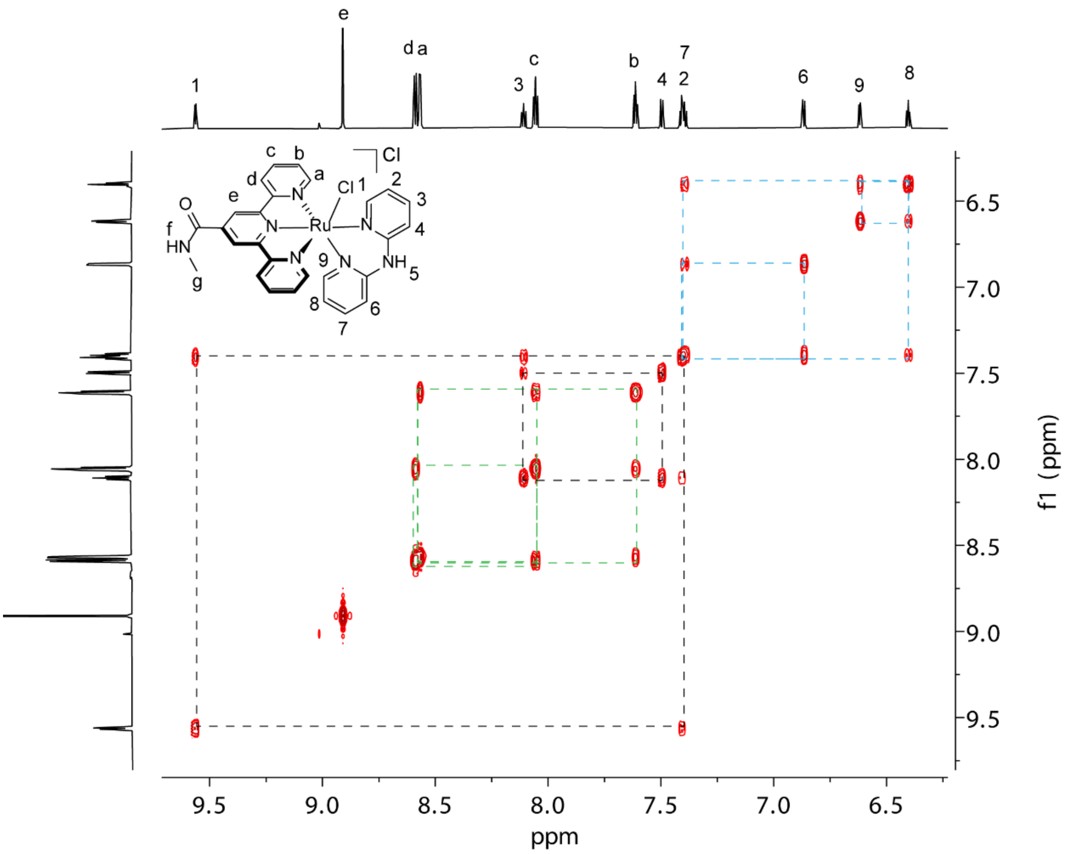

Figure S25 : Enlargement of aromatic region ^{1}H-^{1}H COSY (850 MHz, MeOD) of [pRu8]Cl.

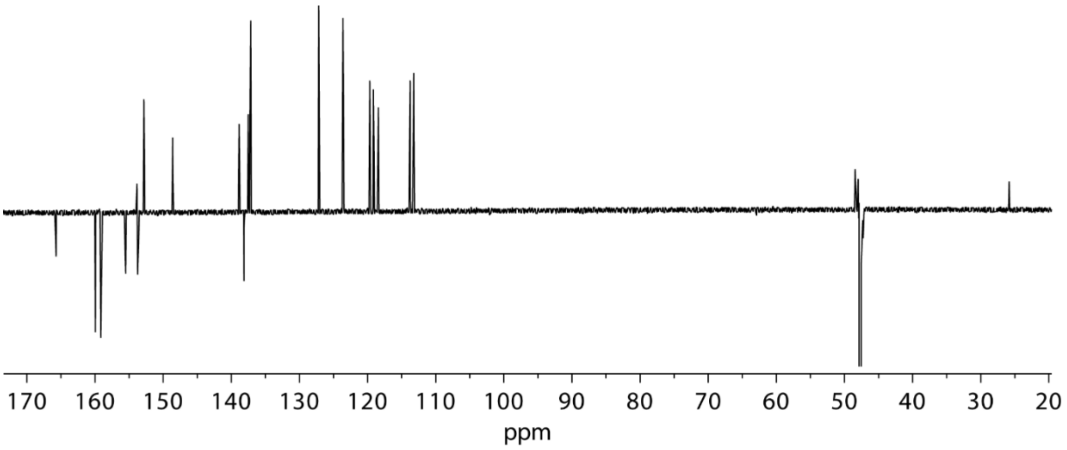

Figure S26 : ^{13}C NMR (214 MHz, MeOD) of [pRu8]Cl.

[Ru(tpa)(Medpa)Cl]Cl – [pRu9]Cl

$[Ru(tpa)Cl_2]_2$ (50 mg, 0.05 mmol, 1.0 equiv.) and Medpa ligand (20 mg, 0.1 mmol, 2.0 equiv.) were added to a microwave vial along with ethylene glycol (2 mL) as the solvent. The mixture was degassed by bubbling nitrogen gas through it for 10 min. The reaction mixture was then stirred and heated in the sealed vial at 180 °C for 1.5 h with a conventional block. After completion of the reaction, the solvent was removed under reduced pressure to yield the crude product as a dark red powder. The product was purified using column chromatography on silica gel with a dichloromethane/methanol gradient (0–10% methanol). This yielded the final product as a dark red powder in 84% yield (59 mg, 0.09 mmol). ^{1}H NMR (850 MHz, MeOD) δ 9.63 (dd, J = 5.6, 1.8 Hz, 1H), 8.95 (s, 2H), 8.61 (d, J = 8.0 Hz, 2H), 8.38 (d, J = 5.6 Hz, 2H), 8.27 (ddd, J = 8.8, 7.3, 1.8 Hz, 1H),

8.06 (t, J = 7.7 Hz, 2H), 7.77 (d, J = 8.5 Hz, 1H), 7.61 (ddd, J = 7.1, 5.5, 1.3 Hz, 3H), 7.57 (ddd, J = 8.7, 7.2, 1.8 Hz, 1H), 7.17 (dd, J = 8.4, 1.2 Hz, 1H), 6.71 (dd, J = 6.2, 1.8 Hz, 1H), 6.56 (ddd, J = 7.3, 6.1, 1.2 Hz, 1H), 3.60 (s, 3H), 3.10 (s, 3H). ^{13}C NMR (214 MHz, MeOD) δ 165.72, 159.60, 159.52, 159.08, 157.78, 154.42, 154.40, 148.95, 139.25, 138.13, 138.03, 137.20, 137.12, 126.93, 123.61, 120.67, 119.83, 115.35, 114.69, 39.62, 25.85. ESI MS calculated for $[C_{28}H_{25}N_7ORuCl]^+$: 612.1, measured: 612.2.

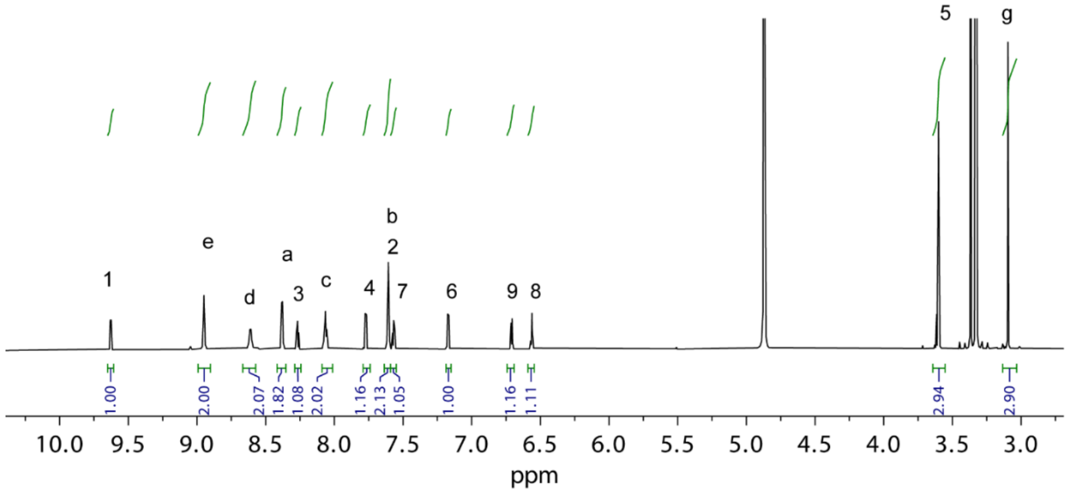

Figure S27 : ^{1}H NMR (850 MHz, MeOD) of [pRu9]Cl with proton assignment.

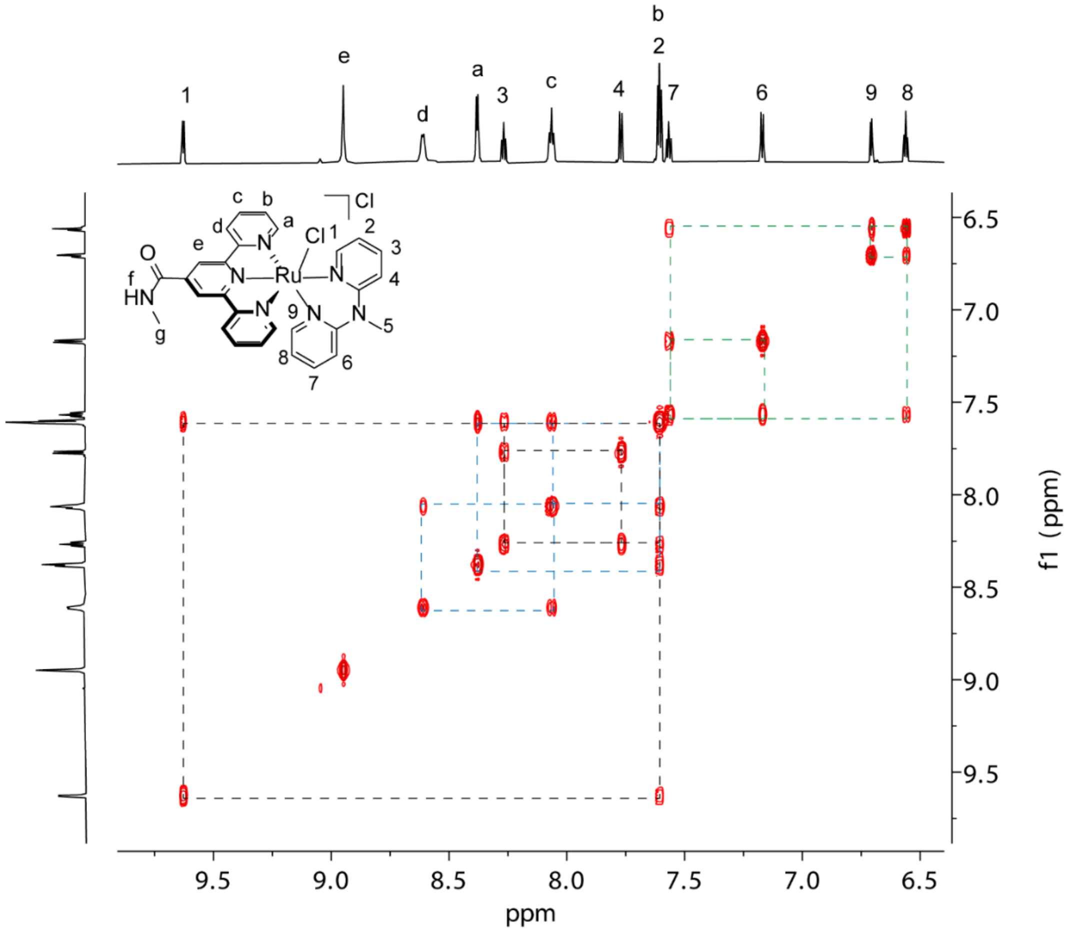

Figure S28 : Enlargement of aromatic region ^{1}H-^{1}H COSY (850 MHz, MeOD) of [pRu9]Cl.

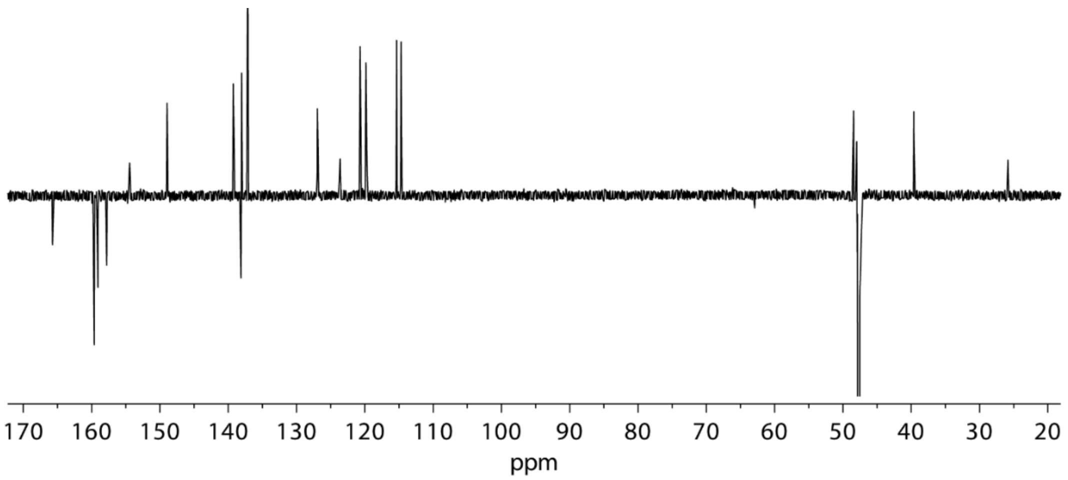

Figure S29 : ^{13}C NMR (214 MHz, MeOD) of [pRu9]Cl.

[Ru(dpn)(dppz)(NAcMet)]Cl_2 – [Ru1]Cl_2

[pRu1]Cl (20 mg, 0.026 mmol, 1.0 equiv.) and N-Acetly-(L)-Methionine (25 mg, 0.132 mmol, 5.0 equiv.) were suspended in 4 mL of H_2O in a microwave vial. The mixture was degassed by bubbling nitrogen gas through it for 10 min. The reaction mixture was then stirred and heated in the sealed vial at 100 °C for 16 h with a conventional block. After cooling down to room temperature, the product was precipitated as [Ru1]$(PF_6)_2$ salt by addition of saturated KPF_6 water (1 mL). The solid was isolated by centrifugation and subsequentially purified by flash column chromatography on silica gel and "Magic Mixture" (acetone:H_2O:sat$KPF_{6aq.}$ 8:1:1) as eluent. The hexafluorophosphate counterions were exchanged with chloride anions through an anion exchange column. The purity of the compounds was confirmed by analytical HPLC (Figure S59). The final product was obtained as a bright red solid in 71 % yield (17 mg, 0.019 mmol). ^1H NMR (400 MHz, MeOD) δ 10.66 (dd, J = 5.5, 1.3 Hz, 1H, H1), 10.12 (dd, J = 8.2, 1.3 Hz, 1H, H3), 9.42 (dd, J = 8.2, 1.3 Hz, 1H, H8), 9.25 (d, J = 8.2 Hz, 2H, Hf), 8.91 (dd, J = 8.6, 2.4 Hz, 2H, He), 8.74 (dd, J = 8.6, 2.0 Hz, 2H, Hd), 8.71 – 8.62 (m, 2H, H2, Hg), 8.56 (dd, J = 8.7, 1.4 Hz, 1H, H7), 8.37 (dt, J = 8.2, 2.1 Hz, 3H, H4, Hc), 8.20 (dt, J = 4.4, 1.8 Hz, 2H, Ha), 8.10 (m, 2H, H5, H6), 7.75 (dd, J = 5.5, 1.3 Hz, 1H, H10), 7.57 (dd, J = 8.2, 5.5 Hz, 1H, H9), 7.47 (ddd, J = 8.2, 4.2, 1.2 Hz, 2H, Hb), 3.90 (dd, J = 7.8, 4.2 Hz, 1H, HD), 1.76 (s, 3H, HE), 1.75 – 1.35 (m, 4H, HC, HB), 1.32 (s, 3H, HA). ^{13}C NMR (151 MHz, MeOD) δ 172.29, 162.83, 160.45, 158.21, 155.45, 155.43, 152.97, 152.44, 151.45, 143.90, 143.61, 142.18, 140.05, 139.04, 137.46, 134.48, 133.59, 132.92, 132.68, 130.57, 130.38, 130.33, 130.22, 127.85, 127.09, 126.62, 126.12, 126.11, 125.59, 121.91, 52.77, 32.30, 30.85, 22.30, 14.64. Elemental Analysis Calculated for $C_{36}H_{33}N_7F_6O_4RuSCl_2$: C, 56.33; H, 3.70; N, 14.28. Found: C, 55.57; H, 3.75; N, 14.21. HR ESI MS calculated for $[C_{36}H_{33}N_7F_6O_4RuS]^{2+}$: 455.1, measured: 455.086.

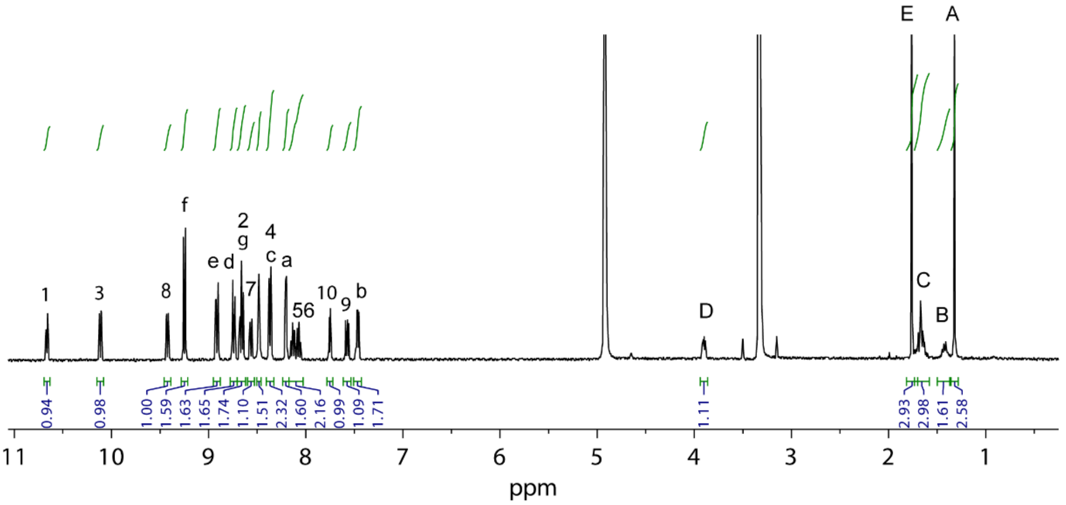

Figure S30 : ^{1}H NMR (400 MHz, MeOD) of $[Ru1]Cl_2$ with proton assignment.

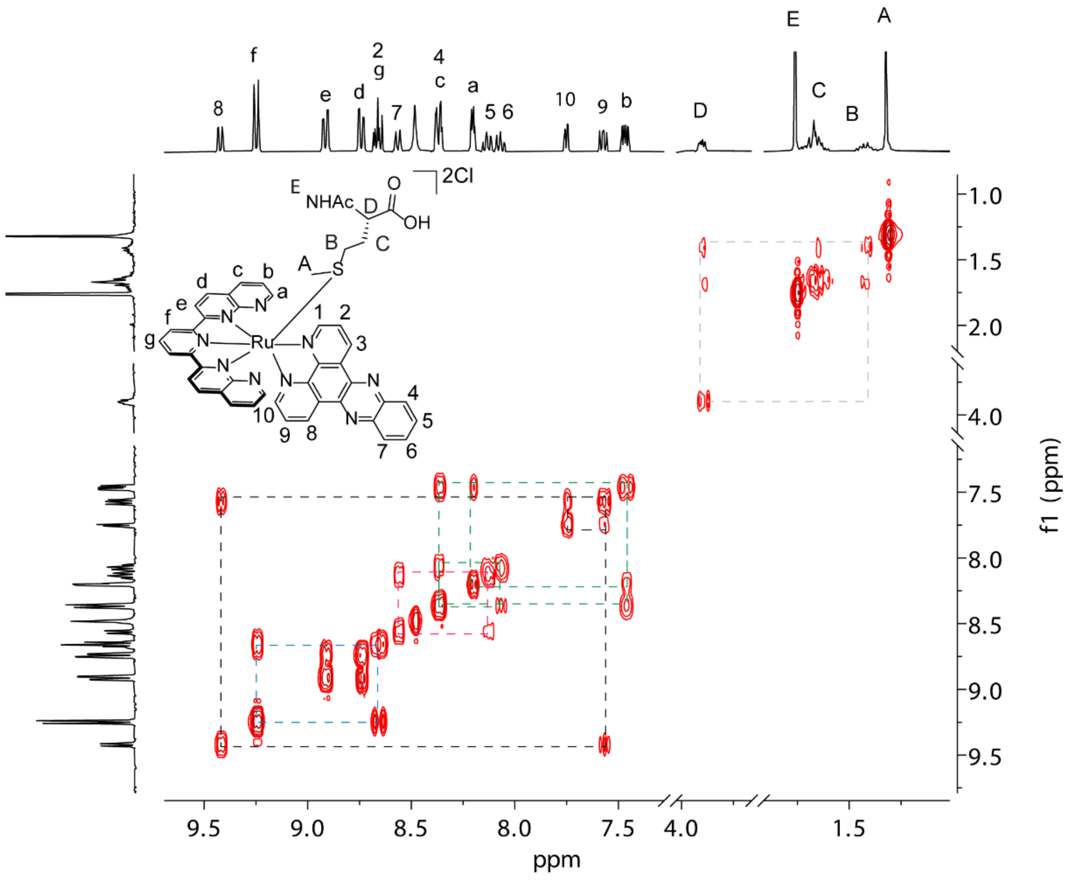

Figure S31 : Enlargement of ^{1}H-^{1}H COSY (400 MHz, MeOD) region of interest of $[Ru1]Cl_2$.

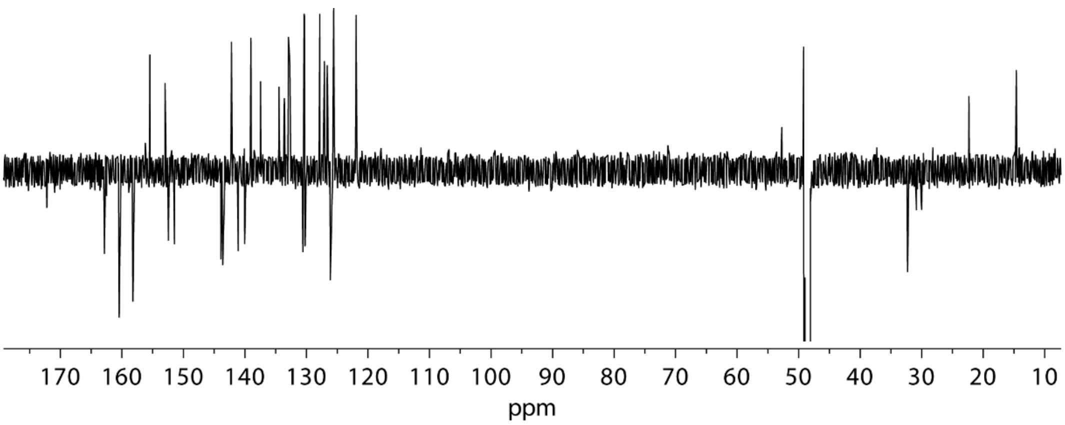

Figure S32 : ^{13}C NMR (214 MHz, MeOD) of [Ru1]Cl₂.

[Ru(tpa)(*N-N*)(NAcMet)]Cl_2 – [Ru2]Cl_2 – [Ru9]Cl_2

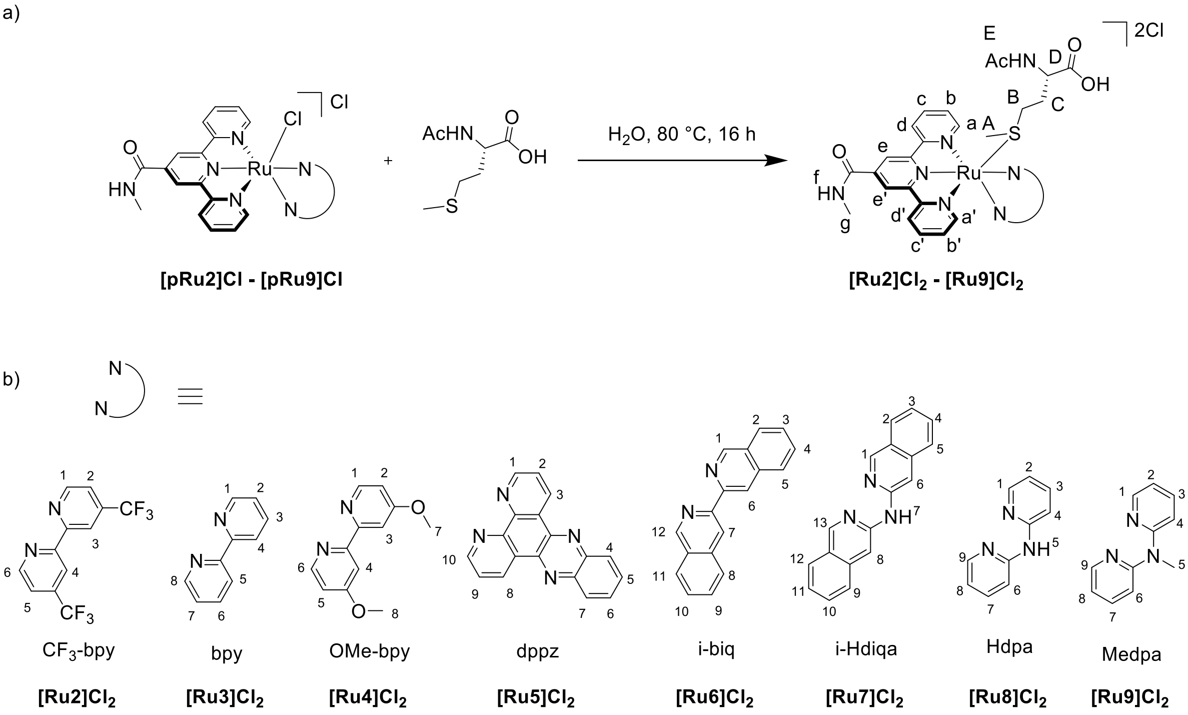

[Ru(tpa)(CF_3-bpy)(NAcMet)]Cl_2 – [Ru2]Cl_2

[pRu2]Cl (20 mg, 0.026 mmol, 1.0 equiv.) and N-Acetly-(L)-Methionine (40 mg, 0.212 mmol, 8.0 equiv.) were suspended in 4 mL of H_2O in a microwave vial. The mixture was degassed by bubbling nitrogen gas through it for 10 min. The reaction mixture was then stirred and heated in the sealed vial at 100 °C for 16 h with a conventional block. After cooling down to room temperature, the product was precipitated as [Ru2]$(PF_6)_2$ salt by addition of saturated KPF_6 water (1 mL). The solid was isolated by centrifugation and subsequentially purified by flash column chromatography on silica gel and "Magic Mixture" (acetone:H_2O:sat$KPF_{6aq.}$ 8:1:1) as eluent. The hexafluorophosphate counterions were exchanged with chloride anions through an anion exchange column. The purity of the compounds was confirmed by analytical HPLC (Figure S59). The final product was obtained as a bright red solid in 76 % yield (19 mg, 0.02 mmol). ^{1}H NMR (400 MHz, MeOD) δ 10.22 (d, J = 5.9 Hz, 1H, H1), 9.49 – 9.44 (m, 1H, H3), 9.26 (t, J = 1.3 Hz, 1H, H4), 9.17 (s, 2H, H3), 8.73 (dd, J = 8.1, 3.1 Hz, 2H, Hd), 8.46 (dd, J = 6.1, 1.9 Hz, 1H, H2), 8.18 (tt, J = 7.9, 1.5 Hz, 2H, Hc), 7.85 (dd, J = 5.5, 1.4 Hz, 2H, Ha), 7.64 (d, J = 5.9 Hz, 1H, H5), 7.55 (dd, J = 6.1, 1.9 Hz, 1H, H6), 7.50 (ddd, J = 7.0, 5.6, 1.2 Hz, 2H, Hb), 4.10 (t, J = 6.1 Hz, 1H, HD), 3.17 (s, 3H, Hg), 1.88 (s, 3H, He), 1.78 – 1.51 (m,

4H, HB, HC), 1.38 (s, 3H, HA). ^{13}C NMR (214 MHz, MeOD, from HSQC) δ 154.87, 154.77, 152.62, 140.71, 130.07, 126.75, 125.19, 123.33, 123.23, 123.04, 122.26, 51.66, 31.26 (2C), 29.89 (2), 27.25, 22.47, 13.88. Elemental Analysis Calculated for $C_{36}H_{33}N_7F_6O_4RuSCl_2$: C, 45.72; H, 3.52; N, 10.37. Found: C, 44.34; H, 3.81; N, 9.96. ESI MS calculated for $[C_{36}H_{33}N_7F_6O_4RuS]^{2+}$: 437.6, measured: 437.5.

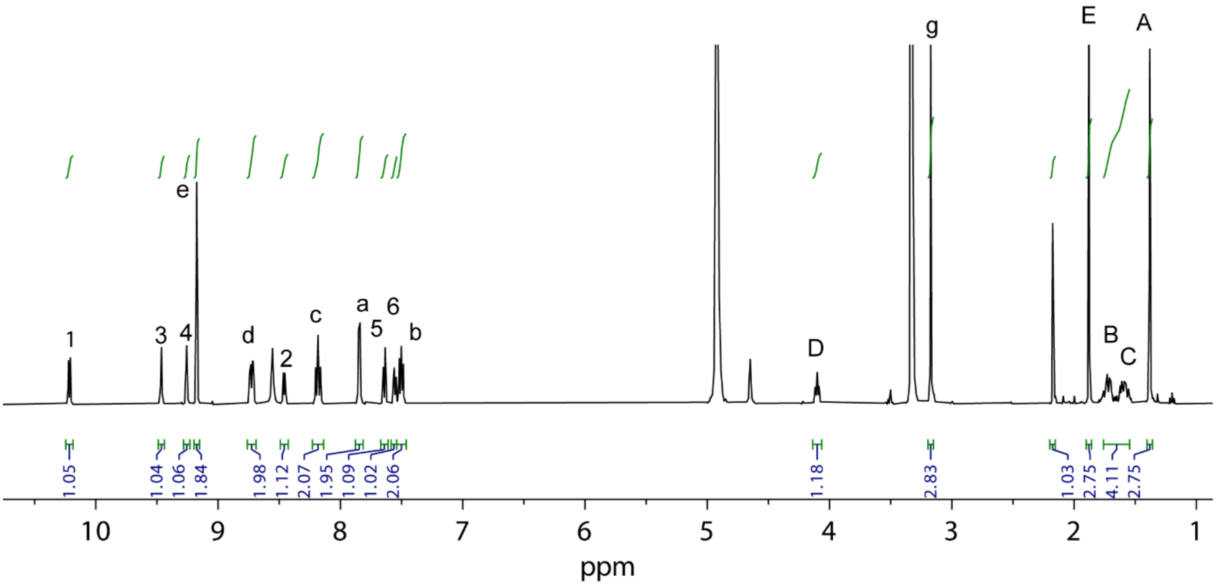

Figure S33 : ^1H NMR (400 MHz, MeOD) of [Ru2]Cl_2 with proton assignment.

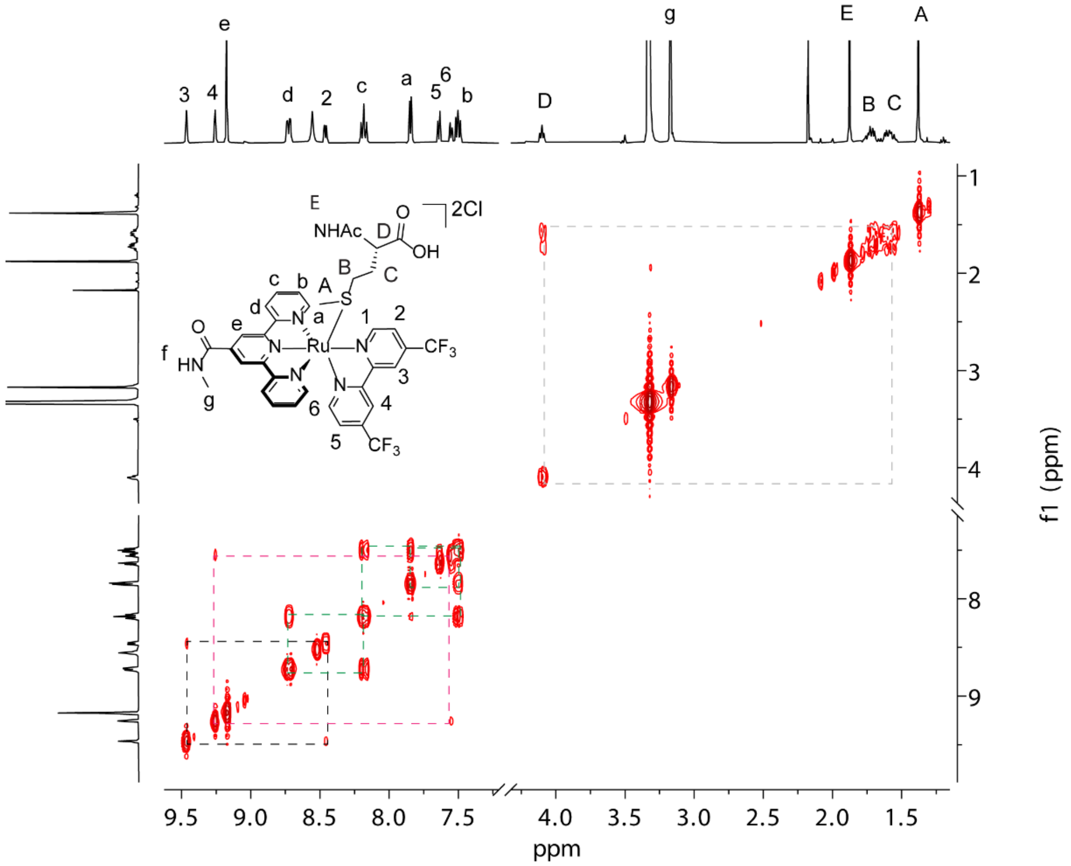

Figure S34 : Enlargement of ^1H-^1H COSY (400 MHz, MeOD) region of interest of [Ru2]Cl_2.

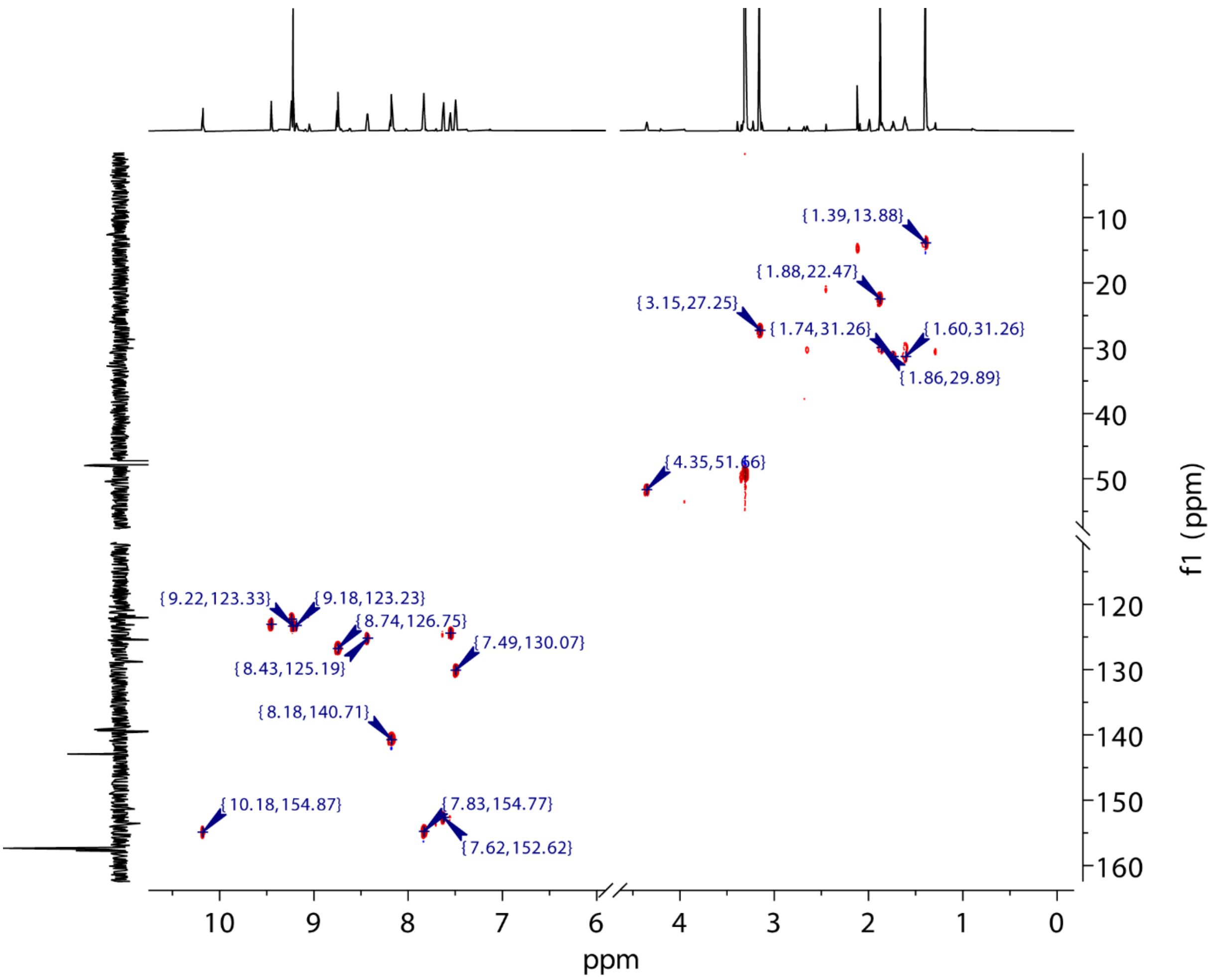

Figure S35 : ^{1}H-^{13}C HSQC NMR (214 MHz, MeOD) of [Ru2]Cl_2.

[Ru(tpa)(bpy)(NacMet)]Cl_2 – [Ru3]Cl_2

[pRu3]Cl (50 mg, 0.081 mmol, 1.0 equiv.) and N-Acetly-(L)-Methionine (124 mg, 0.647 mmol, 8.0 equiv.) were suspended in 4 mL of H_2O in a microwave vial. The mixture was degassed by bubbling nitrogen gas through it for 10 min. The reaction mixture was then stirred and heated in the sealed vial at 100 °C for 16 h with a conventional block. After cooling down to room temperature, the product was precipitated as [Ru3]$(PF_6)_2$ salt by addition of saturated KPF_6 water (1 mL). The solid was isolated by centrifugation and subsequentially purified by flash column chromatography on silica gel and “Magic Mixture” (acetone:H_2O:sat$KPF_{6aq.}$ 8:1:1) as eluent. The hexafluorophosphate counterions were exchanged with chloride anions through an anion exchange column. The purity of the compounds was confirmed by analytical HPLC (Figure S59). The final product was obtained as a bright red solid in 66 % yield (43 mg, 0.053 mmol). ^{1}H NMR (850 MHz, MeOD) δ 9.89 (dt, J = 5.6, 1.2 Hz, 1H, H1), 9.22 (d, J = 1.7 Hz, 2H, He), 8.90 – 8.86 (m, 1H, H4), 8.75 (t, J = 8.0 Hz, 2H, Hd), 8.63 (d, J = 8.1 Hz, 1H, H5), 8.45 (td, J = 8.0, 1.4 Hz, 1H, H3), 8.17 (m, 2H, Hc), 8.12 (ddd, J = 7.7, 5.5, 1.4 Hz, 1H, H2), 7.96 (td, J = 7.7, 1.5 Hz, 1H, H6), 7.84 – 7.81 (m, 2H, Ha), 7.51 (m, 2H, Hb), 7.30 (dd, J = 5.7, 1.5 Hz, 1H, H8), 7.24 (ddd, J = 7.3, 5.8, 1.3 Hz, 1H, H7), 4.36 (dd, J = 9.0, 4.9 Hz, 1H, HD), 3.17 (s, 3H, Hg), 1.90 (s, 3H, HE), 1.79 –1.59 (m, 4H, HB, HC), 1.41 (s, 3H, HA). ^{13}C NMR (214 MHz, MeOD) δ 173.66, 173.10, 166.23, 159.23, 159.23, 159.05, 158.03, 157.83, 154.36, 153.37, 150.80, 143.41, 140.44, 139.81, 139.77, 139.65, 130.15, 130.11, 129.17, 128.53, 126.63, 126.01, 125.26, 125.22, 123.26, 123.22, 123.17, 51.47, 31.37, 29.84, 27.37, 22.39, 14.13. Elemental Analysis Calculated for $C_{34}H_{35}N_7O_4RuSCl_2$: C, 55.27; H, 4.78; N, 13.27. Found: C, 53.98; H, 5.11; N, 12.82. ESI MS calculated for $[C_{34}H_{35}N_7O_4RuS]^{2+}$: 369.6, measured: 369.6

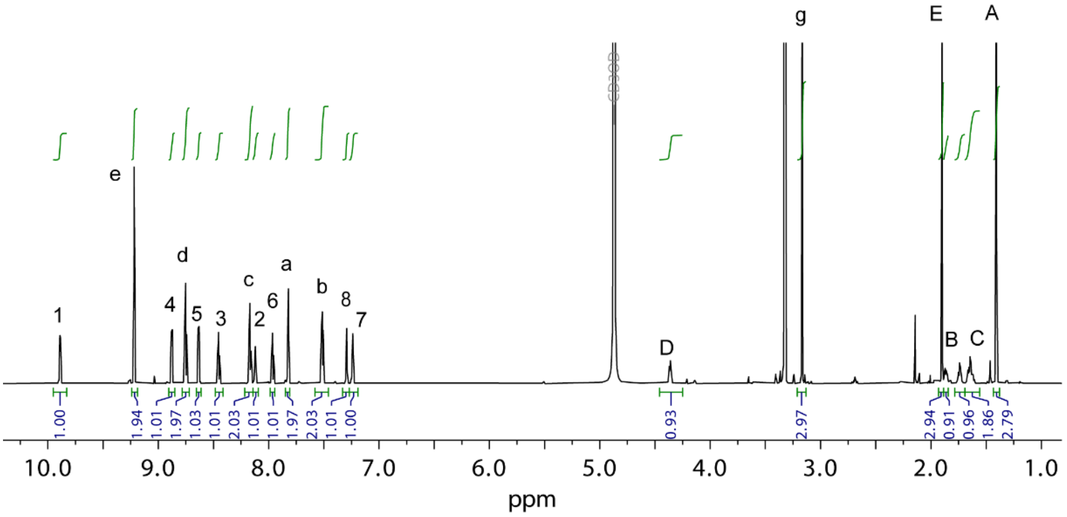

Figure S36 : ^{1}H NMR (850 MHz, MeOD) of $[Ru3]Cl_2$ with proton assignment.

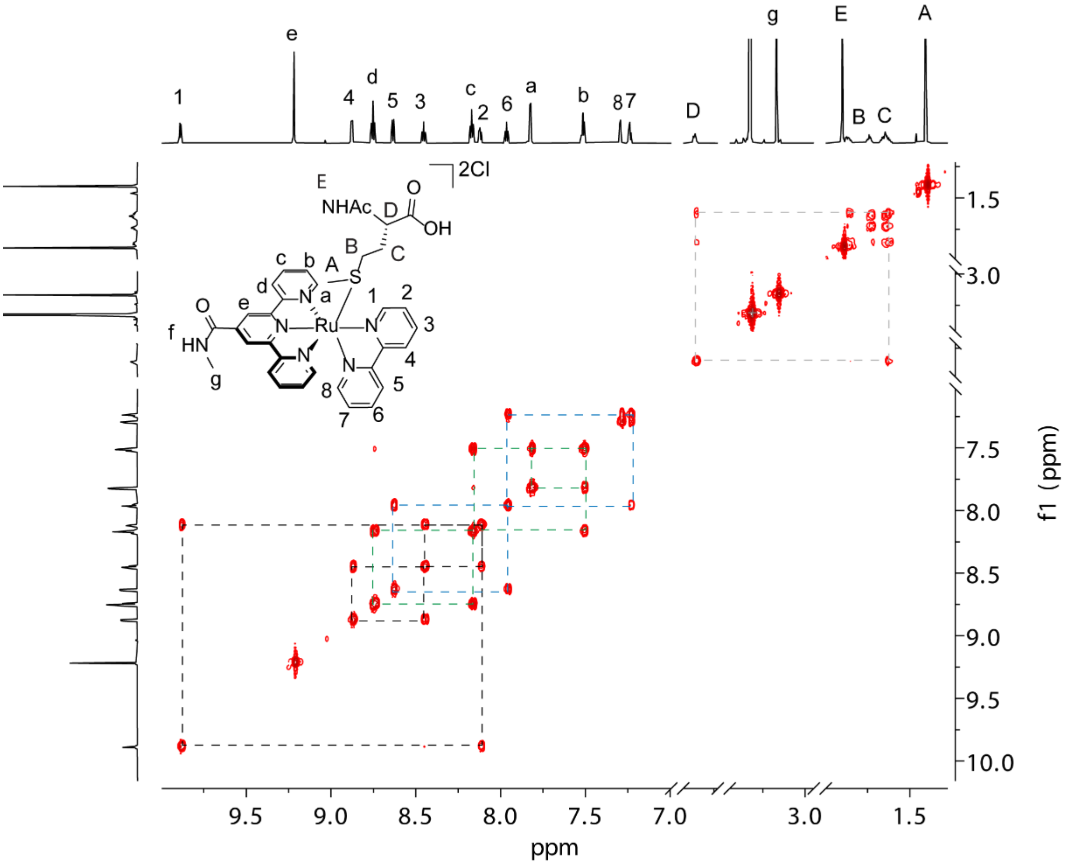

Figure S37 : Enlargement of ^{1}H-^{1}H COSY (850 MHz, MeOD) region of interest of $[Ru3]Cl_2$.

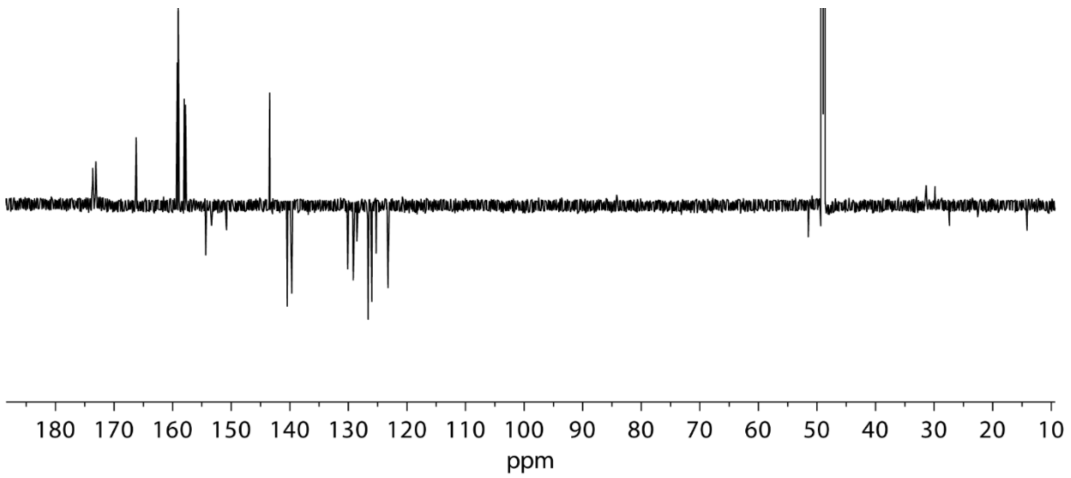

Figure S38 : ^{13}C NMR (214 MHz, MeOD) of $[Ru3]Cl_2$.

[Ru(tpa)(OMe-bpy)(NAcMet)]Cl_2 – [Ru4]Cl_2

[pRu4]Cl (50 mg, 0.074 mmol, 1.0 equiv.) and N-Acetly-(L)-Methionine (112 mg, 0.589 mmol, 8.0 equiv.) were suspended in 4 mL of H_2O in a microwave vial. The mixture was degassed by bubbling nitrogen gas through it for 10 min. The reaction mixture was then stirred and heated in the sealed vial at 100 °C for 16 h with a conventional block. After cooling down to room temperature, the product was precipitated as $[Ru4](PF_6)_2$ salt by addition of saturated KPF_6 water (1 mL). The solid was isolated by centrifugation and subsequentially purified by flash column chromatography on silica gel and "Magic Mixture" (acetone:H_2O:sat$KPF_{6aq.}$ 8:1:1) as eluent. The hexafluorophosphate counterions were exchanged with chloride anions through an anion exchange column. The purity of the compounds was confirmed by analytical HPLC (Figure S60). The final product was obtained as a bright red solid in 75 % yield (48 mg, 0.055 mmol). ^1H NMR (400 MHz, MeOD) δ 9.64 (d, J = 6.6 Hz, 1H, H1), 9.10 (s, 2H, He), 8.68 (dd, J = 8.1, 3.4 Hz, 2H, Hd), 8.43 (d, J = 2.7 Hz, 1H, H4), 8.21 – 8.10 (m, 3H, H5, Hc), 7.88 (dd, J = 5.7, 1.4 Hz, 2H, Ha), 7.72 (dd, J = 6.6, 2.7 Hz, 1H, H2), 7.52 (ddd, J = 7.6, 5.5, 1.3 Hz, 2H, Hb), 6.97 (d, J = 6.5 Hz, 1H, H8), 6.77 (dd, J = 6.7, 2.7 Hz, 1H, H7), 4.25 (s, 3H, H3), 4.05 (dd, J = 7.2, 5.3 Hz, 1H, HD), 3.91 (s, 3H, H6), 3.16 (s, 3H, Hg), 1.88 (s, 3H, HE), 1.78 – 1.45 (m, 4H, HB, HC), 1.34 (s, 3H, HA). ^{13}C NMR (214 MHz, MeOD, from HSQC) δ 154.09, 153.79, 151.06, 140.03, 129.97, 126.26, 122.84, 115.03, 114.45, 112.59, 111.91, 57.13, 57.03, 51.76, 31.26 (2C), 29.89 (2C), 27.25, 22.37, 13.88. Elemental Analysis Calculated for $C_{36}H_{39}N_7O_6RuSCl_2$: C, 49.71; H, 4.52; N, 11.27. Found: C, 48.96; H, 4.80; N, 10.99. ESI MS calculated for $[C_{36}H_{39}N_7O_6RuS]^{2+}$: 399.6, measured: 399.6.

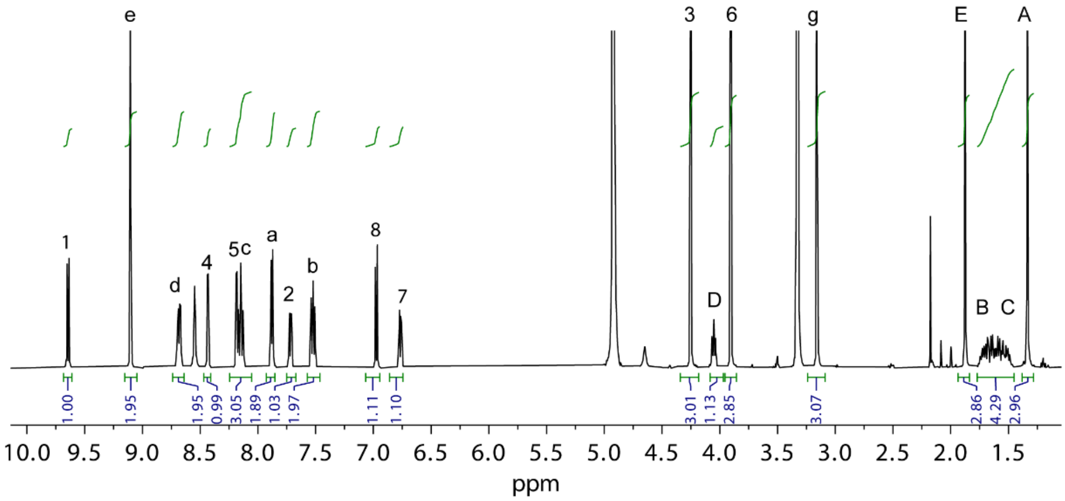

Figure S39 : ^{1}H NMR (400 MHz, MeOD) of $[Ru4]Cl_2$ with proton assignment.

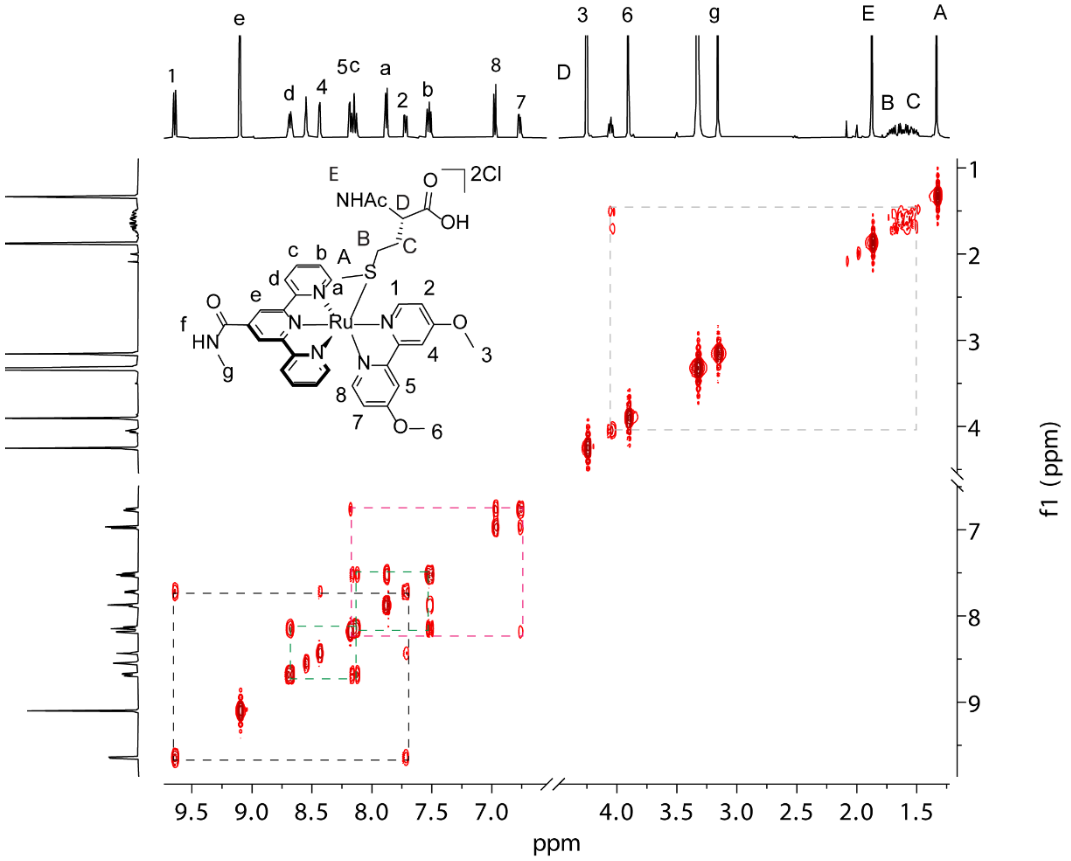

Figure S40 : Enlargement of ^{1}H-^{1}H COSY (400 MHz, MeOD) region of interest of $[Ru4]Cl_2$.

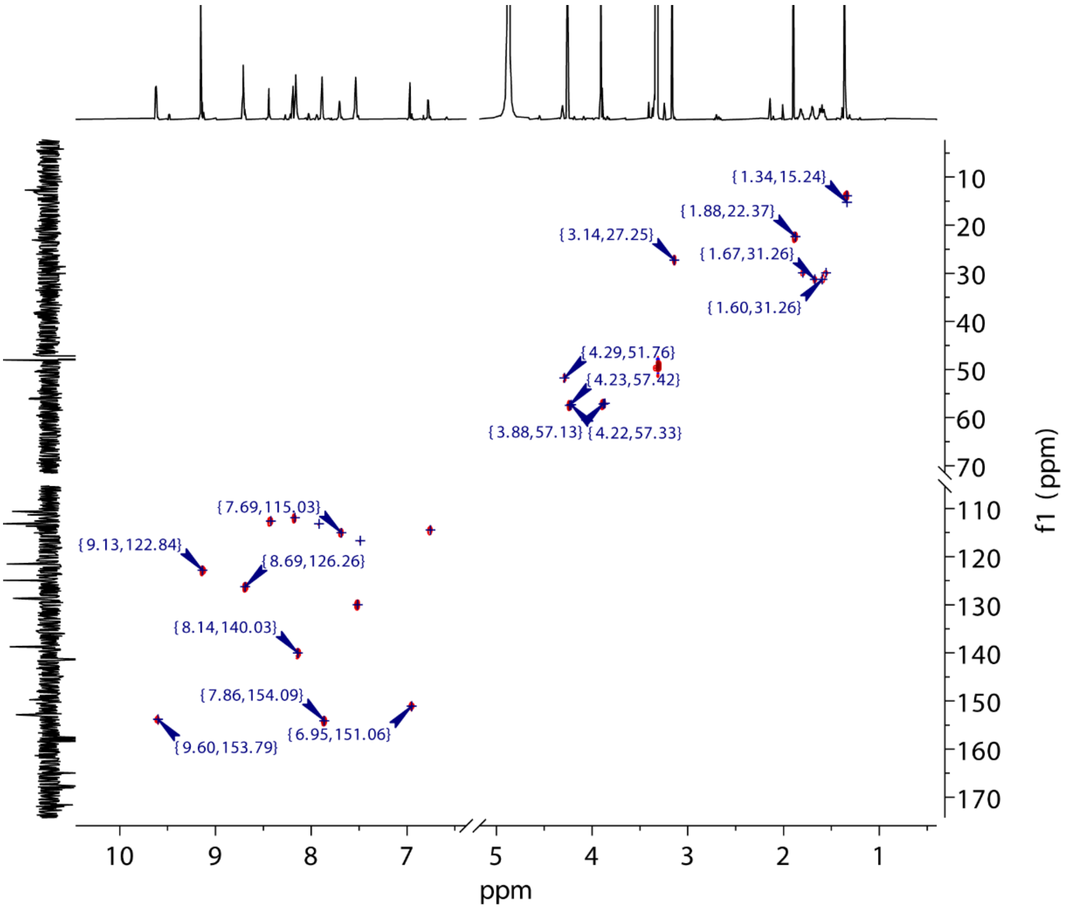

Figure S41 : ^{1}H-^{13}C HSQC NMR (214 MHz, MeOD) of $[Ru4]Cl_2$.

[Ru(tpa)(dppz)(NAcMet)]Cl_2 – [Ru5]Cl_2

[pRu5]Cl (50 mg, 0.067 mmol, 1.0 equiv.) and N-Acetly-(L)-Methionine (102 mg, 0.537 mmol, 8.0 equiv.) were suspended in 4 mL of H_2O in a microwave vial. The mixture was degassed by bubbling nitrogen gas through it for 10 min. The reaction mixture was then stirred and heated in the sealed vial at 100 °C for 16 h with a conventional block. After cooling down to room temperature, the product was precipitated as $[Ru5](PF_6)_2$ salt by addition of saturated KPF_6 water (1 mL). The solid was isolated by centrifugation and subsequentially purified by flash column chromatography on silica gel and “Magic Mixture” (acetone:H_2O:sat$KPF_{6aq.}$ 8:1:1) as eluent. The hexafluorophosphate counterions were exchanged with chloride anions through an anion exchange column. The purity of the compounds was confirmed by analytical HPLC (Figure S60). The final product was obtained as a bright red solid in 62 % yield (39 mg, 0.042 mmol). ^{1}H NMR (850 MHz, MeOD) δ 10.33 (d, J = 5.1 Hz, 1H, H1), 10.13 (d, J = 8.2 Hz, 1H, H3), 9.65 (d, J = 7.9 Hz, 1H, H10), 9.22 (s, 2H, He), 8.76 (t, J = 7.6 Hz, 2H, Hd), 8.71 – 8.53 (m, 2H, H2, H4), 8.48 (d, J = 8.2 Hz, 1H, H7), 8.24 – 8.05 (m, 4H, H5, H6, Hc), 7.93 – 7.82 (m, 2H, Ha), 7.79 (d, J = 5.5 Hz, 1H, H8), 7.73 (dd, J = 8.0, 5.5 Hz, 1H, H9), 7.40 (t, J = 6.6 Hz, 2H, Hb), 4.15 (dd, J = 7.5, 4.9 Hz, 1H, HD), 3.20 (s, 3H, Hg), 1.90 (s, 3H, HE), 1.85 – 1.58 (m, 4H, HB, HD), 1.48 (s, 3H, HA). ^{13}C NMR (214 MHz, MeOD) δ 175.22, 171.08, 165.16, 157.98, 157.66, 157.64, 153.39, 151.42, 149.97, 149.89, 143.01, 142.88, 142.36, 140.00, 139.55, 138.99, 134.26, 134.14, 132.18, 132.11, 131.35, 130.50, 129.52, 129.44, 128.61, 127.47, 126.60, 125.20, 125.18, 121.86, 121.84, 121.80, 52.91, 52.84, 30.42, 29.77, 13.03. Elemental Analysis Calculated for $C_{42}H_{37}N_9O_4RuSCl_2$: C, 53.90; H, 3.99; N, 13.47. Found: C, 53.35; H, 4.47; N, 12.98. ESI MS calculated for $[C_{42}H_{37}N_9O_4RuS]^{2+}$: 432.6, measured: 432.5.

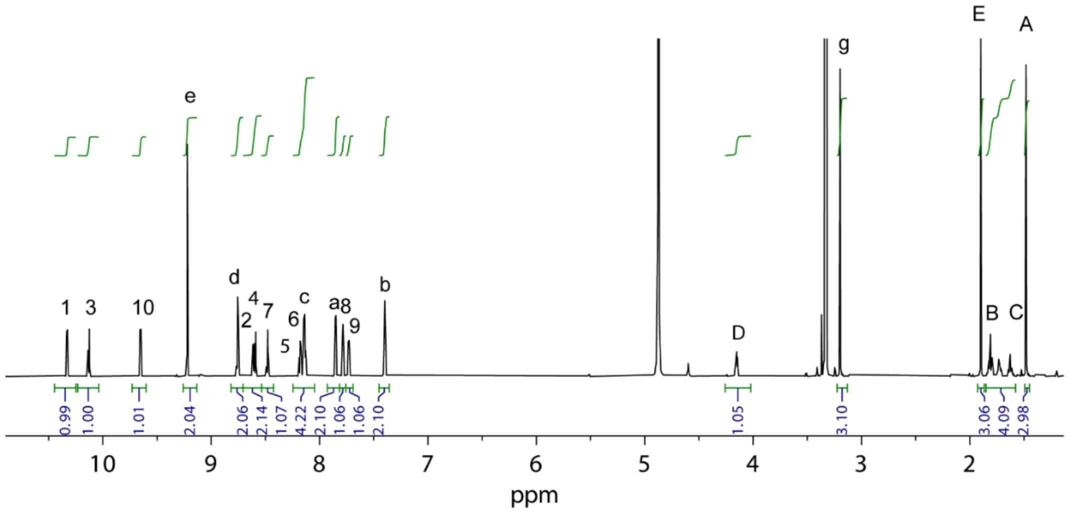

Figure S42 : ^1H NMR (850 MHz, MeOD) of $[Ru5]Cl_2$ with proton assignment.

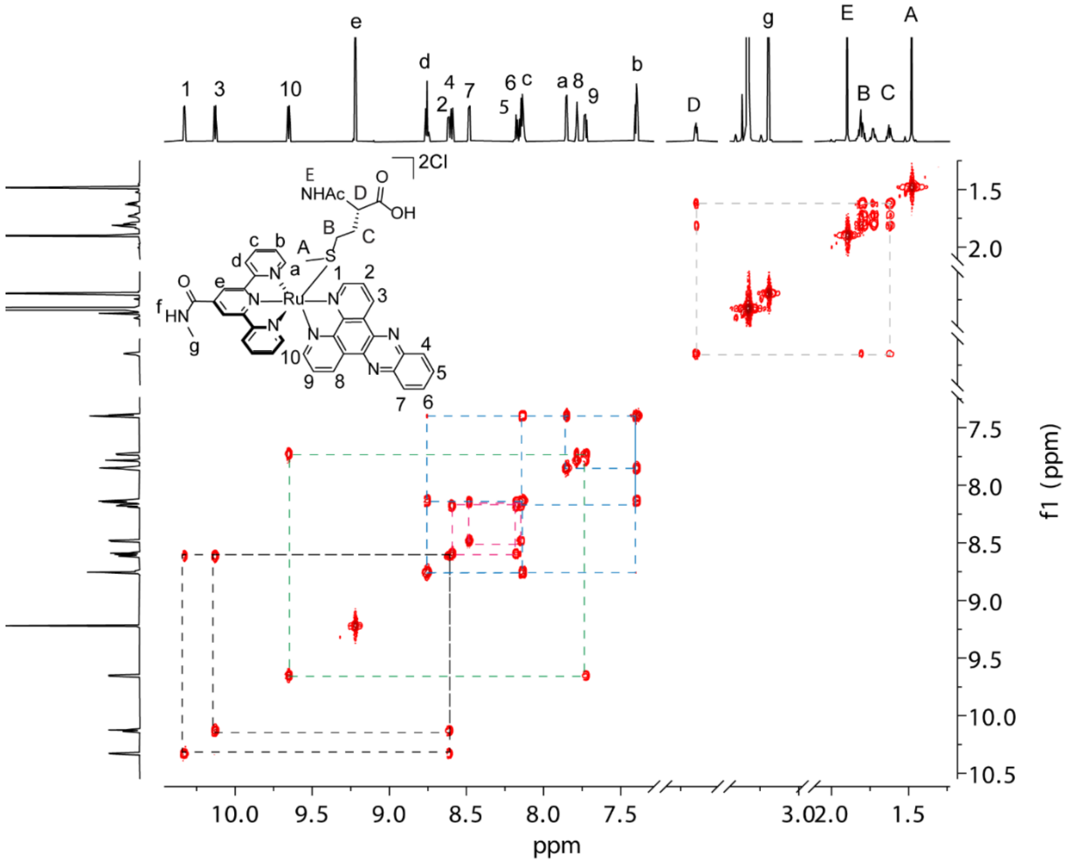

Figure S43 : Enlargement of ^1H-^1H COSY (850 MHz, MeOD) region of interest of $[Ru5]Cl_2$.

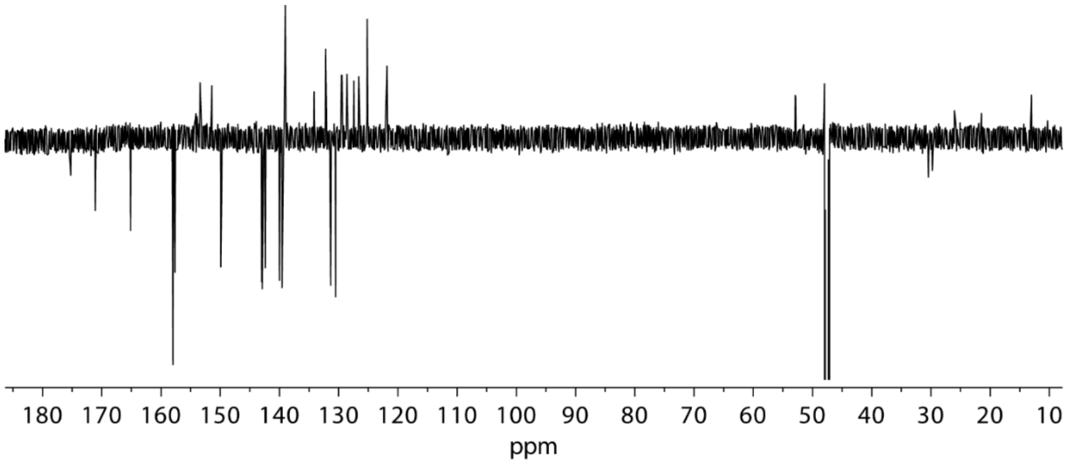

Figure S44 : ^{13}C NMR (214 MHz, MeOD) of $[Ru5]Cl_2$.

[Ru(tpa)(i-biq)(NAcMet)]Cl_2 – [Ru6]Cl_2

[pRu6]Cl (50 mg, 0.069 mmol, 1.0 equiv.) and N-Acetly-(L)-Methionine (106 mg, 0.556 mmol, 8.0 equiv.) were suspended in 4 mL of H_2O in a microwave vial. The mixture was degassed by bubbling nitrogen gas through it for 10 min. The reaction mixture was then stirred and heated in the sealed vial at 100 °C for 16 h with a conventional block. After cooling down to room temperature, the product was precipitated as $[Ru6](PF_6)_2$ salt by addition of saturated KPF_6 water (1 mL). The solid was isolated by centrifugation and subsequentially purified by flash column chromatography on silica gel and "Magic Mixture" (acetone:H_2O:sat$KPF_{6aq.}$ 8:1:1) as eluent. The hexafluorophosphate counterions were exchanged with chloride anions through an anion exchange column. The purity of the compounds was confirmed by analytical HPLC (Figure S60). The final product was obtained as a bright red solid in 80 % yield (51 mg, 0.056 mmol). ^1H NMR (850 MHz, MeOD) δ 10.60 (s, 1H, H1), 9.42 (s, 1H, H6), 9.20 (s, 2H, He), 9.18 (s, 1H, H7), 8.73 (tt, *J* = 8.0, 1.0 Hz, 2H, Hd), 8.62 (d, *J* = 8.1 Hz, 1H, H2), 8.45 – 8.38 (m, 1H, H5), 8.15 – 8.09 (m, 4H, Ha, H4, H11), 8.05 (ddd, *J* = 8.1, 7.1, 1.1 Hz, 1H, H3), 7.98 – 7.94 (m, 2H, Hc), 7.93 (d, *J* = 1.0 Hz, 1H, H12), 7.84 (ddd, *J* = 8.1, 6.7, 1.2 Hz, 1H, H10), 7.77 (dd, *J* = 8.5, 1.0 Hz, 1H, H8), 7.67 – 7.62 (m, 1H, H9), 7.44 (ddt, *J* = 7.2, 5.7, 1.4 Hz, 2H, Hb), 4.22 (t, *J* = 6.5 Hz, 1H, HD), 3.20 (s, 3H, Hg), 1.87 (s, 3H, HE), 1.86 – 1.78 - 1.64 (m, 4H, HC, HB), 1.42 (s, 3H, HA). ^{13}C NMR (214 MHz, MeOD) δ 171.14, 165.34, 158.17, 157.80, 152.90, 152.86, 152.79, 149.44, 149.15, 141.87, 138.84, 135.90, 135.33, 133.15, 130.17, 129.76, 129.72, 128.87, 128.55, 128.28, 127.49, 127.15, 127.09, 125.18, 121.82, 121.78, 121.15, 121.11, 120.43, 120.39, 47.89, 12.76. Elemental Analysis Calculated for $C_{42}H_{39}N_7O_4RuSCl_2$: C, 55.44; H, 4.32; N, 10.78. Found: C, 55.09; H, 4.59; N, 10.94. ESI MS calculated for $[C_{42}H_{39}N_7O_4RuS]^{2+}$: 419.6, measured: 419.2.

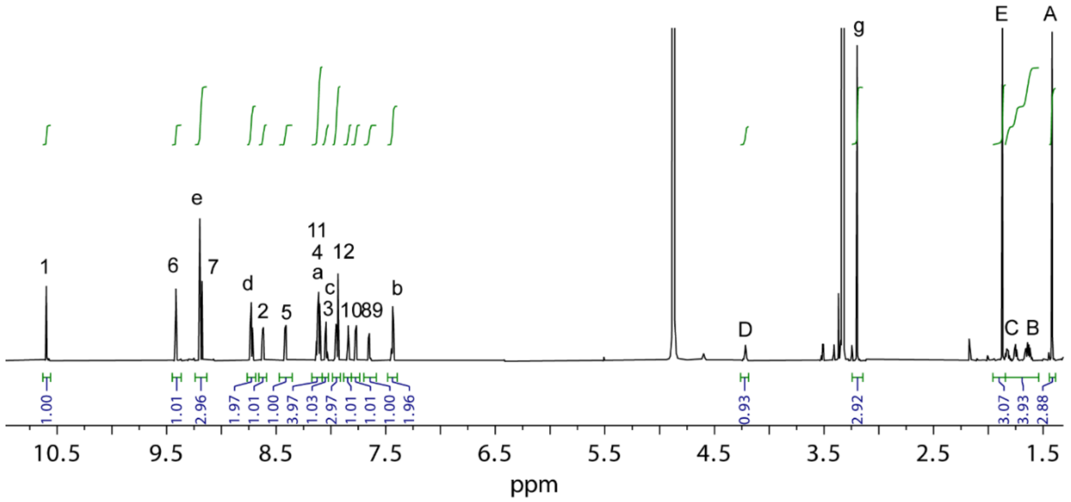

Figure S45 : ^{1}H NMR (850 MHz, MeOD) of $[Ru6]Cl_2$ with proton assignment.

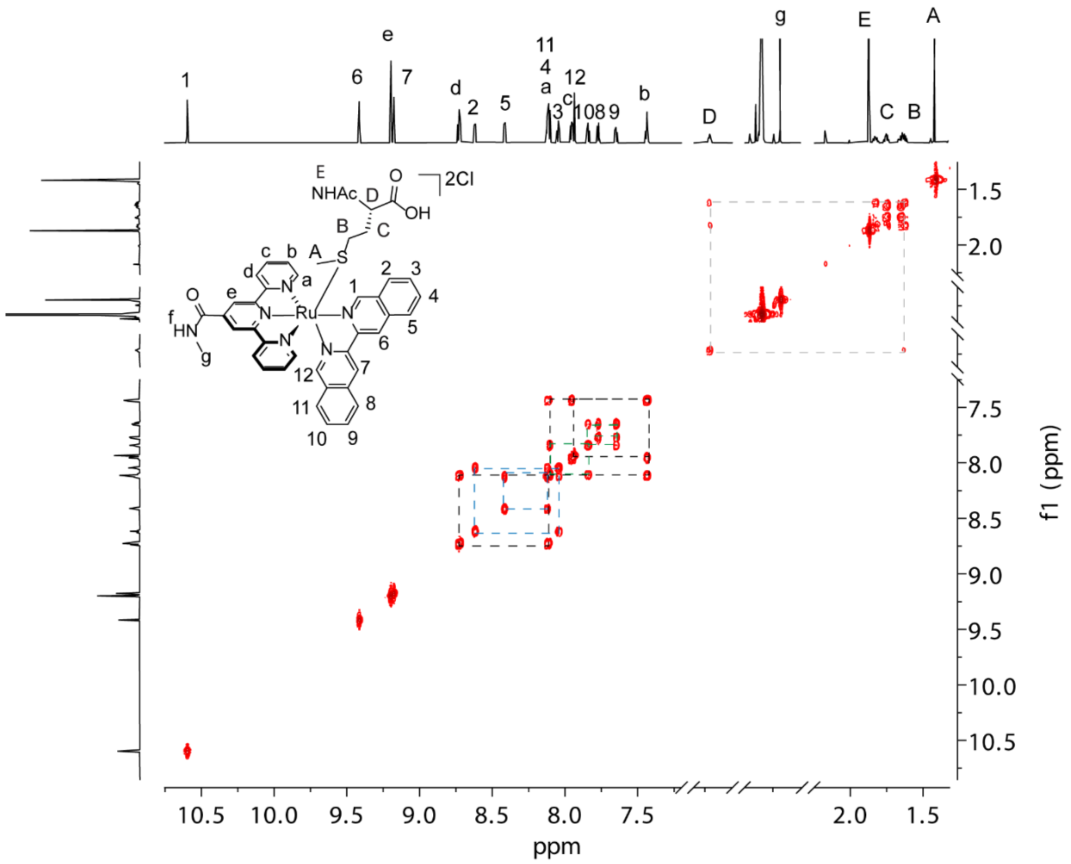

Figure S46 : Enlargement of ^{1}H-^{1}H COSY (850 MHz, MeOD) region of interest of $[Ru6]Cl_2$.

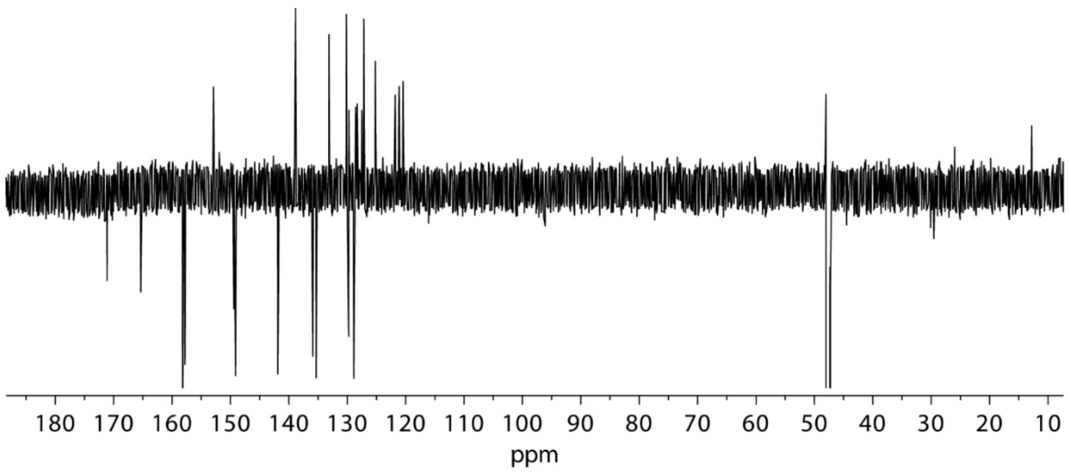

Figure S47 : ^{13}C NMR (214 MHz, MeOD) of $[Ru6]Cl_2$.

$[Ru(tpa)(i\text{-}Hdiqa)(NAcMet)]Cl_2$ – $[Ru7]Cl_2$

[pRu7]Cl (50 mg, 0.068 mmol, 1.0 equiv.) and N-Acetly-(L)-Methionine (104 mg, 0.545 mmol, 8.0 equiv.) were suspended in 4 mL of H_2O in a microwave vial. The mixture was degassed by bubbling nitrogen gas through it for 10 min. The reaction mixture was then stirred and heated in the sealed vial at 100 °C for 16 h with a conventional block. After cooling down to room temperature, the product was precipitated as $[Ru7](PF_6)_2$ salt by addition of saturated KPF_6 water (1 mL). The solid was isolated by centrifugation and subsequentially purified by flash column chromatography on silica gel and "Magic Mixture" (acetone:H_2O:sat$KPF_{6aq.}$ 8:1:1) as eluent. The hexafluorophosphate counterions were exchanged with chloride anions through an anion exchange column. The purity of the compounds was confirmed by analytical HPLC (Figure S61). The final product was obtained as a bright red solid in 46 % yield (46 mg, 0.050 mmol). ^1H NMR (400 MHz, MeOD) δ 10.18 (s, 1H, H1), 9.07 (dd, J = 9.8, 1.3 Hz, 2H, He, He'), 8.81 – 8.65 (m, 4H, Hd, Hd',Ha, Ha'), 8.46 (d, J = 8.3 Hz, 1H, H2), 8.24 (dtd, J = 9.4, 7.8, 1.5 Hz, 2H, Hc, Hc'), 8.09 (d, J = 8.4 Hz, 1H, H5), 7.97 – 7.89 (m, 2H, H6), 7.77 – 7.57 (m, 5H, H3, Hb, Hb', H9, H12), 7.52 – 7.46 (m, 2H, H10, H8), 7.39 (s, 1H, H13), 7.31 (ddd, J = 8.1, 6.6, 1.2 Hz, 1H, H11), 4.06 (t, J = 6.2 Hz, 1H, HD), 3.14 (s, 3H, Hg), 1.83 (s, 3H, HA), 1.79 – 1.43 (m, 4H, HC, HB), 1.22 (s, 3H, HE). ^{13}C NMR (214 MHz, MeOD) δ 172.35, 171.62, 164.99, 159.92, 158.75, 158.08, 158.07, 158.01, 154.44, 154.15, 150.99, 150.95, 150.16, 149.83, 141.46, 139.09, 138.68, 137.87, 133.12, 132.99, 128.30, 128.20, 127.85, 127.42, 126.87, 126.43, 126.40, 125.87, 125.45, 125.34, 125.17, 124.84, 121.85, 121.81, 121.71, 121.67, 108.87, 107.42, 107.38, 50.17, 50.12, 30.06, 28.50, 25.94, 25.89, 14.03, 12.92. Elemental Analysis Calculated for $C_{42}H_{40}N_8O_4RuSCl_2$: C, 54.54; H, 4.36; N, 12.12. Found: C, 53.66; H, 4.62; N, 11.86. ESI MS calculated for $[C_{42}H_{40}N_8O_4RuS]^{2+}$: 427.1, measured: 426.9.

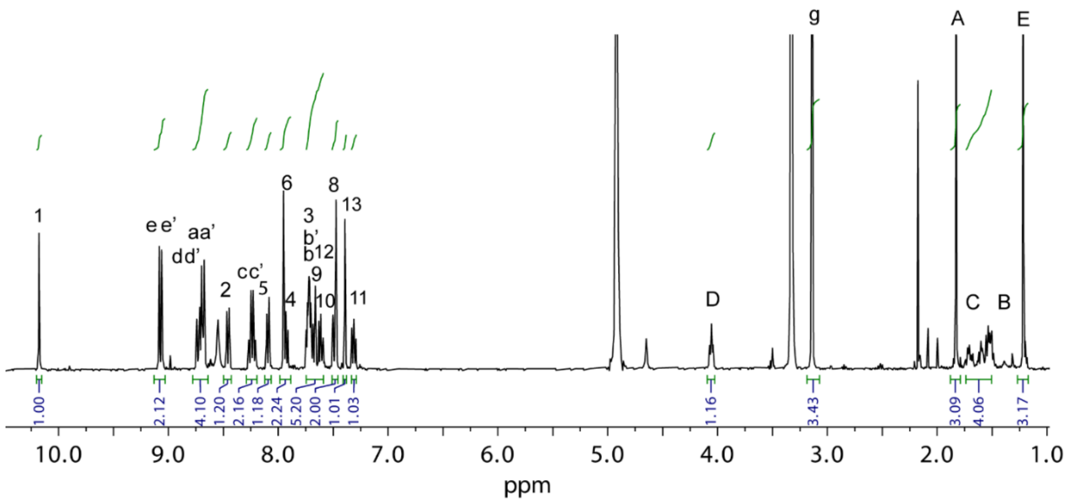

Figure S48 : ^{1}H NMR (400 MHz, MeOD) of $[Ru7]Cl_2$ with proton assignment.

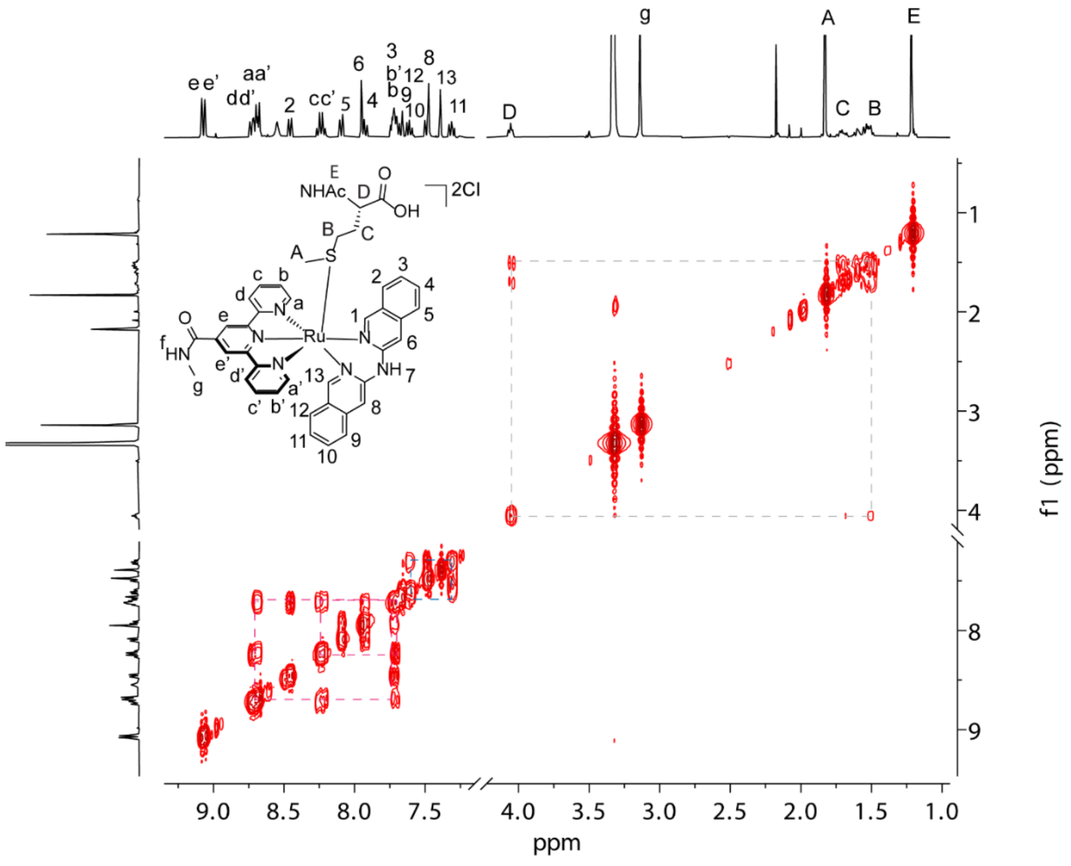

Figure S49 : Enlargement of ^{1}H-^{1}H COSY (400 MHz, MeOD) region of interest of $[Ru7]Cl_2$.

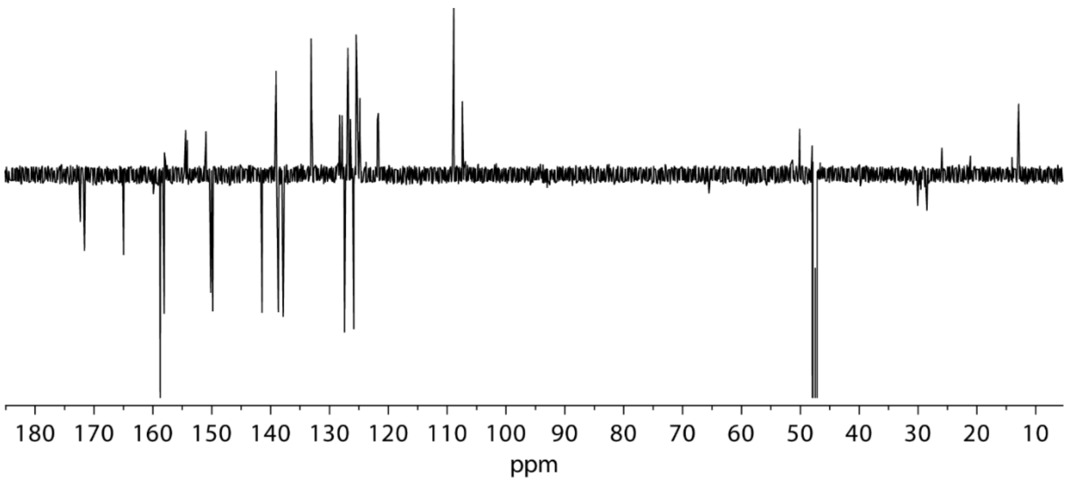

Figure S50 : ^{13}C NMR (214 MHz, MeOD) of $[Ru7]Cl_2$

$[Ru(tpa)(Hdpa)(NAcMet)]Cl_2$ – $[Ru8]Cl_2$

[pRu8]Cl (50 mg, 0.079 mmol, 1.0 equiv.) and N-Acetly-(L)-Methionine (104 mg, 0.631 mmol, 8.0 equiv.) were suspended in 4 mL of H_2O in a microwave vial. The mixture was degassed by bubbling nitrogen gas through it for 10 min. The reaction mixture was then stirred and heated in the sealed vial at 100 °C for 16 h with a conventional block. After cooling down to room temperature, the product was precipitated as $[Ru8](PF_6)_2$ salt by addition of saturated KPF_6 water (1 mL). The solid was isolated by centrifugation and subsequentially purified by flash column chromatography on silica gel and “Magic Mixture” (acetone:H_2O:sat$KPF_{6aq.}$ 8:1:1) as eluent. The hexafluorophosphate counterions were exchanged with chloride anions through an anion exchange column. The purity of the compounds was confirmed by analytical HPLC (Figure S61). The final product was obtained as a bright red solid in 89 % yield (58 mg, 0.070 mmol). ^1H NMR (850 MHz, MeOD) δ 9.16 – 9.07 (m, 3H, H1, He, He’), 8.81 – 8.69 (m, 2H, Hd, Hd’), 8.62 (m, 2H, Ha, Ha’), 8.25 (m, 2H, Hc, Hc’), 8.20 (ddd, J = 8.6, 7.2, 1.7 Hz, 1H, H3), 7.80 – 7.73 (m, 2H, Hb, Hb’), 7.64 – 7.54 (m, 3H, H4, H7, H2), 7.04 (dd, J = 8.2, 1.3 Hz, 1H, H6), 6.69 (dd, J = 6.3, 1.7 Hz, 1H, H8), 6.62 (ddd, J = 7.5, 6.2, 1.3 Hz, 1H, H9), 4.22 (dd, J = 8.9, 5.0 Hz, 1H, HD), 3.13 (s, 3H, Hg), 1.90 (s, 3H, HE), 1.86 – 1.45 (m, 4H, HC, HB), 1.23 (s, 3H, HE). ^{13}C NMR (214 MHz, MeOD) δ 172.20, 171.68, 164.88, 158.81, 158.79, 158.16, 154.81, 154.42, 153.93, 153.79, 153.14, 153.09, 146.37, 141.19, 139.96, 139.31, 139.28, 139.06, 128.36, 128.31, 125.18, 125.11, 121.67, 121.63, 121.57, 121.53, 121.00, 119.61, 118.38, 115.46, 114.04, 114.02, 50.18, 50.12, 30.06, 28.43, 25.93, 25.87, 21.17, 21.12, 12.87. Elemental Analysis Calculated for $C_{34}H_{36}N_8O_4RuSCl_2$: C, 49.52; H, 4.40; N, 13.59. Found: C, 49.11; H, 4.61; N, 13.43. ESI MS calculated for $[C_{34}H_{36}N_8O_4RuS]^{2+}$: 377.1, measured: 377.2.

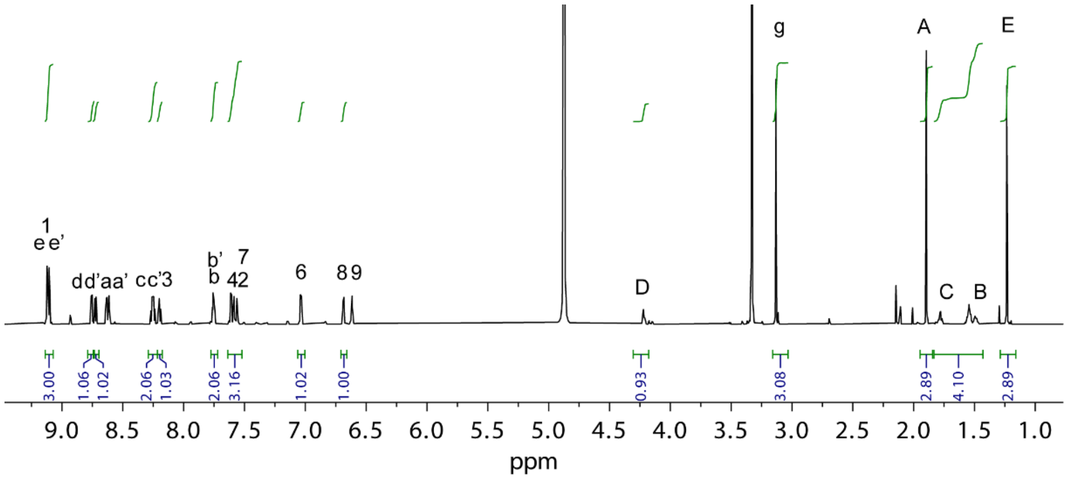

Figure S51 : ^{1}H NMR (850 MHz, MeOD) of $[Ru8]Cl_2$ with proton assignment.

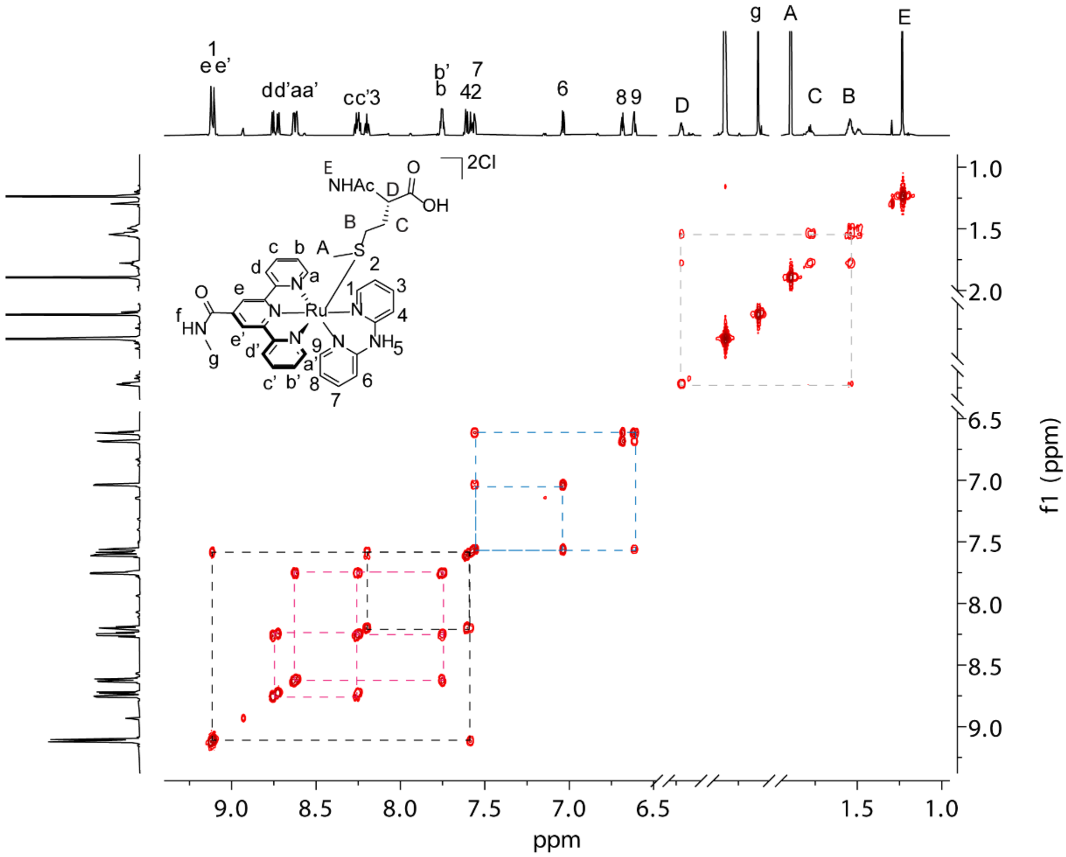

Figure S52 : Enlargement of ^{1}H-^{1}H COSY (850 MHz, MeOD) region of interest of $[Ru8]Cl_2$.

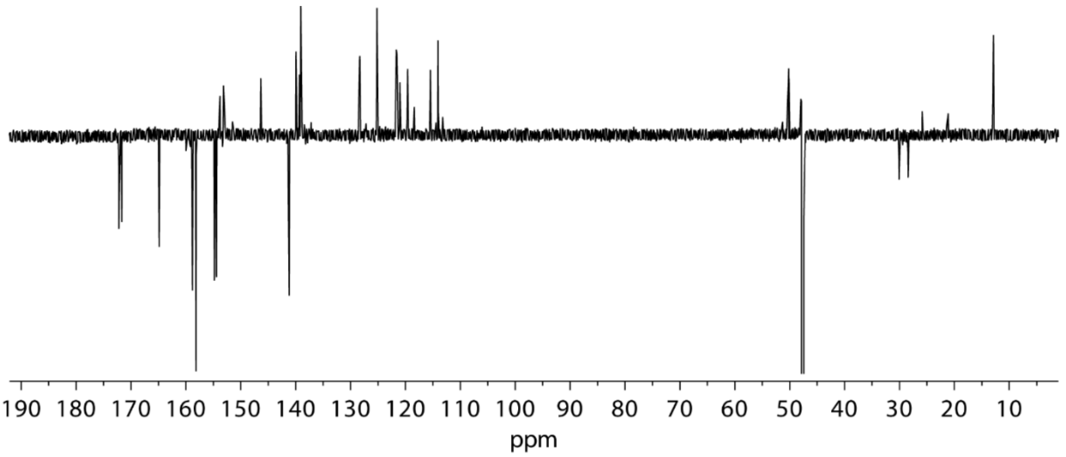

Figure S53 : ^{13}C NMR (214 MHz, MeOD) of $[Ru8]Cl_2$

[Ru(tpa)(Medpa)(NAcMet)]Cl_2 – [Ru9]Cl_2

[pRu9]Cl (50 mg, 0.079 mmol, 1.0 equiv.) and N-Acetly-(L)-Methionine (104 mg, 0.631 mmol, 8.0 equiv.) were suspended in 4 mL of H_2O in a microwave vial. The mixture was degassed by bubbling nitrogen gas through it for 10 min. The reaction mixture was then stirred and heated in the sealed vial at 100 °C for 16 h with a conventional block. After cooling down to room temperature, the product was precipitated as $[Ru9](PF_6)_2$ salt by addition of saturated KPF_6 water (1 mL). The solid was isolated by centrifugation and subsequentially purified by flash column chromatography on silica gel and "Magic Mixture" (acetone:H_2O:sat$KPF_{6aq.}$ 8:1:1) as eluent. The hexafluorophosphate counterions were exchanged with chloride anions through an anion exchange column. The purity

of the compounds was confirmed by analytical HPLC (Figure S61). The final product was obtained as a bright red solid in 76 % yield (49 mg, 0.058 mmol). ^{1}H NMR (850 MHz, MeOD, 298K) δ 9.28 (d, J = 5.8 Hz, 1H, H1), 8.33 (ddd, J = 8.7, 7.3, 1.7 Hz, 1H, H3), 7.83 (dd, J = 8.6, 1.3 Hz, 1H, H4), 7.78 – 7.64 (m, 2H, H2, H7), 7.29 (dd, J = 8.5, 1.3 Hz, 1H, H6), 6.78 (dd, J = 6.1, 1.8 Hz, 1H, H8), 6.74 (ddd, J = 7.3, 6.1, 1.2 Hz, 1H, H9), 3.94 (t, J = 6.2 Hz, 1H, HD), 3.57 (s, 3H, H5), 3.13 (s, 3H, Hg), 1.87 (s, 3H, HA), 1.73 - 1.40 (m, 4H, HC, HB), 1.24 (s, 3H, HE). ^{1}H NMR (500 MHz, MeOD, 328 K) δ 9.27 (dd, J = 5.8, 1.7 Hz, 1H, H1), 9.01 (s, 2H, He), 8.69 (s, 2H, Hd), 8.43 (t, J = 6.2 Hz, 2H, Ha), 8.32 (ddd, J = 8.7, 7.3, 1.8 Hz, 1H, H3), 8.23 (td, J = 7.8, 3.9 Hz, 2H, Hc), 7.82 (d, J = 8.5 Hz, 1H, H4), 7.79 – 7.66 (m, 4H, H7, H2, Hb), 7.28 (d, J = 8.4 Hz, 1H, H6), 6.81 – 6.68 (m, 2H, H8, H9), 3.89 (t, J = 6.0 Hz, 1H, HD), 3.57 (s, 3H, H5), 3.13 (s, 3H, Hg), 1.86 (s, 3H, Ha), 1.68 – 1.43 (m, 4H, HC, HB), 1.25 (s, 3H, HE). ^{13}C NMR (214 MHz, MeOD, 298 K) δ 175.19, 170.94, 168.45, 165.28, 158.47, 158.32, 158.05, 157.97, 153.85, 147.06, 141.40, 140.30, 139.70, 138.94, 122.32, 120.68, 116.87, 115.19, 53.17, 39.37, 30.33, 29.93, 25.95, 21.48, 13.09. Elemental Analysis Calculated for $C_{34}H_{36}N_8O_4RuSCl_2$: C, 50.12; H, 4.57; N, 13.36. Found: C, 48.30; H, 4.89; N, 13.09. ESI MS calculated for $[C_{34}H_{36}N_8O_4RuS]^{2+}$: 384.1, measured: 384.0.

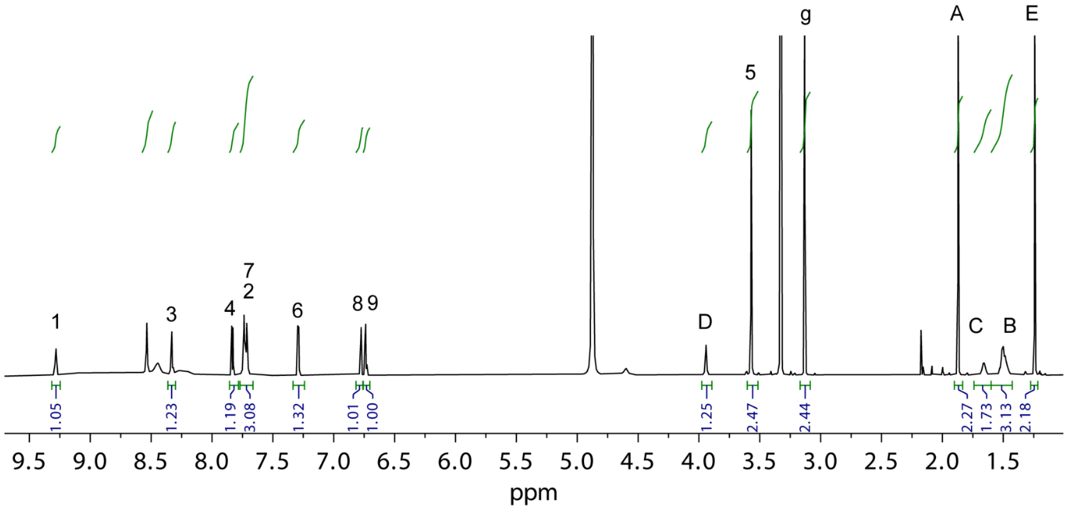

Figure S54 : ^{1}H NMR (500 MHz, MeOD) at 298 K of $[Ru9]Cl_2$ with proton assignment.

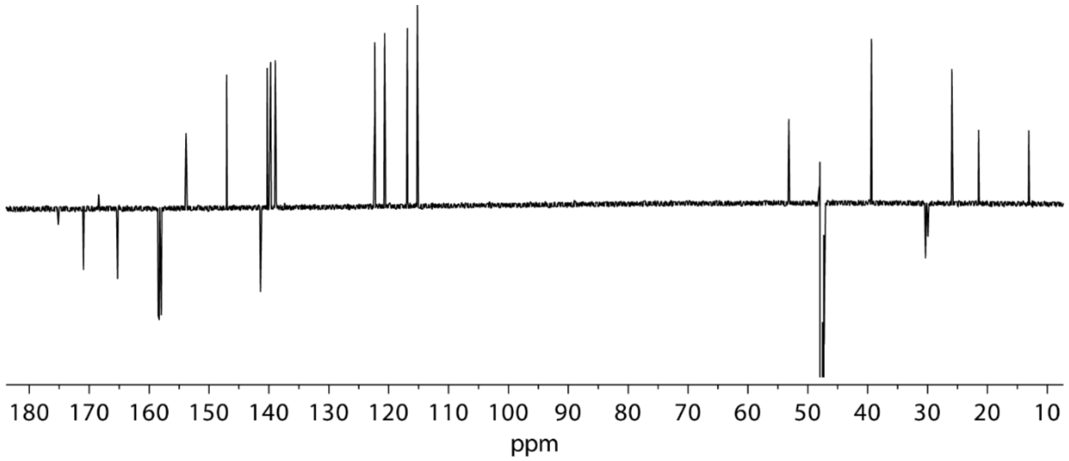

Figure S55 : ^{13}C NMR (214 MHz, MeOD) at 298 K of $[Ru9]Cl_2$.

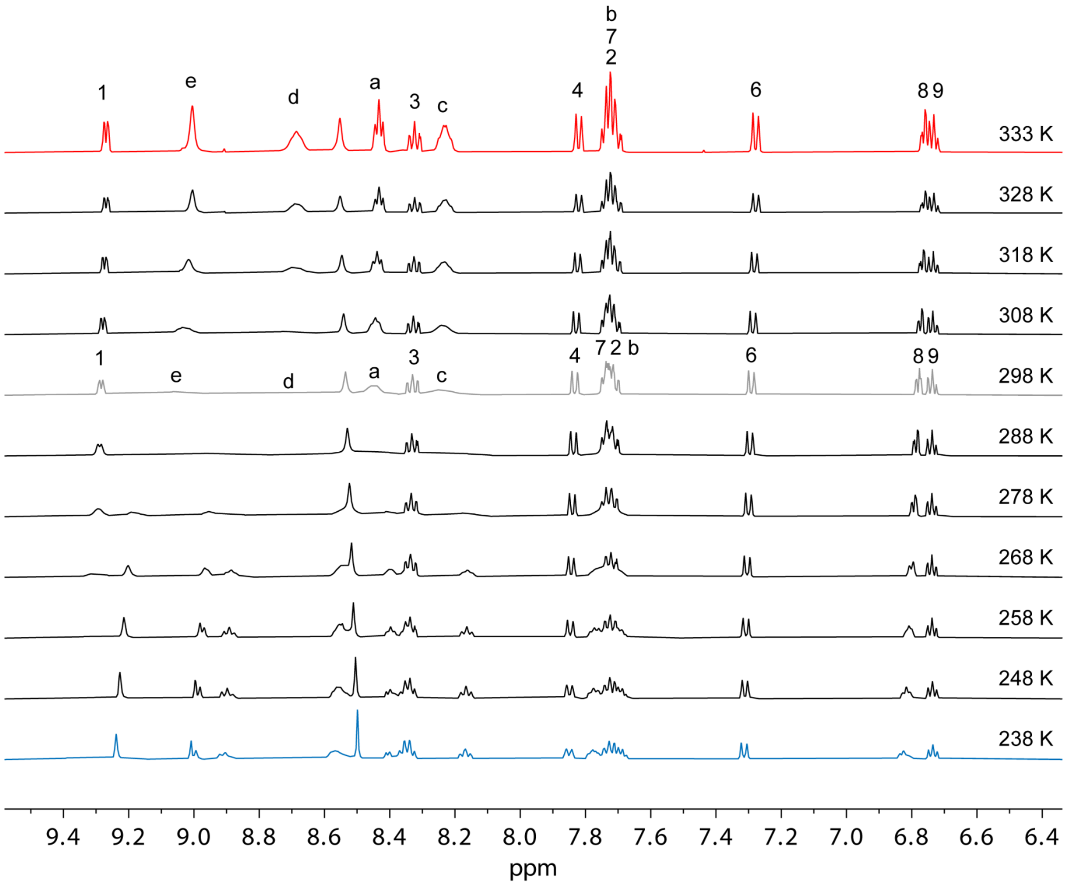

Figure S56 : Variable Temperature ^{1}H NMR (500 MHz, MeOD) of $[Ru9]Cl_2$.

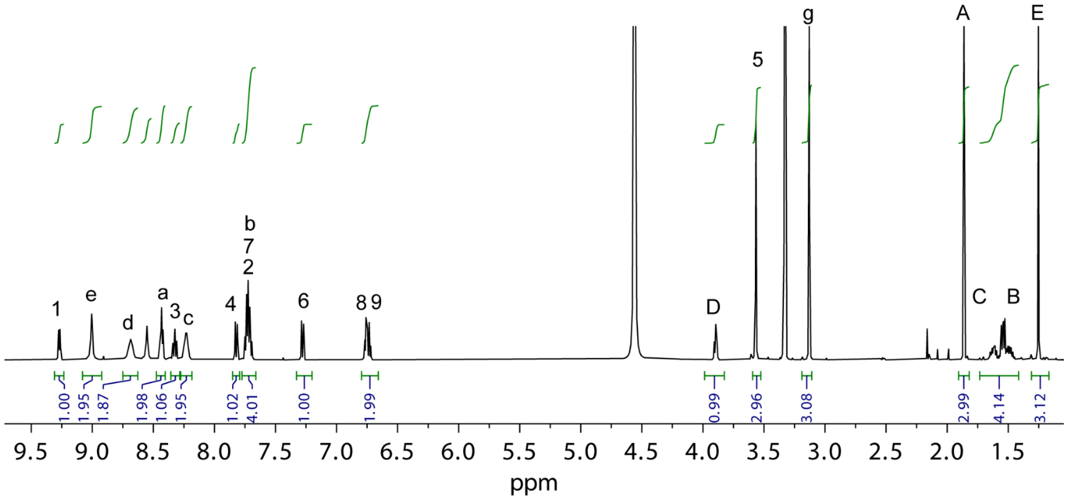

Figure S57 : ^{1}H NMR (500 MHz, MeOD) at 328 K of $[Ru9]Cl_2$ with proton assignment.

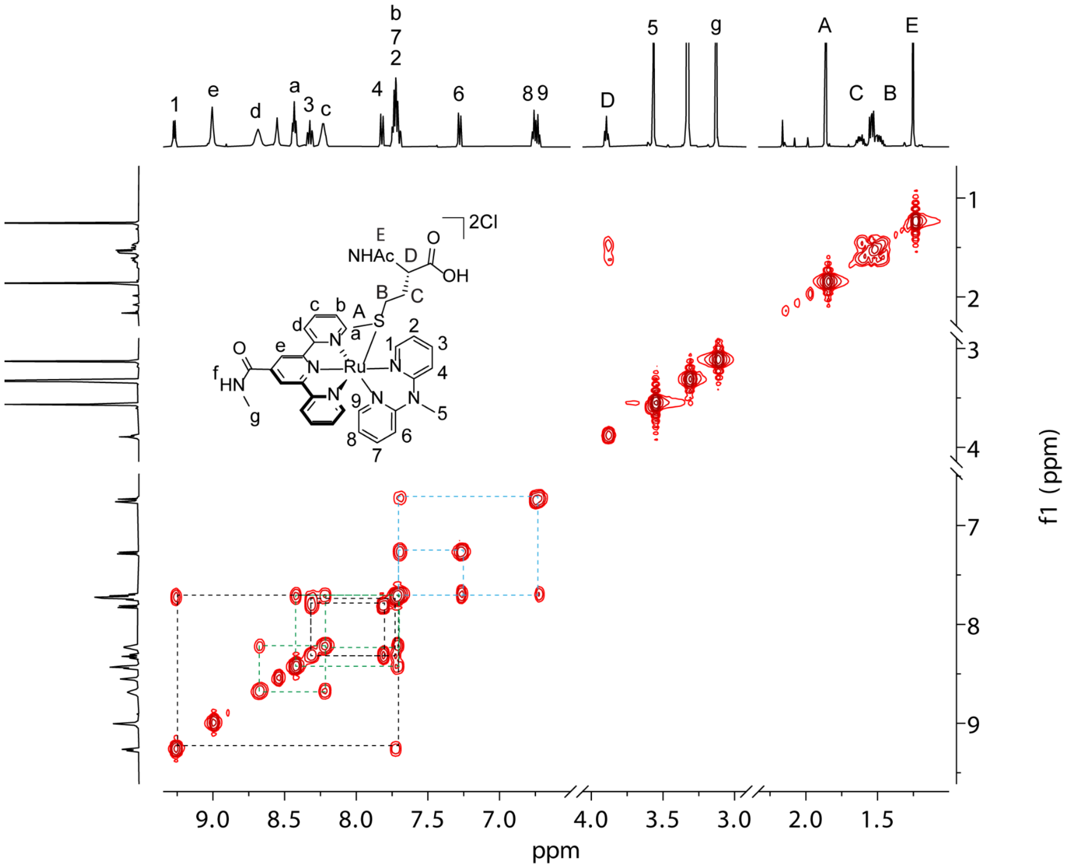

Figure S58 : Enlargement of ^{1}H-^{1}H COSY (500 MHz, MeOD) region of interest at 328 K of $[Ru9]Cl_2$.

Analytical HPLC

The gradient used for separation was ACN:H_2O 10:90 -> 90:10 in 20 min with a flow rate of 2 mL/min. The product fractions were monitored by four UV detectors, and the traces were collected at the UV detector of 272 nm.

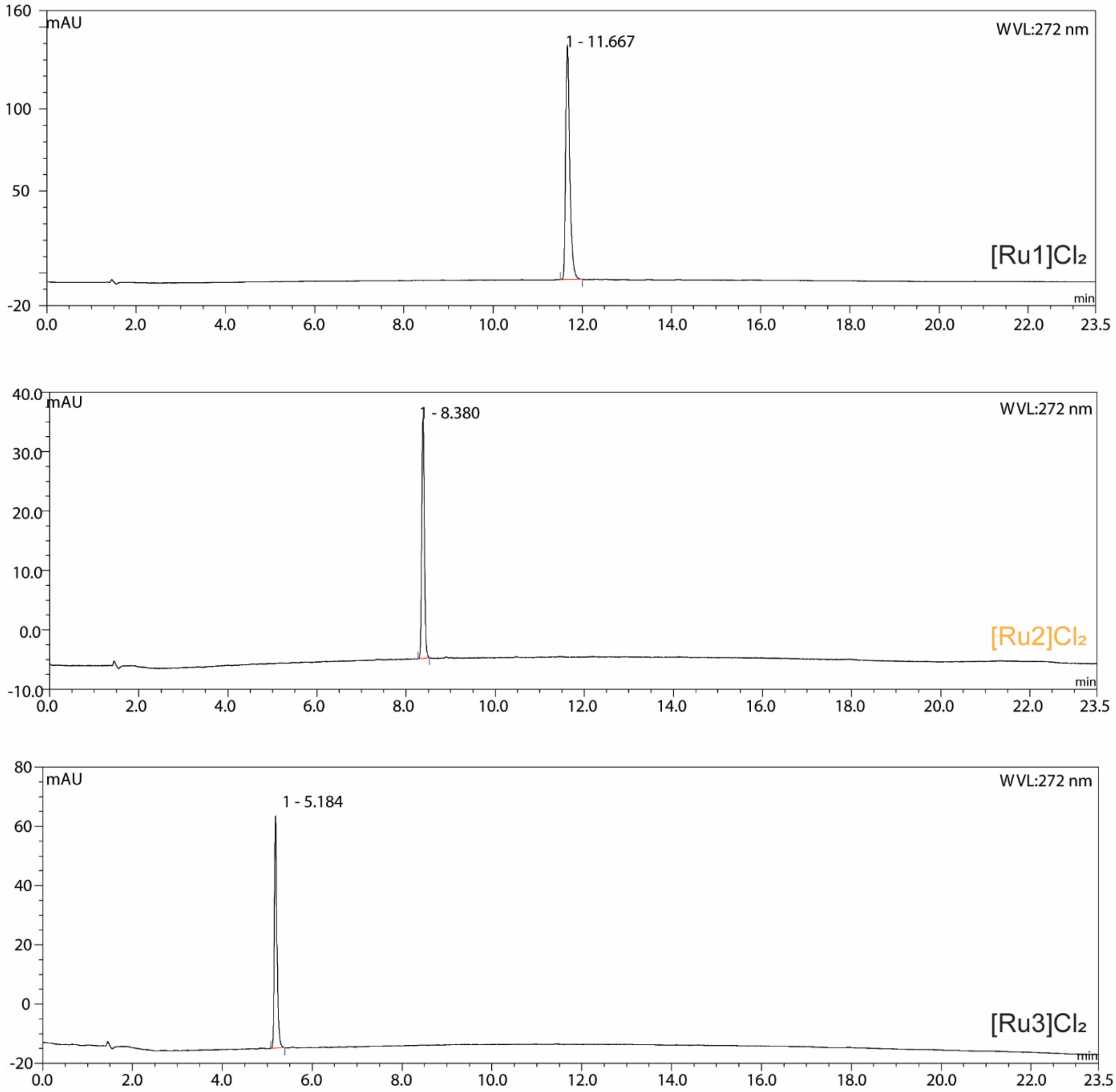

Figure S59: Analytical HPLC trace of [Ru1]Cl_2 -[Ru3]Cl_2.

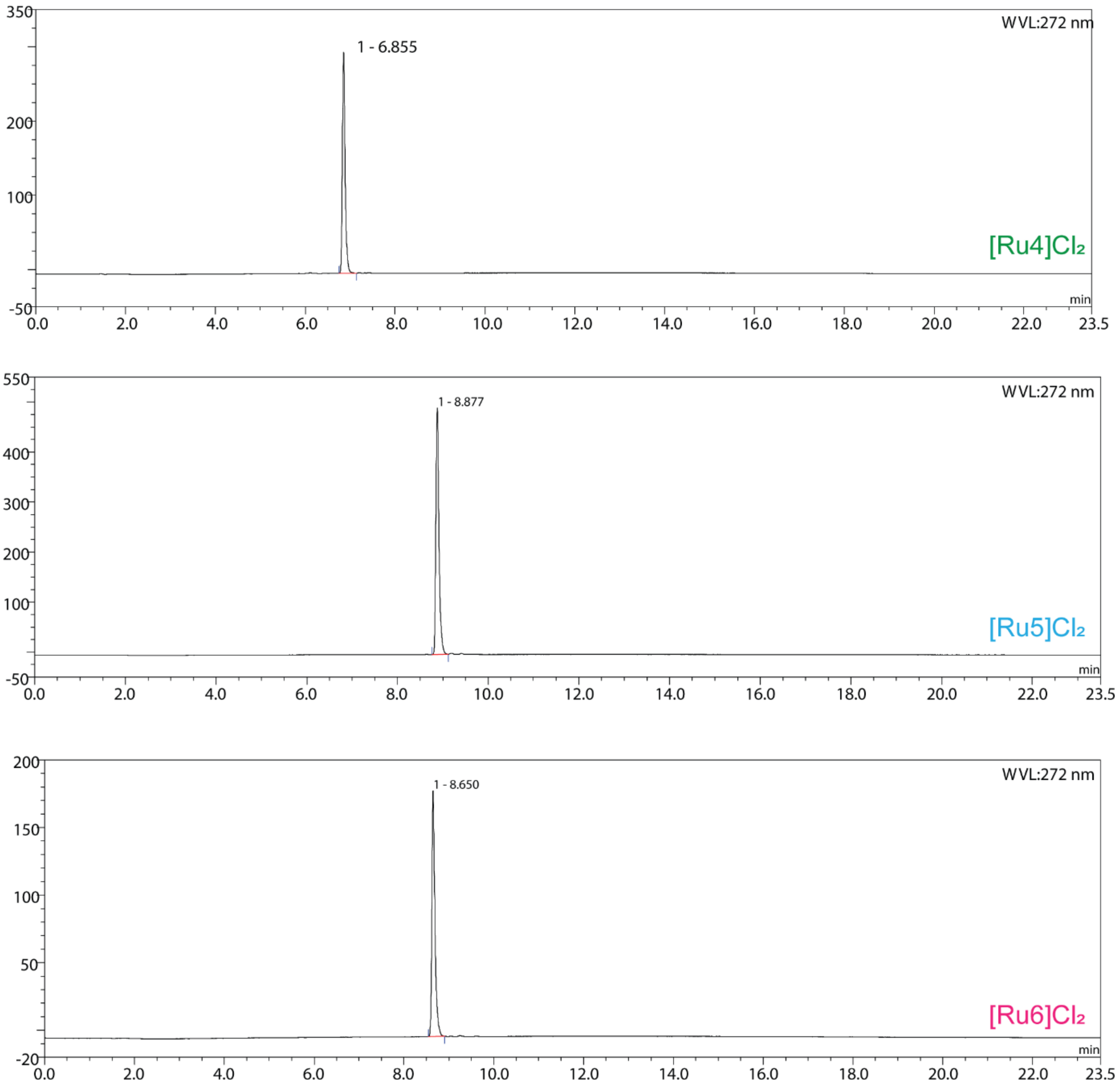

Figure S60: Analytical HPLC trace of $[Ru4]Cl_2$ -$[Ru6]Cl_2$.

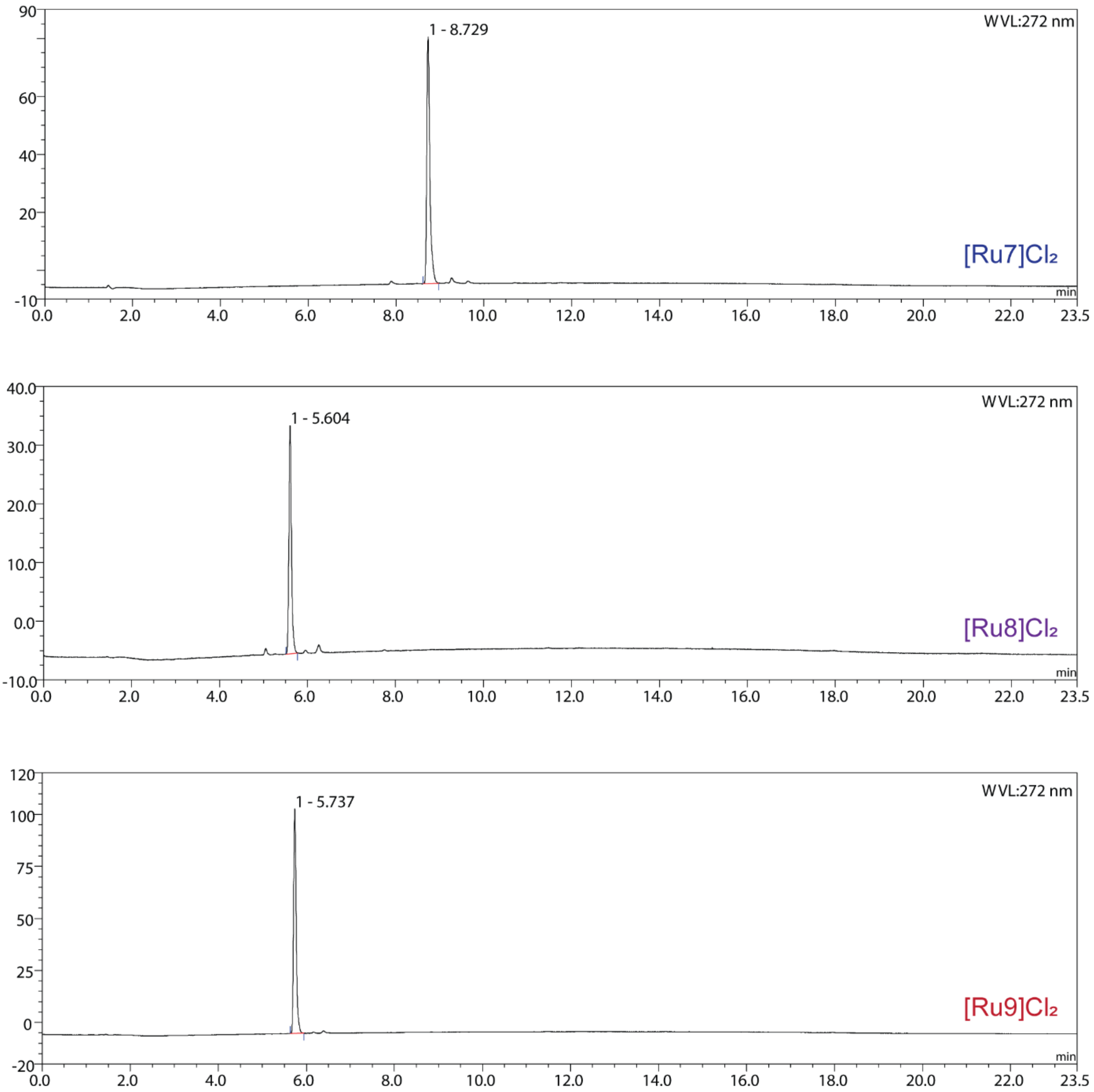

Figure S61: Analytical HPLC trace of [Ru7]Cl_2 -[Ru9]Cl_2.

3. Photochemistry

Absorption and emission properties of [Ru1]Cl_2 -[Ru9]Cl_2

For determination of the molar absorption coefficient (ε), the compounds were dissolved in milliQ to make a stock solution of 100 μM. This solution was then diluted to solutions of 25 μM, 50 μM, 75 μM and 100 μM. The absorption of these different concentrations was measured with Agilent Technologies Cary 60 UV-Vis spectrometer. The resulting data was used to determine the ε at λ_{max}

with the use of Lambert-Beer Law. Emission spectra were measured in water at 25 °C with an excitation wavelength of 480 nm.

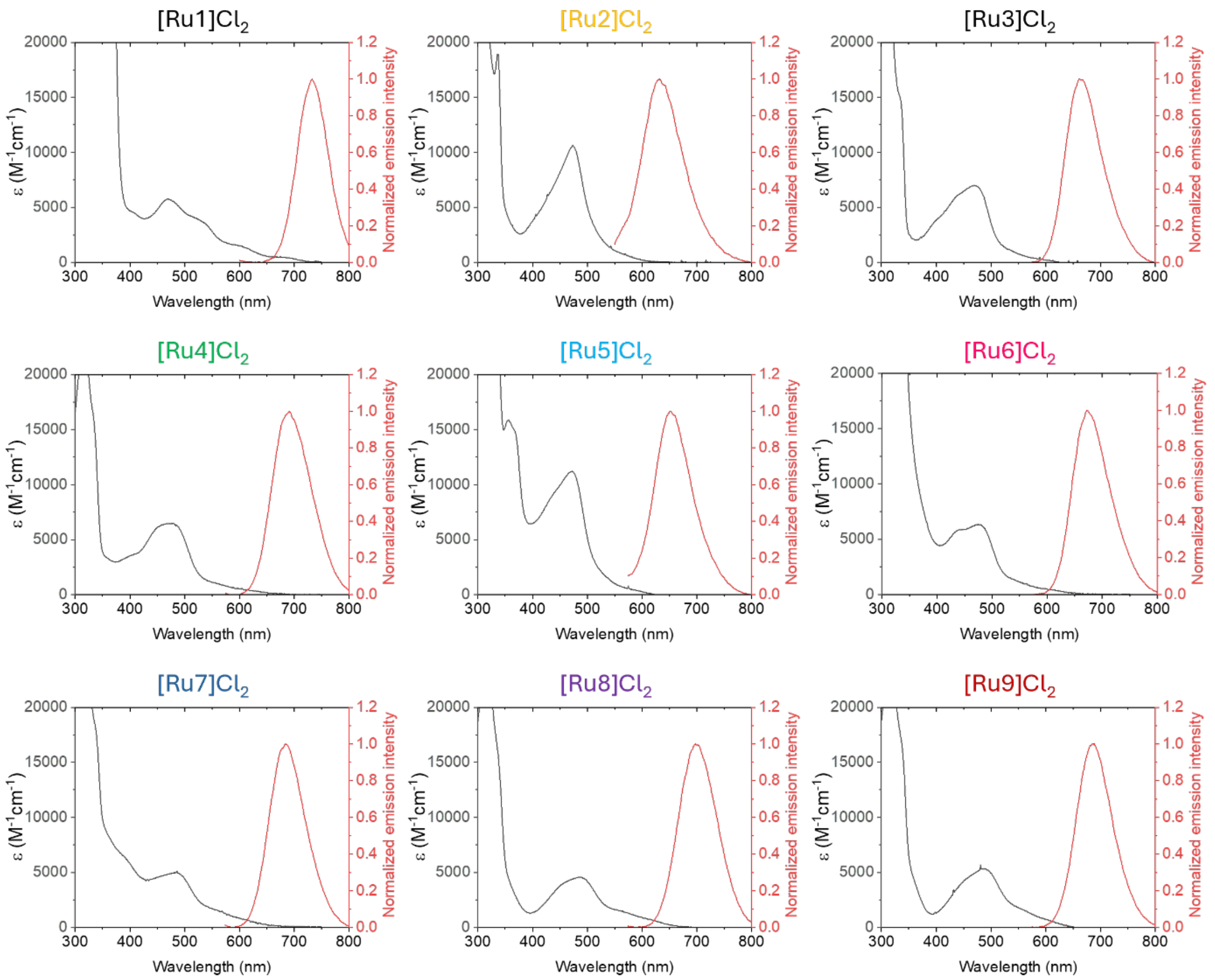

Figure S62: Absorption and normalized emission spectra of $[Ru1]Cl_2$ -$[Ru9]Cl_2$ in water.

Phosphorescence Quantum Yields

The steady-state emission measurements were performed on a previously reported custom-built setup utilizing a slightly modified experimental procedure, results are presented in **Table S1** and **Figure S63** below.[5] $[Ru(bpy)_3]Cl_2$ was used as a reference with reported $\Phi_P = 0.040 \pm 0.002$ in air-saturated H_2O.[6] All the compounds were dissolved in 3 mL of H_2O and transferred into a macro fluorescence cuvette from Hellma Analytics (lightpaths: 1 cm x 1 cm). The irradiation of samples was done at 298 K using 450 nm LRD0450 Laserglow fiber-coupled laser set to 80 mW at the cuvette with help of PM100USB Thorlabs power meter. The UV-Vis and emission spectra were recorded at 298 K with Agilent Cary 60 UV-Vis and Avantes 2048L StarLine spectrometers respectively. The emission spectrums were acquired within 100 ms. All the spectral data was processed with OriginPro 9.1 and MS Excel 2016.

Table S1: Determination of phosphorescence quantum yields in H_2O.

Compound	Absorbance at 450 nm	Integrated emission	Emission λ_{max}, nm	Φ_P
$[Ru(bpy)_3]Cl_2$	0.0963	1552.8924	619	0.04000
Ru1	0.0857	35.9731	664	0.00104
Ru2	0.1088	4.9182	632	0.00011
Ru3	0.1243	73.1765	773	0.00146
Ru4	0.0975	97.8511	691	0.00249
Ru5	0.1137	81.6640	652	0.00178
Ru6	0.1175	92.7042	676	0.00196
Ru7	0.0951	38.7728	687	0.00101
Ru8	0.0959	42.0595	700	0.00109
Ru9	0.1012	58.9684	688	0.00145

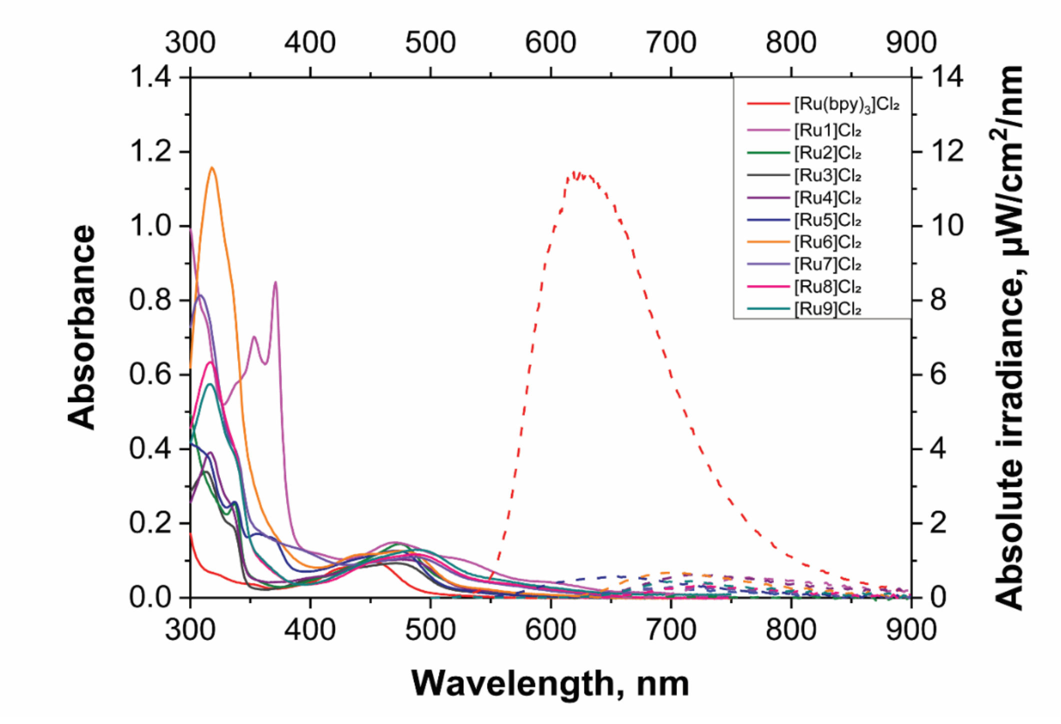

Figure S63: Absorption (solid) and emission (dash) spectrums of compounds from **Table 1**, the absorption at 450 nm was adjusted to 0.1 for all of them.

Singlet oxygen quantum yield measurement

The steady-state singlet oxygen emission measurements were performed on a previously reported custombuilt setup utilizing a slightly modified experimental procedure, results are presented in **Table S2** and **Figure S64-66** below.[5,7,8] Perinaphthenone was used as a reference with reported Φ_Δ ($^1O_2$) = 0.98 ± 0.07 in airsaturated ACN.[9] [Ru(bpy)$_3$]Cl$_2$ was used as validation compound with reported Φ_Δ ($^1O_2$) = 0.57 ± 0.06 in airsaturated ACN.[10] The NIR spectra were acquired within 20 s at 298 K with Avantes NIR256-1.7TEC spectrometer. Other conditions were similar to those described in the phosphorescence section above.

Table S2: Determination of singlet oxygen quantum yields in ACN for [Ru1]Cl$_2$.

**Compound**	**Absorbance at 450 nm**	**Integrated emission**	**Φ_Δ**
Perinaphthenone	0.0981	1.1673	0.980
[Ru(bpy)$_3$]Cl$_2$	0.0997	0.6869	0.568
[Ru1]Cl$_2$	0.1027	0.0558	0.045

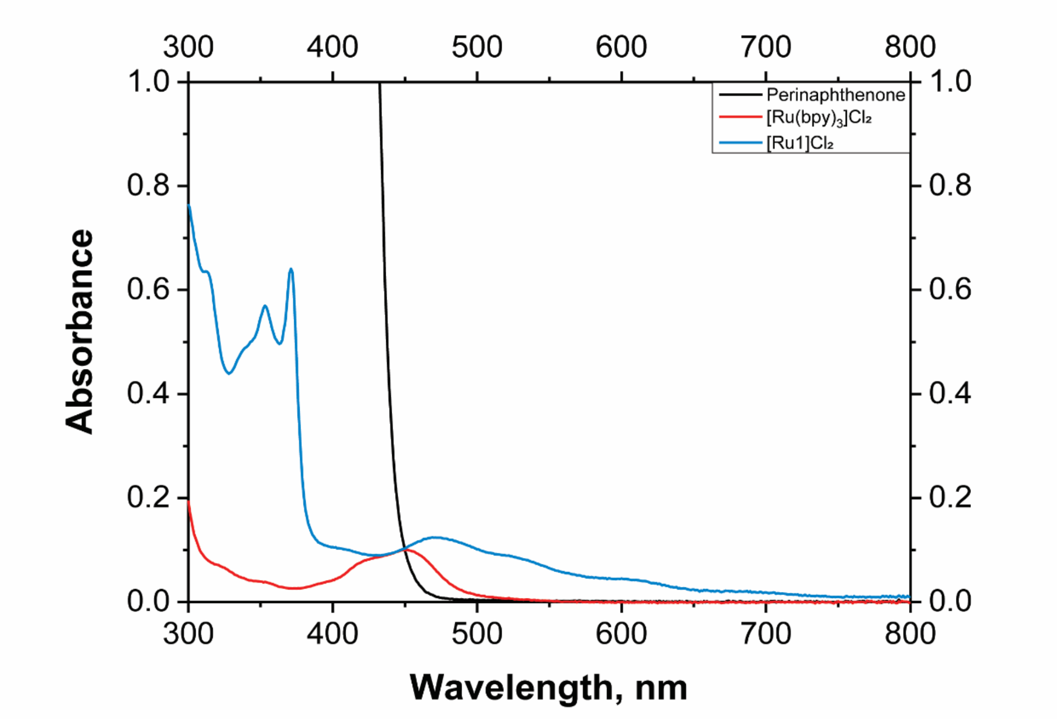

Figure S64: UV-Vis spectra of compounds from **Table S2** in ACN, the absorption at 450 nm was adjusted to 0.1 for all of them.

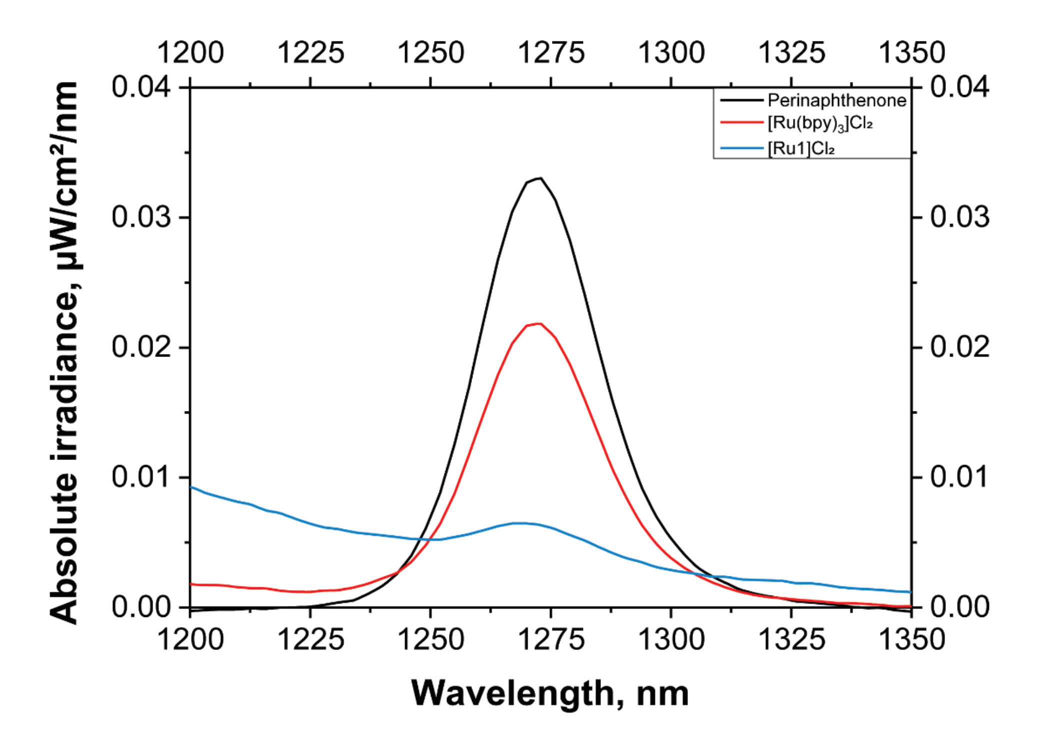

Figure S65: Raw NIR emission spectra of compounds from **Table S2** in ACN.

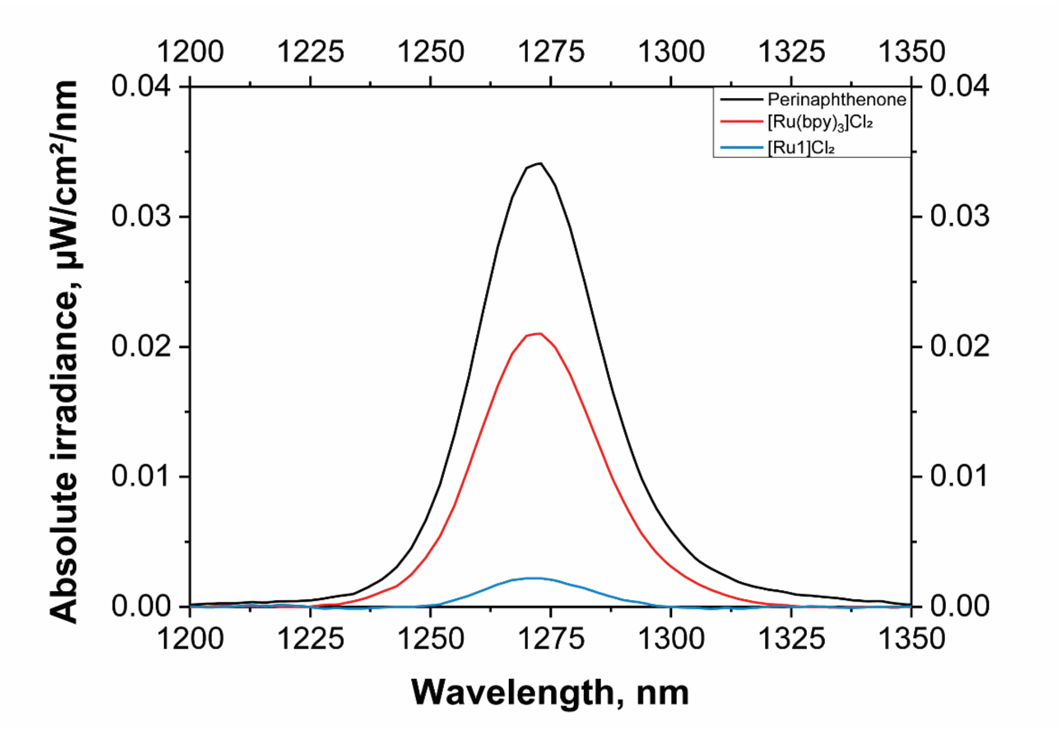

Figure S66: Normalized NIR emission spectra of compounds from **Table S2** in ACN for proper graphical comparison (backgrounds were subtracted and the emission intensities were adjusted as if compounds absorbance at 450 nm was exactly 0.1).

Table S3: Determination of singlet oxygen quantum yields in ACN for $[Ru2]Cl_2$ - $[Ru9]Cl_2$.

Compound	Absorbance at 450 nm	Integrated emission	Φ_Δ
Perinaphthenone	0.1177	0.0984	0.980
$[Ru(bpy)_3]Cl_2$	0.0947	0.0496	0.614
$[Ru2]Cl_2$	0.1094	<0.0001	<0.0001
$[Ru3]Cl_2$	0.0993	<0.0001	<0.0001
$[Ru4]Cl_2$	0.1157	<0.0001	<0.0001
$[Ru5]Cl_2$	0.1067	<0.0001	<0.0001
$[Ru6]Cl_2$	0.1280	<0.0001	<0.0001
$[Ru7]Cl_2$	0.1188	0.0028	0.027
$[Ru8]Cl_2$	0.1164	0.0018	0.018
$[Ru9]Cl_2$	0.0942	0.0020	0.025

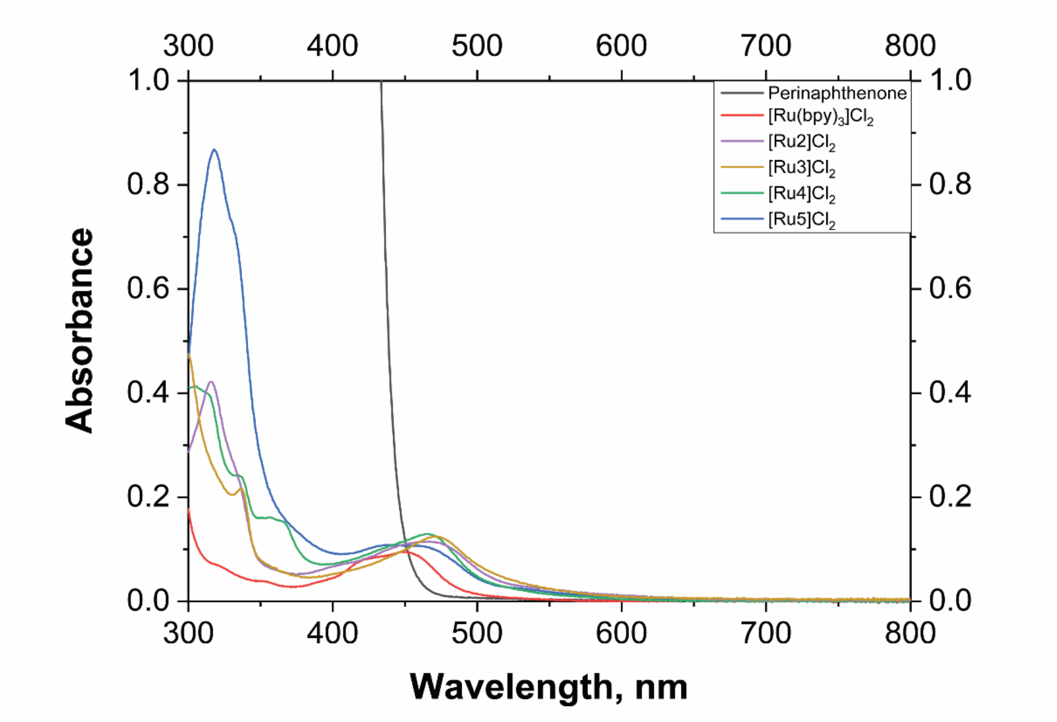

Figure S67: UV-Vis spectra of compounds from **Table S3** $[Ru2]Cl_2$ - $[Ru5]Cl_2$ in ACN, the absorption at 450 nm was adjusted to 0.1 for all of them.

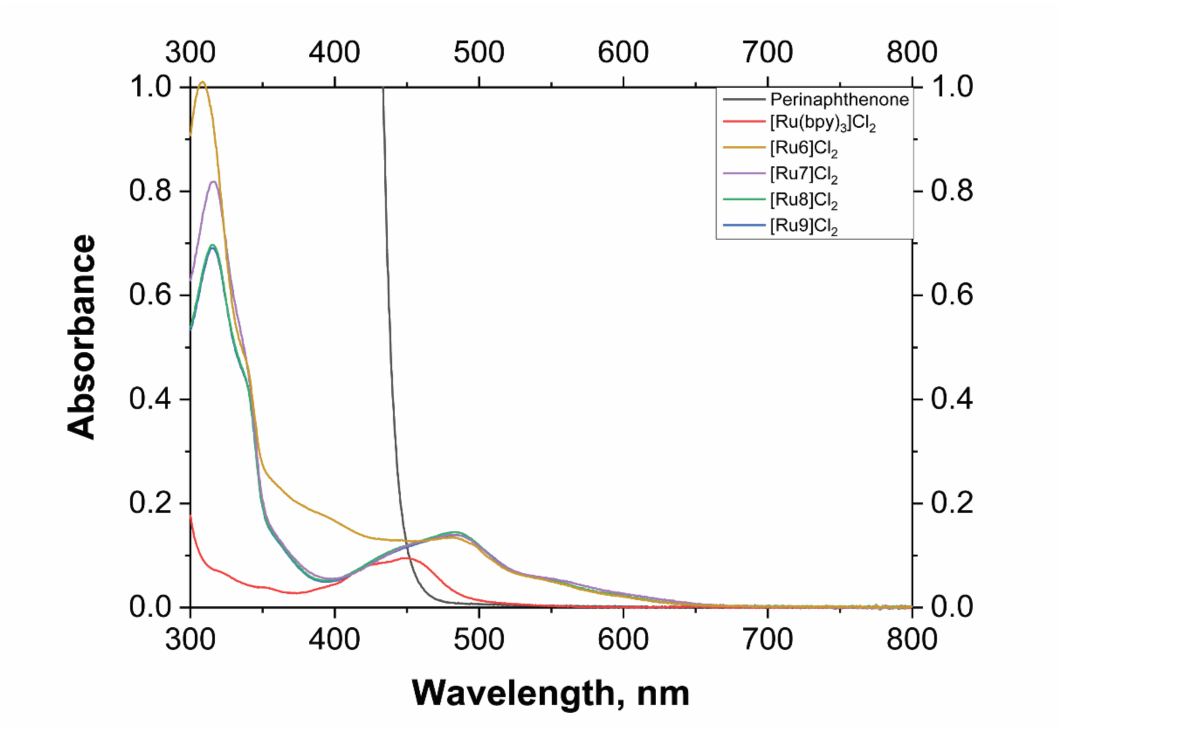

Figure S68: UV-Vis spectra of compounds from **Table 3** [Ru6]Cl_2 - [Ru9]Cl_2 in ACN, the absorption at 450 nm was adjusted to 0.1 for all of them.

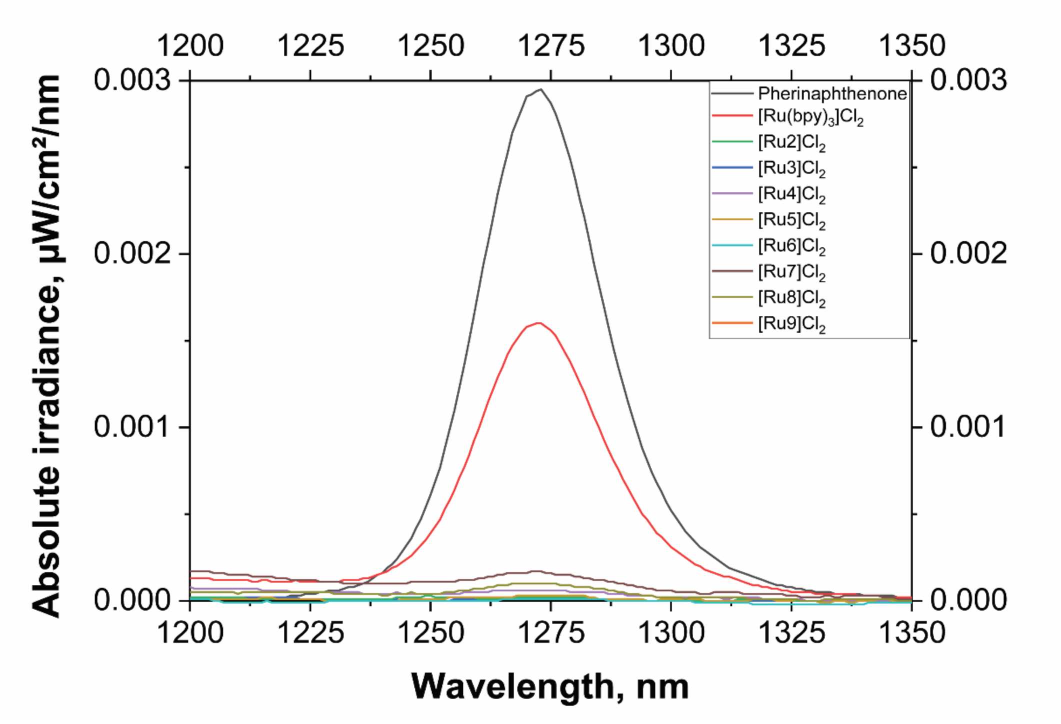

Figure S69: Raw NIR emission spectra of compounds from **Table 3** in ACN.

Ligand Photosubstition Properties

Photosubstitution experiments were performed with a 1 cm quartz cuvette containing 3 mL of a 50-100 μM solution of the compound in milliQ. This solution was irradiated for 120 min or until there was no visible change. The UV-Vis spectrum was measured from 250-800 nm, every 30 or 60 s. The solutions were irradiated from the top of the cuvette, with either a 625 nm (red, 12.4 mW) or a 515 nm (green, 3.3 mW) LED. The solution was magnetically stirred during irradiation and kept at 25 °C. Mass spectra of the solution were recorded before and after irradiation to show the photosubstitution process had reached completion and confirm the nature of the photoproducts.

To test the thermal stability of the compounds, solutions were kept in the dark for 16 h, and absorption spectra were measured every 30 min. Mass spectra were also recorded immediately after sample preparation and again after 24 h. For compounds [Ru5]Cl_2 and [Ru9]Cl_2, ^1H NMR spectra in D_2O were collected immediately after preparation, after 24 h in the dark, and following sequential irradiation with green light (520 nm).

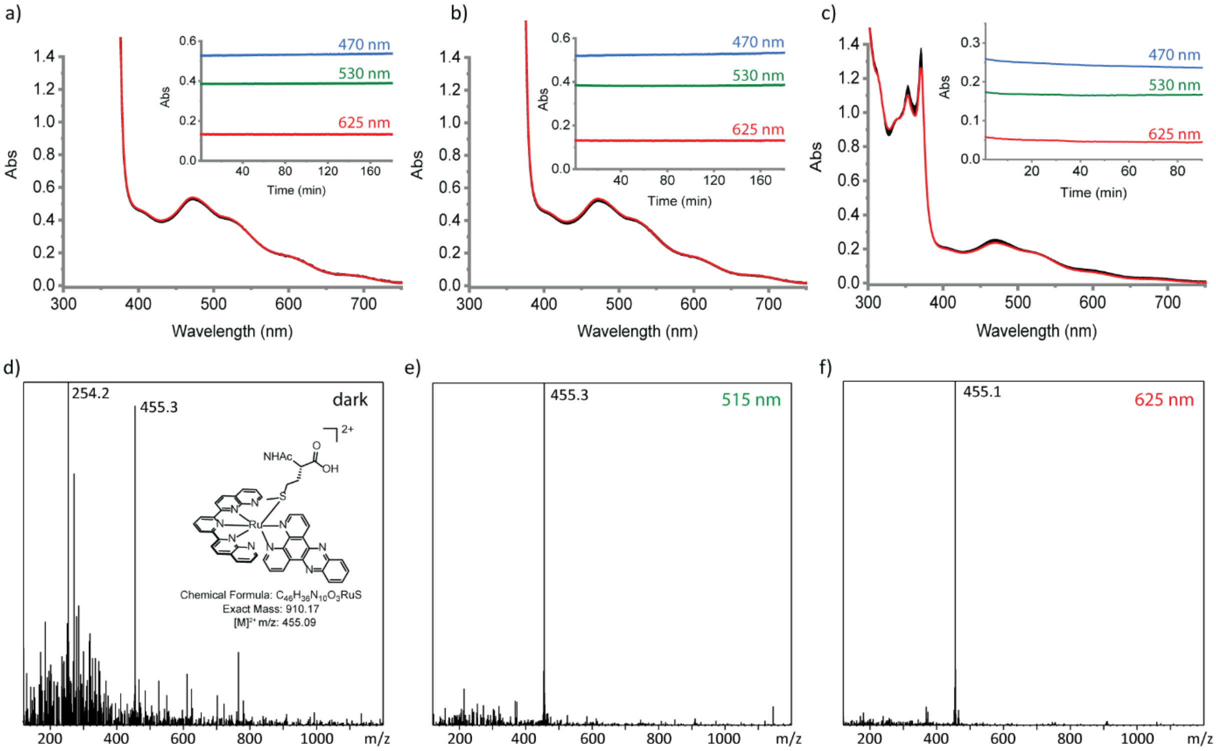

Figure S70: Irradiation of [Ru1]Cl_2 a) in acetonitrile with 515 nm; b) in acetonitrile with 625 nm and c) in water with 515 nm. ESI MS spectra of water solution of d) sample kept in the dark for 24 h, e) sample irradiated with 515 nm light for 6 hours and f) sample irradiated with 625 nm light for 6 hours. No visible change can be observed upon irradiation. The expected m/z for [Ru1]Cl_2 is 455.09, which can be observed in d)-f).

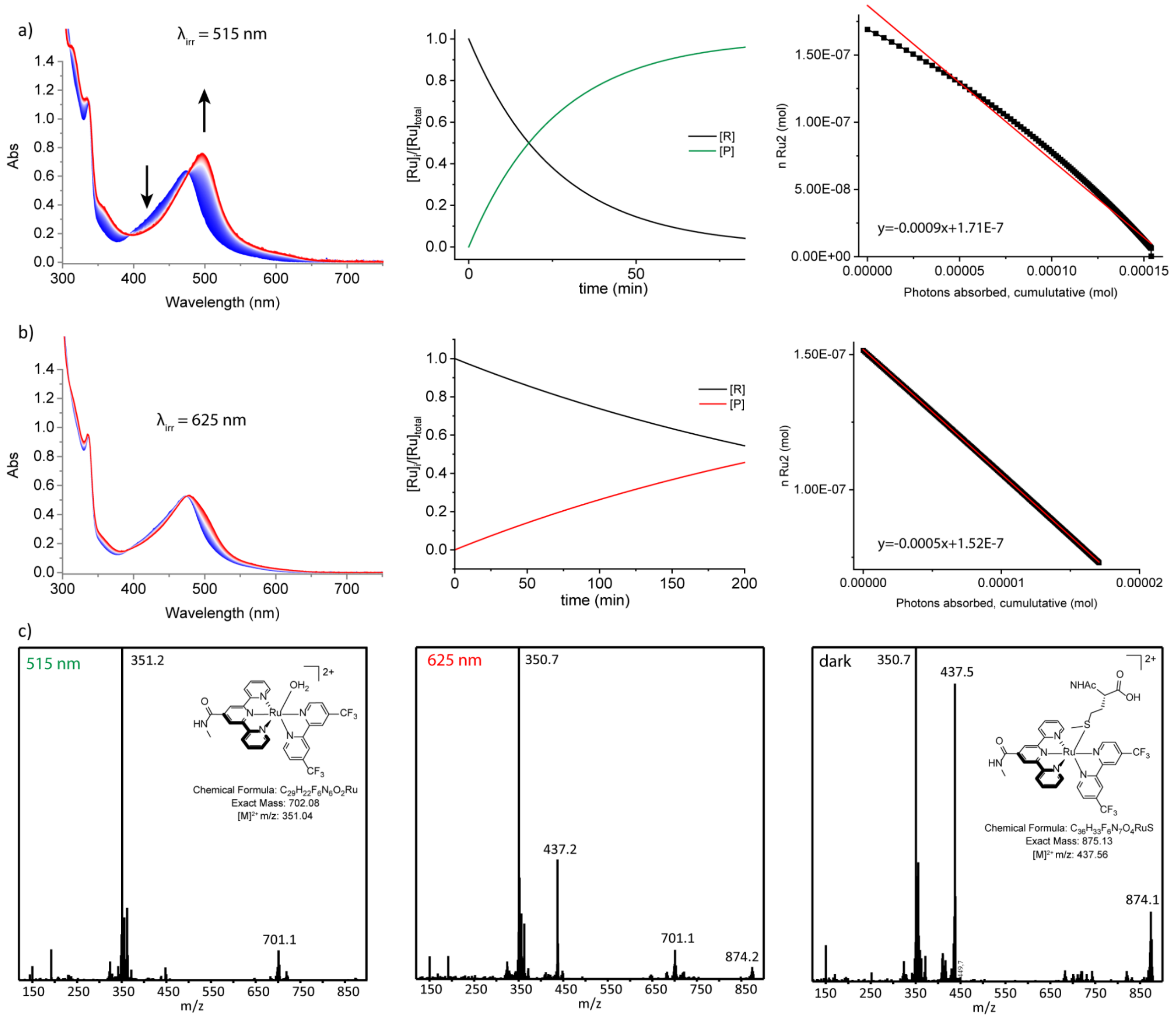

Figure S71: Investigation of the photosubstitution reactivity of [Ru2]Cl_2 in water under a) 515 nm and b) 625 nm light irradiation. From left to right are reported the absorption spectral change, the molar ratio change over time and the amount of initial reagent plotted against the cumulative amount of photons absorbed. The slopes are numerically equal to the opposite of the photosubstitution quantum yield. c) ESI MS spectra of a sample of the complex under green light irradiation, red light irradiation and kept in the dark for 24 h. The expected m/z for [Ru2]Cl_2 is 437.56, which can be observed in the sample kept in the dark, and the expected m/z of the correspondent aqua complex is 351.04.

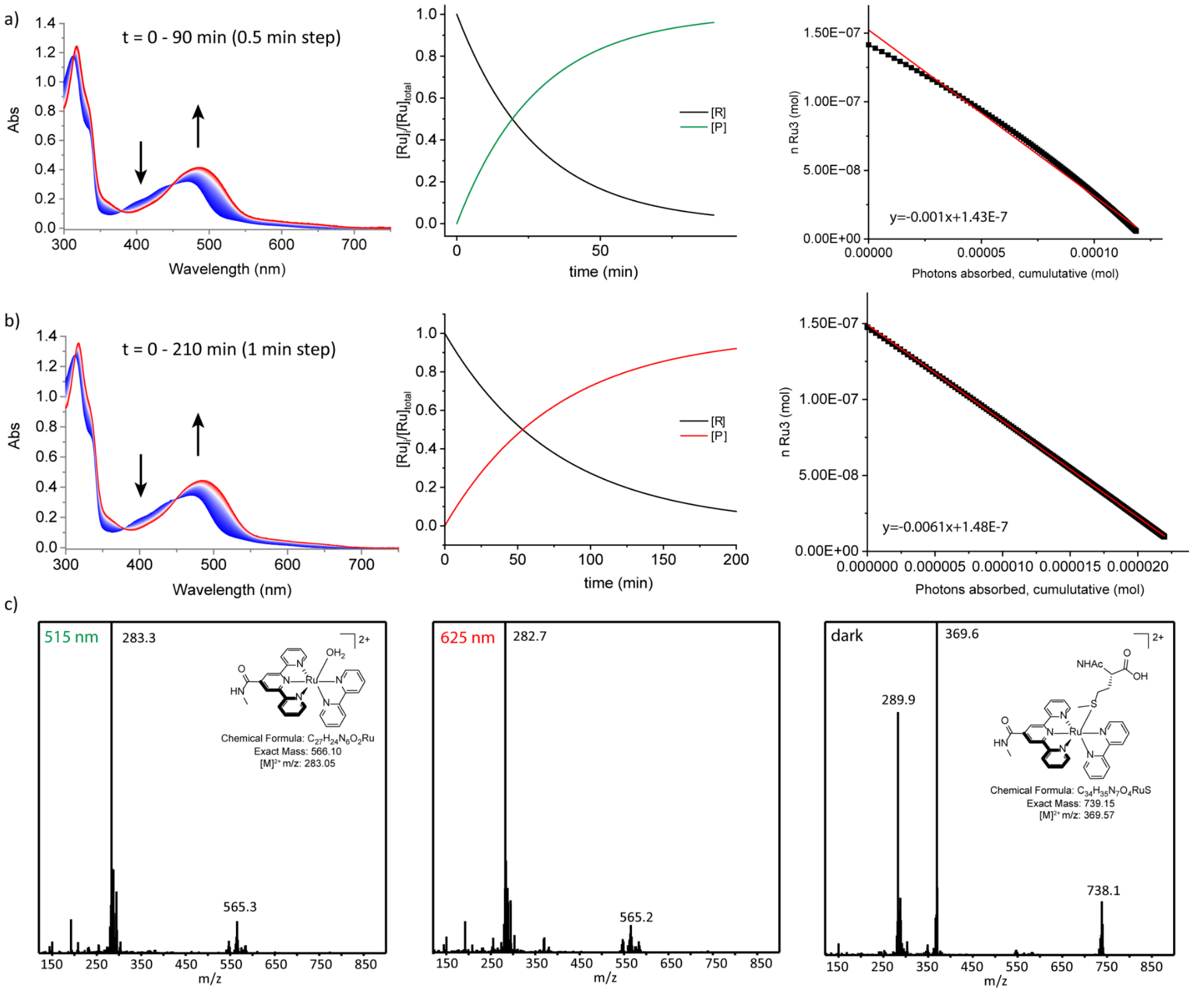

Figure S72: Investigation of the photosubstitution reactivity of [Ru3]Cl_2 in water under a) 515 nm and b) 625 nm light irradiation. From left to right are reported the absorption spectral change, the molar ratio change over time and the amount of initial reagent plotted against the cumulative amount of photons absorbed. The slopes are numerically equal to the opposite of the photosubstitution quantum yield. c) ESI MS spectra of a sample of the complex under green light irradiation, red light irradiation and kept in the dark for 24 h. The expected m/z for [Ru3]Cl_2 is 369.57, which can be observed in the sample kept in the dark, and the expected m/z of the correspondent aqua complex is 283.05.

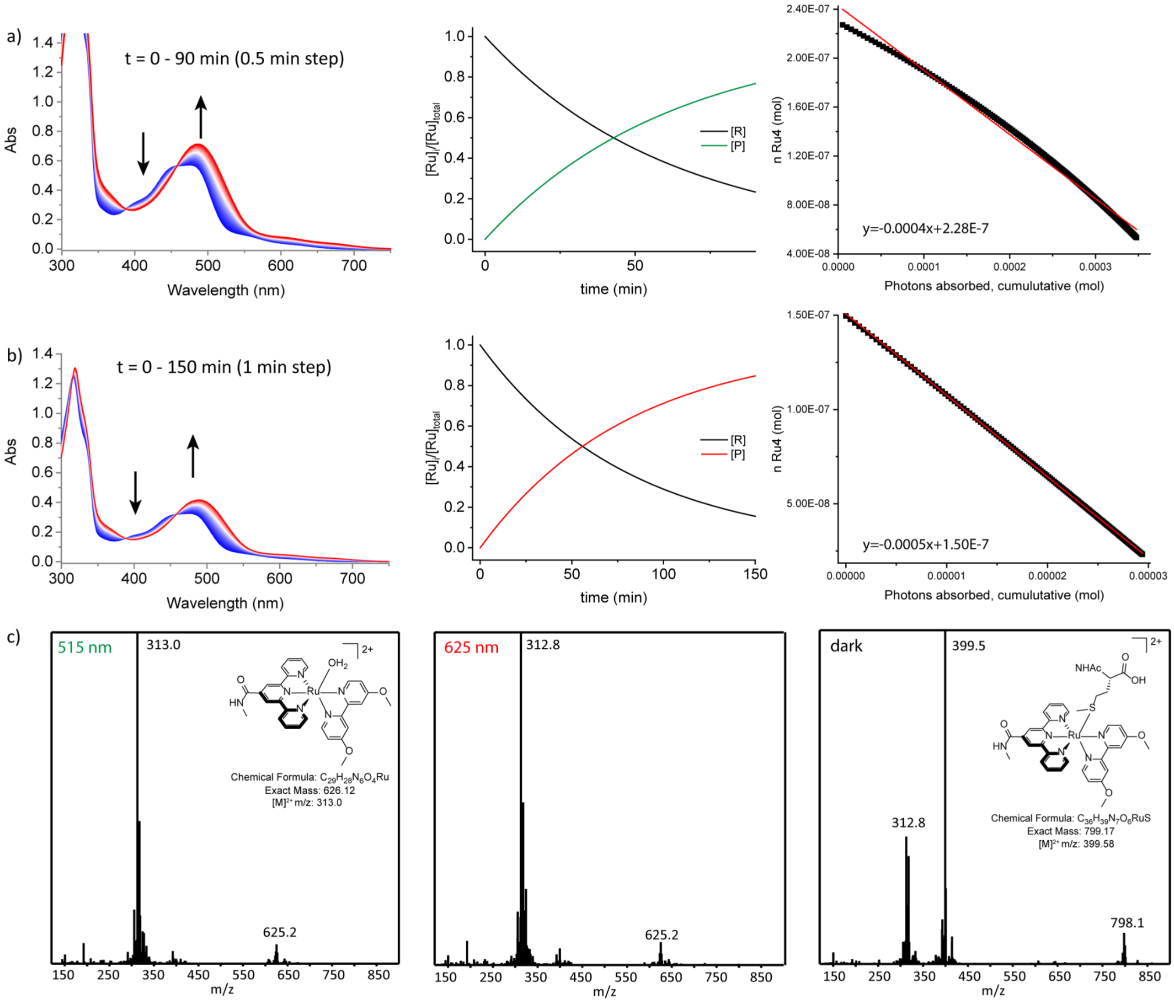

Figure S73: Investigation of the photosubstitution reactivity of [Ru4]Cl_2 in water under a) 515 nm and b) 625 nm light irradiation. From left to right are reported the absorption spectral change, the molar ratio change over time and the amount of initial reagent plotted against the cumulative amount of photons absorbed. The slopes are numerically equal to the opposite of the photosubstitution quantum yield. c) ESI MS spectra of a sample of the complex under green light irradiation, red light irradiation and kept in the dark for 24 h. The expected m/z for [Ru4]Cl_2 is 399.58, which can be observed in the sample kept in the dark, and the expected m/z of the correspondent aqua complex is 313.0.

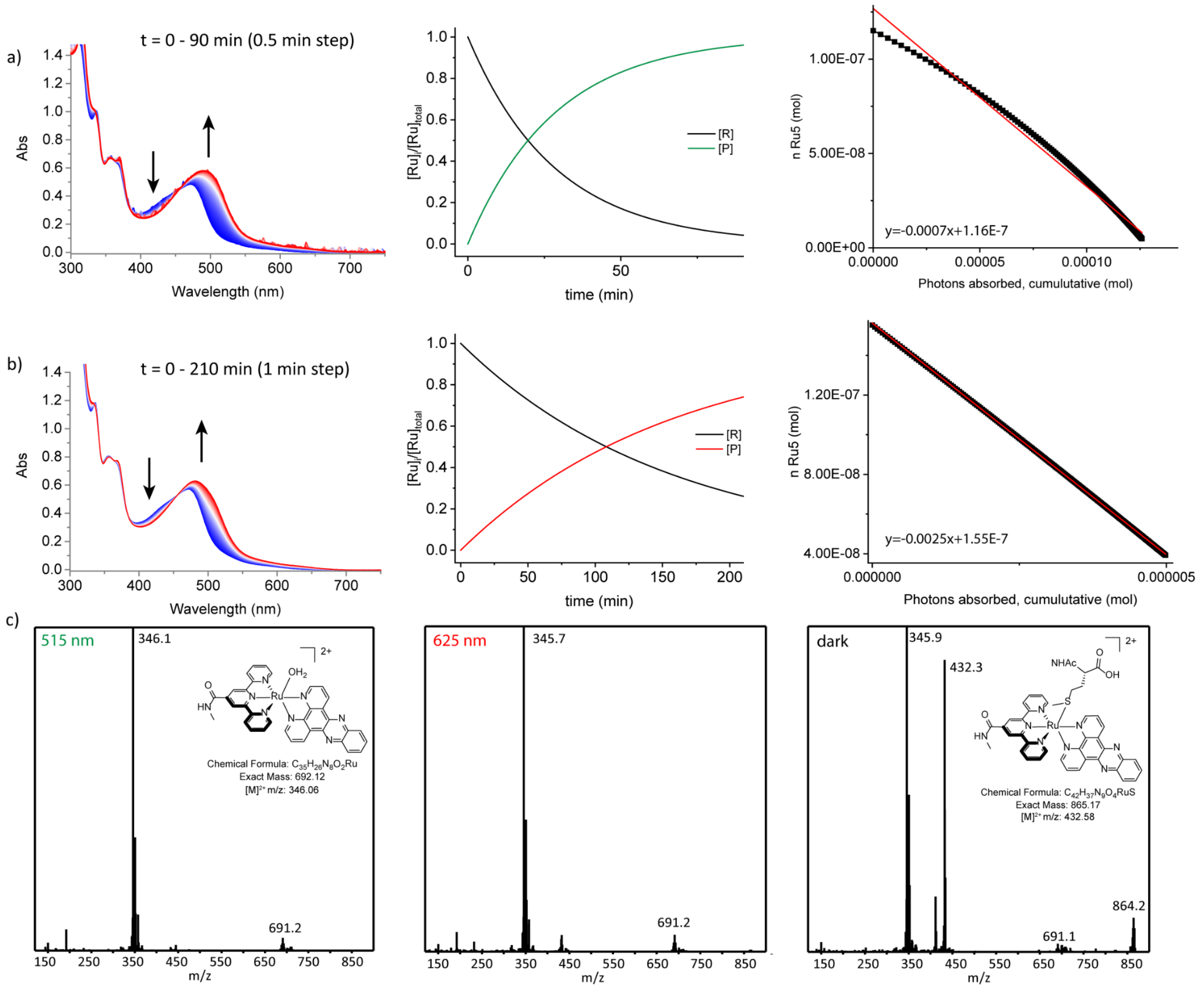

Figure S74: Investigation of the photosubstitution reactivity of [Ru5]Cl_2 in water under a) 515 nm and b) 625 nm light irradiation. From left to right are reported the absorption spectral change, the molar ratio change over time and the amount of initial reagent plotted against the cumulative amount of photons absorbed. The slopes are numerically equal to the opposite of the photosubstitution quantum yield. c) ESI MS spectra of a sample of the complex under green light irradiation, red light irradiation and kept in the dark for 24 h. The expected m/z for [Ru5]Cl_2 is 432.58, which can be observed in the sample kept in the dark, and the expected m/z of the correspondent aqua complex is 346.06.

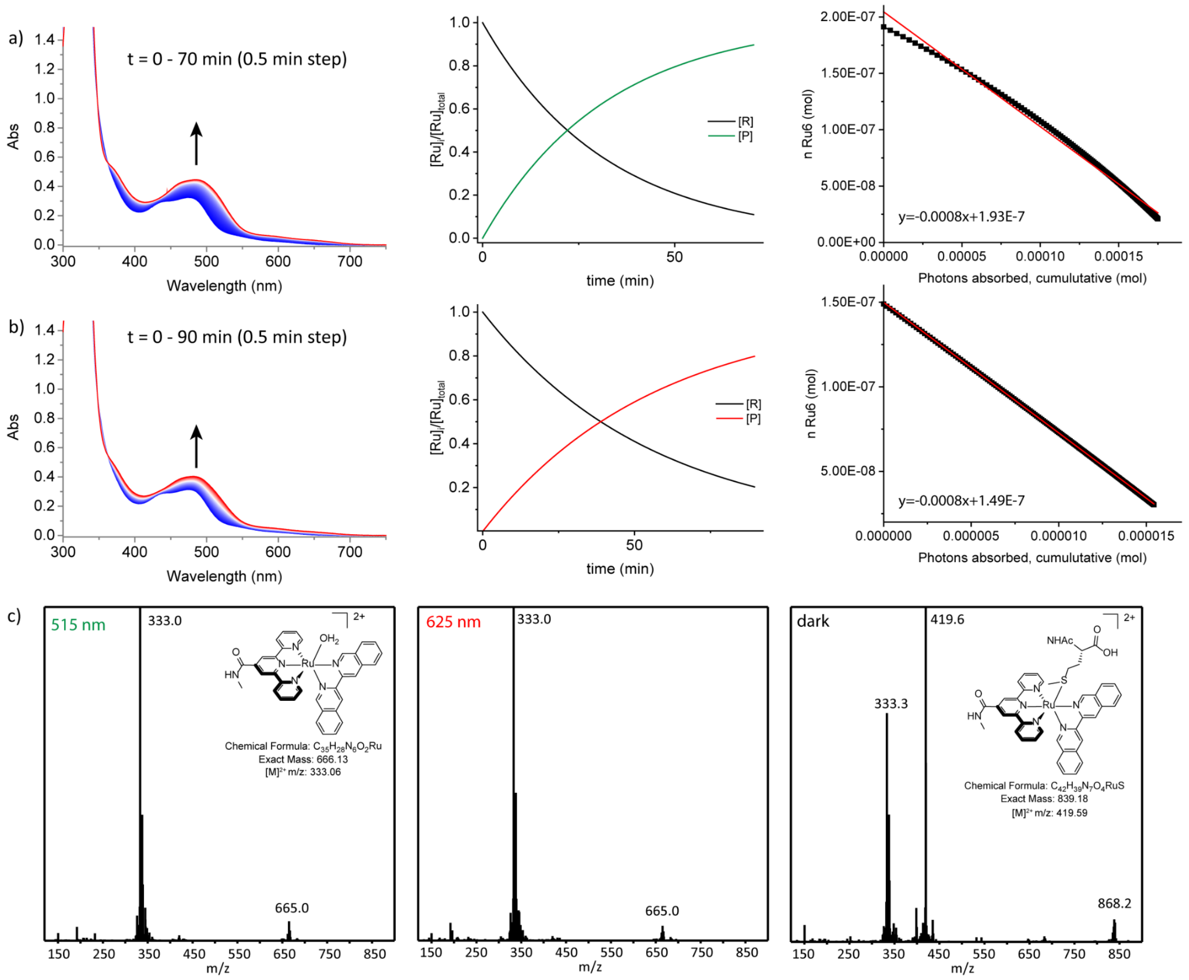

Figure S75: Investigation of the photosubstitution reactivity of $[Ru6]Cl_2$ in water under a) 515 nm and b) 625 nm light irradiation. From left to right are reported the absorption spectral change, the molar ratio change over time and the amount of initial reagent plotted against the cumulative amount of photons absorbed. The slopes are numerically equal to the opposite of the photosubstitution quantum yield. c) ESI MS spectra of a sample of the complex under green light irradiation, red light irradiation and kept in the dark for 24 h. The expected m/z for $[Ru6]Cl_2$ is 419.59, which can be observed in the sample kept in the dark, and the expected m/z of the correspondent aqua complex is 333.06.

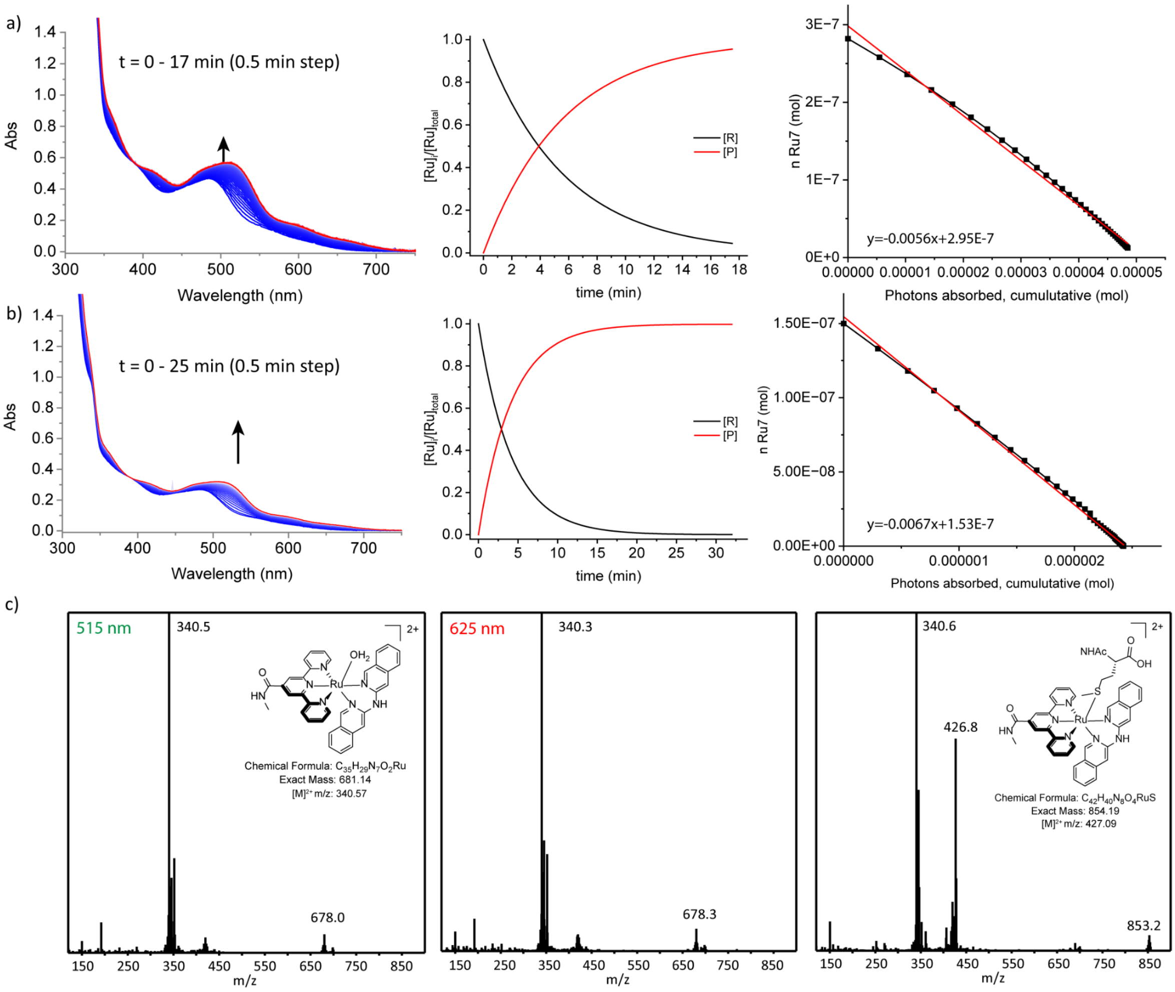

Figure S76: Investigation of the photosubstitution reactivity of $[Ru7]Cl_2$ in water under a) 515 nm and b) 625 nm light irradiation. From left to right are reported the absorption spectral change, the molar ratio change over time and the amount of initial reagent plotted against the cumulative amount of photons absorbed. The slopes are numerically equal to the opposite of the photosubstitution quantum yield. c) ESI MS spectra of a sample of the complex under green light irradiation, red light irradiation and kept in the dark for 24 h. The expected m/z for $[Ru7]Cl_2$ is 427.09, which can be observed in the sample kept in the dark, and the expected m/z of the correspondent aqua complex is 340.5.

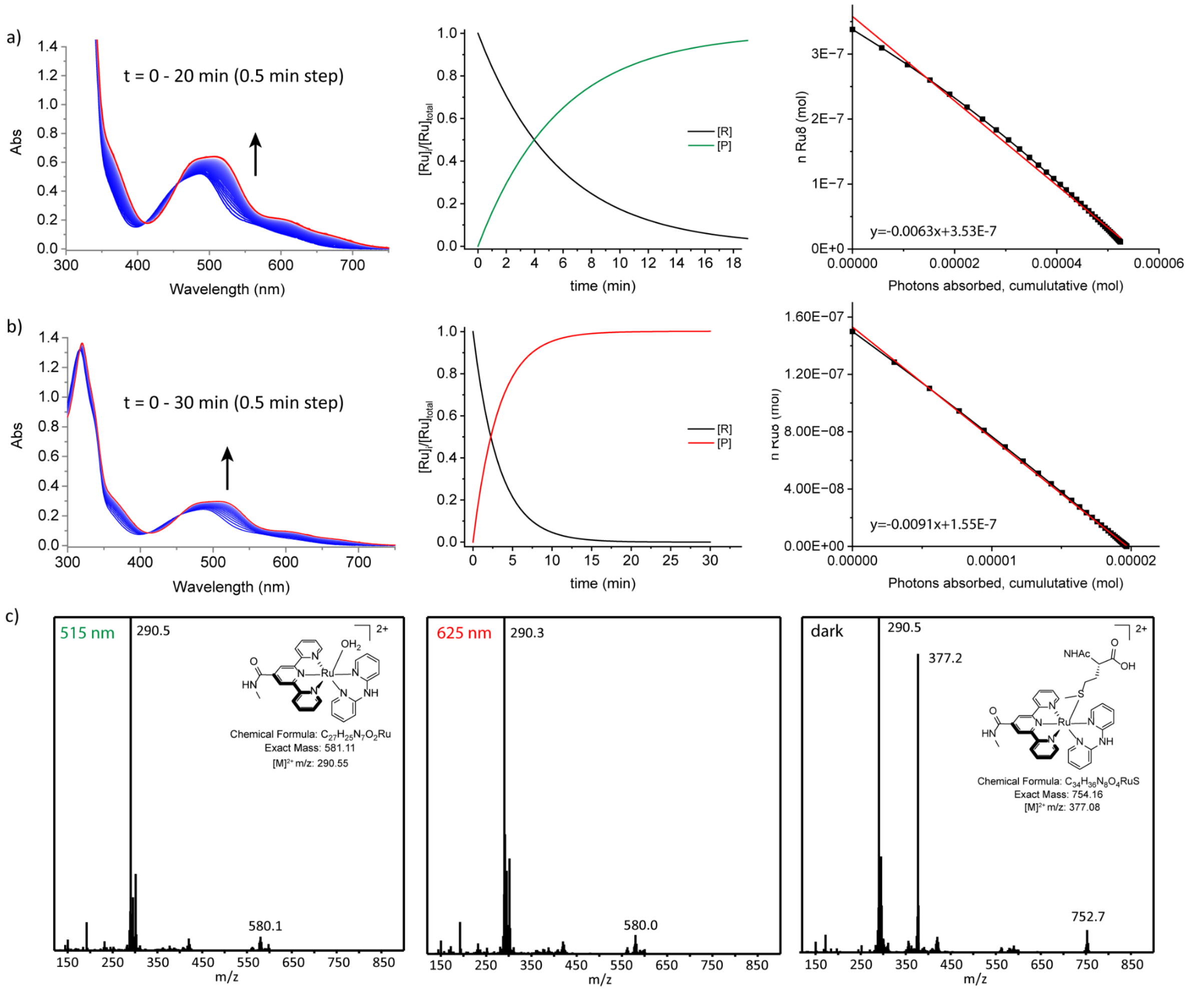

Figure S77: Investigation of the photosubstitution reactivity of $[Ru8]Cl_2$ in water under a) 515 nm and b) 625 nm light irradiation. From left to right are reported the absorption spectral change, the molar ratio change over time and the amount of initial reagent plotted against the cumulative amount of photons absorbed. The slopes are numerically equal to the opposite of the photosubstitution quantum yield. c) ESI MS spectra of a sample of the complex under green light irradiation, red light irradiation and kept in the dark for 24 h. The expected m/z for $[Ru8]Cl_2$ is 377.2, which can be observed in the sample kept in the dark, and the expected m/z of the correspondent aqua complex is 290.55.

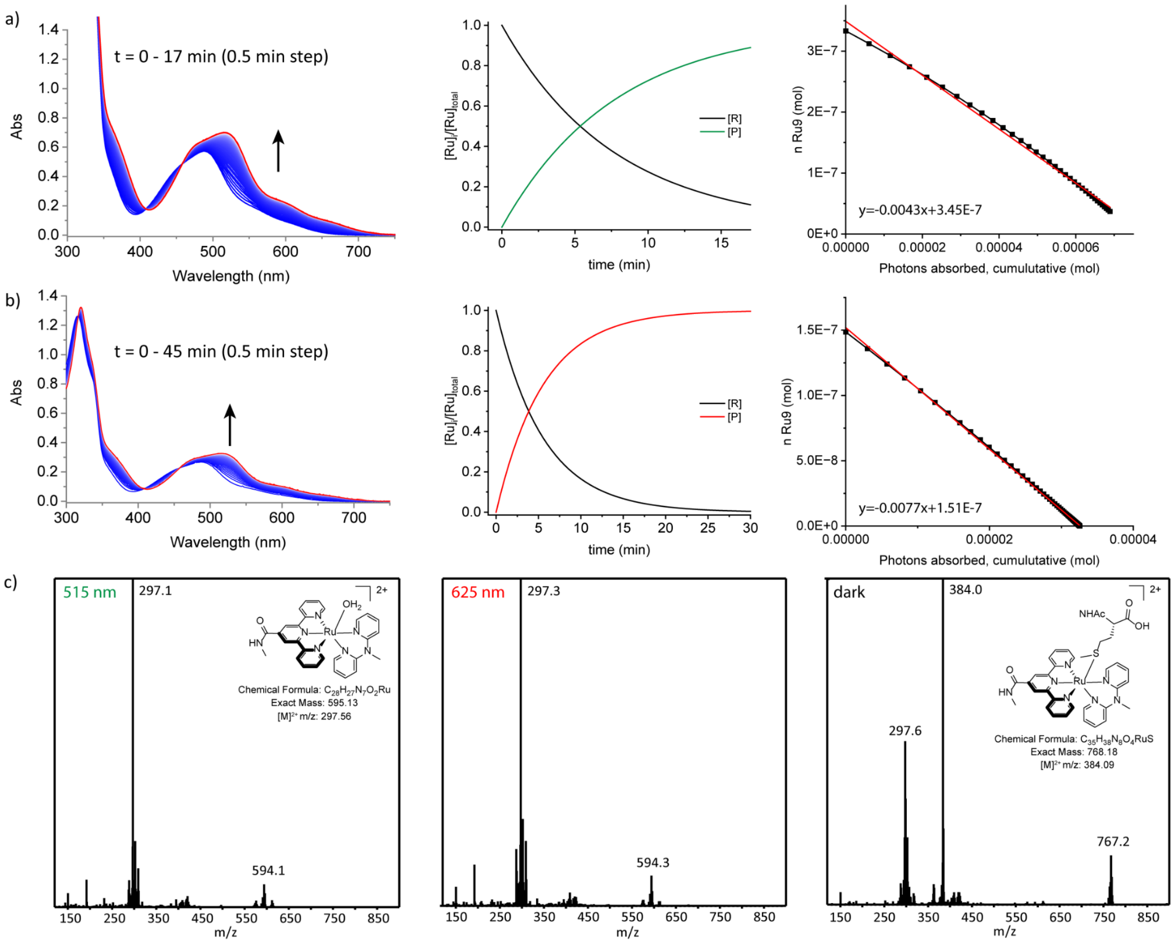

Figure S78: Investigation of the photosubstitution reactivity of [Ru9]Cl_2 in water under a) 515 nm and b) 625 nm light irradiation. From left to right are reported the absorption spectral change, the molar ratio change over time and the amount of initial reagent plotted against the cumulative amount of photons absorbed. The slopes are numerically equal to the opposite of the photosubstitution quantum yield. c) ESI MS spectra of a sample of the complex under green light irradiation, red light irradiation and kept in the dark for 24 h. The expected m/z for [Ru9]Cl_2 is 384.09, which can be observed in the sample kept in the dark, and the expected m/z of the correspondent aqua complex is 297.56.

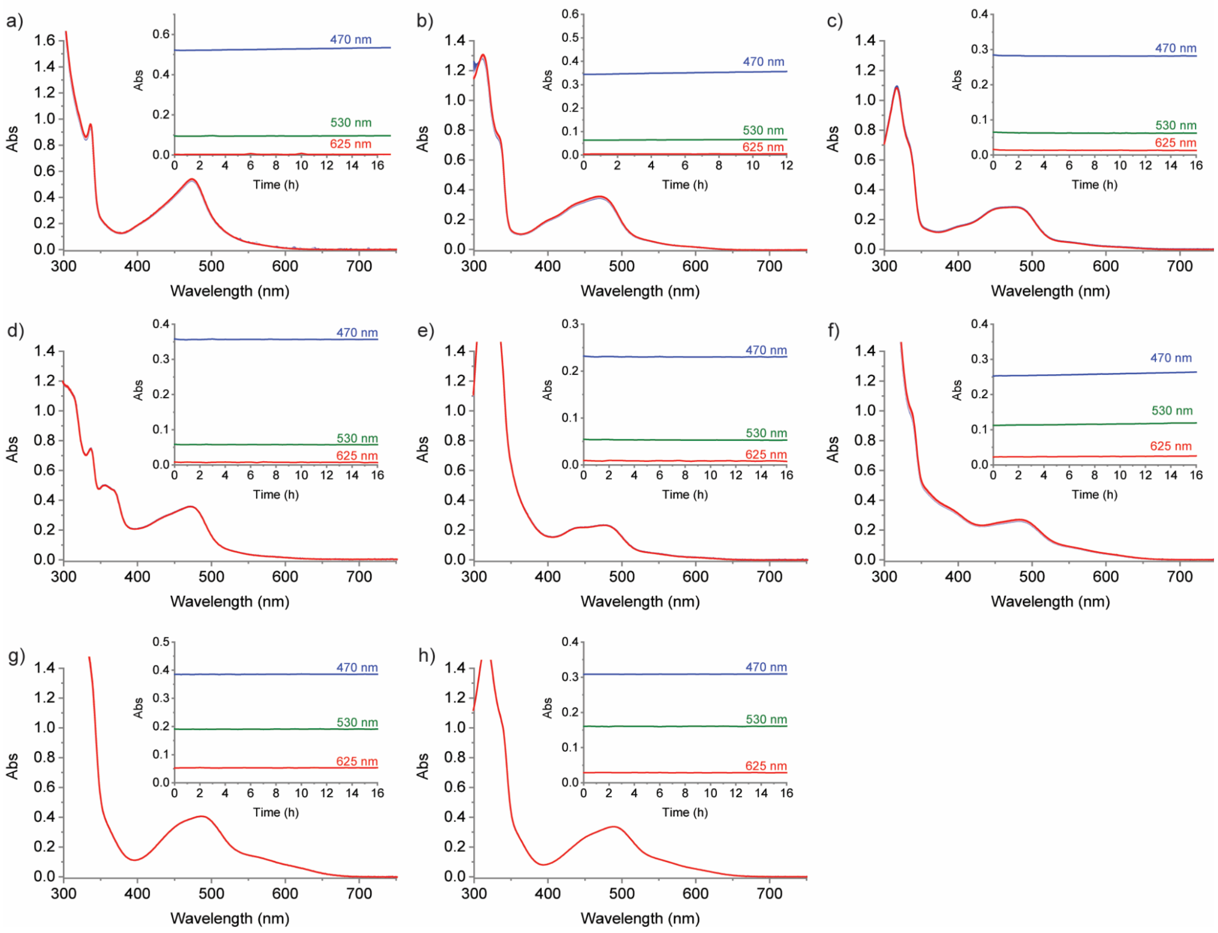

Figure S79: Time evolution of the UV-Vis absorption spectra of MilliQ solution of compound a) [Ru2]Cl_2, b) [Ru3]Cl_2, c) [Ru4]Cl_2, d) [Ru5]Cl_2, e) [Ru6]Cl_2, f) [Ru7]Cl_2, g) [Ru8]Cl_2, h) [Ru9]Cl_2, when kept in the dark at 298 K for 16 h. Spectra were measured every 30 min.

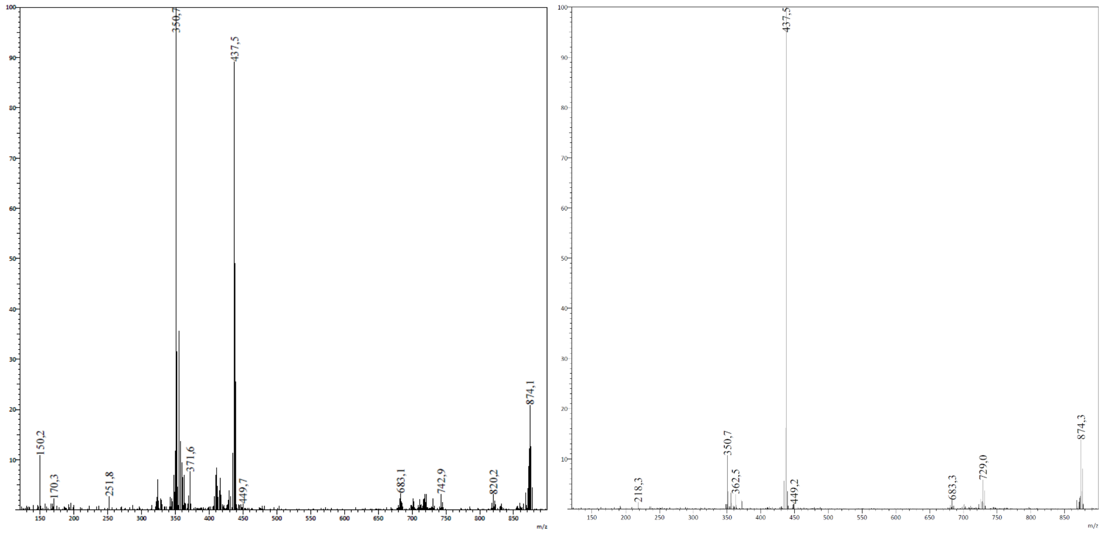

Figure S80: ESI-MS of [Ru2]Cl_2 in H_2O before (left) and after (right) UV-Vis dark stability test.

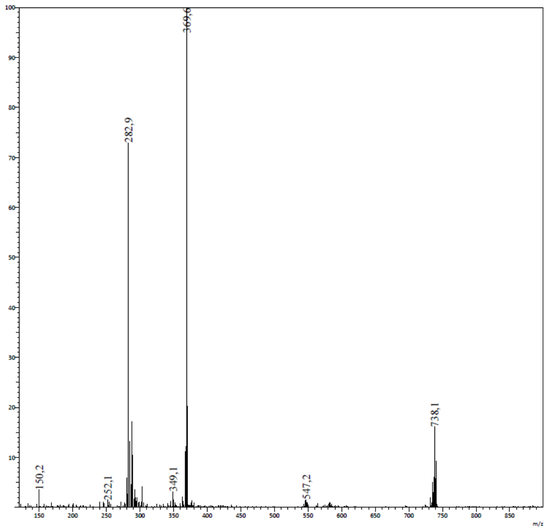

Figure S81: ESI-MS of [Ru3]Cl_2 in H_2O after UV-Vis dark stability test.

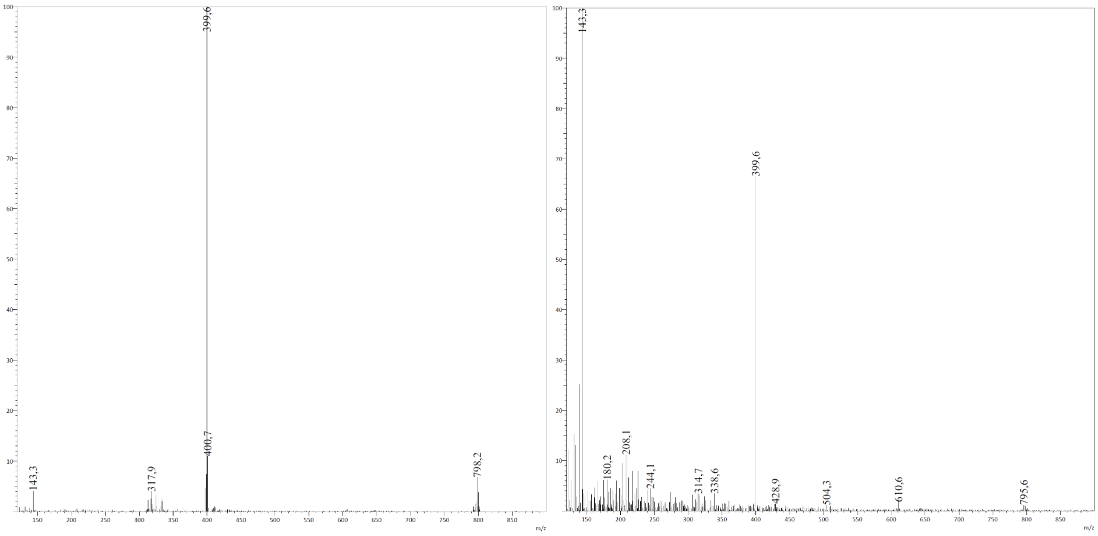

Figure S82: ESI-MS of [Ru4]Cl_2 in H_2O before (left) and after (right) UV-Vis dark stability test.

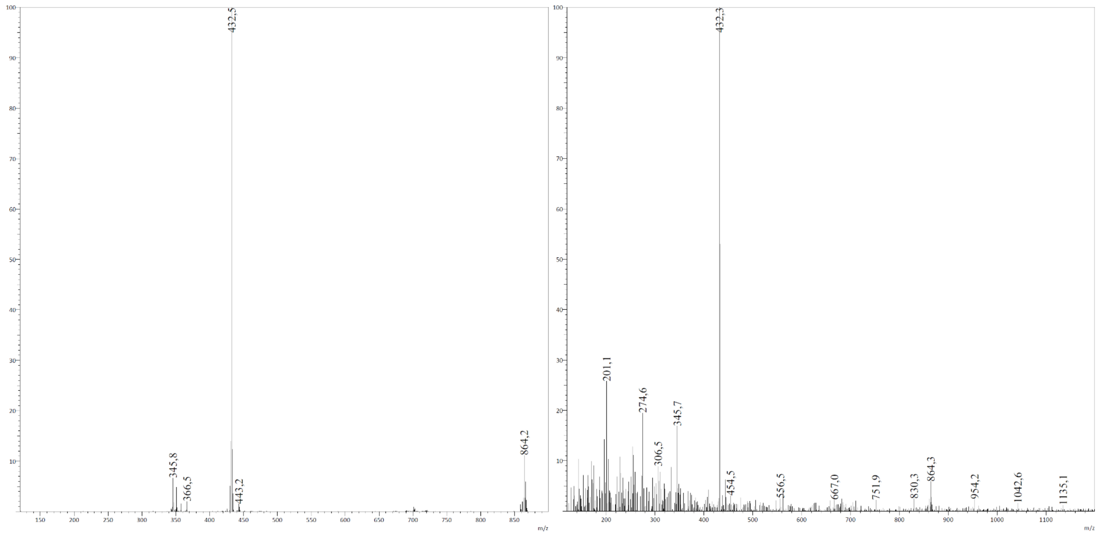

Figure S83: ESI-MS of [Ru5]Cl_2 in H_2O before (left) and after (right) UV-Vis dark stability test.

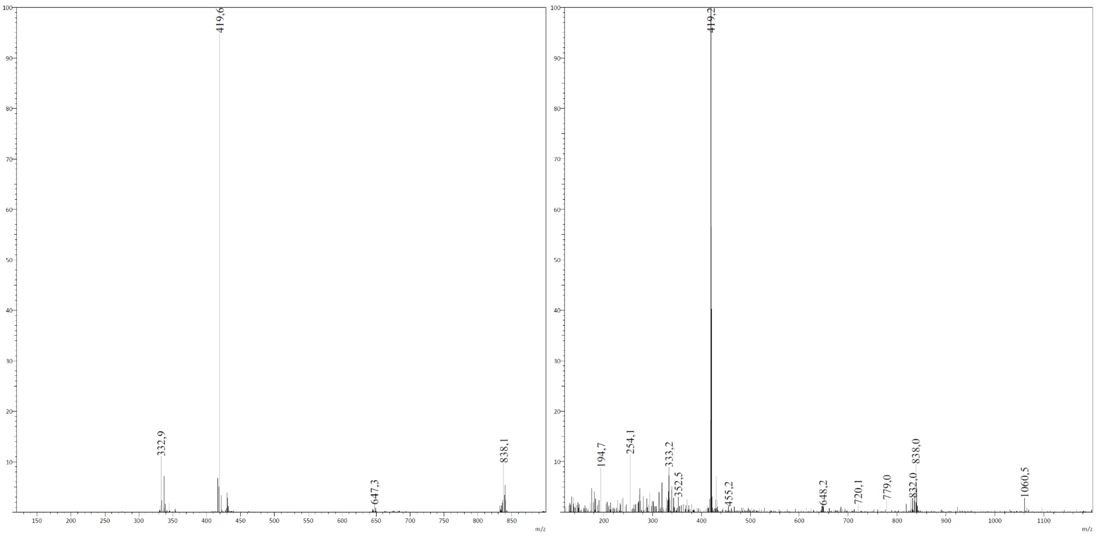

Figure S84: ESI-MS of $[Ru6]Cl_2$ in H_2O before (left) and after (right) UV-Vis dark stability test.

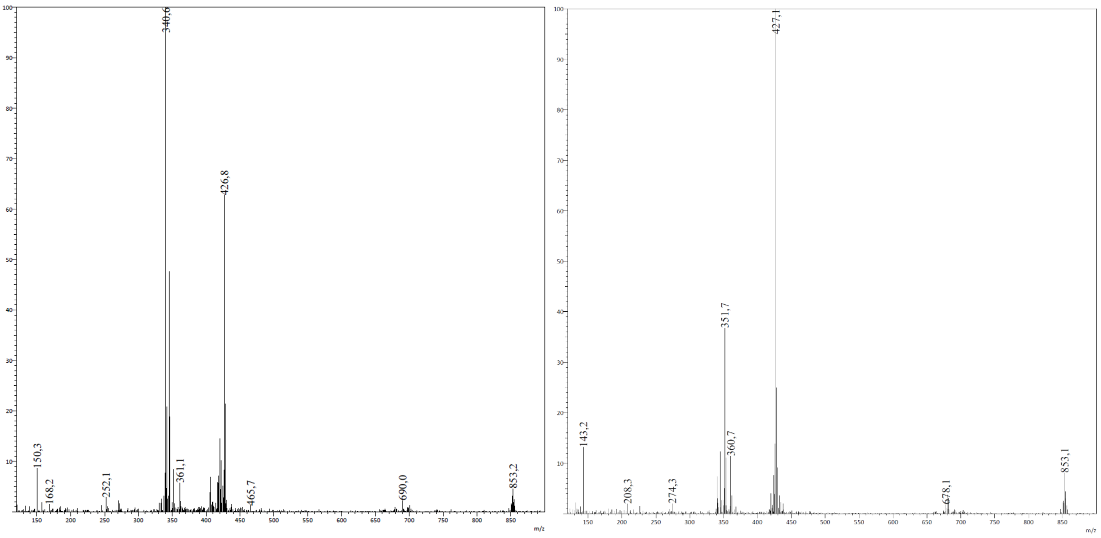

Figure S85: ESI-MS of $[Ru7]Cl_2$ in H_2O before (left) and after (right) UV-Vis dark stability test.

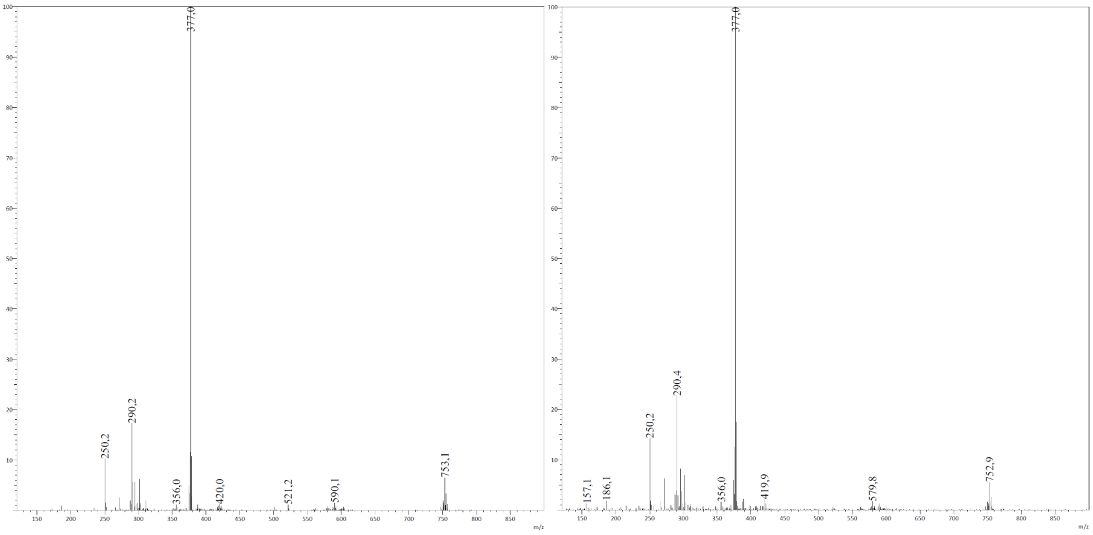

Figure S86: ESI-MS of $[Ru8]Cl_2$ in H_2O before (left) and after (right) UV-Vis dark stability test.

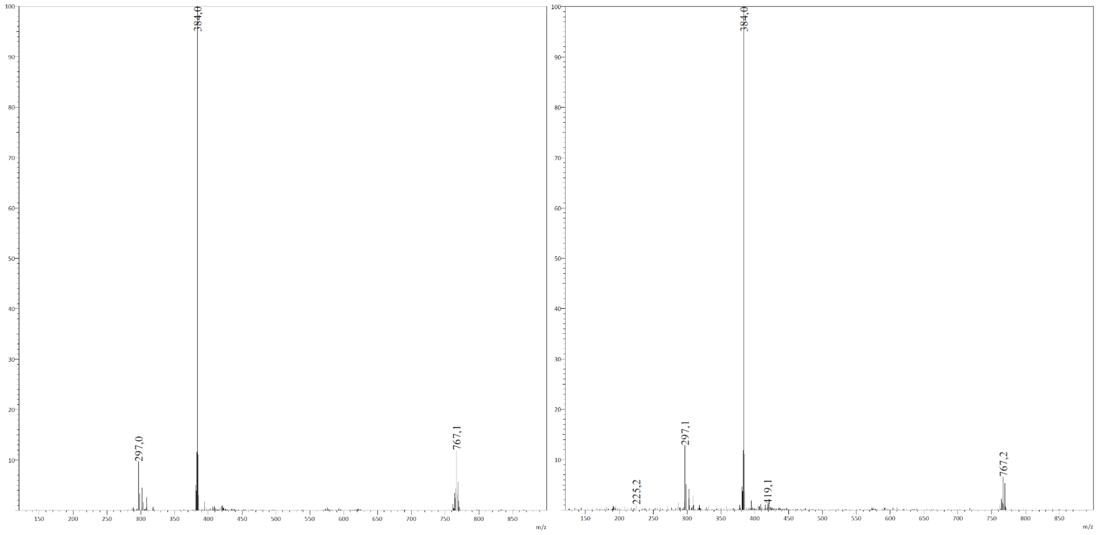

Figure S87: ESI-MS of $[Ru9]Cl_2$ in H_2O before (left) and after (right) UV-Vis dark stability test.

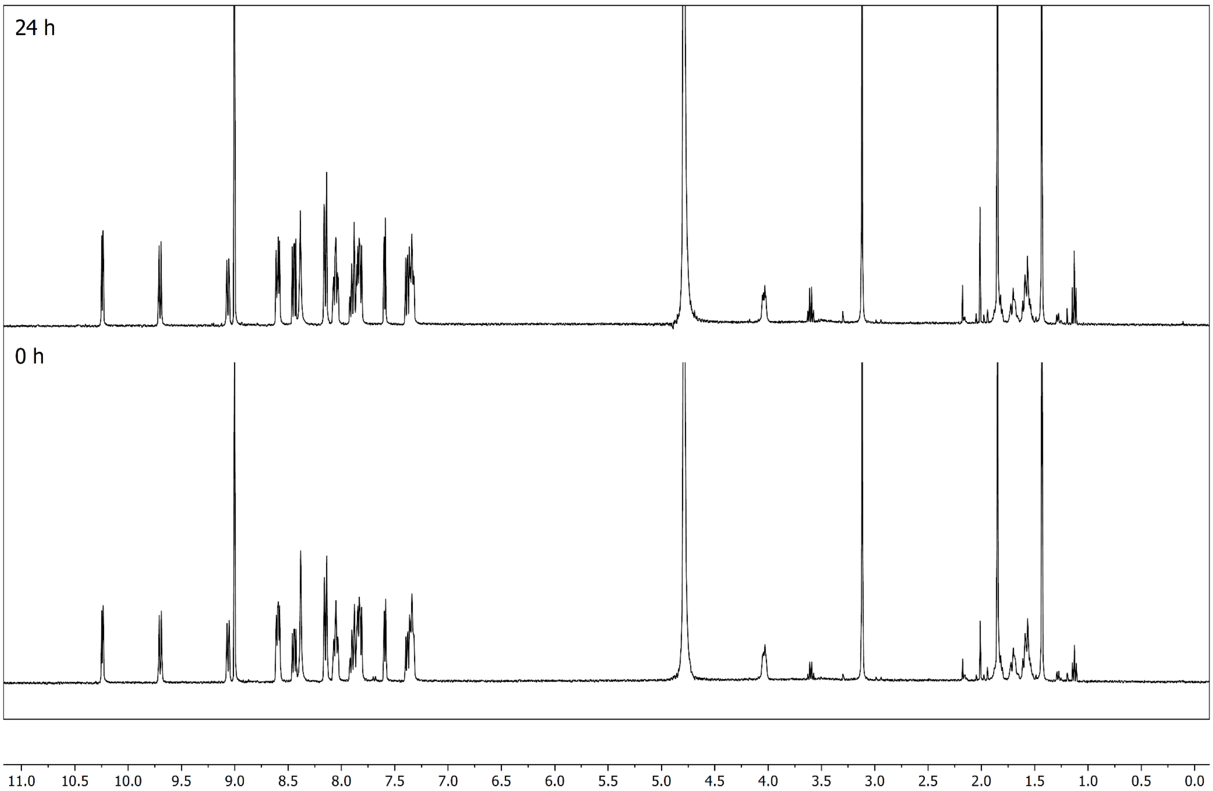

Figure S88: Evolution of ^1H NMR spectra of $[Ru5]Cl_2$ in D_2O in dark at 25 °C for 24 h. The data were collected at intervals of 0 and 24 h on Bruker Avance 400 MHz.

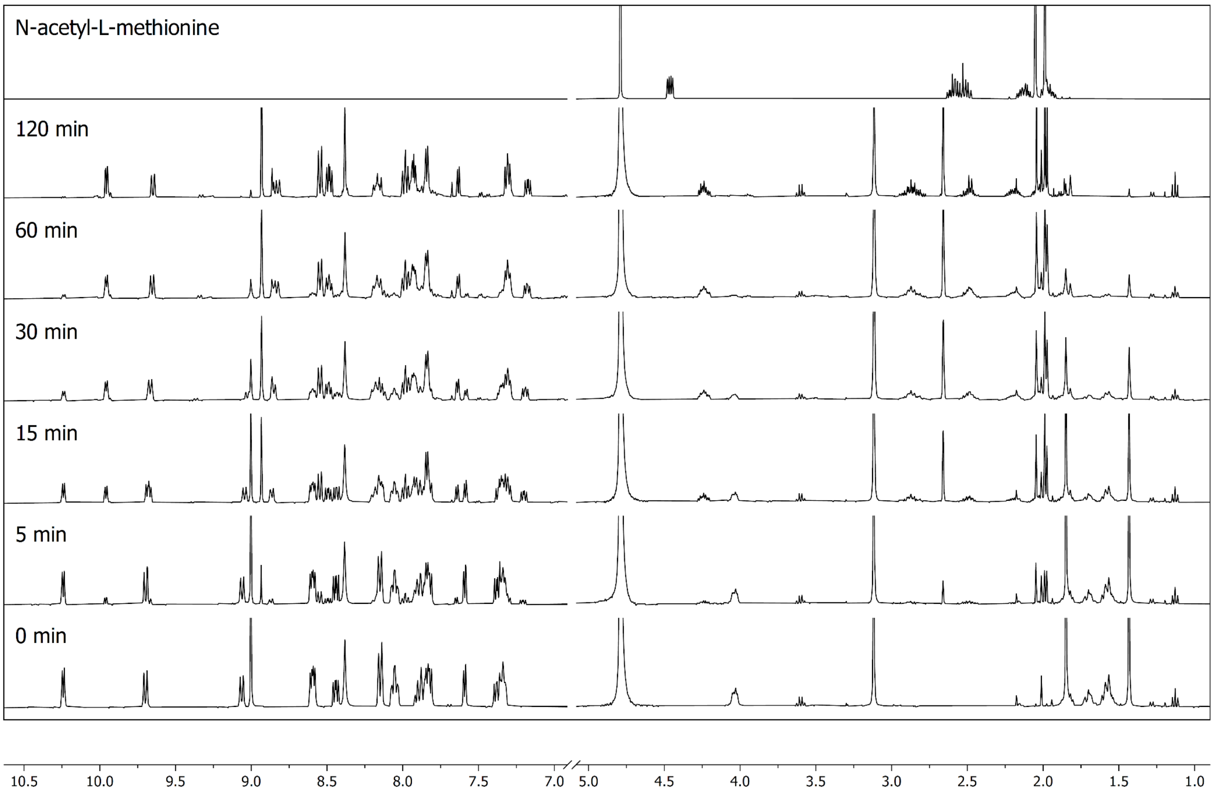

Figure S89: Evolution of ^1H NMR spectra of $[Ru5]Cl_2$ in D_2O upon irradiation with 530 nm green light at 25 °C for 2 h. The data were collected at intervals of 0, 5, 15, 30, 60, and 120 min on Bruker Avance 400 MHz and compared to spectrums of free *N*-acetyl-L-methionine.

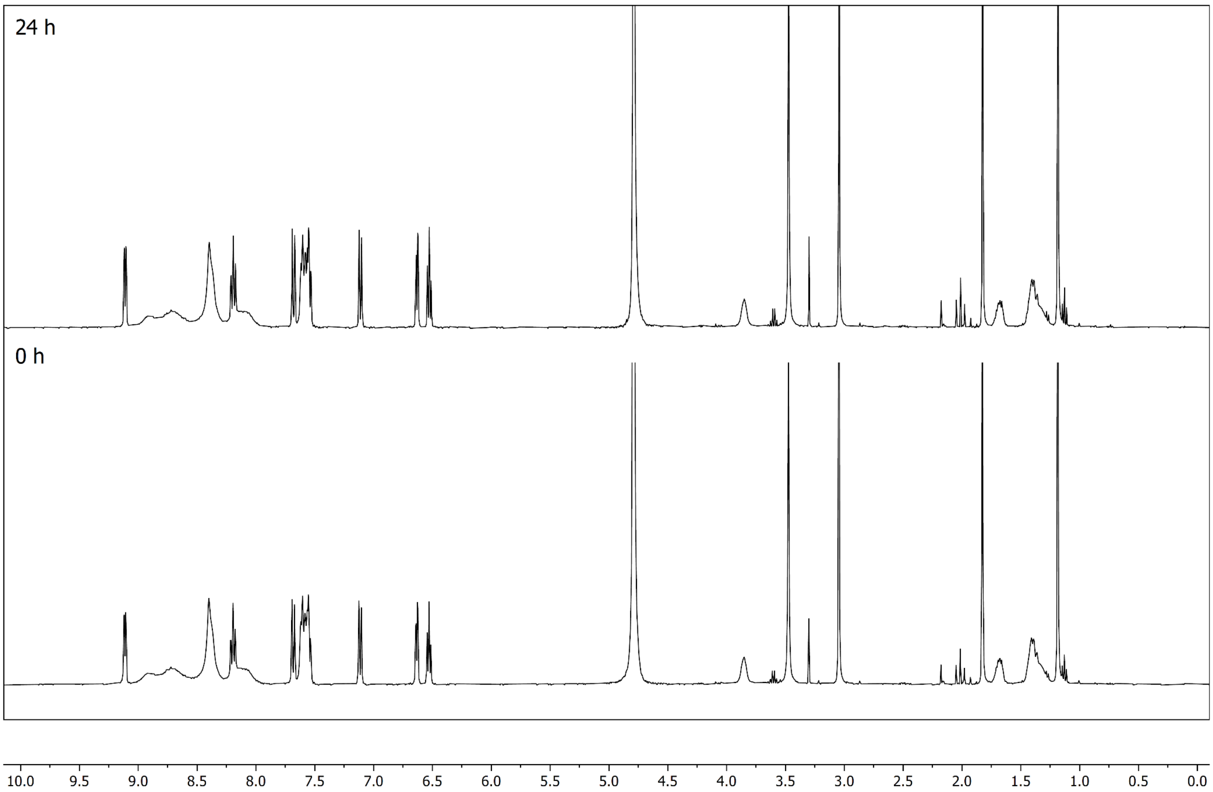

Figure S90: Evolution of ^{1}H NMR spectra of $[Ru9]Cl_2$ in D_2O in dark at 25 °C for 24 h. The data were collected at intervals of 0 and 24 h on Bruker Avance 400 MHz.

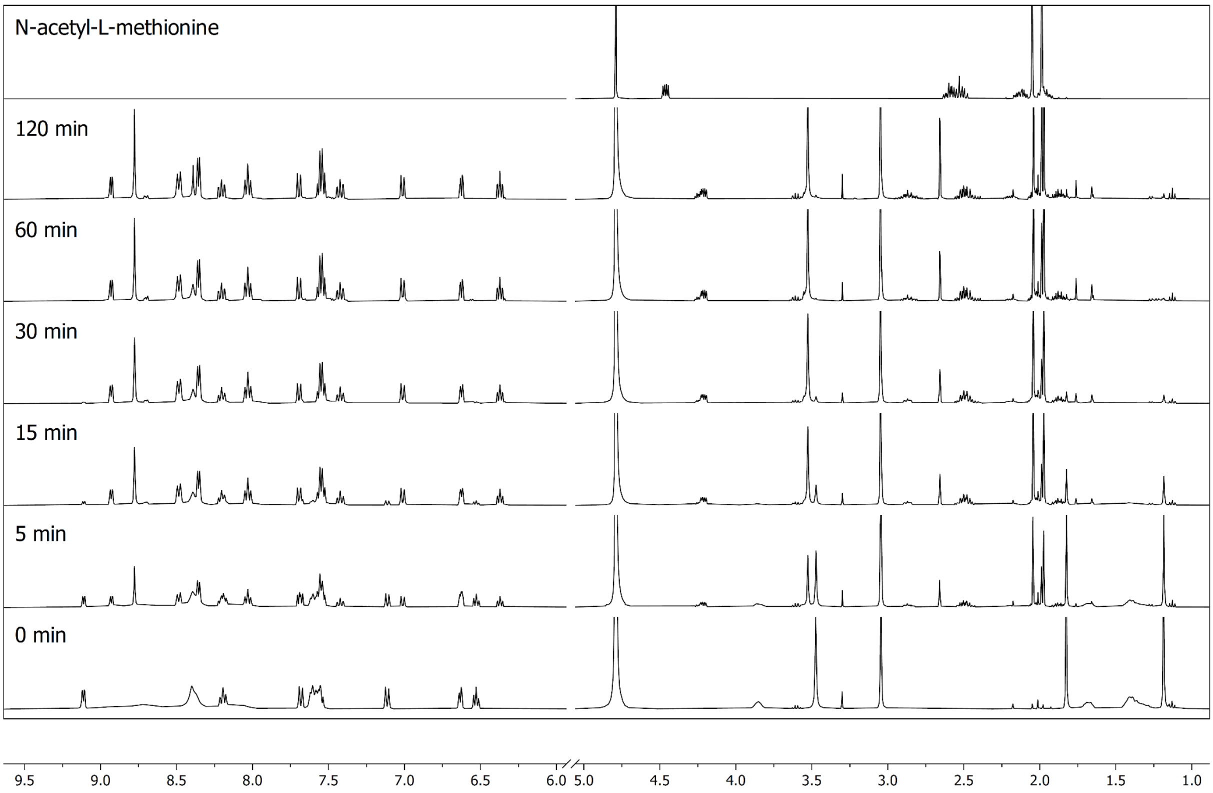

Figure S91: Evolution of ^{1}H NMR spectra of $[Ru9]Cl_2$ in D_2O upon irradiation with 530 nm green light at 25 °C for 2 h. The data were collected at intervals of 0, 5, 15, 30, 60, and 120 min on Bruker Avance 400 MHz and compared to spectrums of free *N*-acetyl-L-methionine.

4. [Ru(dpn)(i-Hdiqa)(AcMet)]Cl_2 - [Ru10]Cl_2

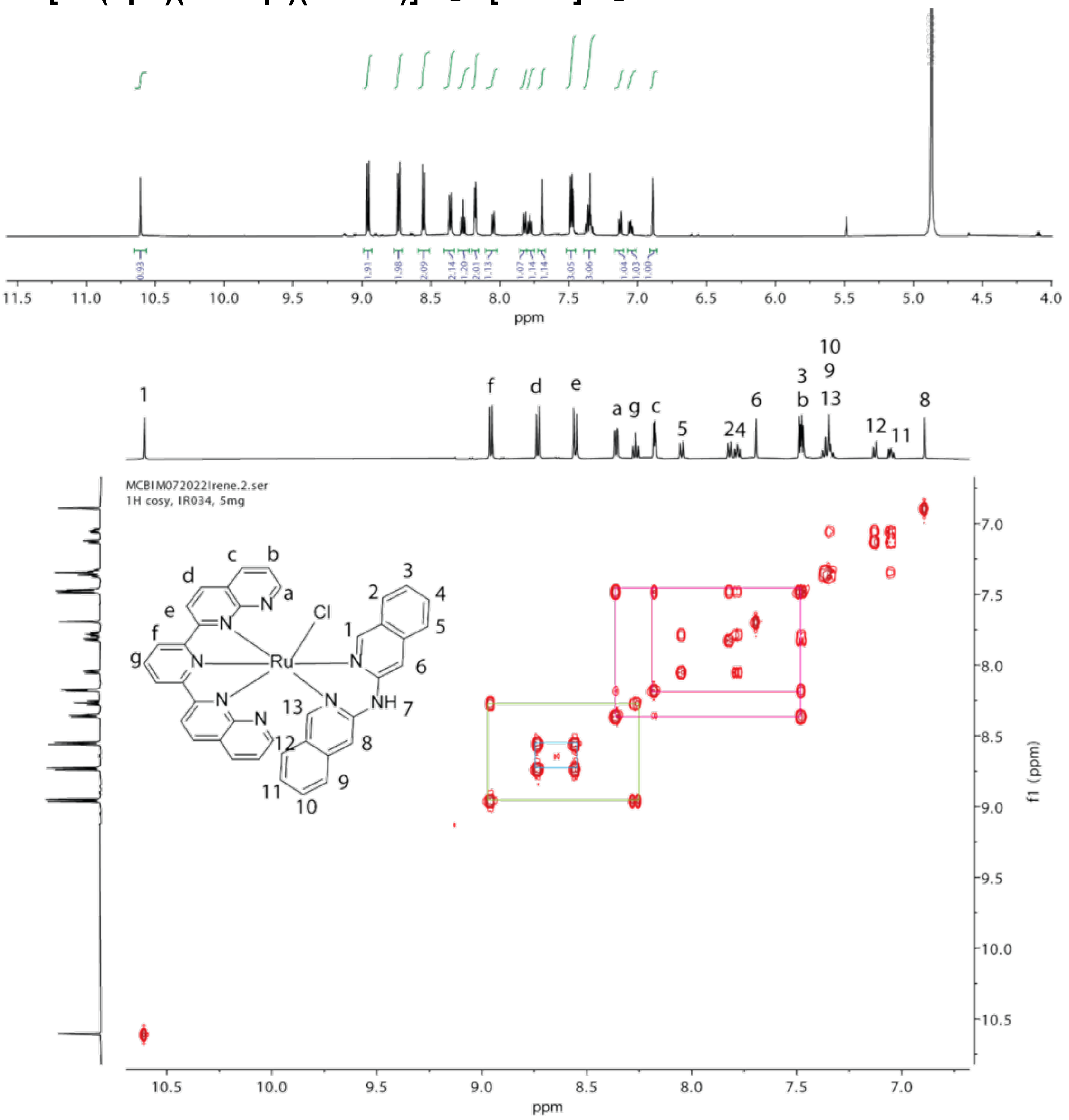

Figure S92: ^1H NMR and ^1H-^1H COSY NMR in MeOD with assignment for the complex [pRu10]Cl.

a) ESI-MS

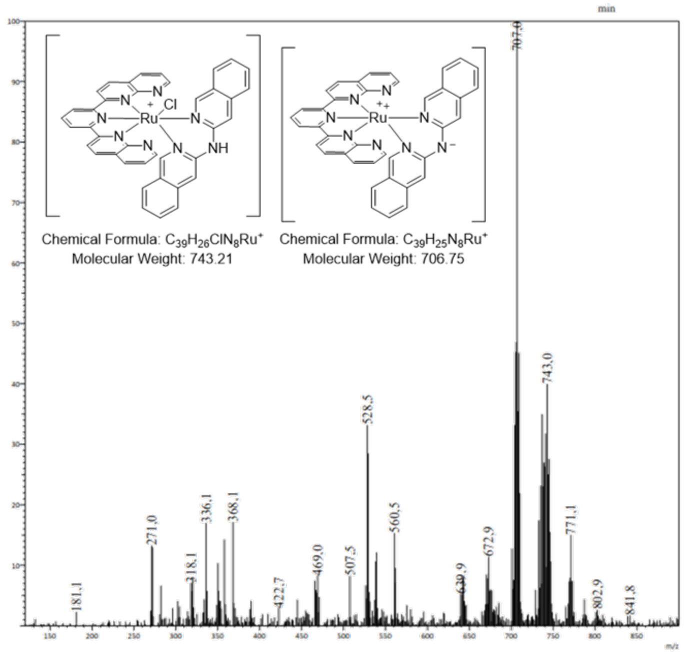

b) ^1H NMR MeOD

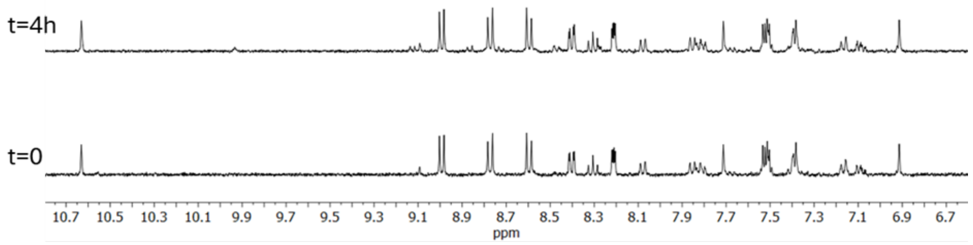

Figure S93: Thermal stability of [pRu10]Cl. a) ESI-MS spectrum where the highest intensity peak is assigned to the complex without the Cl- ligand. b) ^1H NMR in MeOD of a solution of [pRu10]Cl readily prepared and after 4 h. It is possible to observe the appearance of small peaks indicating the poor stability of the mentioned compound.

a)

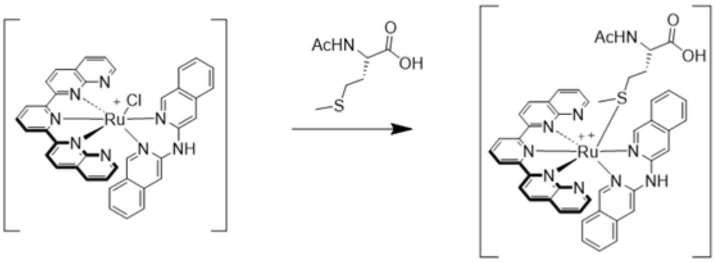

b)

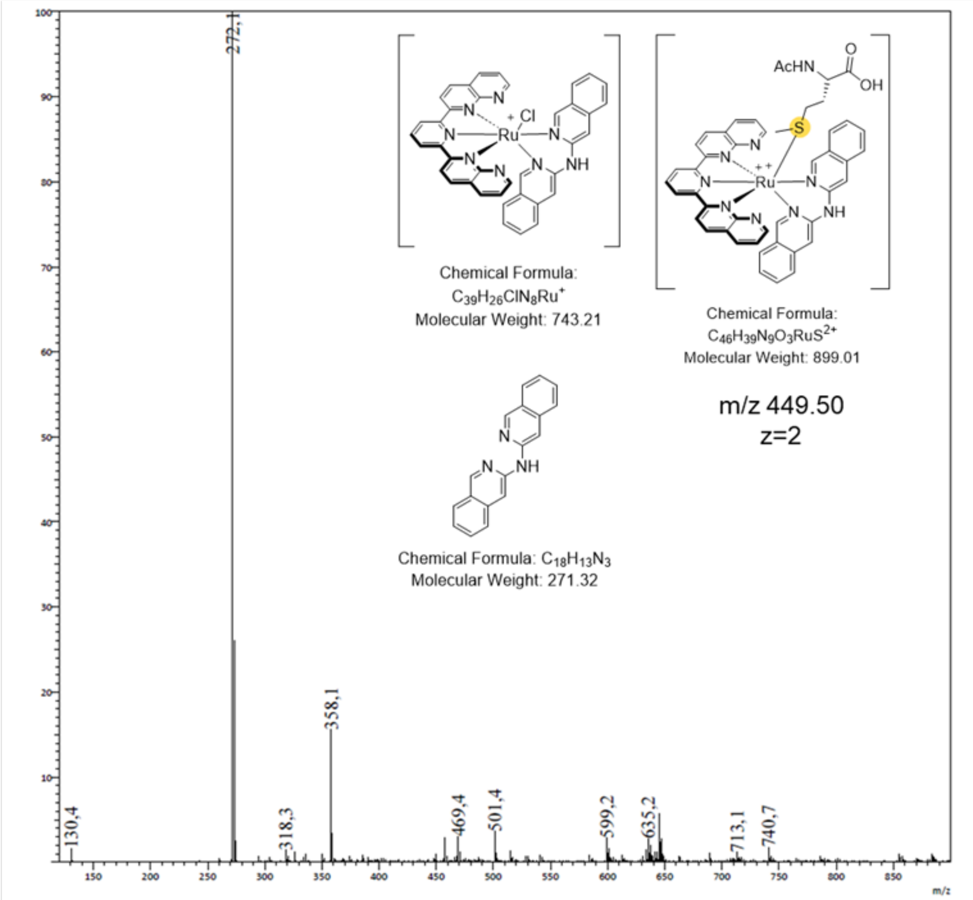

Figure S94: Synthesis attempt of [Ru10]Cl_2. a) Reaction scheme. b) ESI-MS of the crude mixture. The highest peak is assigned to the ligand iHdiqa, supporting the decomposition of the complex rather than the formation of the desired compound.

5. Single Crystal X-ray Crystallography

Single crystals suitable for X-ray crystallography were grown at room temperature by slow vapor diffusion of isopropyl ether into a methanol solution of [Ru6]$(PF_6)_2$.

All reflection intensities were measured at 110(2) K using a SuperNova diffractometer (equipped with Atlas detector) with Cu Kα radiation (λ = 1.54178 Å) under the program CrysAlisPro (Version CrysAlisPro 1.171.42.49, Rigaku OD, 2022). The same program was used to refine the cell dimensions and for data reduction. The structure was solved with the program SHELXS-2018/3[11] and was refined on F^2 with SHELXL-2018/3 (Sheldrick, 2018). Analytical numeric absorption correction using a multifaceted crystal model was applied using CrysAlisPro. The temperature of the data collection was controlled using the system Cryojet (manufactured by Oxford Instruments). The H atoms were placed at calculated positions using the instructions AFIX 13, AFIX 23, AFIX 43, AFIX 87 or AFIX 137 with isotropic displacement parameters having values 1.2 or 1.5 Ueq of the attached C, N or O atoms.

The asymmetric unit contains one Ru complex, two PF_6^- counter-ions, and some amount of very disordered lattice solvent molecules (most likely MeOH). The moiety C45>C54 is disordered over two orientations, and the occupancy factor of the major component refines to 0.630(6). The two counter-ions are disordered over either two or three orientations, and all occupancy factors can be retrieved from the final cif. The contribution from the highly disordered lattice solvent molecules was removed from the final refinement using the SQUEEZE procedure in PLATON.[12]

Table S4. Crystallographic Data for [Ru6]$(PF_6)_2$

Compound	[Ru6]$(PF_6)_2$
CCDC number	2455211
Crystal data	
Chemical formula	$C_{42}H_{37}N_7O_4RuS\cdot 2(F_6P)$
M_r	1125.85
Crystal system, space group	Triclinic, *P*-1
Temperature (K)	110
a, *b*, *c* (Å)	12.1446 (4), 14.2415 (3), 15.1863 (5)
α, β, ɣ (°)	88.009 (2), 83.647 (3), 86.621 (2)
V ($Å^3$)	2604.86 (13)
Z	2
Radiation type	Cu *K*α
μ (mm^{-1})	4.18
Crystal size (mm)	0.33 × 0.25 × 0.05
Data collection	
Diffractometer	SuperNova, Dual, Cu at zero, Atlas
Absorption correction	Analytical<br>*CrysAlis PRO* 1.171.42.49 (Rigaku Oxford Diffraction, 2022)<br>Analytical numeric absorption correction using a multifaceted crystal model based on expressions derived by R.C. Clark & J.S. Reid. (Clark, R. C. & Reid, J. S. (1995). Acta Cryst. A51, 887-897)<br>Empirical absorption correction using spherical harmonics, implemented in SCALE3 ABSPACK scaling algorithm.
T_{min}, T_{max}	0.384, 0.868
No. of measured, independent and observed [*I* > 2s(*I*)] reflections	35592, 9284, 7956
R_{int}	0.058
$(\sin \Theta/\lambda)_{max}$ ($Å^{-1}$)	0.598
Refinement	
$R[F^2 > 2\sigma(F^2)]$, $wR(F^2)$, *S*	0.071, 0.203 1.09
No. of reflections	9284
No. of parameters	893
No. of restraints	1035
H-atom treatment	H-atom parameters constrained

$\Delta\rho_{max}$, $\Delta\rho_{min}$ (e Å^{-3})	0.89, -0.75

Computer programs: *CrysAlis PRO* 1.171.42.49 (Rigaku OD, 2022), *SHELXS2018*/3 (Sheldrick, 2018), *SHELXL2018*/3 (Sheldrick, 2018), *SHELXTL* v6.10 (Sheldrick, 2008).

6. Computational Methods

Confidence interval linear regression[13]

Given a linear regression model where:

$$\hat{y}=\beta_0+\beta_1 x \quad \text{(S1)}$$

The 95% confidence interval for the predicted mean response at a specific point $x=x_0$ is:

$$\hat{y}(x_0) \pm t_{\alpha/2,v} \cdot s \cdot \sqrt{\frac{1}{n}+\frac{(x_0\text{-}\bar{x})^2}{\sum_{i=1}^{n}(x_i\text{-}\bar{x})^2}} \quad \text{(S2)}$$

where:

- $\hat{y}(x_0)$ is the predicted value at (x_0)
- $t_{\alpha/2,v}$ is the critical value from the t-distribution for 95% confidence and $v=n\text{-}2$ degrees of freedom
- s standard error of the estimate computed as:

$$\sqrt{\frac{1}{n\text{-}2}+\sum_{i=1}^{n}(y_i\text{-}\hat{y}_i)^2} \quad \text{(S3)}$$

- $\bar{x}$ mean of the x-values
- n number of observations

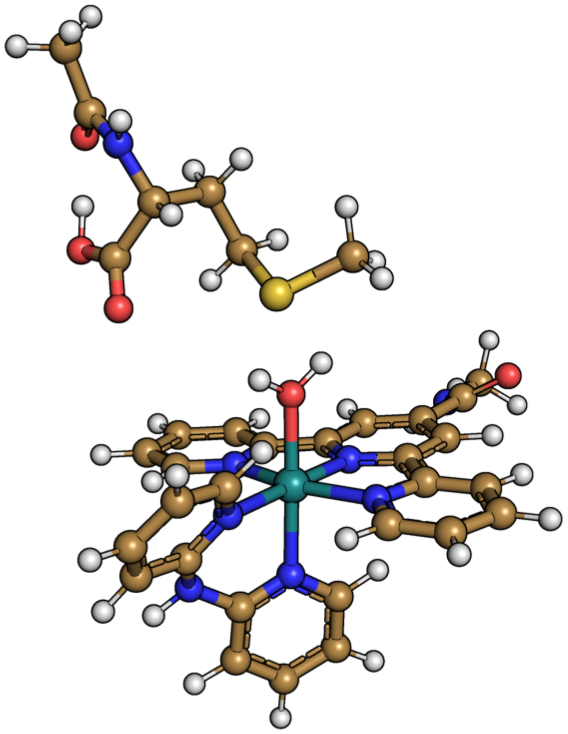

Figure S95: The configuration of compound $[Ru8]^{2+}$used to calculate the force energy differences between GFN1-xTB and DFT. All water molecules that were not directly coordinating or semi-coordinating with ruthenium are not visualized for clarity.

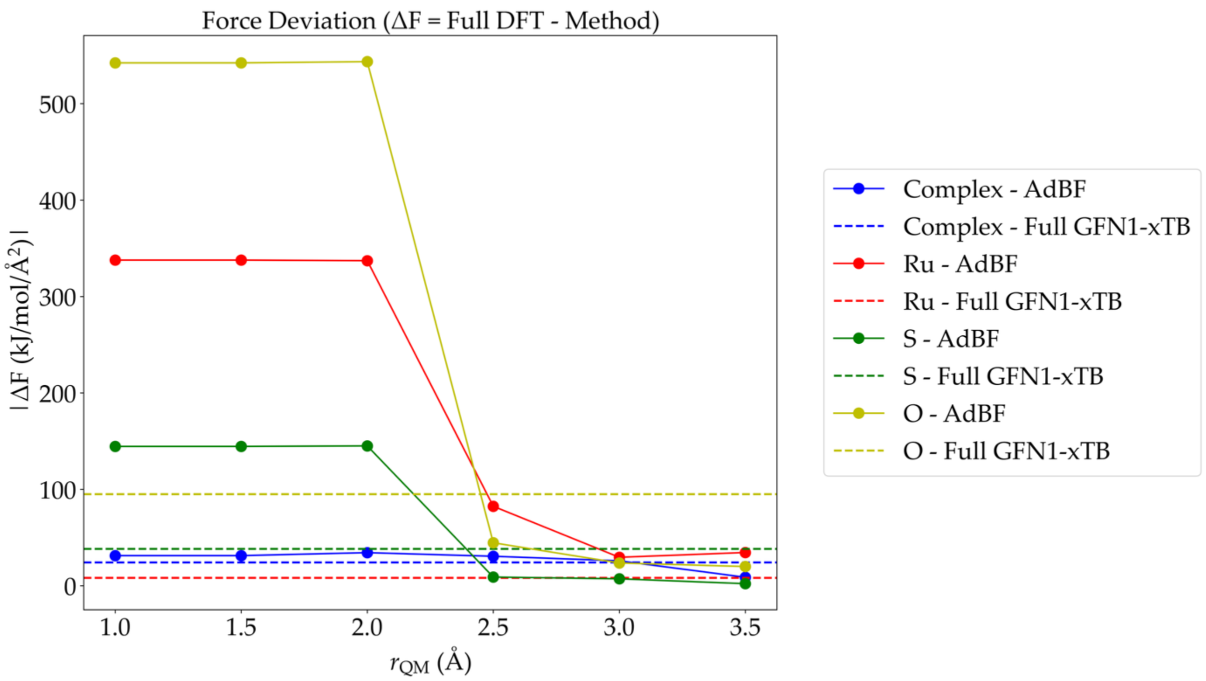

Figure S96: The force error for the parameters of the adaptive buffer method. Convergence of the force error of the GFN1-xTB adaptive buffer method can be seen around r_{QM} = 2.5 Å. The value chosen for this parameter was r_{QM} = 3.0 Å. All forces generated with the adaptive buffer method were comparable or lower than a full GFN1-xTB simulation as indicated with the x values. The r_{buffer} was set to 1.0 Å. Level of theory used for the DFT calculations was PBE[14]/DZVP-MOLOPT-GTH[15,16]/D3[17].

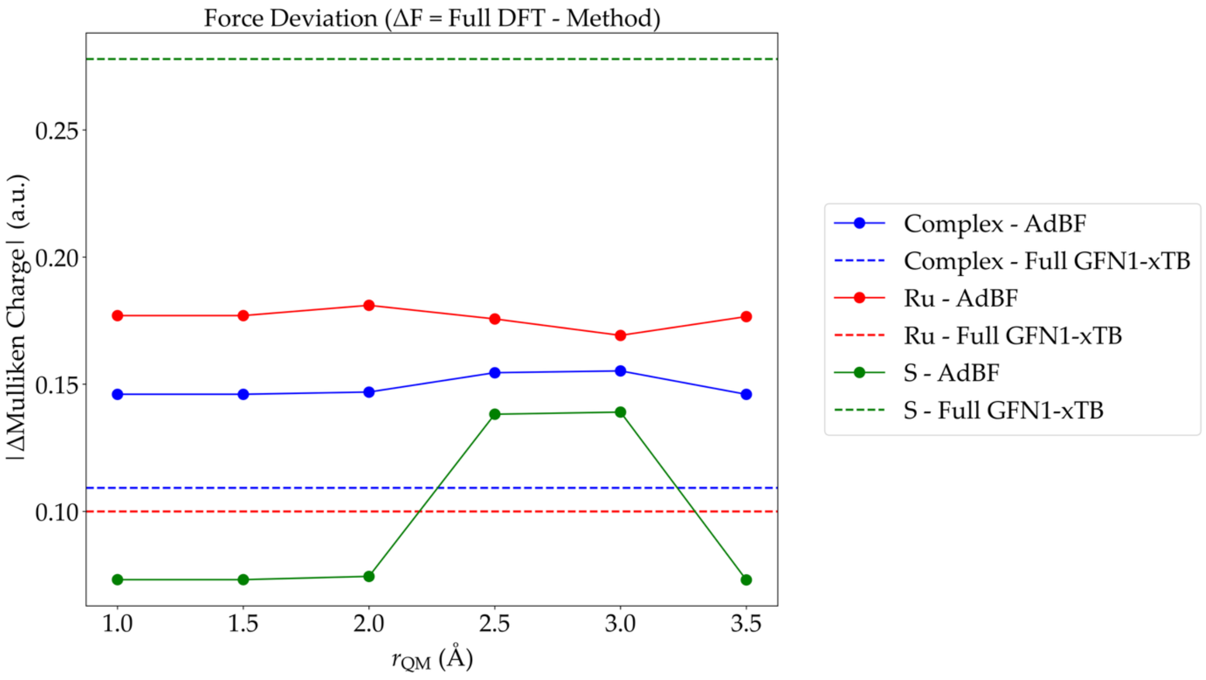

Figure S97: The Mulliken charge error for the parameters of the adaptive buffer method. The oxygen atom was not included as CP2K does not print the charges of the adaptive buffer by atom index, but by atom type making it impossible to differentiate between the oxygens in the dynamic QM zone. Level of theory used for the DFT calculations was PBE[14]/DZVP-MOLOPT-GTH[15,16]/D3[17].

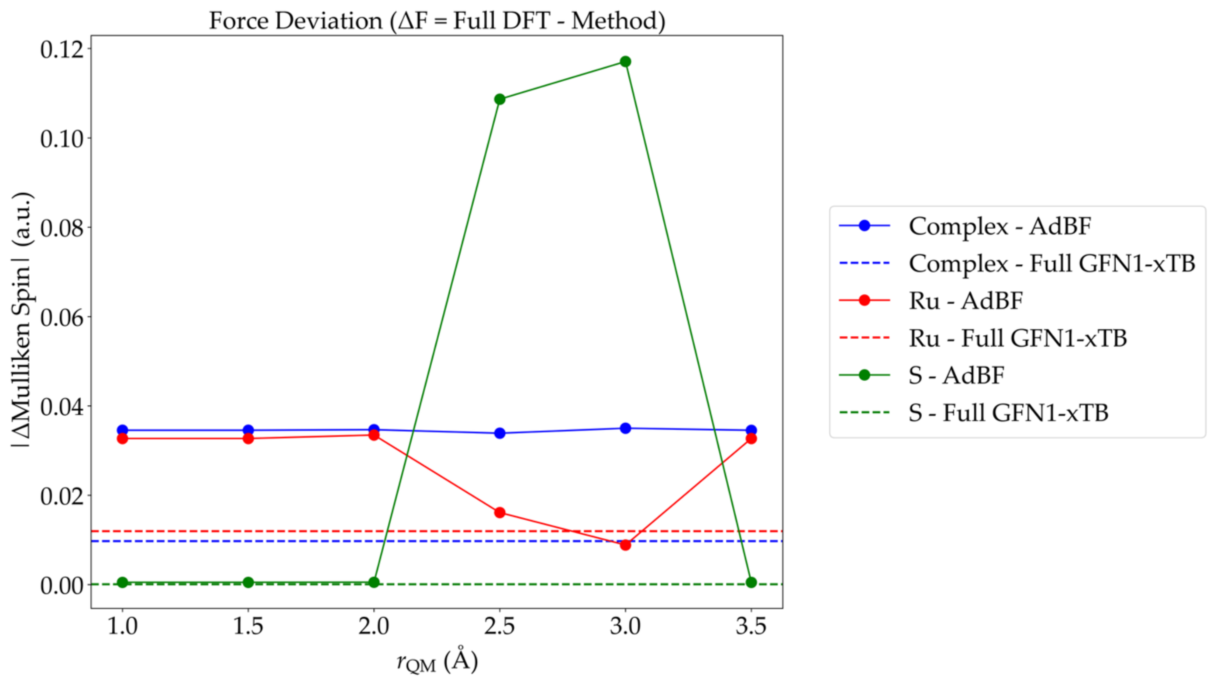

Figure S98: The Mulliken spin error for the parameters of the adaptive buffer method. The oxygen atom was not included as CP2K does not print the charges of the adaptive buffer by atom index, but by atom type making it impossible to differentiate between the oxygens in the dynamic QM zone. Level of theory used for the DFT calculation was PBE[14]/DZVP-MOLOPT-GTH[15,16]/D3[17].

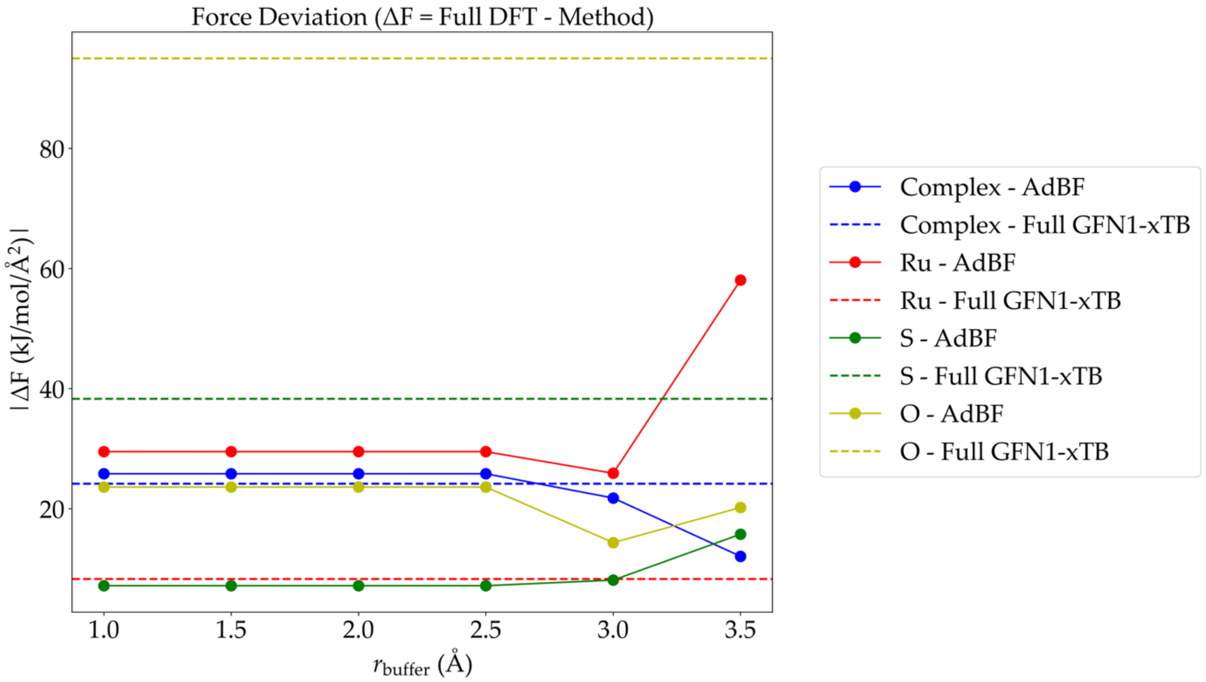

Figure S99: The force error for the parameters of the adaptive buffer method. The error of the force does not change significantly by changing the r_{buffer} parameter, so it was set to the computationally most inexpensive r_{buffer} = 1.0 Å. The r_{QM} was set to 3.0 Å. Level of theory used for the DFT calculations was PBE[14]/DZVP-MOLOPT-GTH[15,16]/D3[17].

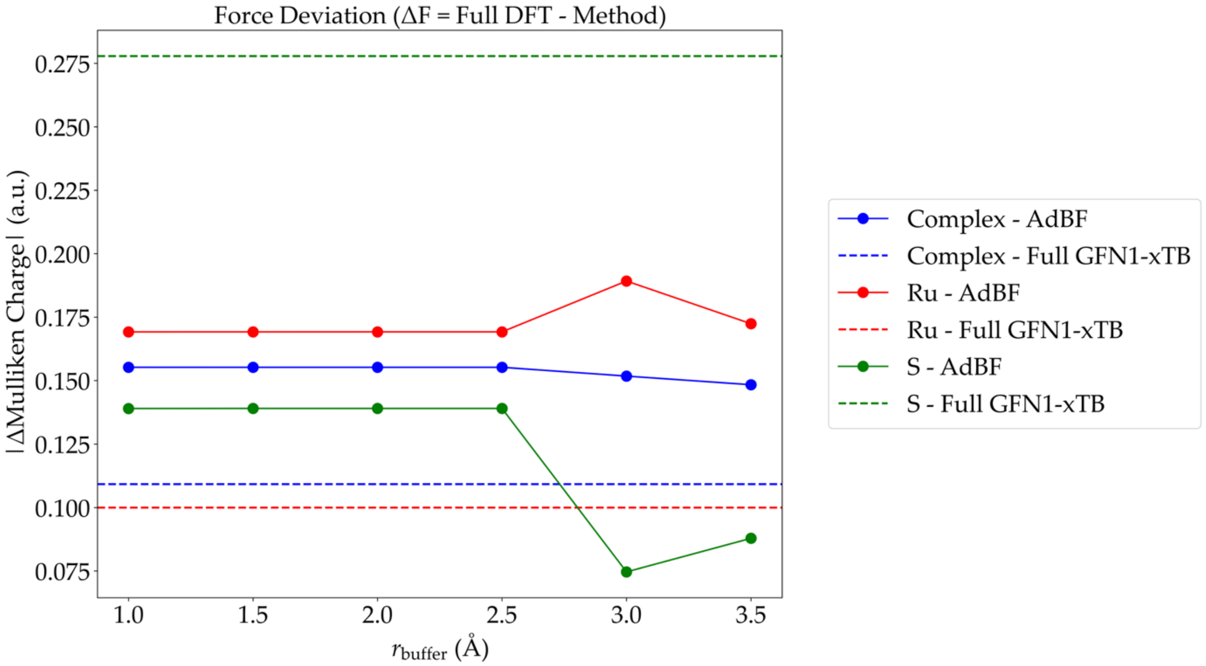

Figure S100: The Mulliken charge error for the parameters of the adaptive buffer method. The oxygen atom was not included as CP2K does not print the charges of the adaptive buffer by atom index, but by atom type making it impossible to differentiate between the oxygens in the dynamic QM zone. Level of theory used for the DFT calculations was PBE[14]/DZVP-MOLOPT-GTH[15,16]/D3[17].

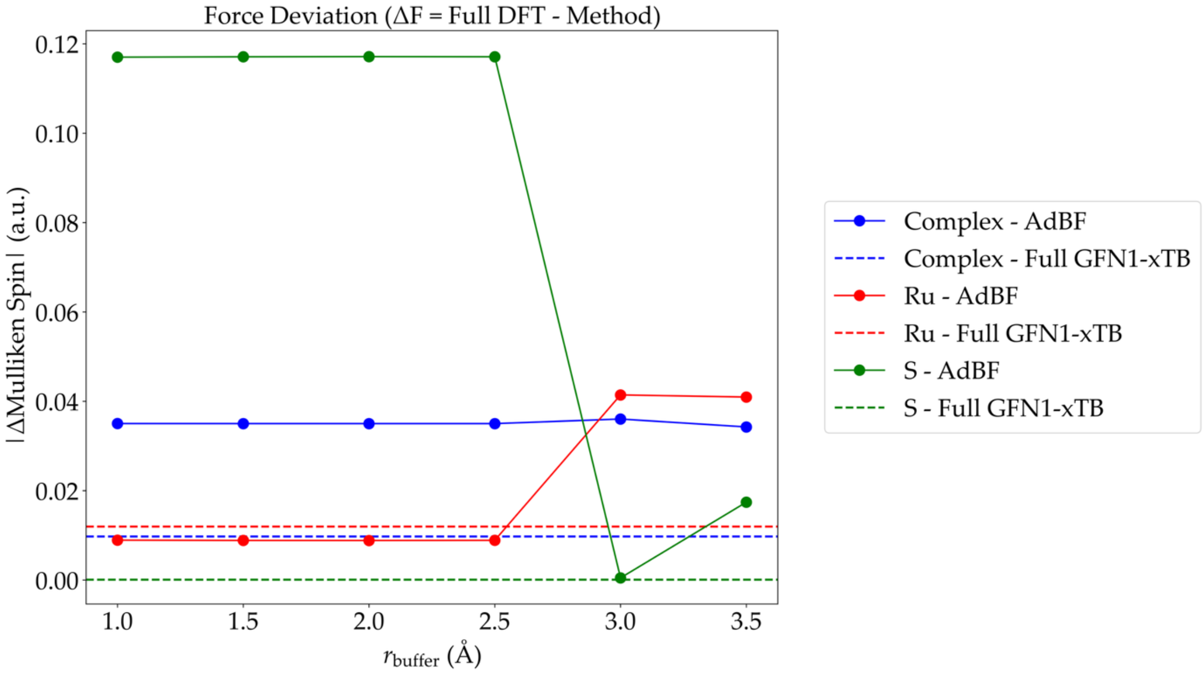

Figure S101: The Mulliken spin error for the parameters of the adaptive buffer method. The oxygen atom was not included as CP2K does not print the charges of the adaptive buffer by atom index, but by atom type making it impossible to differentiate between the oxygens in the dynamic QM zone. Level of theory used for the DFT calculations was PBE[14]/DZVP-MOLOPT-GTH[15,16]/D3[17].

Table S5. The Mayer bond orders (MBO) of the Ru-S and Ru-N_{trans} bond, where N_{trans} is the nitrogen *trans* to the sulphur atom, of the ^{1}GS and the ^{3}MLCT states.

Complex	Bond	^{1}GS	^{3}MLCT	ΔMBO (%)
**[Ru1]$^{2+}$**	Ru-N_{trans}	0.245	0.235	-4.4
	Ru-S	0.721	0.728	0.9
**[Ru2]$^{2+}$**	Ru-N_{trans}	0.253	0.334	31.8
	Ru-S	0.754	0.800	6.1
**[Ru3]$^{2+}$**	Ru-N_{trans}	0.254	0.264	2.4
	Ru-S	0.746	0.804	7.7
**[Ru4]$^{2+}$**	Ru-N_{trans}	0.249	0.265	6.5
	Ru-S	0.729	0.772	5.9
**[Ru5]$^{2+}$**	Ru-N_{trans}	0.252	0.258	2.2
	Ru-S	0.750	0.819	9.2
**[Ru6]$^{2+}$**	Ru-N_{trans}	0.229	0.266	6.3
	Ru-S	0.729	0.784	7.5
**[Ru7]$^{2+}$**	Ru-N_{trans}	0.229	0.254	10.7
	Ru-S	0.752	0.775	3.0
**[Ru8]$^{2+}$**	Ru-N_{trans}	0.236	0.263	11.7
	Ru-S	0.730	0.762	4.3
**[Ru9]$^{2+}$**	Ru-N_{trans}	0.234	0.256	9.6
	Ru-S	0.751	0.782	4.1

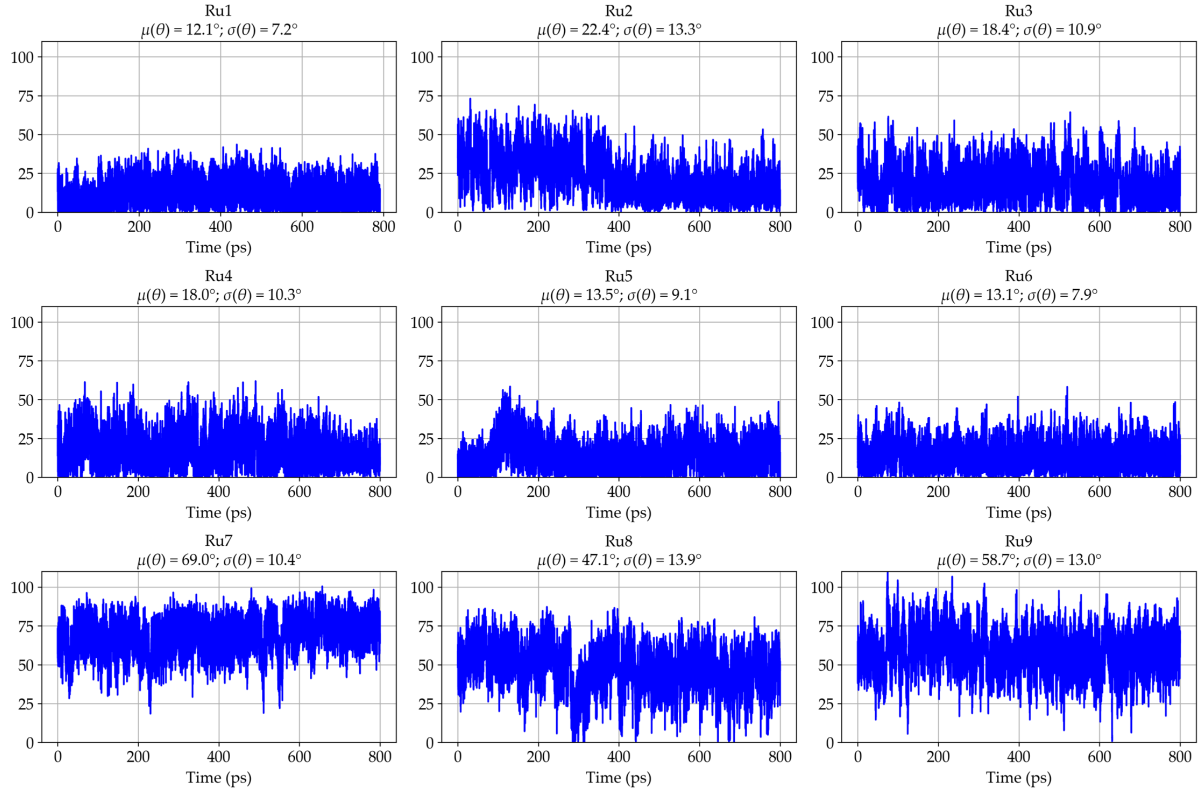

Figure S102: The angle of the plane θ defined in Figure 9 over the trajectory for compounds $[Ru1]^{2+}$ - $[Ru9]^{2+}$.

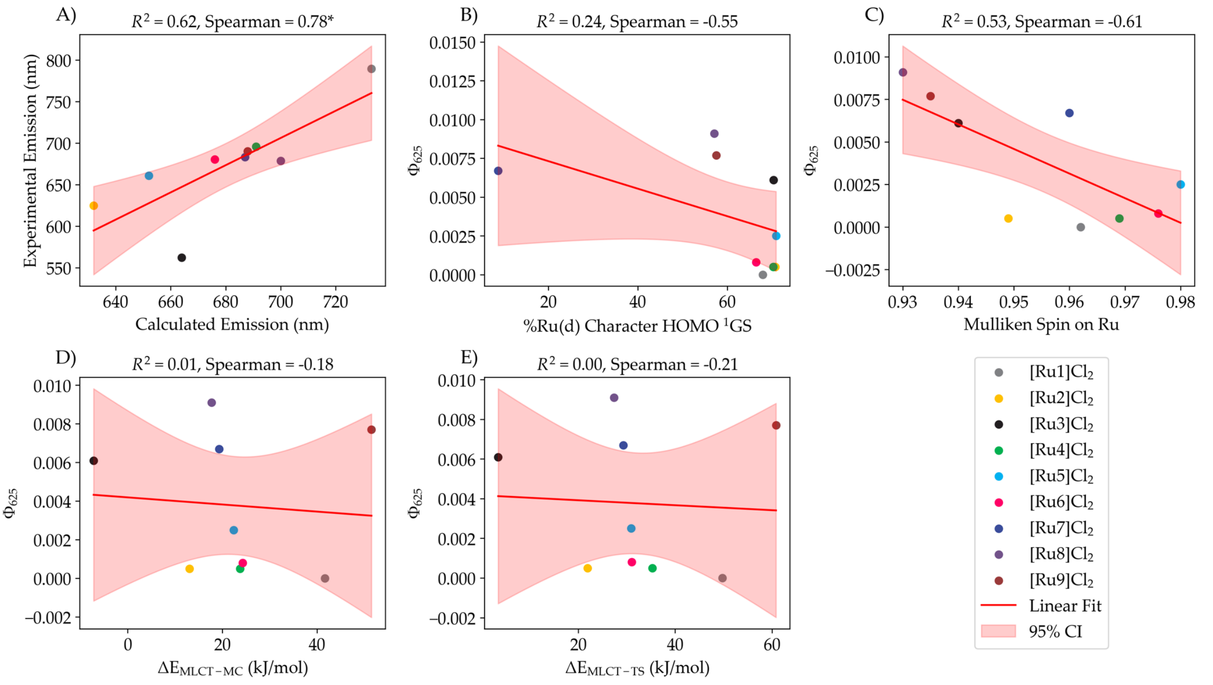

Figure S103: Static DFT modeling predict emission maxima but cannot predict photosubstitution quantum yields of compounds [Ru1]Cl$_2$-[Ru9]Cl$_2$. A) Plot of the experimental vs. calculated maximum emission wavelengths. B-D) Plots of the red-light photosubstitution quantum yield vs. B) the percentage of ruthenium d orbitals character in the HOMO of the ground singlet state, C) the Mulliken spin density on the ruthenium atom in the ^{3}MLCT excited state, or D) The energy difference between the MLCT and MC state ($\Delta E_{MLCT-MC}$) and E) the energy difference between the MLCT and TS state ($\Delta E_{MLCT-TS}$). The red line in the graph is the linear fit of the data points. A Spearman correlation coefficient without a * indicates p≥ 0.05 (not statistically significant). A single * indicates that $p < 0.05$, suggesting the correlation is statistically significant with a less than a 5% chance of occurring by random chance. The 95% confidence interval (CI) around the linear regression line was calculated by propagating the standard error of the regression residuals, accounting for the degrees of freedom (number of data points minus two), and scaled by the critical value of the t-distribution at 95% confidence (equation S2).

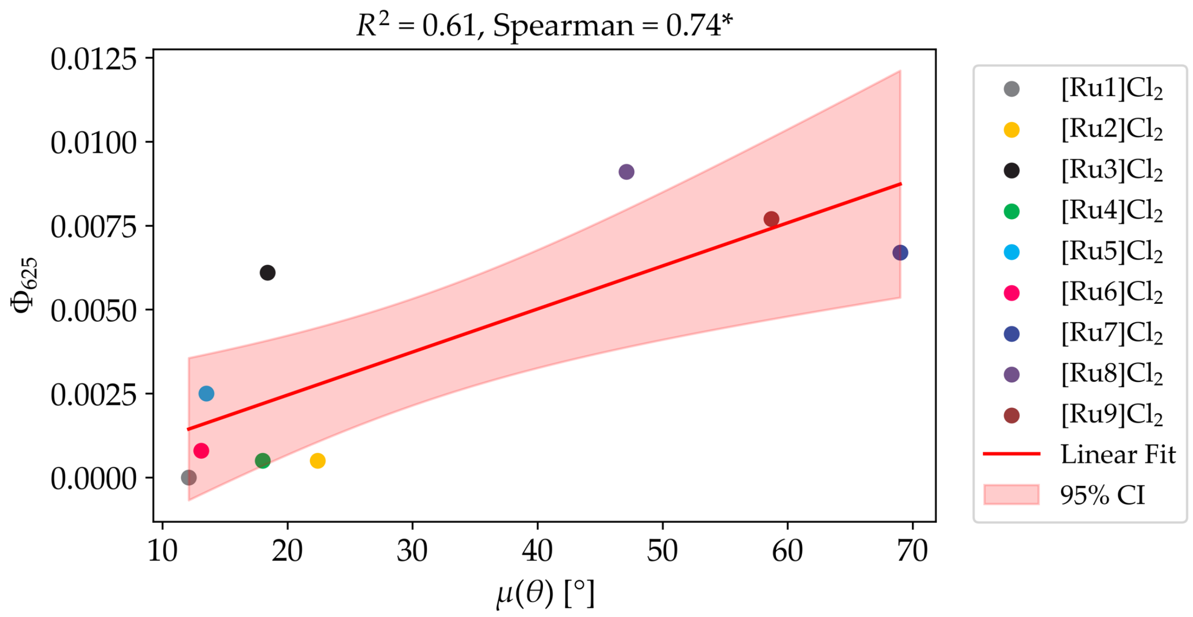

Figure S104: The red-light photosubstitution quantum yield as a function of μ(θ) (Figure 9). A single * indicates that $p < 0.05$, suggesting the correlation is statistically significant with a less than a 5% chance of occurring by random chance. The 95% confidence interval (CI) around the linear regression line was calculated by propagating the standard error of the regression residuals, accounting for the degrees of freedom (number of data points minus two), and scaled by the critical value of the t-distribution at 95% confidence (equation S2).

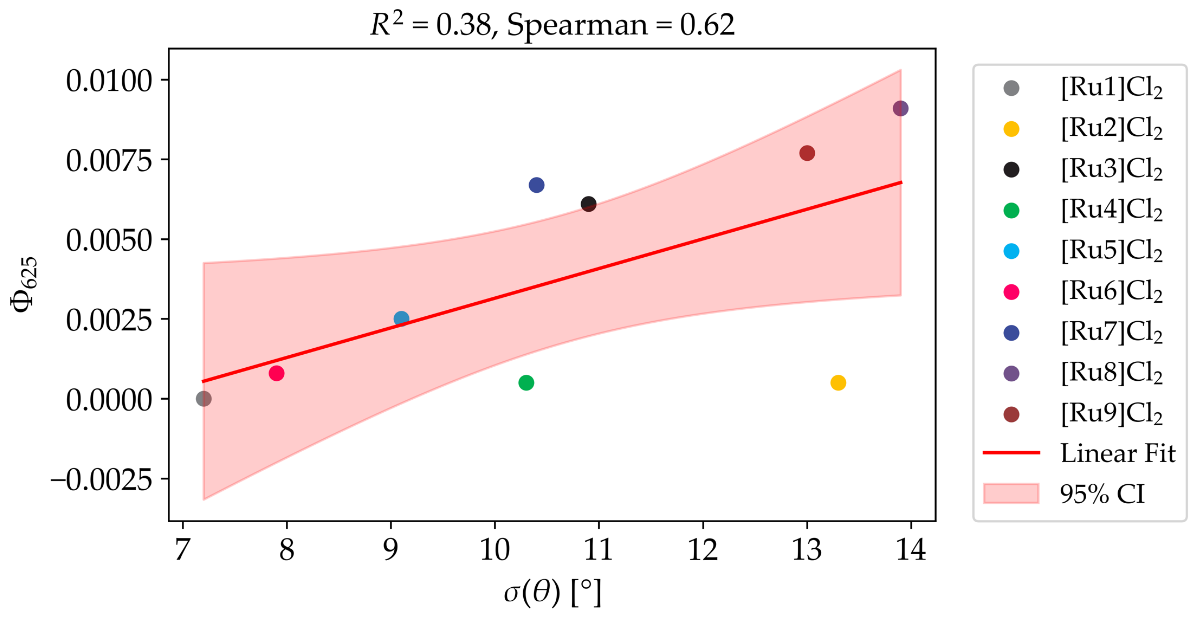

Figure S105: The red-light photosubstitution quantum yield as a function of σ(θ) (Figure 9). A Spearman correlation coefficient without a * indicates $p \geq 0.05$ (not statistically significant). The 95% confidence interval (CI) around the linear regression line was calculated by propagating the standard error of the regression residuals, accounting for the degrees of freedom (number of data points minus two), and scaled by the critical value of the t-distribution at 95% confidence (equation S2).

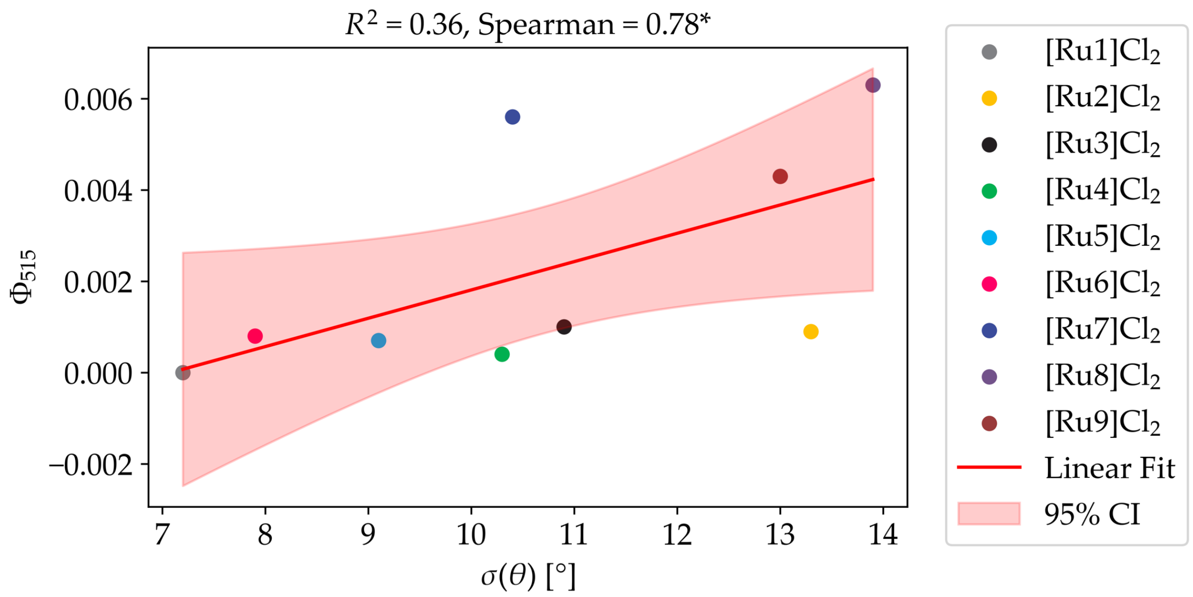

Figure S106: The green-light photosubstitution quantum yield as a function of σ(θ) (Figure 9). A single * indicates that $p < 0.05$, suggesting the correlation is statistically significant with a less than a 5% chance of occurring by random chance. The 95% confidence interval (CI) around the linear regression line was calculated by propagating the standard error of the regression residuals, accounting for the degrees of freedom (number of data points minus two), and scaled by the critical value of the t-distribution at 95% confidence (equation S2).

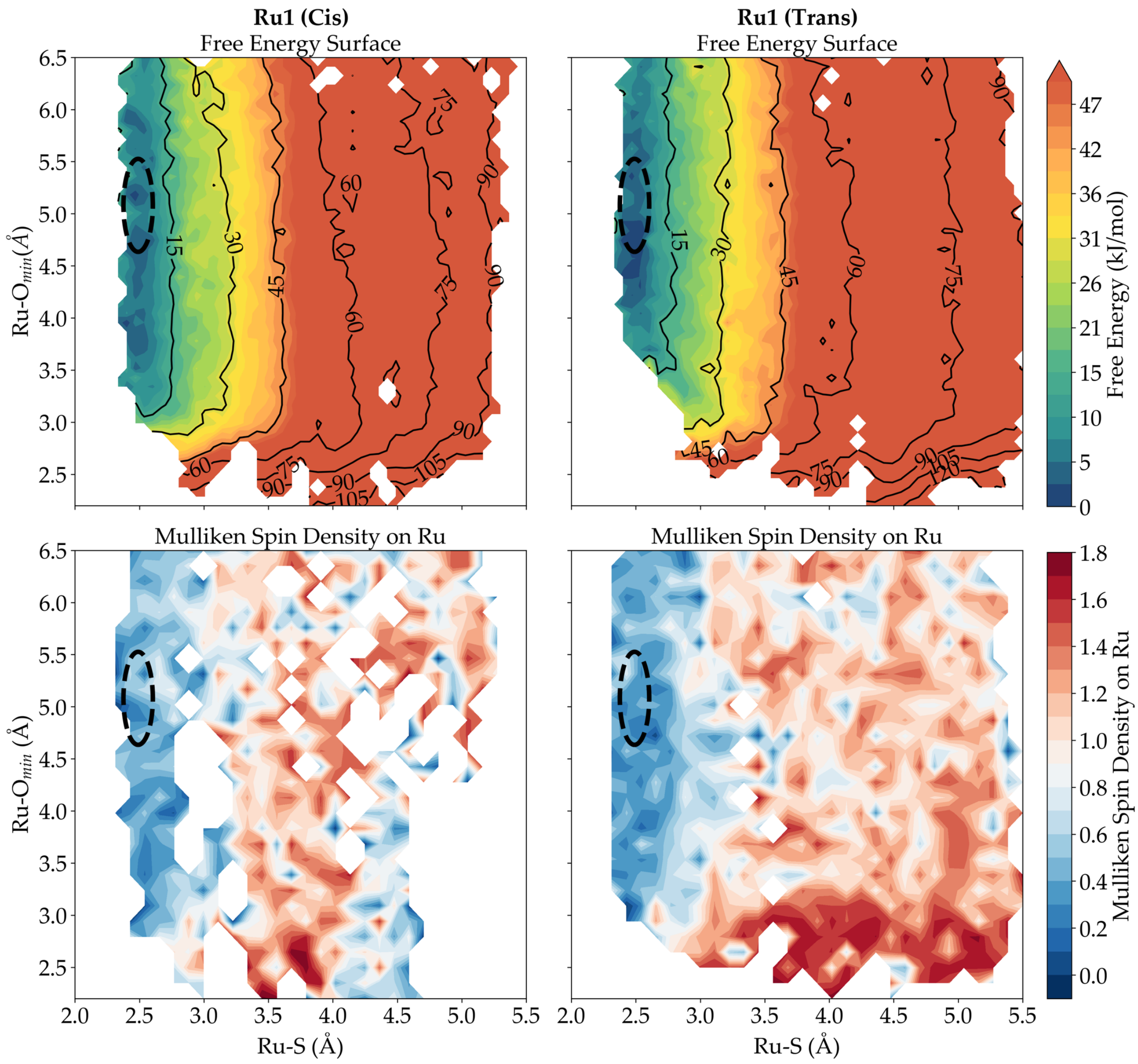

Figure S107: Free energy surfaces for the cis and trans pathways of compound $[Ru1]^{2+}$ according to the AQUAMEPhT protocol. The Mulliken spin values on the ruthenium atom, and energy surfaces were derived from configurations extracted using the delta-net algorithm.[18] DFT single-point calculations (PBE[14]/DZVP-MOLOPT-GTH[16]/D3[17]) were then performed, treating the same atoms at the QM level as in the extracted configurations The black dotted circle represents the 95% probable region on the CV surface where photon absorption is most likely to occur. For visual guidance, the most probable trajectory along the minimum energy path is shown in cyan. The path was manually added to aid interpretation.

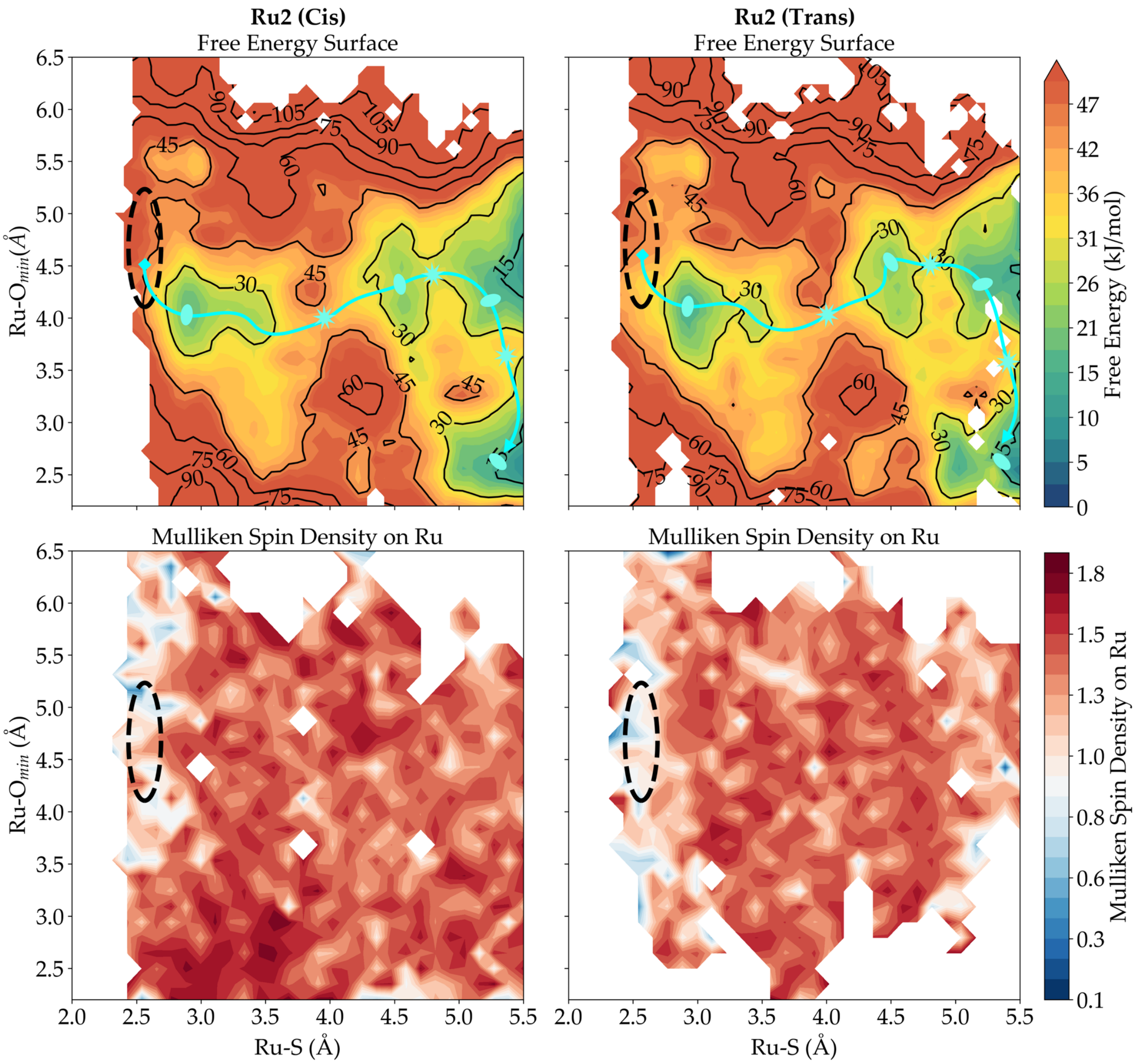

Figure S108: Free energy surfaces for the cis and trans pathways of compound $[Ru2]^{2+}$ according to the AQUAMEPhT protocol. The Mulliken spin values on the ruthenium atom, and energy surfaces were derived from configurations extracted using the delta-net algorithm.[18] DFT single-point calculations (PBE[14]/DZVP-MOLOPT-GTH[16]/D3[17]) were then performed, treating the same atoms at the QM level as in the extracted configurations. The black dotted circle represents the 95% probable region on the CV surface where photon absorption is most likely to occur. For visual guidance, the most probable trajectory along the minimum energy path is shown in cyan. Energy minima are marked with ovals, and approximate transition states with stars. The path and markers were manually added to aid interpretation.

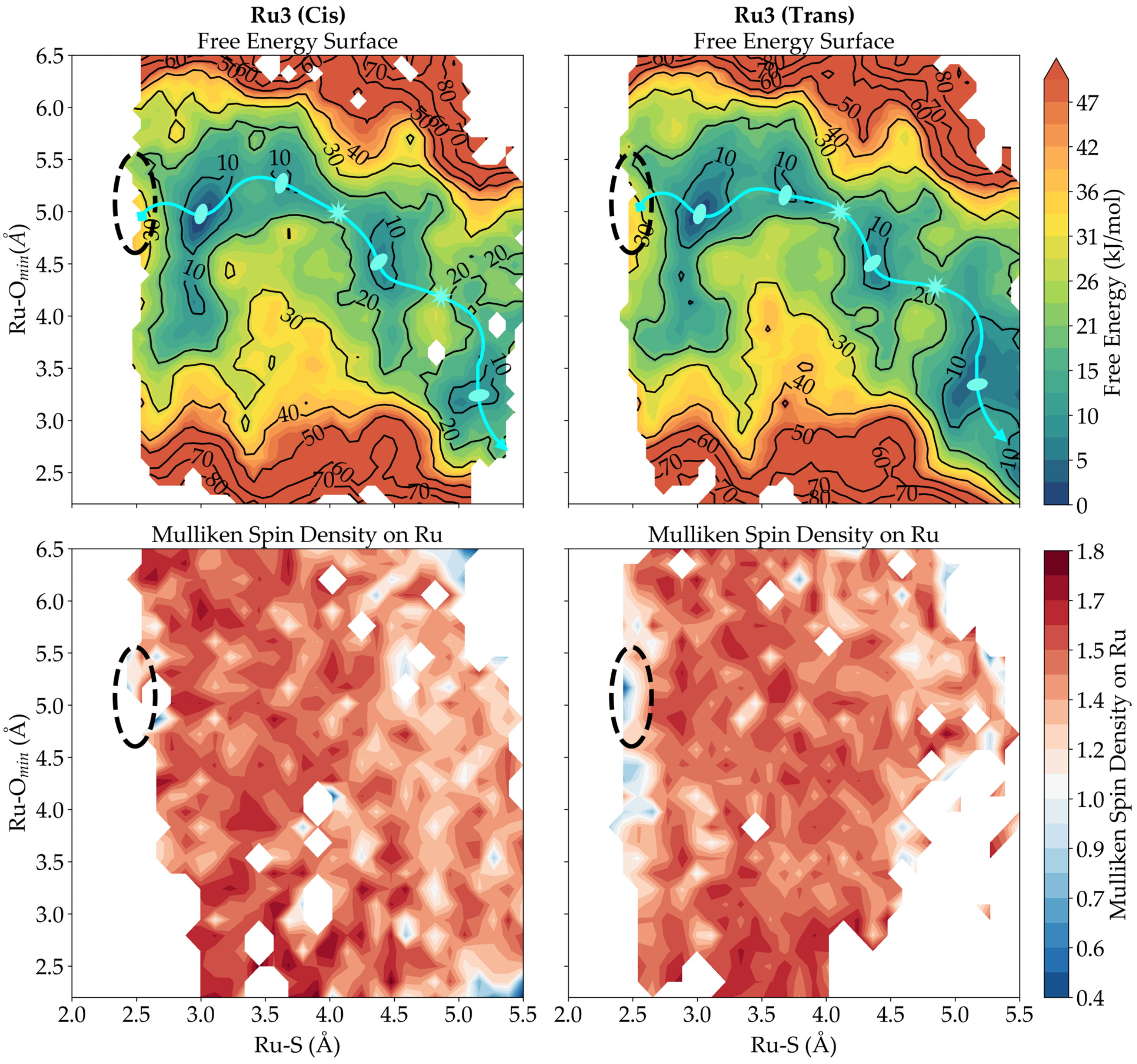

Figure S109: Free energy surfaces for the cis and trans pathways of compound $[Ru3]^{2+}$ according to the AQUAMEPhT protocol. The Mulliken spin values on the ruthenium atom, and energy surfaces were derived from configurations extracted using the delta-net algorithm.[18] DFT single-point calculations (PBE[14]/DZVP-MOLOPT-GTH[16]/D3[17]) were then performed, treating the same atoms at the QM level as in the extracted configurations. The black dotted circle represents the 95% probable region on the CV surface where photon absorption is most likely to occur. For visual guidance, the most probable trajectory along the minimum energy path is shown in cyan. Energy minima are marked with ovals, and approximate transition states with stars. The path and markers were manually added to aid interpretation.

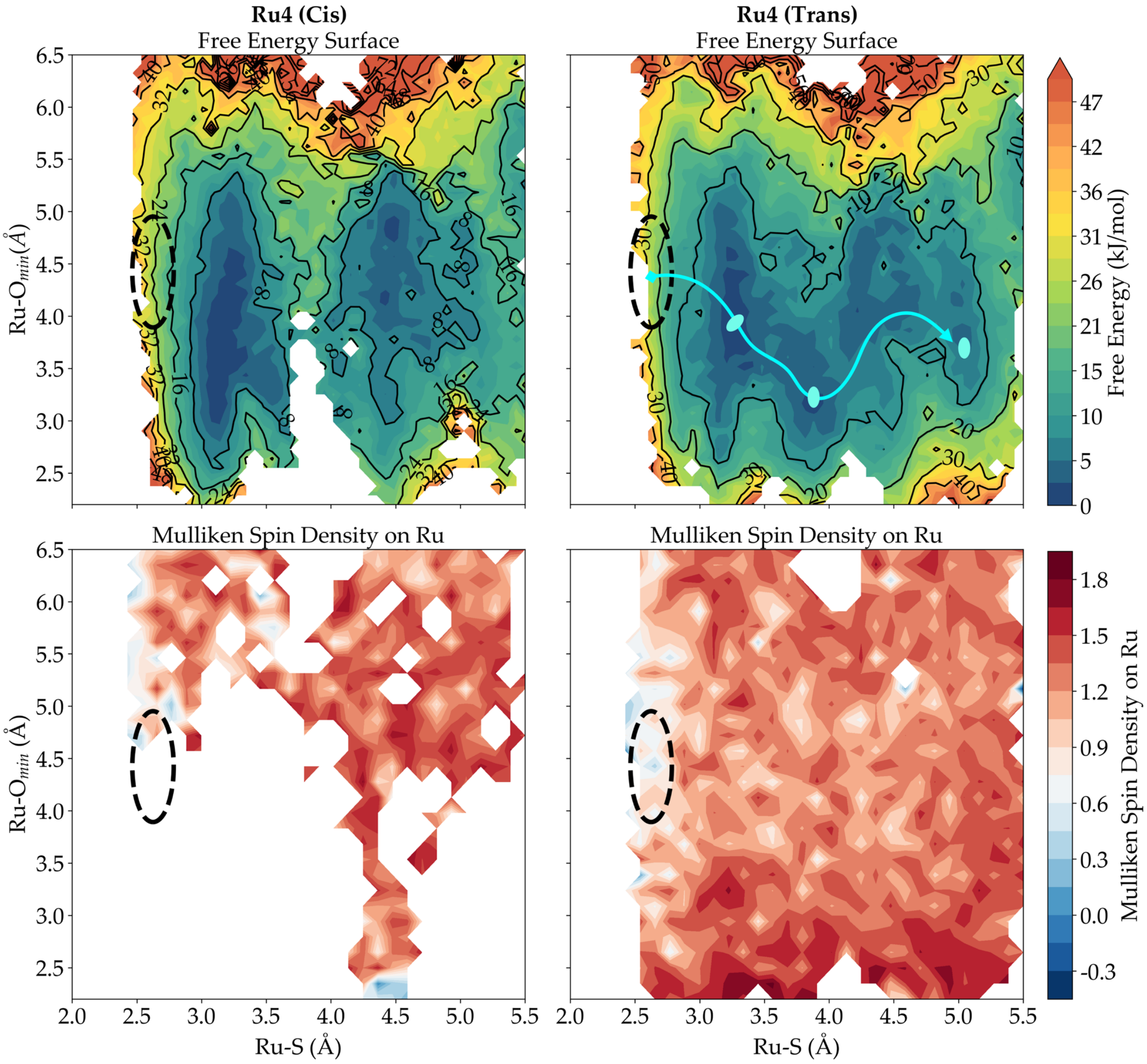

Figure S110: Free energy surfaces for the cis and trans pathways of compound $[Ru4]^{2+}$ according to the AQUAMEPhT protocol. The Mulliken spin values on the ruthenium atom, and energy surfaces were derived from configurations extracted using the delta-net algorithm.[18] DFT single-point calculations (PBE[14]/DZVP-MOLOPT-GTH[16]/D3[17]) were then performed, treating the same atoms at the QM level as in the extracted configurations. The black dotted circle represents the 95% probable region on the CV surface where photon absorption is most likely to occur. For visual guidance, the most probable trajectory along the minimum energy path is shown in cyan. Energy minima are marked with ovals, and approximate transition states with stars. The path and markers were manually added to aid interpretation.

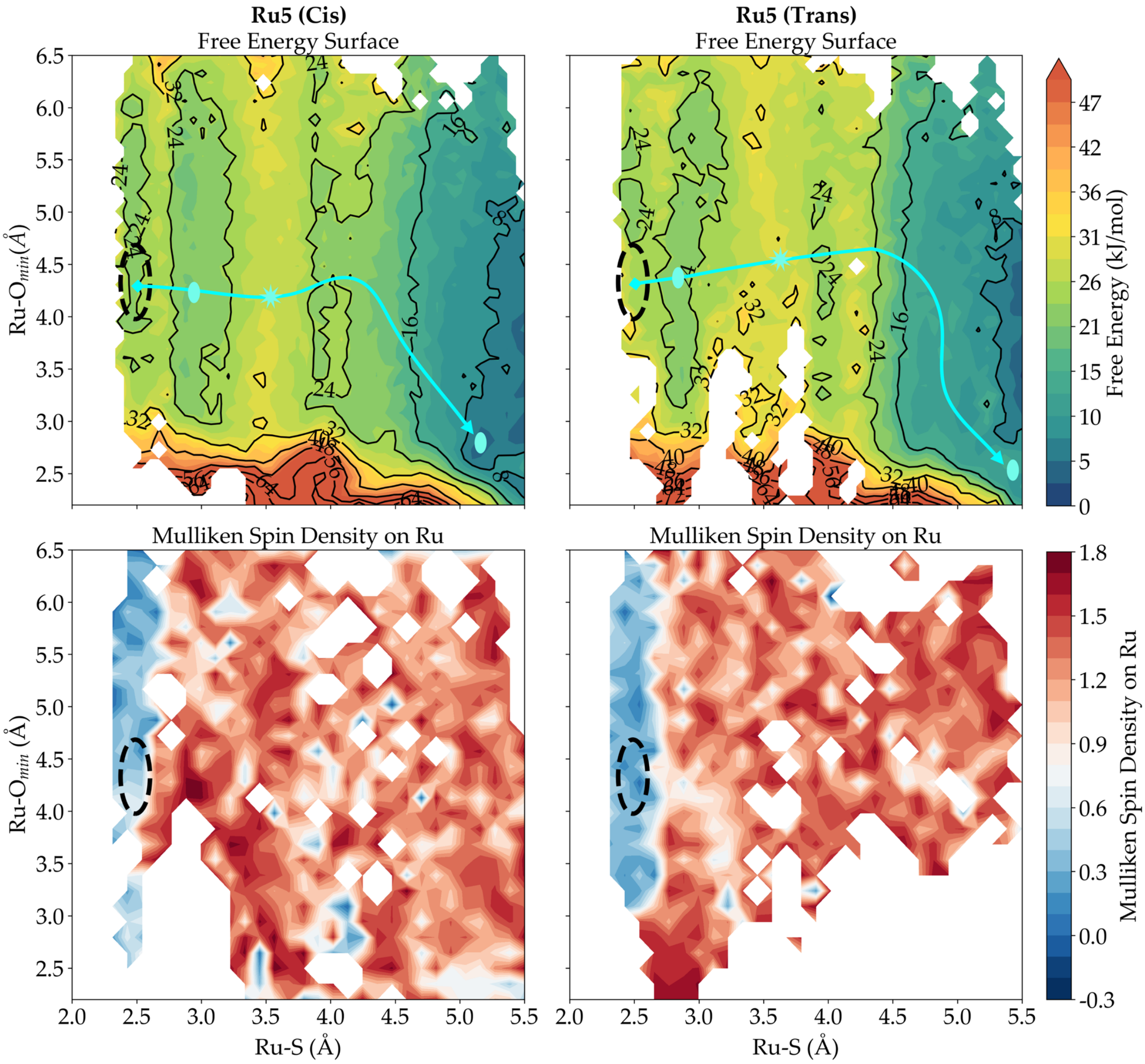

Figure S111: Free energy surfaces for the cis and trans pathways of compound $[Ru5]^{2+}$ according to the AQUAMEPhT protocol. The Mulliken spin values on the ruthenium atom, and energy surfaces were derived from configurations extracted using the delta-net algorithm.[18] DFT single-point calculations (PBE[14]/DZVP-MOLOPT-GTH[16]/D3[17]) were then performed, treating the same atoms at the QM level as in the extracted configurations. The black dotted circle represents the 95% probable region on the CV surface where photon absorption is most likely to occur. For visual guidance, the most probable trajectory along the minimum energy path is shown in cyan. Energy minima are marked with ovals, and approximate transition states with stars. The path and markers were manually added to aid interpretation.

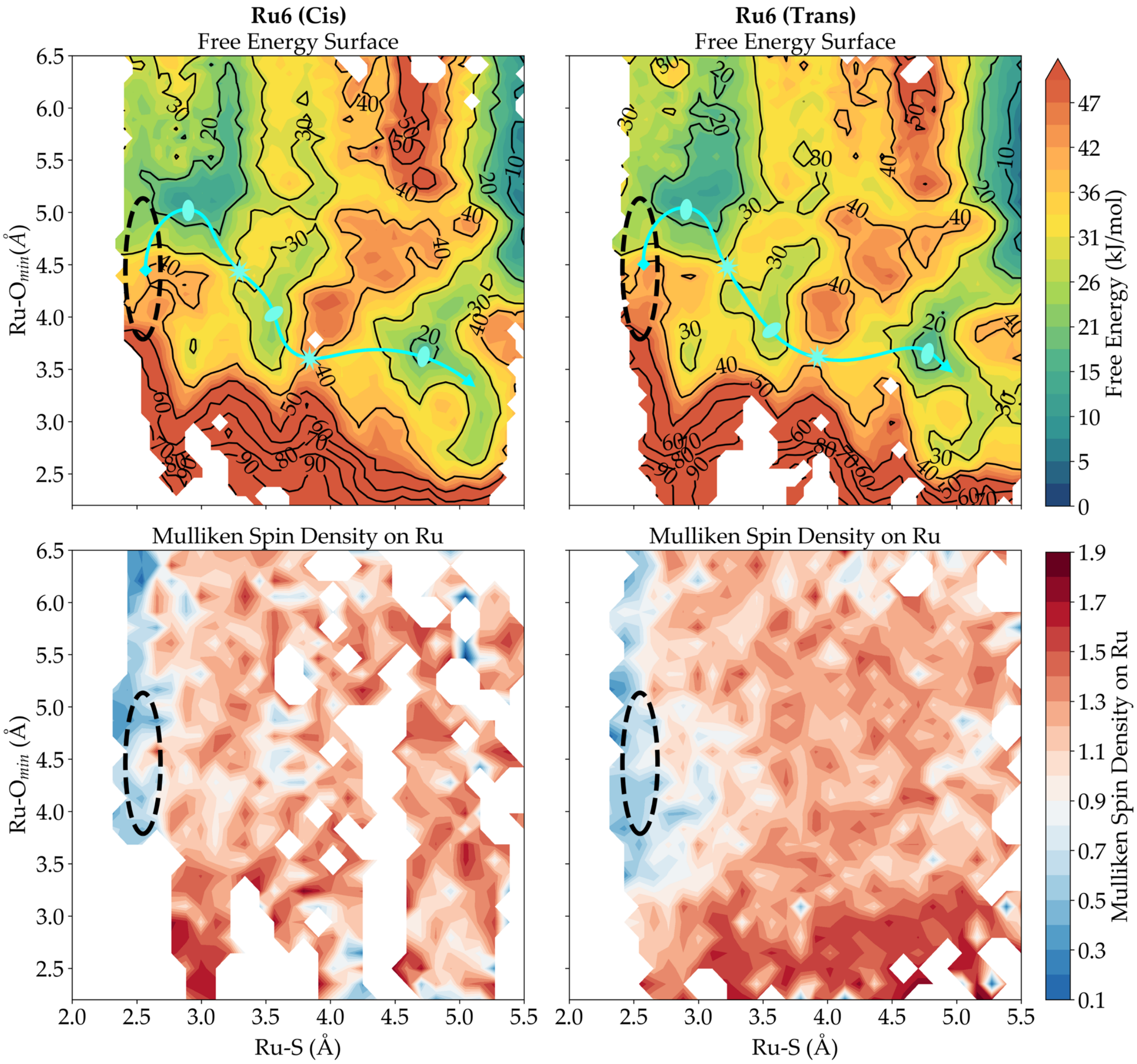

Figure S112: Free energy surfaces for the cis and trans pathways of compound $[Ru6]^{2+}$ according to the AQUAMEPhT protocol. The Mulliken spin values on the ruthenium atom, and energy surfaces were derived from configurations extracted using the delta-net algorithm.[18] DFT single-point calculations (PBE[14]/DZVP-MOLOPT-GTH[16]/D3[17]) were then performed, treating the same atoms at the QM level as in the extracted configurations. The black dotted circle represents the 95% probable region on the CV surface where photon absorption is most likely to occur. For visual guidance, the most probable trajectory along the minimum energy path is shown in cyan. Energy minima are marked with ovals, and approximate transition states with stars. The path and markers were manually added to aid interpretation.

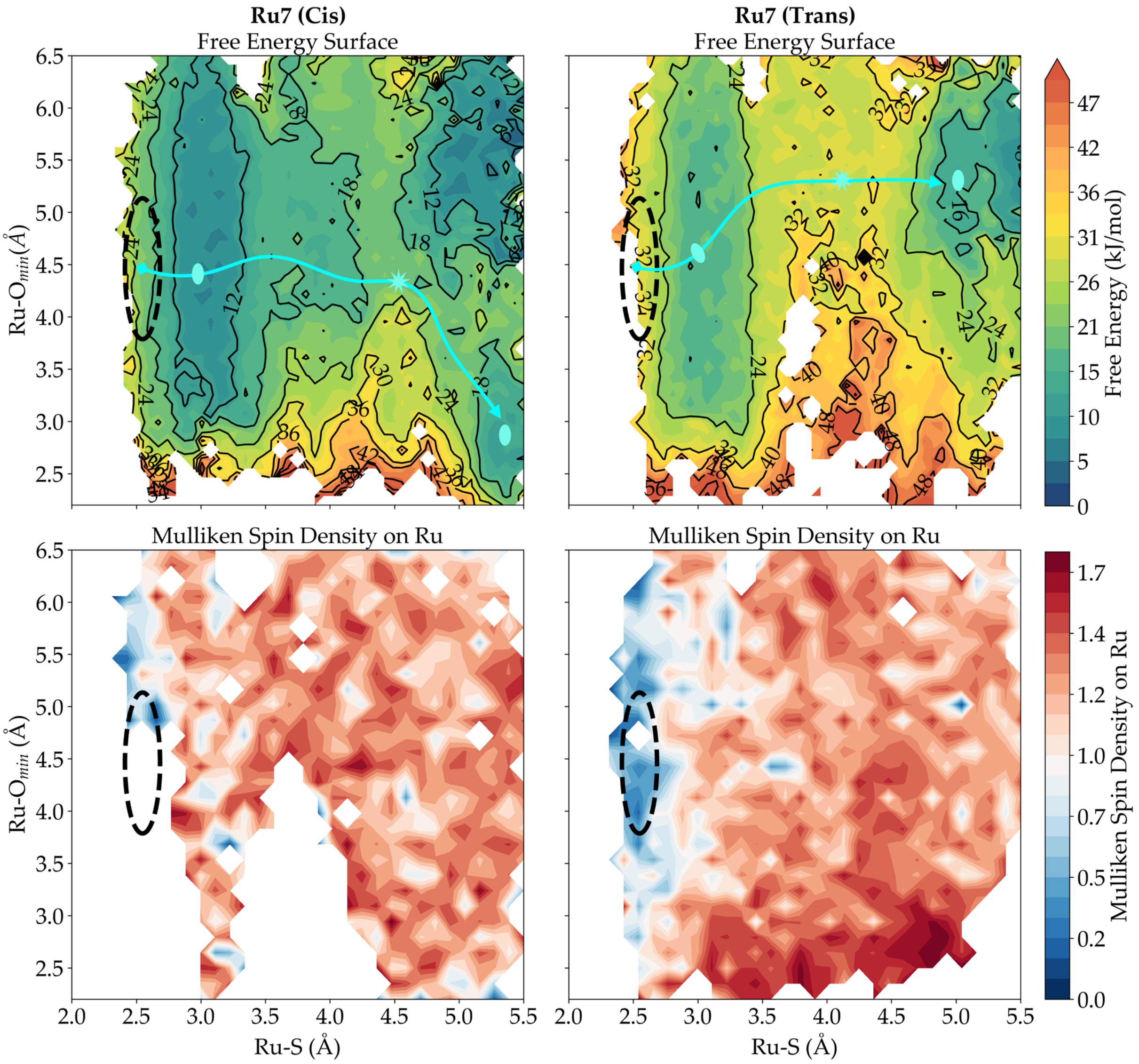

Figure S113: Free energy surfaces for the cis and trans pathways of compound $[Ru7]^{2+}$ according to the AQUAMEPhT protocol. The Mulliken spin values on the ruthenium atom, and energy surfaces were derived from configurations extracted using the delta-net algorithm.[18] DFT single-point calculations (PBE[14]/DZVP-MOLOPT-GTH[16]/D3[17]). were then performed, treating the same atoms at the QM level as in the extracted configurations. The black dotted circle represents the 95% probable region on the CV surface where photon absorption is most likely to occur. For visual guidance, the most probable trajectory along the minimum energy path is shown in cyan. Energy minima are marked with ovals, and approximate transition states with stars. The path and markers were manually added to aid interpretation.

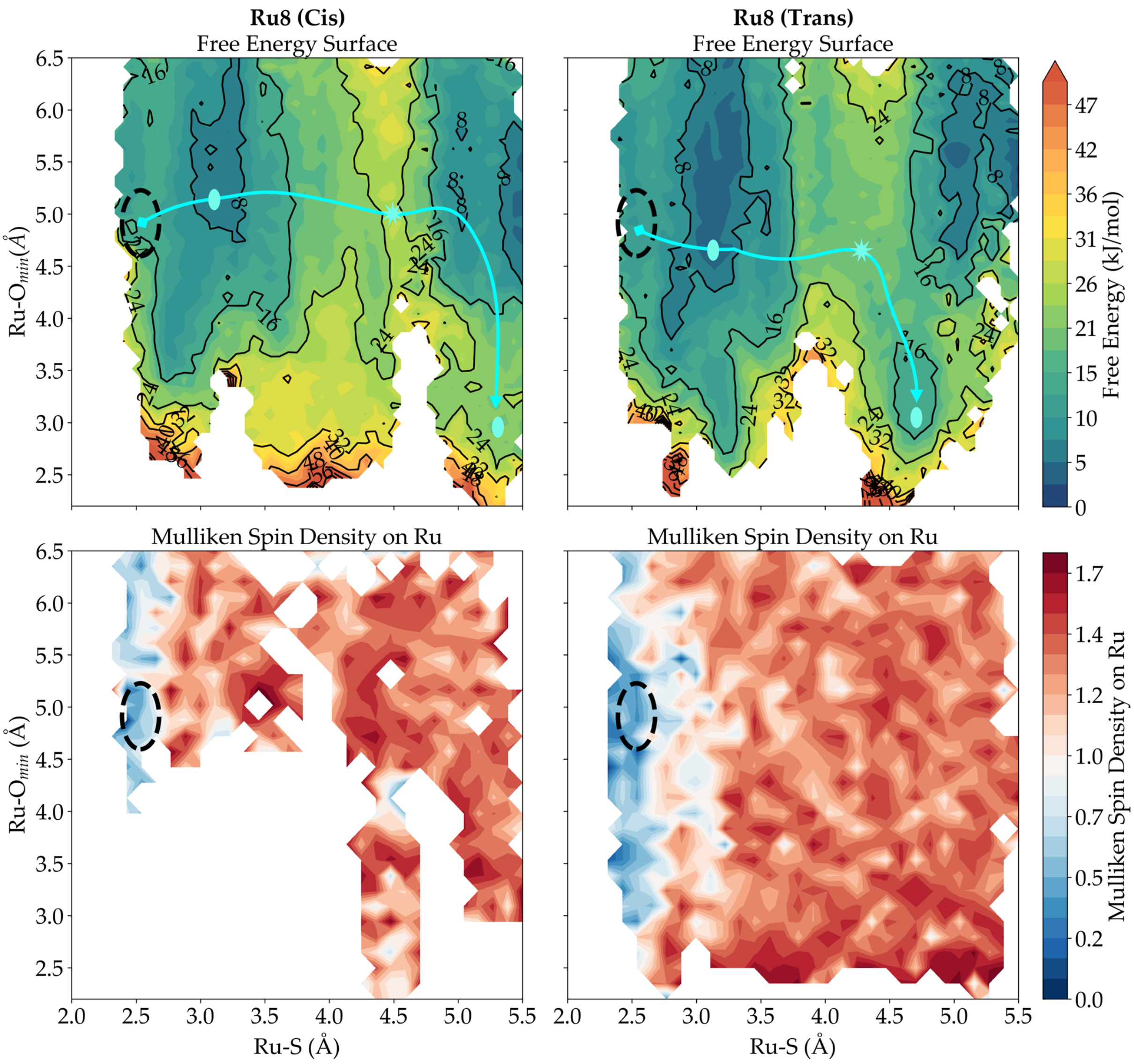

Figure S114: Free energy surfaces for the cis and trans pathways of compound $[Ru8]^{2+}$ according to the AQUAMEPhT protocol. The Mulliken spin values on the ruthenium atom, and energy surfaces were derived from configurations extracted using the delta-net algorithm.[18] DFT single-point calculations (PBE[14]/DZVP-MOLOPT-GTH[16]/D3[17]). were then performed, treating the same atoms at the QM level as in the extracted configurations. The red dotted circle represents the 95% probable region on the CV surface where photon absorption is most likely to occur. For visual guidance, the most probable trajectory along the minimum energy path is shown in cyan. Energy minima are marked with ovals, and approximate transition states with stars. The path and markers were manually added to aid interpretation.

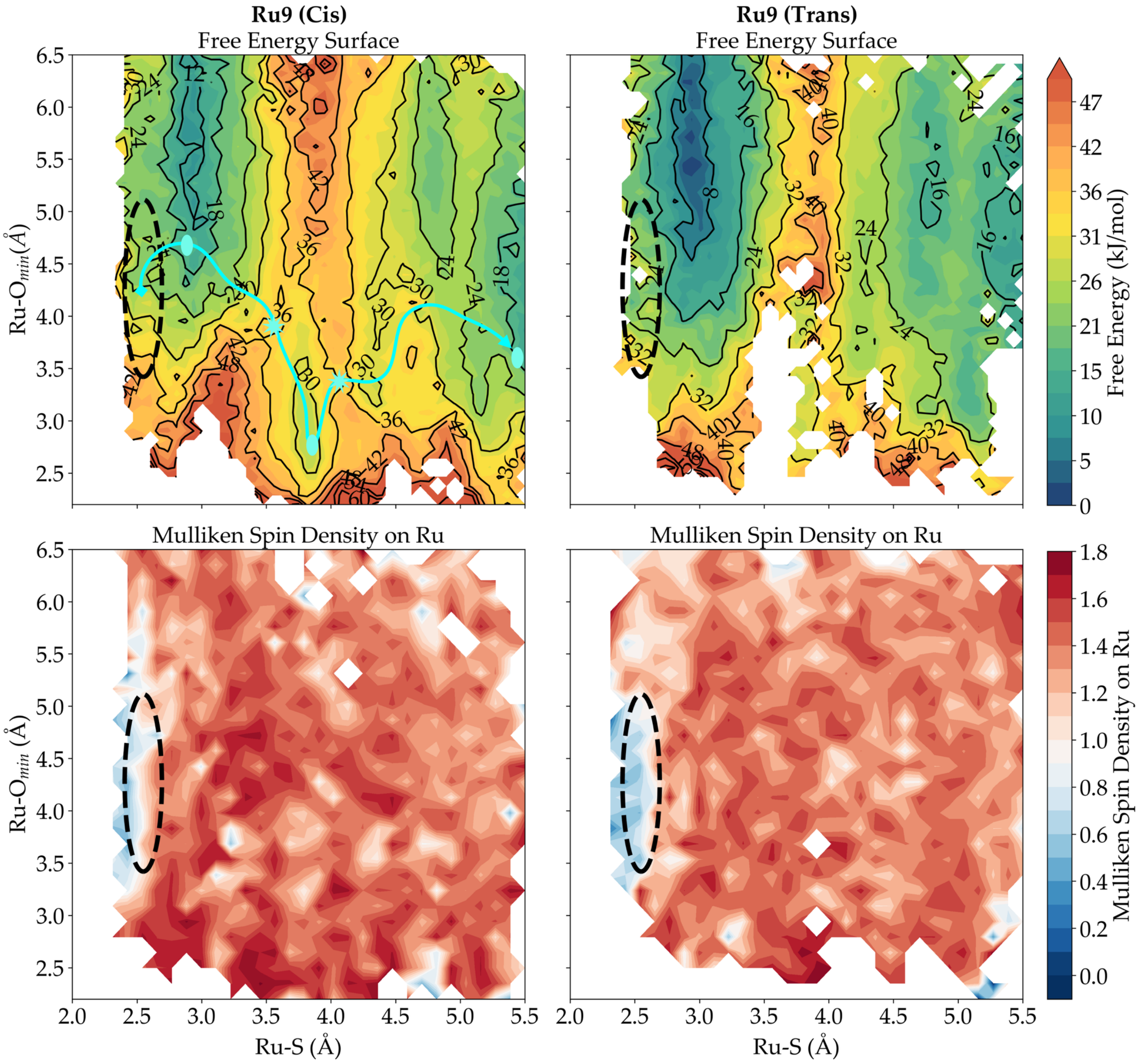

Figure S115: Free energy surfaces for the cis and trans pathways of compound $[Ru9]^{2+}$ according to the AQUAMEPhT protocol. The Mulliken spin values on the ruthenium atom, and energy surfaces were derived from configurations extracted using the delta-net algorithm.[18] DFT single-point calculations (PBE[14]/DZVP-MOLOPT-GTH[16]/D3[17]). were then performed, treating the same atoms at the QM level as in the extracted configurations. The black dotted circle represents the 95% probable region on the CV surface where photon absorption is most likely to occur. For visual guidance, the most probable trajectory along the minimum energy path is shown in cyan. Energy minima are marked with ovals, and approximate transition states with stars. The path and markers were manually added to aid interpretation.

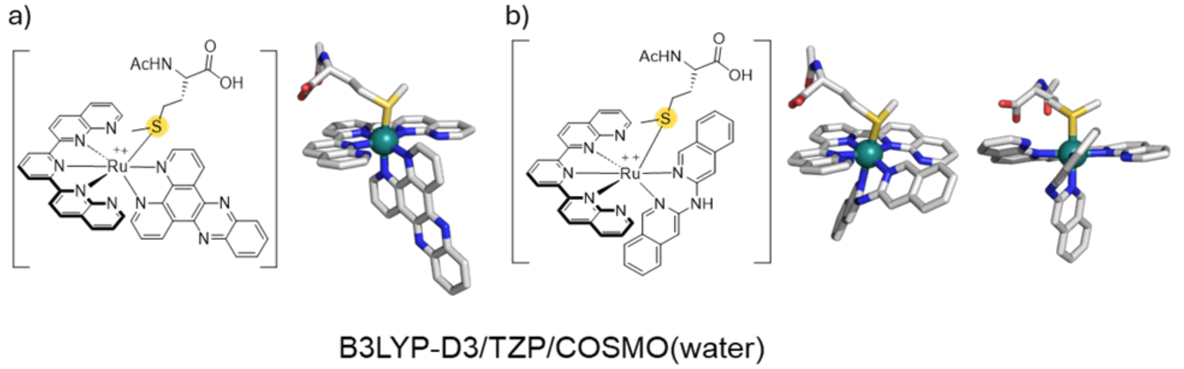

Figure S1116: Comparison of molecular models (B3LYP[19]-D3[17]/TZP[20]/COSMO(water)[21]) for a) [Ru1]Cl_2 and b) [Ru10]Cl_2.

7. References

(1) Lifshits, L. M.; Iii, J. A. R.; Konda, P.; Monro, S.; Cole, H. D.; Dohlen, D. von; Kim, S.; Deep, G.; Thummel, R. P.; Cameron, C. G.; Gujar, S.; McFarland, S. A. Near-Infrared Absorbing Ru(II) Complexes Act as Immunoprotective Photodynamic Therapy (PDT) Agents against Aggressive Melanoma. *Chem. Sci.* **2020**, *11* (43), 11740–11762. https://doi.org/10.1039/D0SC03875J.

(2) Busemann, A.; Flaspohler, I.; Zhou, X.-Q.; Schmidt, C.; Goetzfried, S. K.; van Rixel, V. H. S.; Ott, I.; Siegler, M. A.; Bonnet, S. Ruthenium-Based PACT Agents Based on Bisquinoline Chelates: Synthesis, Photochemistry, and Cytotoxicity. *J Biol Inorg Chem* **2021**, *26* (6), 667–674. https://doi.org/10.1007/s00775-021-01882-8.

(3) Bretin, L.; Husiev, Y.; Ramu, V.; Zhang, L.; Hakkennes, M.; Abyar, S.; Johns, A. C.; Le Dévédec, S. E.; Betancourt, T.; Kornienko, A.; Bonnet, S. Red-Light Activation of a Microtubule Polymerization Inhibitor via Amide Functionalization of the Ruthenium Photocage. *Angewandte Chemie International Edition* **2024**, *63* (5), e202316425. https://doi.org/10.1002/anie.202316425.

(4) Sauvageot, E.; Lafite, P.; Duverger, E.; Marion, R.; Hamel, M.; Gaillard, S.; Renaud, J.-L.; Daniellou, R. Iridium Complexes Inhibit Tumor Necrosis Factor-α by Utilizing Light and Mixed Ligands. *Journal of Organometallic Chemistry* **2016**, *808*, 122–127. https://doi.org/10.1016/j.jorganchem.2016.02.001.

(5) Zhou, X.-Q.; Busemann, A.; Meijer, M. S.; Siegler, M. A.; Bonnet, S. The Two Isomers of a Cyclometallated Palladium Sensitizer Show Different Photodynamic Properties in Cancer Cells. *Chem. Commun.* **2019**, *55* (32), 4695–4698. https://doi.org/10.1039/C8CC10134E.

(6) Suzuki, K.; Kobayashi, A.; Kaneko, S.; Takehira, K.; Yoshihara, T.; Ishida, H.; Shiina, Y.; Oishi, S.; Tobita, S. Reevaluation of Absolute Luminescence Quantum Yields of Standard Solutions Using a Spectrometer with an Integrating Sphere and a Back-Thinned CCD Detector. *Phys. Chem. Chem. Phys.* **2009**, *11* (42), 9850. https://doi.org/10.1039/b912178a.

(7) Ossola, R.; Jönsson, O. M.; Moor, K.; McNeill, K. Singlet Oxygen Quantum Yields in Environmental Waters. *Chem. Rev.* **2021**, *121* (7), 4100–4146. https://doi.org/10.1021/acs.chemrev.0c00781.

(8) Partanen, S. B.; Erickson, P. R.; Latch, D. E.; Moor, K. J.; McNeill, K. Dissolved Organic Matter Singlet Oxygen Quantum Yields: Evaluation Using Time-Resolved Singlet Oxygen Phosphorescence. *Environ. Sci. Technol.* **2020**, *54* (6), 3316–3324. https://doi.org/10.1021/acs.est.9b07246.

(9) Schmidt, R.; Tanielian, C.; Dunsbach, R.; Wolff, C. Phenalenone, a Universal Reference Compound for the Determination of Quantum Yields of Singlet Oxygen O2(1Δg) Sensitization. *Journal of Photochemistry and Photobiology A: Chemistry* **1994**, *79* (1–2), 11–17. https://doi.org/10.1016/1010-6030(93)03746-4.

(10) Abdel-Shafi, A. A.; Beer, P. D.; Mortimer, R. J.; Wilkinson, F. Photosensitized Generation of Singlet Oxygen from Vinyl Linked Benzo-Crown-Ether−Bipyridyl Ruthenium(II) Complexes. *J. Phys. Chem. A* **2000**, *104* (2), 192–202. https://doi.org/10.1021/jp991876z.

(11) Sheldrick, G. M. SHELXT – Integrated Space-Group and Crystal-Structure Determination. *Acta Crystallographica Section A* **2015**, *71* (1), 3–8. https://doi.org/10.1107/S2053273314026370.

(12) Spek, A. L. Structure Validation in Chemical Crystallography. *Acta Cryst D* **2009**, *65* (2), 148–155. https://doi.org/10.1107/S090744490804362X.

(13) Montgomery, D. C.; Peck, E. A.; Vining, G. G. Introduction to Linear Regression Analysis.

(14) Perdew, J. P.; Burke, K.; Ernzerhof, M. Generalized Gradient Approximation Made Simple. *Phys. Rev. Lett.* **1996**, *77* (18), 3865–3868. https://doi.org/10.1103/PhysRevLett.77.3865.

(15) Kühne, T. D.; Iannuzzi, M.; Ben, M. D.; Rybkin, V. V.; Seewald, P.; Stein, F.; Laino, T.; Khaliullin, R. Z.; Schütt, O.; Schiffmann, F.; Golze, D.; Wilhelm, J.; Chulkov, S.; Bani-Hashemian, M. H.; Weber, V.; Borštnik, U.; Taillefumier, M.; Jakobovits, A. S.; Lazzaro, A.; Pabst, H.; Müller, T.; Schade, R.; Guidon, M.; Andermatt, S.; Holmberg, N.; Schenter, G. K.; Hehn, A.; Bussy, A.; Belleflamme, F.; Tabacchi, G.; Glöß, A.; Lass, M.; Bethune, I.; Mundy, C. J.; Plessl, C.; Watkins, M.; VandeVondele, J.; Krack, M.; Hutter, J. CP2K: An Electronic Structure and Molecular Dynamics Software Package - Quickstep: Efficient and Accurate Electronic Structure Calculations. *The Journal of Chemical Physics* **2020**, *152* (19), 194103. https://doi.org/10.1063/5.0007045.

(16) VandeVondele, J.; Hutter, J. Gaussian Basis Sets for Accurate Calculations on Molecular Systems in Gas and Condensed Phases. *The Journal of Chemical Physics* **2007**, *127* (11), 114105. https://doi.org/10.1063/1.2770708.

(17) Grimme, S.; Antony, J.; Ehrlich, S.; Krieg, H. A Consistent and Accurate Ab Initio Parametrization of Density Functional Dispersion Correction (DFT-D) for the 94 Elements H-Pu. *The Journal of Chemical Physics* **2010**, *132* (15), 154104. https://doi.org/10.1063/1.3382344.
(18) Crosskey, M.; Maggioni, M. ATLAS: A Geometric Approach to Learning High-Dimensional Stochastic Systems Near Manifolds. *Multiscale Model. Simul.* **2017**, *15* (1), 110–156. https://doi.org/10.1137/140970951.
(19) Stephens, P. J.; Devlin, F. J.; Chabalowski, C. F.; Frisch, M. J. Ab Initio Calculation of Vibrational Absorption and Circular Dichroism Spectra Using Density Functional Force Fields. *J. Phys. Chem.* **1994**, *98* (45), 11623–11627. https://doi.org/10.1021/j100096a001.
(20) Chong, D. P.; Van Lenthe, E.; Van Gisbergen, S.; Baerends, E. J. Even-tempered slater-type orbitals revisited: From hydrogen to krypton. *Journal of Computational Chemistry* **2004**, *25* (8), 1030–1036. https://doi.org/10.1002/jcc.20030.
(21) Pye, C. C.; Ziegler, T. An Implementation of the Conductor-like Screening Model of Solvation within the Amsterdam Density Functional Package. *Theoretical Chemistry Accounts: Theory, Computation, and Modeling (Theoretica Chimica Acta)* **1999**, *101* (6), 396–408. https://doi.org/10.1007/s002140050457.
